# Supplementary material for: A comparative analysis of the mobility of 45 proteins in the synaptic bouton
Source: EMBO J. 2020 Jul 6;39(16):e104596. doi: 10.15252/embj.2020104596 (PMC7429486; doi:10.15252/embj.2020104596)
Supplement: Supplementary file 1 — Appendix [file EMBJ-39-e104596-s001.pdf]

Table of Contents

Appendix Fig. S1. Correlation between the synapto-axonal distribution of proteins in FRAP experiments and in immunostainings **1**

Appendix Fig. S2. Typical super-resolution images for the different proteins analyzed here **2**

Appendix Fig. S3. Measured FRAP parameters for each individual protein **47**

β-Actin 49
α-SNAP 50
α-synuclein 51
Amphiphysin 52
AP180 53
AP2 54
Calmodulin 1 55
Clathrin light chain 56
Complexin 1 57
Complexin 2 58
CSP 59
Doc2a 60
Dynamin 1 61
Endophilin 62
Epsin 1 63
Hsc70 64
Intersectin 65
mEGFP 66
membrane mEGFP 67
Munc13a 68
Munc18a 69
NSF 70
PIP5KIgamma 71
Rab3 72
Rab5 73
Rab7 74
SCAMP 1 75
Septin 5 76
SNAP23 77
SNAP25 78
SNAP29 79
SV2B 80
Synapsin 81
Synaptogyrin 1 82
Synaptophysin 83
Synaptotagmin 1 84
Synaptotagmin 7 85
Syndapin 1 86
Syntaxin 1 87
Syntaxin 16 88
Tubulin 89
VAMP1 90
VAMP2 91
VAMP4 92
vATPase 93
vGluT1 94
Vtia1a 95

Appendix Fig. S4. Time constants and immobile fractions of analyzed proteins in axons and synapses **96**

Appendix Fig. S5. An analysis of the correlation between overexpression levels and mobility rates **97**

Table of Contents

Appendix Fig. S6. Our FRAP results reproduce previous results obtained on knock-in tagged proteins or on endogenous proteins labeled with fluorescent tags 102

Appendix Fig. S7. Protein mobility is affected by association with synaptic vesicles 103

Appendix Fig. S8. Comparison of the axonal time constants of vesicular and non-vesicular proteins 104

Appendix Fig. S9. Correlation between the time constants and the immobile fractions in axons and synapses 105

Appendix Fig. S10. An analysis of basic movement parameters of the proteins 106

Appendix Fig. S11. Correlations between FRAP parameters and the amino acid composition of the proteins 107

Appendix Fig. S12. Correlation of protein mobility parameters to different cell biology parameters 108

Appendix Fig. S13. Correlations between FRAP parameters and nucleotide composition of proteins' mRNAs 109

Appendix Fig. S14. A comparison of protein movement parameters to protein lifetimes 110

Appendix Fig. S15. FCS experiments for determining protein mobility in synapses and axons 111

Appendix Fig. S16. Calculation of protein diffusion coefficients using a simple FRAP interpretation model 112

Appendix Fig. S17. 3D model of a synapse, and synaptic parameters measured from serial section electron microscopy 113

Appendix Fig. S18. The in silico models, in combination with the available literature, suggest accurate numbers of synaptic vesicle proteins per vesicle 114

Appendix Fig. S19. The in silico models suggest accurate enrichment of the proteins in the vesicles 115

Appendix Fig. S20. The in silico models suggest accurate enrichment of soluble proteins in the synaptic vesicle cluster 116

Appendix Fig. S21. An analysis of basic movement parameters of the proteins 117

Appendix Fig. S22. The shapes and colors of the proteins shown in the Movies EV1-4 118

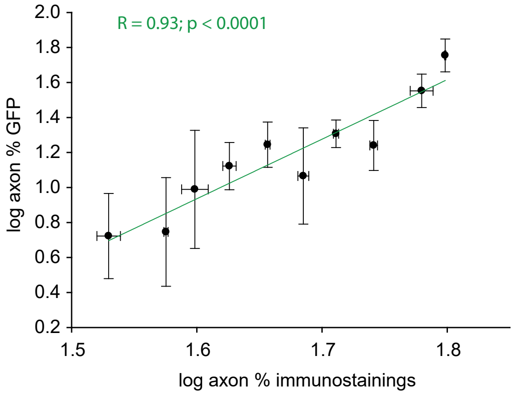

Appendix Fig. S1. Correlation between the synapto-axonal distribution of proteins in FRAP experiments and in immunostainings. We determined the fraction of each GFP-tagged protein that is found in the axon, as % of the amount found in the synapse, in every FRAP movie we performed. In separate experiments, we immunostained neurons for the proteins of interest and for synaptophysin, as a marker for the vesicle cluster, and for bassoon, as a marker for the active zone. These immunostainings (shown in Appendix Fig. S2) enabled us to determine the same parameter, the fraction of the protein found in the axon, as % of the intensity in the nearest synapses. We then compared the two measurements, in the form of a two-dimensional scatter plot. The symbols indicate the different proteins analyzed (binned, for clarity; each symbol represents the mean of 2-8 proteins, ± SEM or ± range of values).

Multi-page figure

Appendix Fig. S2. Typical super-resolution images for the different proteins analyzed here. Neurons were immunostained for the protein of interest (red), and for synaptophysin (green), as a marker for the vesicle cluster, and bassoon (blue), as a marker for the active zone (using the antibodies indicated in methods). Images were then obtained in STED mode (protein of interest) or in confocal mode (synaptophysin and bassoon). Scale bar, 5 µm.

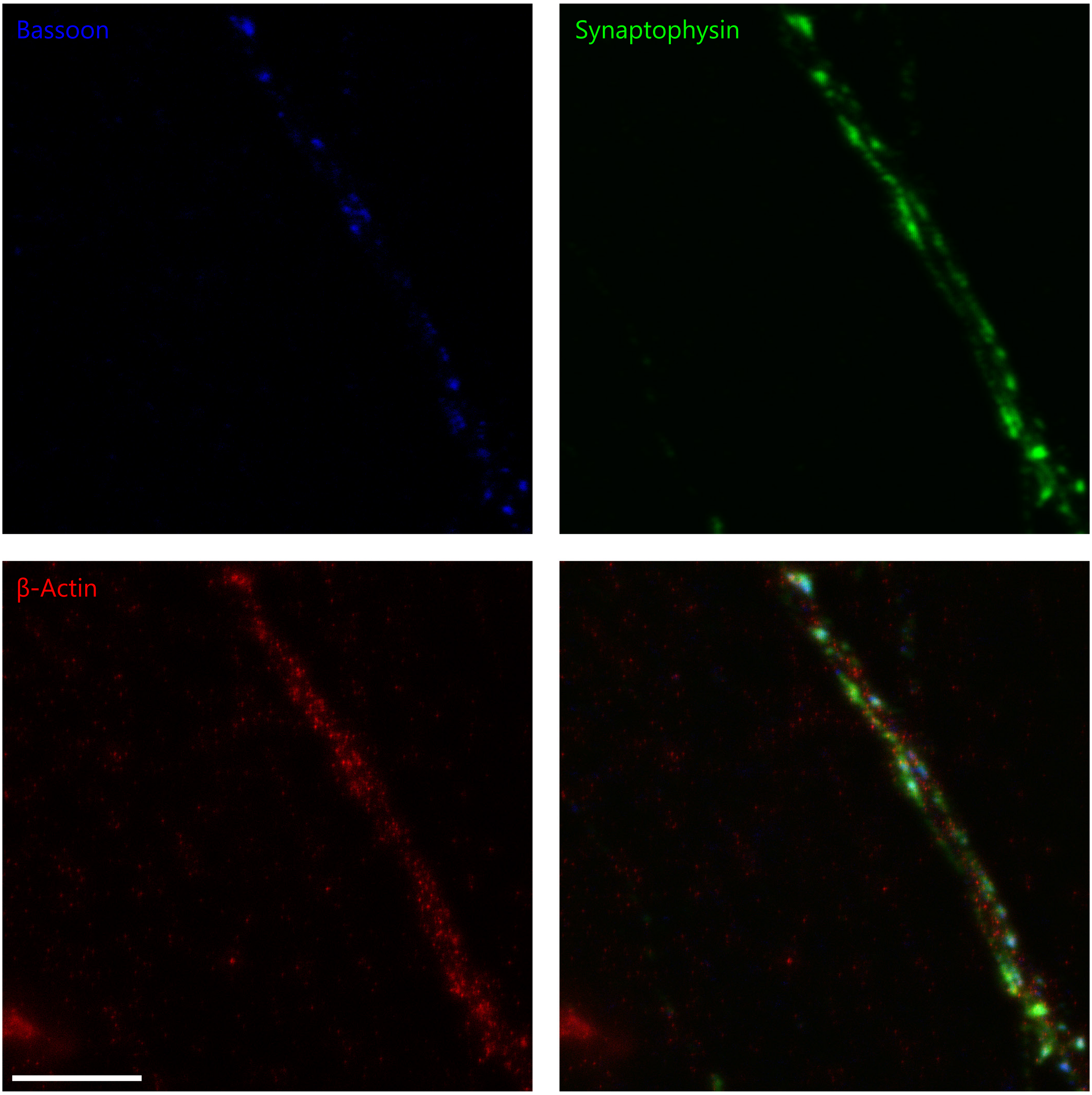

Scale bar: 5 µm

Antibodies used:
Bassoon - Synaptic Systems (Göttingen, Germany), 141 021
Synaptophysin - Synaptic Systems (Göttingen, Germany), 101 011
β-Actin - Sigma (Taufkirchen, Germany), A1978

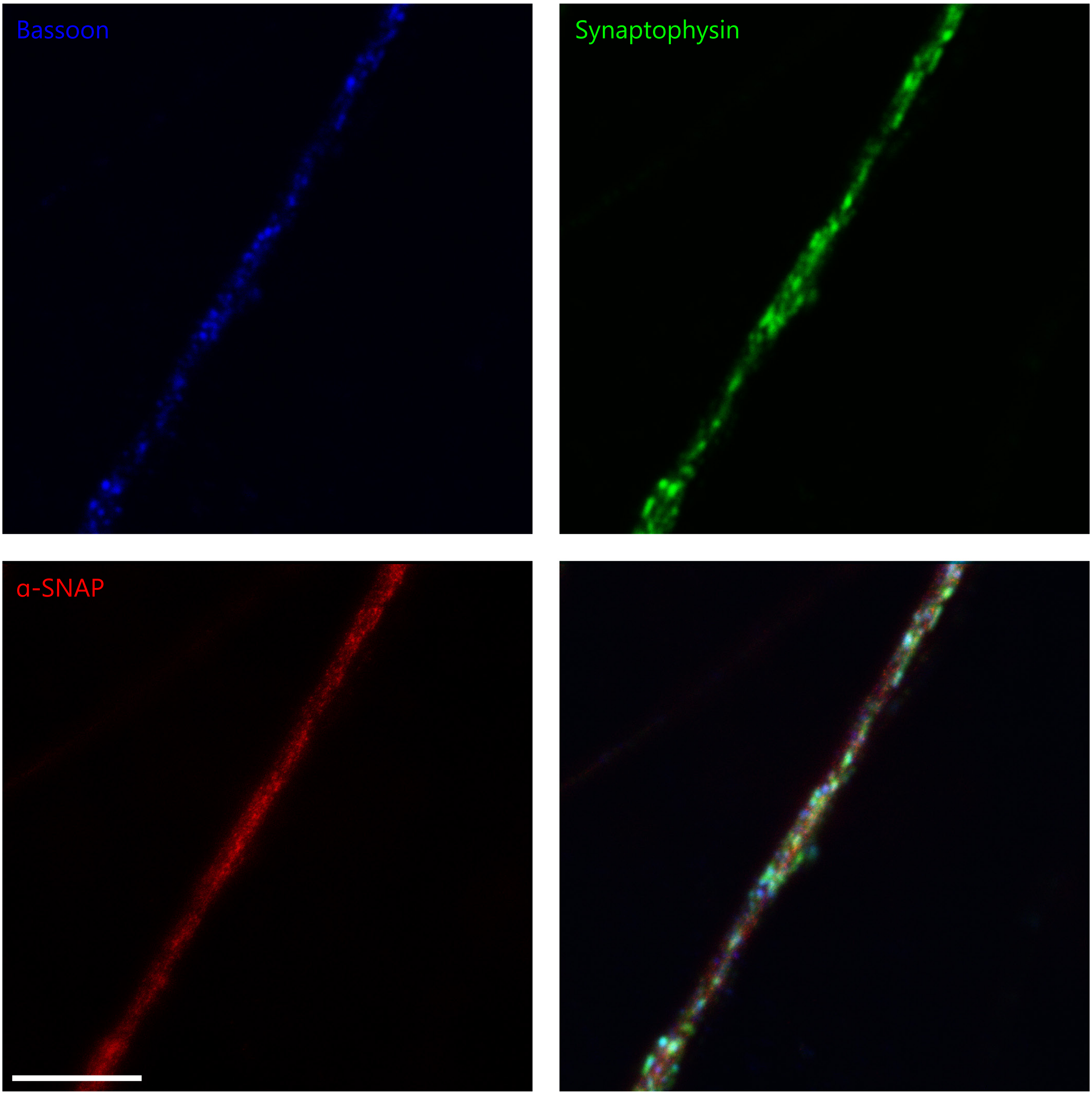

Scale bar: 5 µm

Antibodies used:
Bassoon - Synaptic Systems (Göttingen, Germany), 141 021
Synaptophysin - Synaptic Systems (Göttingen, Germany), 101 011
α-SNAP - Synaptic Systems (Göttingen, Germany), 112 111

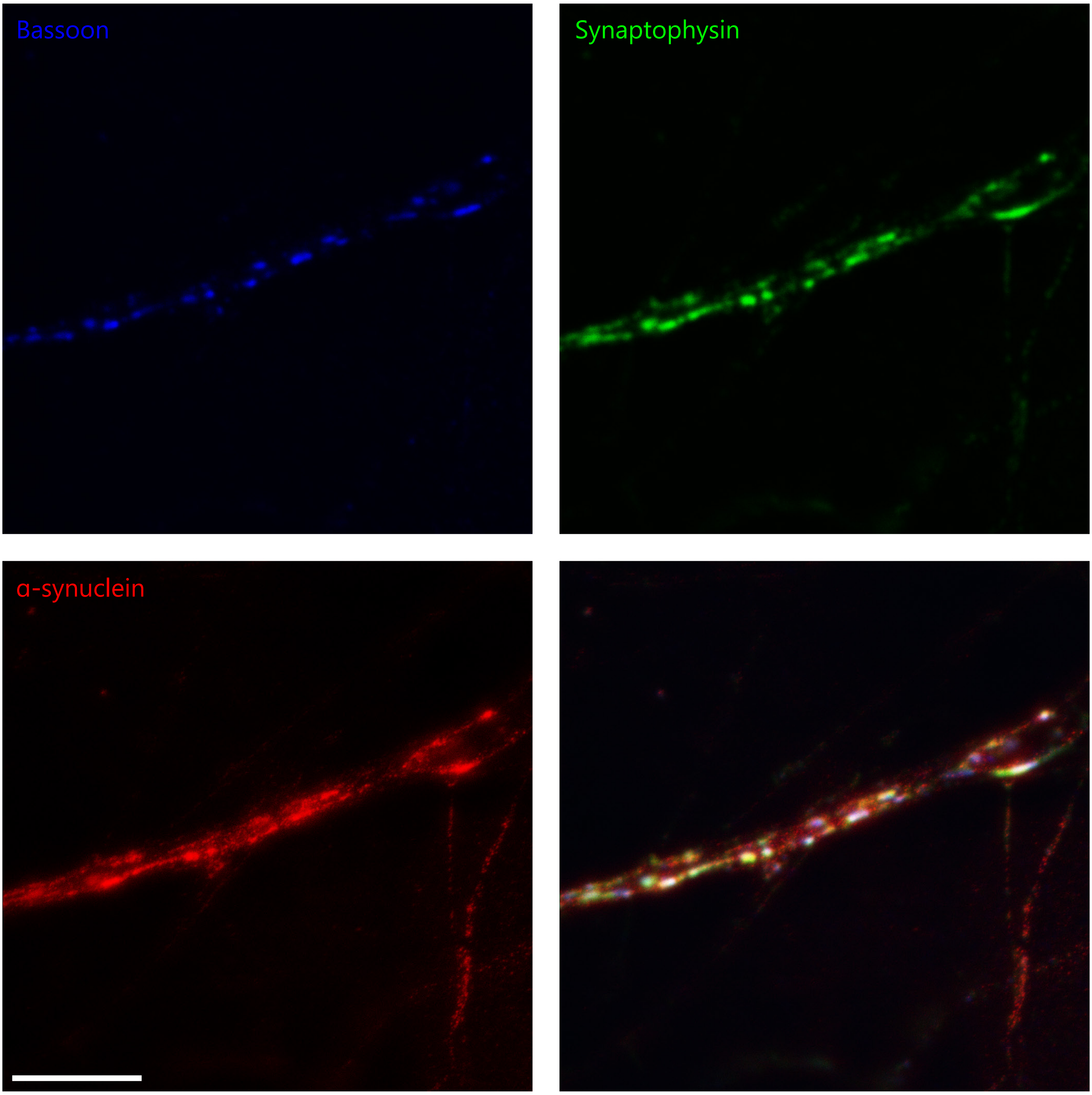

Scale bar: 5 µm

Antibodies used:
Bassoon - Synaptic Systems (Göttingen, Germany), 141 021
Synaptophysin - Synaptic Systems (Göttingen, Germany), 101 011
α-synuclein - Synaptic Systems (Göttingen, Germany), 128 002

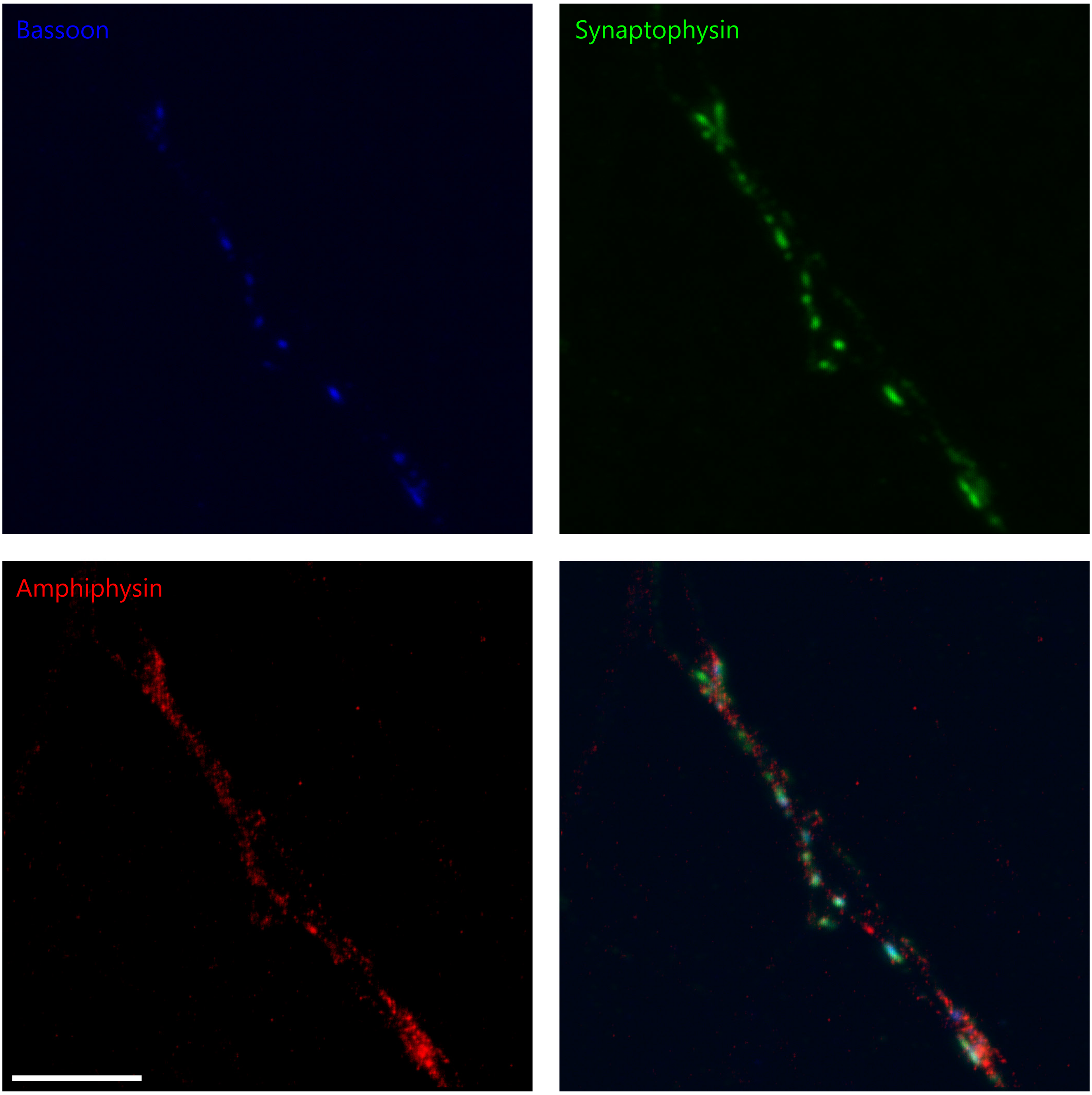

Scale bar: 5 μm

Antibodies used:
Bassoon - Synaptic Systems (Göttingen, Germany), 141 021
Synaptophysin - Synaptic Systems (Göttingen, Germany), 101 011
Amphiphysin - Synaptic Systems (Göttingen, Germany), 120 002

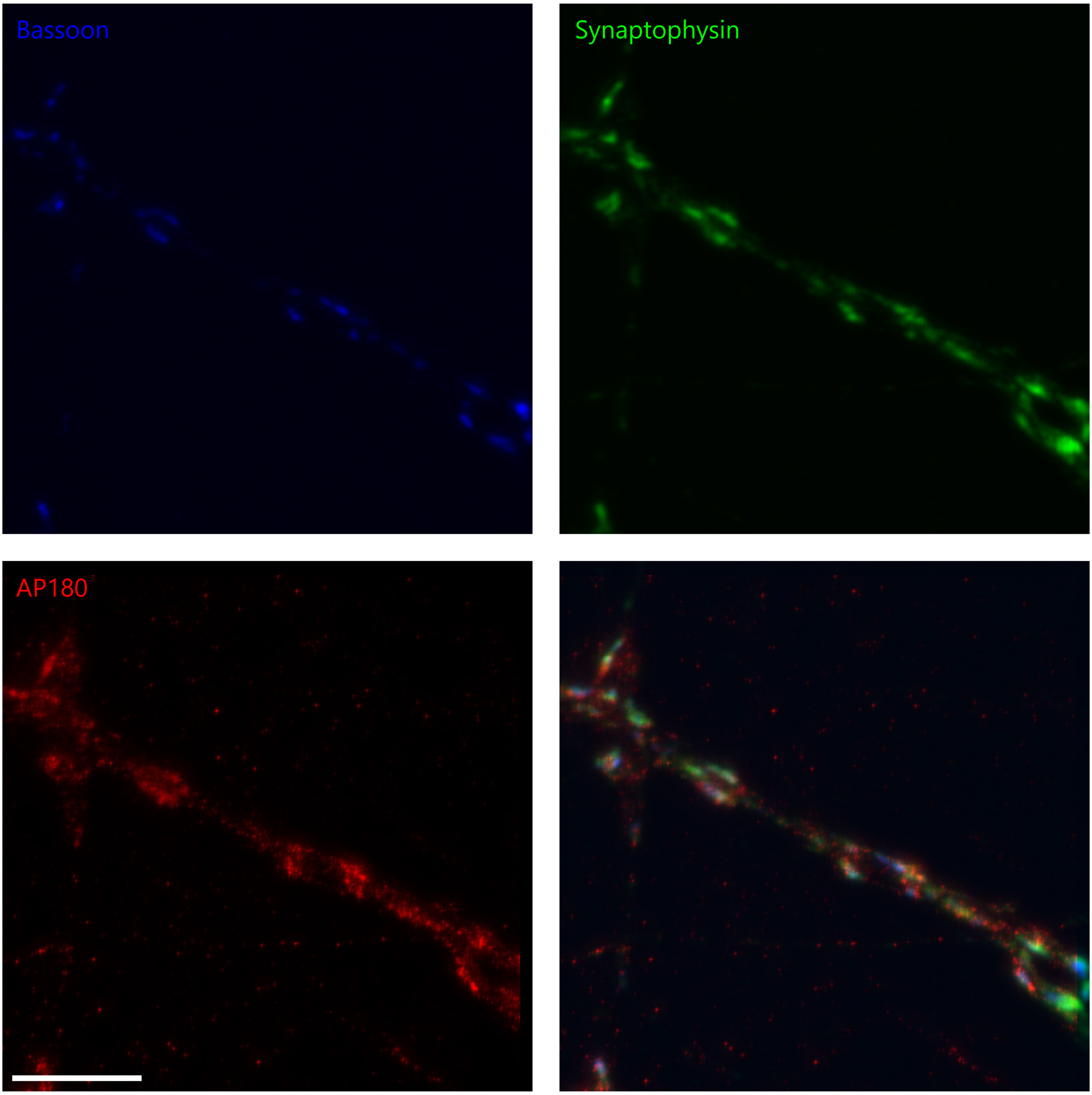

Scale bar: 5 µm

Antibodies used:
Bassoon - Synaptic Systems (Göttingen, Germany), 141 021
Synaptophysin - Synaptic Systems (Göttingen, Germany), 101 011
AP180 - Synaptic Systems (Göttingen, Germany), 155 003

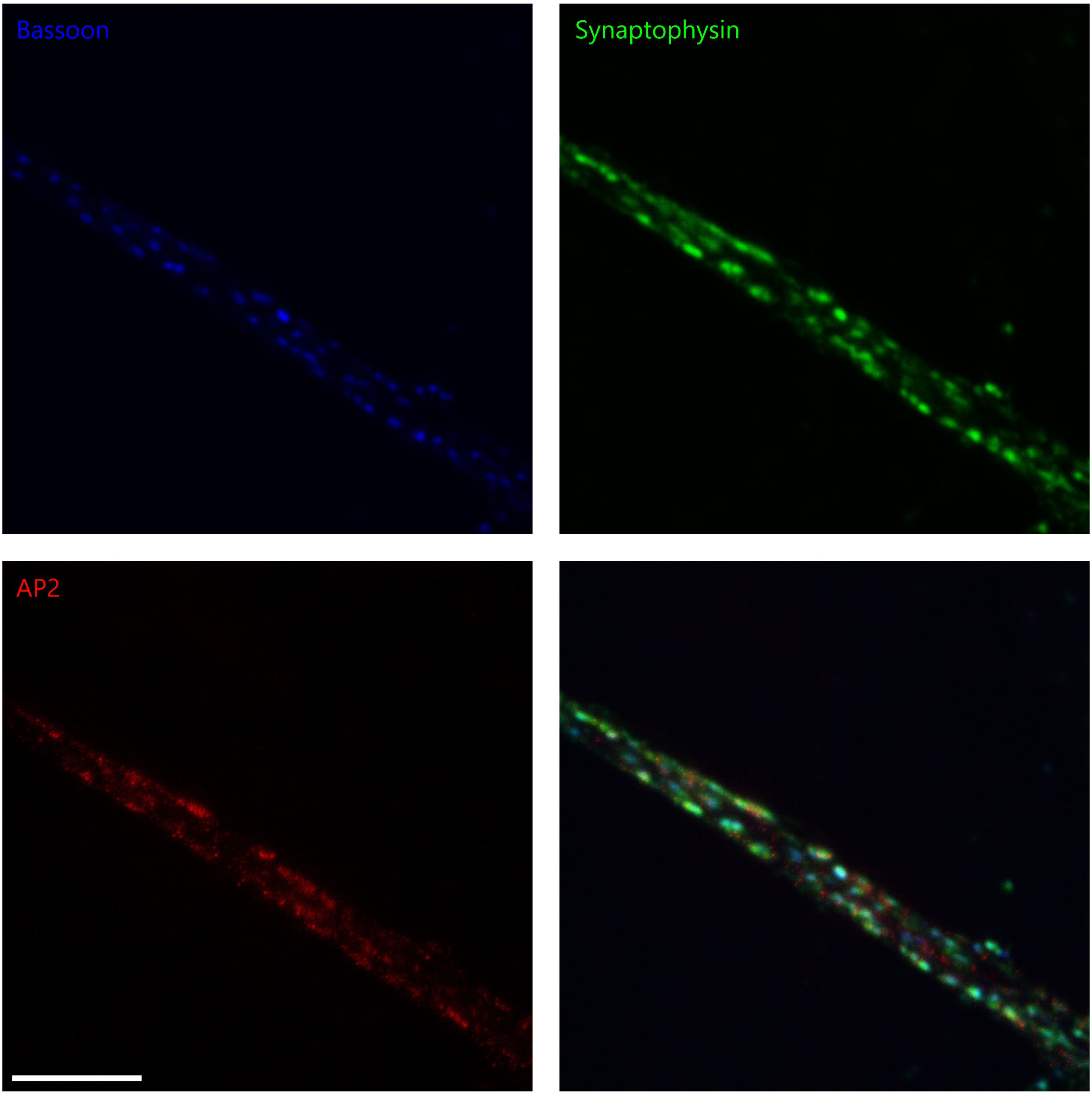

Scale bar: 5 µm

Antibodies used:
Bassoon - Synaptic Systems (Göttingen, Germany), 141 021
Synaptophysin - Synaptic Systems (Göttingen, Germany), 101 011
AP2 - Santa Cruz (Heidelberg, Germany), sc-99026

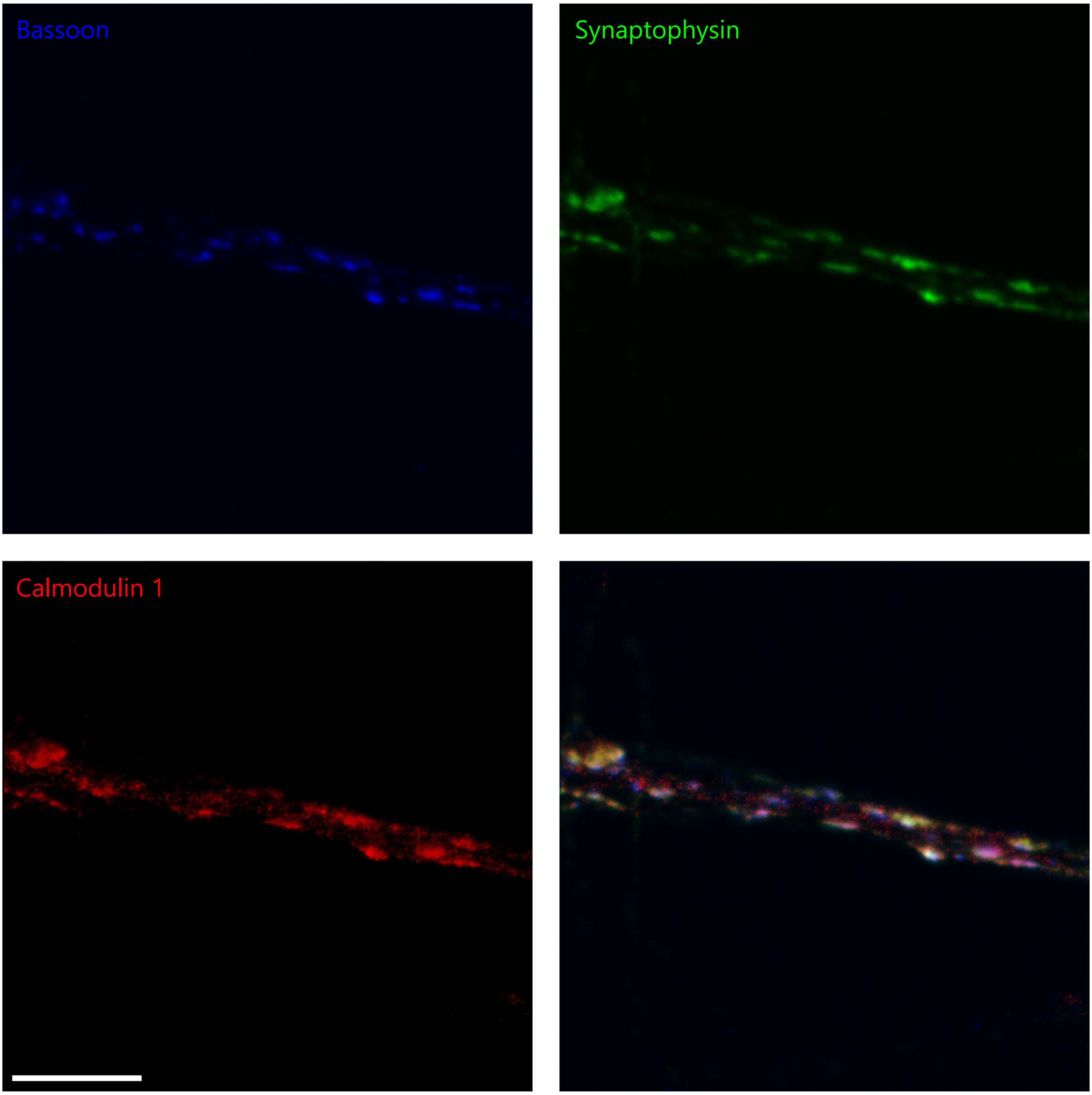

Scale bar: 5 µm

Antibodies used:
Bassoon - Synaptic Systems (Göttingen, Germany), 141 021
Synaptophysin - Synaptic Systems (Göttingen, Germany), 101 011
Calmodulin 1 - Novus Biologicals (Littleton, Colorado, USA), NB110-55649

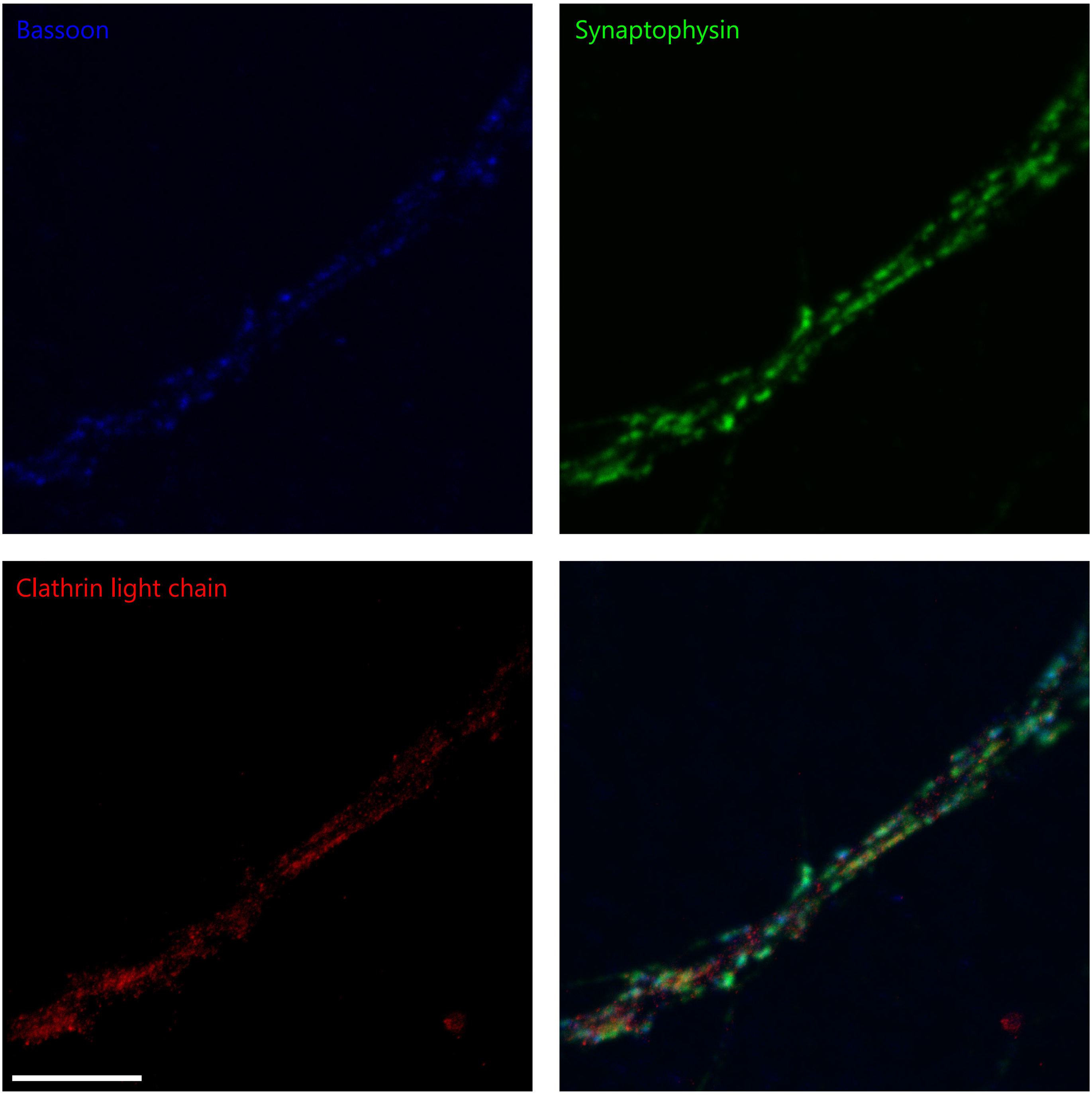

Scale bar: 5 µm

Antibodies used:
Bassoon - Synaptic Systems (Göttingen, Germany), 141 021
Synaptophysin - Synaptic Systems (Göttingen, Germany), 101 011
Clathrin light chain - Synaptic Systems (Göttingen, Germany), 113 001

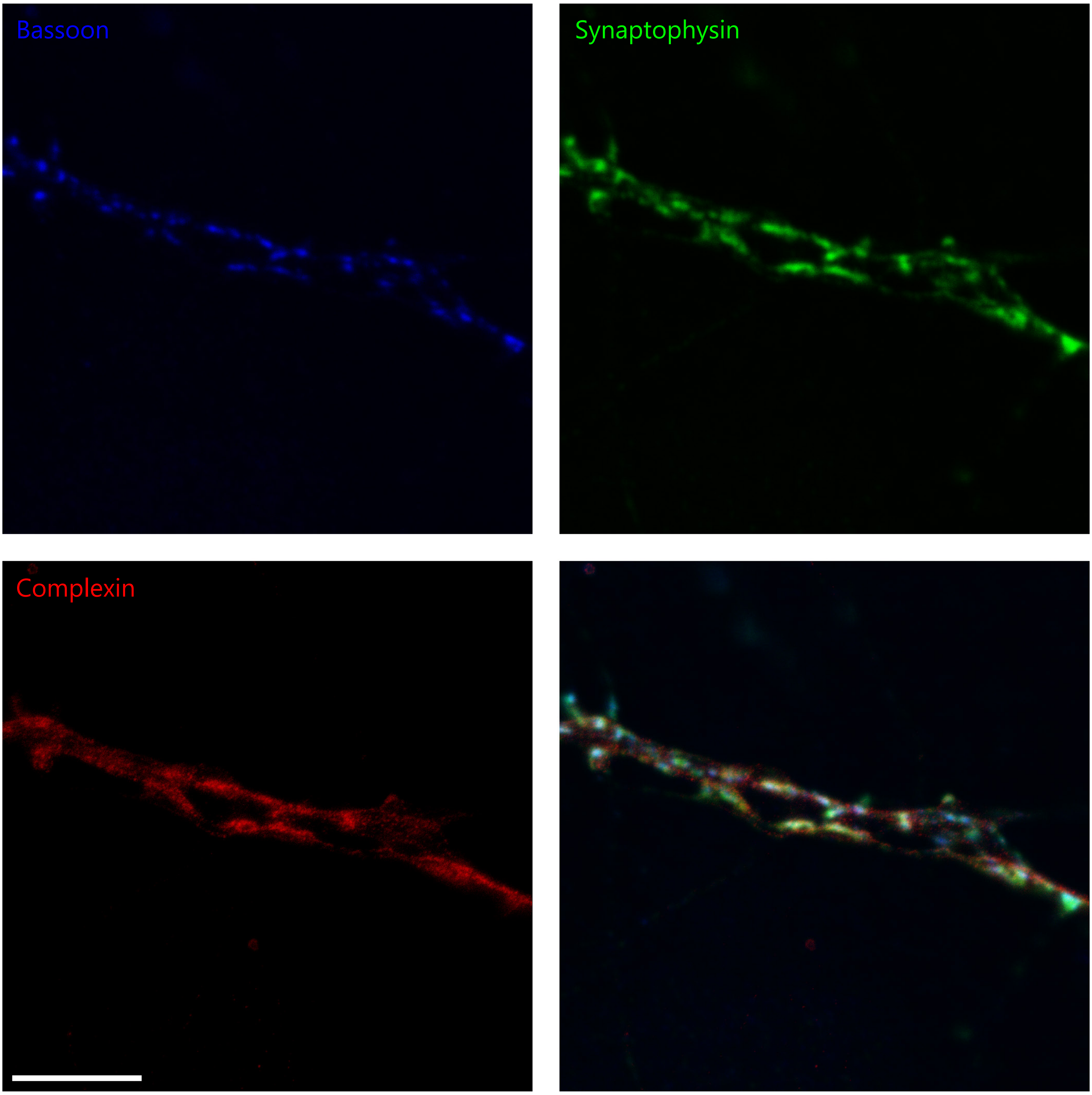

Scale bar: 5 µm

Antibodies used:
Bassoon - Synaptic Systems (Göttingen, Germany), 141 021
Synaptophysin - Synaptic Systems (Göttingen, Germany), 101 011
Complexin - Synaptic Systems (Göttingen, Germany), 122 002

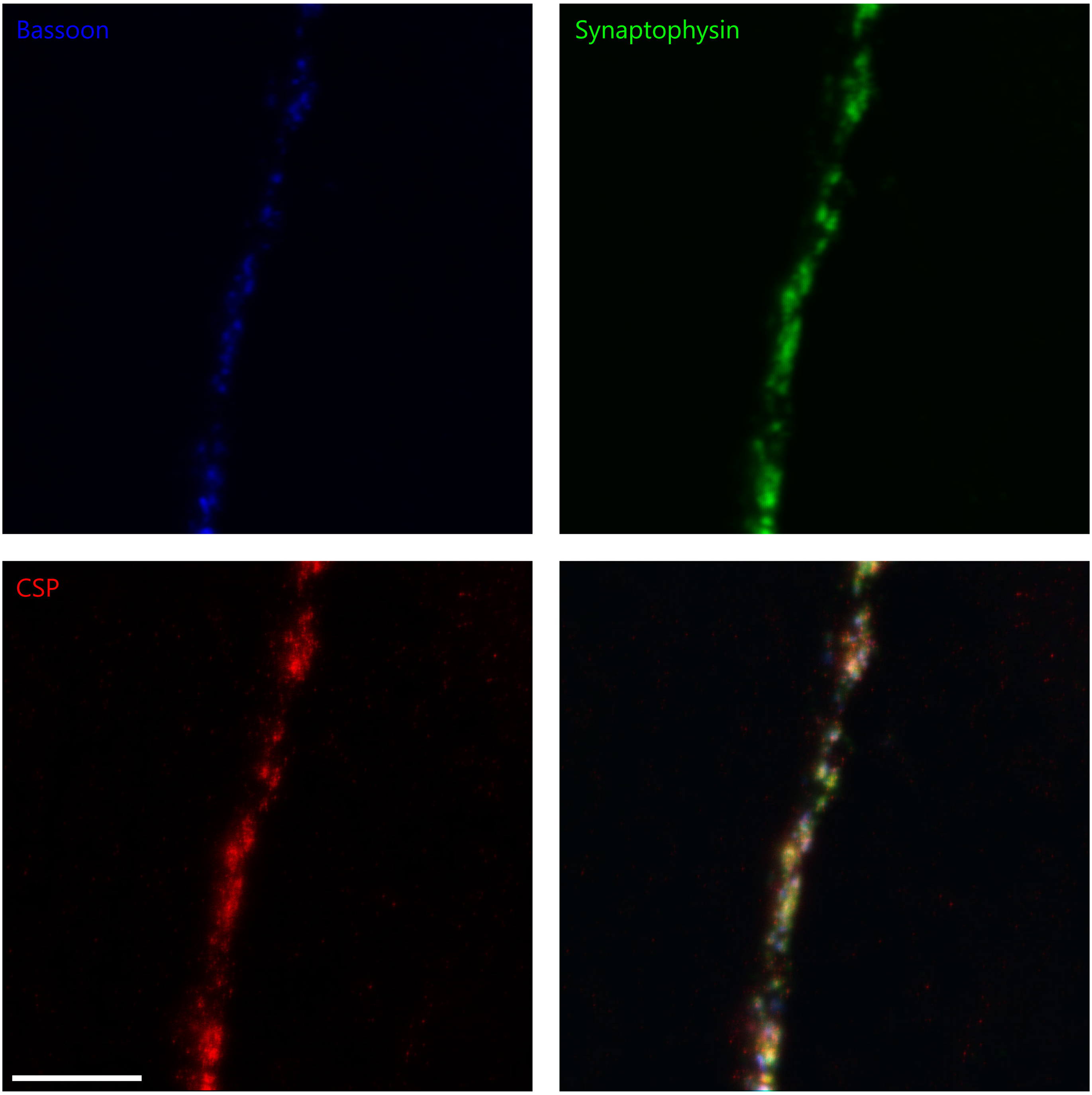

Scale bar: 5 µm

Antibodies used:
Bassoon - Synaptic Systems (Göttingen, Germany), 141 021
Synaptophysin - Synaptic Systems (Göttingen, Germany), 101 011
CSP - Synaptic Systems (Göttingen, Germany), 154 003

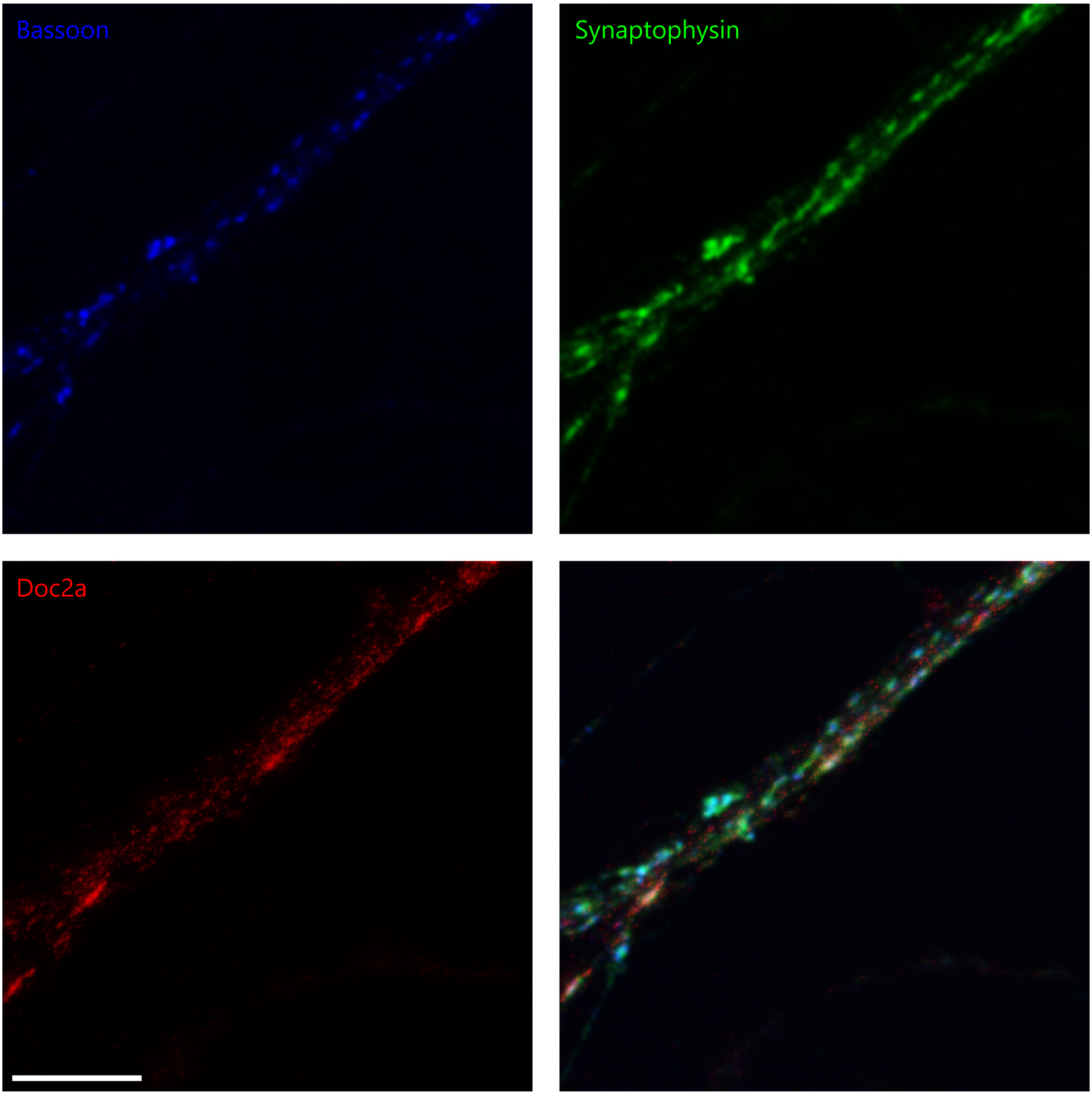

Scale bar: 5 µm

Antibodies used:
Bassoon - Synaptic Systems (Göttingen, Germany), 141 021
Synaptophysin - Synaptic Systems (Göttingen, Germany), 101 011
Doc2a - Synaptic Systems (Göttingen, Germany), 174 203

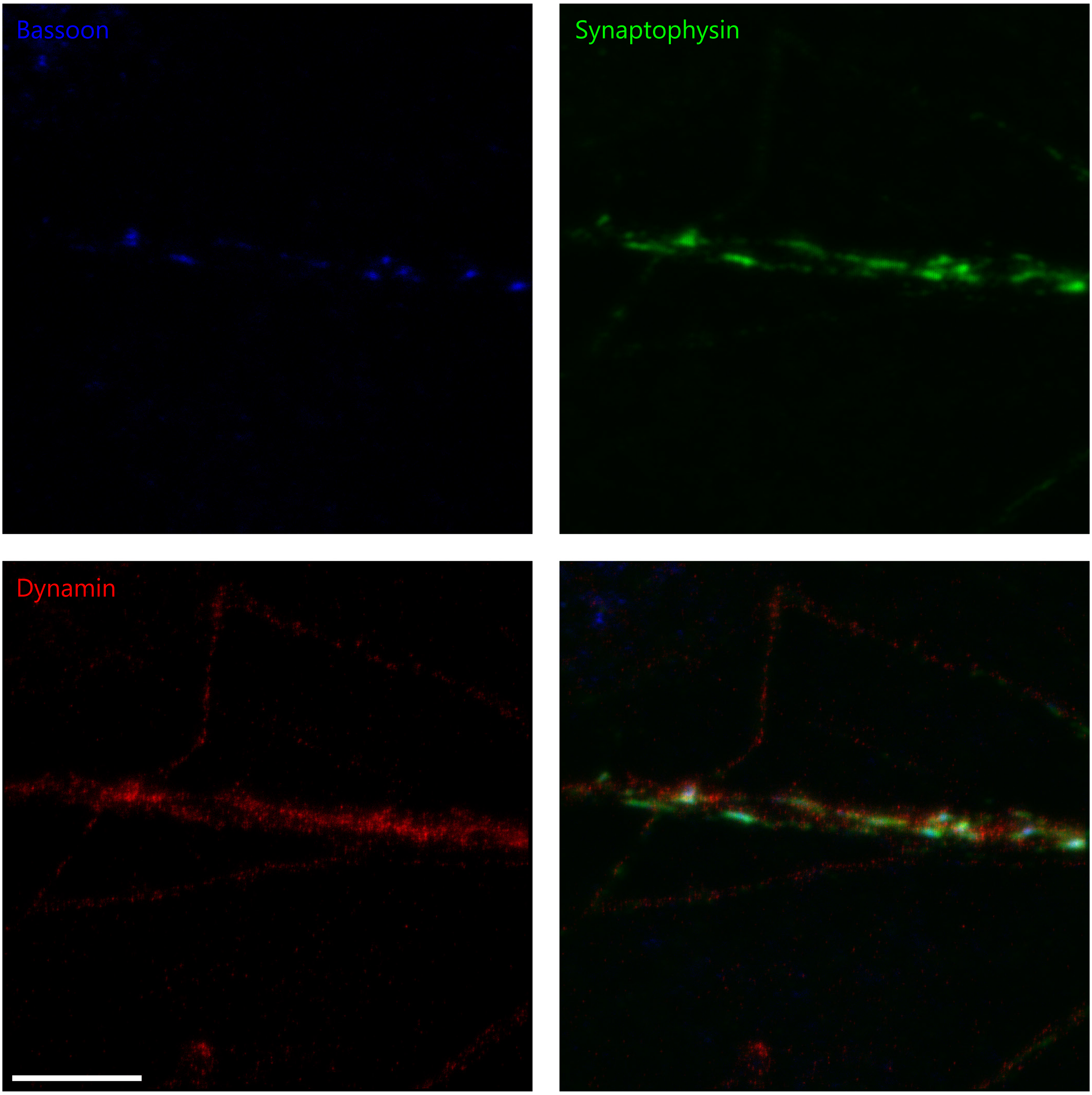

Scale bar: 5 µm

Antibodies used:
Bassoon - Synaptic Systems (Göttingen, Germany), 141 021
Synaptophysin - Synaptic Systems (Göttingen, Germany), 101 011
Dynamin - BD Biosciences (Heidelberg, Germany), 610245

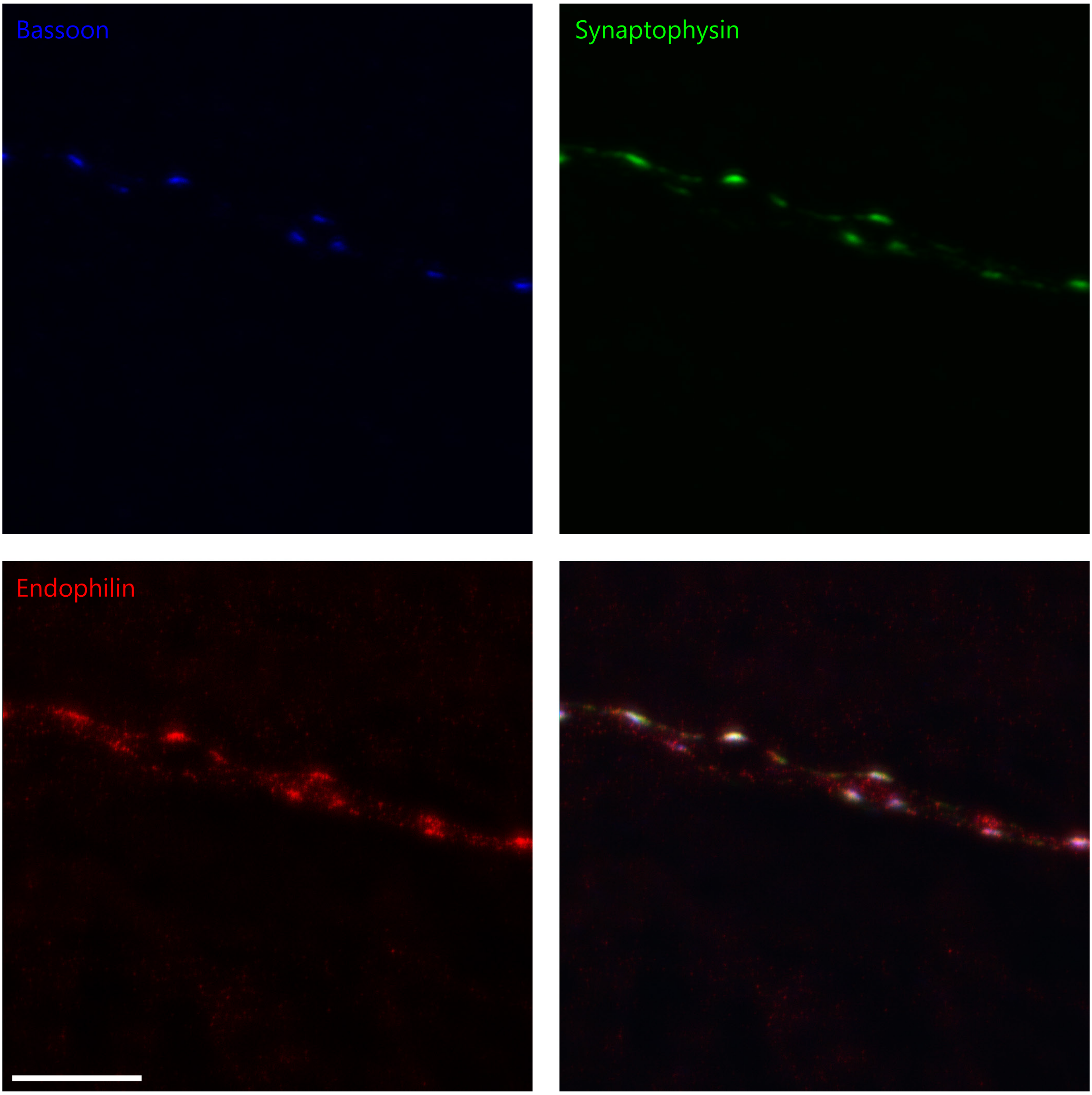

Scale bar: 5 µm

Antibodies used:
Bassoon - Synaptic Systems (Göttingen, Germany), 141 021
Synaptophysin - Synaptic Systems (Göttingen, Germany), 101 011
Endophilin - Synaptic Systems (Göttingen, Germany), 159 002

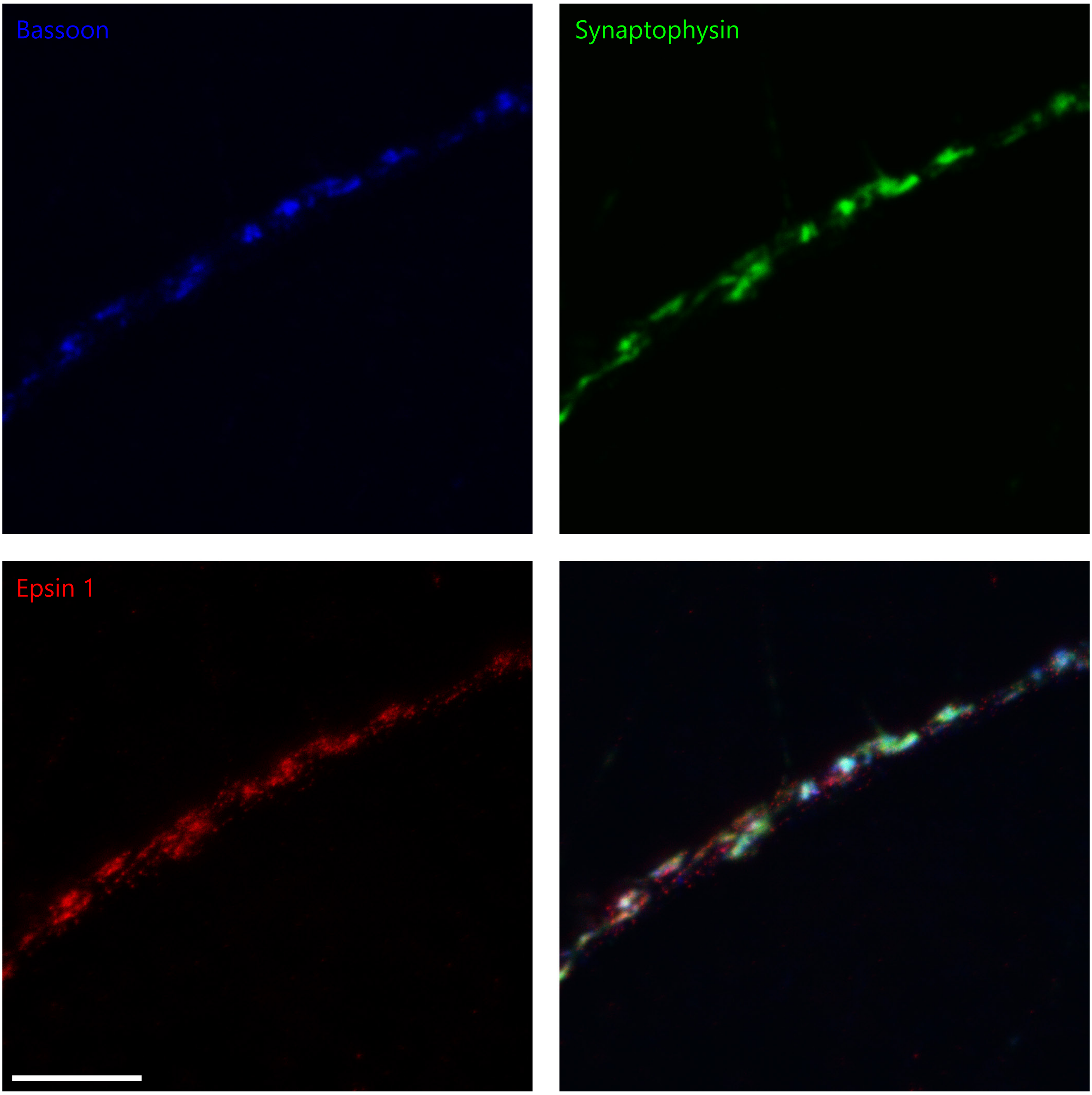

Scale bar: 5 µm

Antibodies used:
Bassoon - Synaptic Systems (Göttingen, Germany), 141 021
Synaptophysin - Synaptic Systems (Göttingen, Germany), 101 011
Epsin 1 - Novus Biologicals (Littleton, Colorado, USA), NBP1-40602

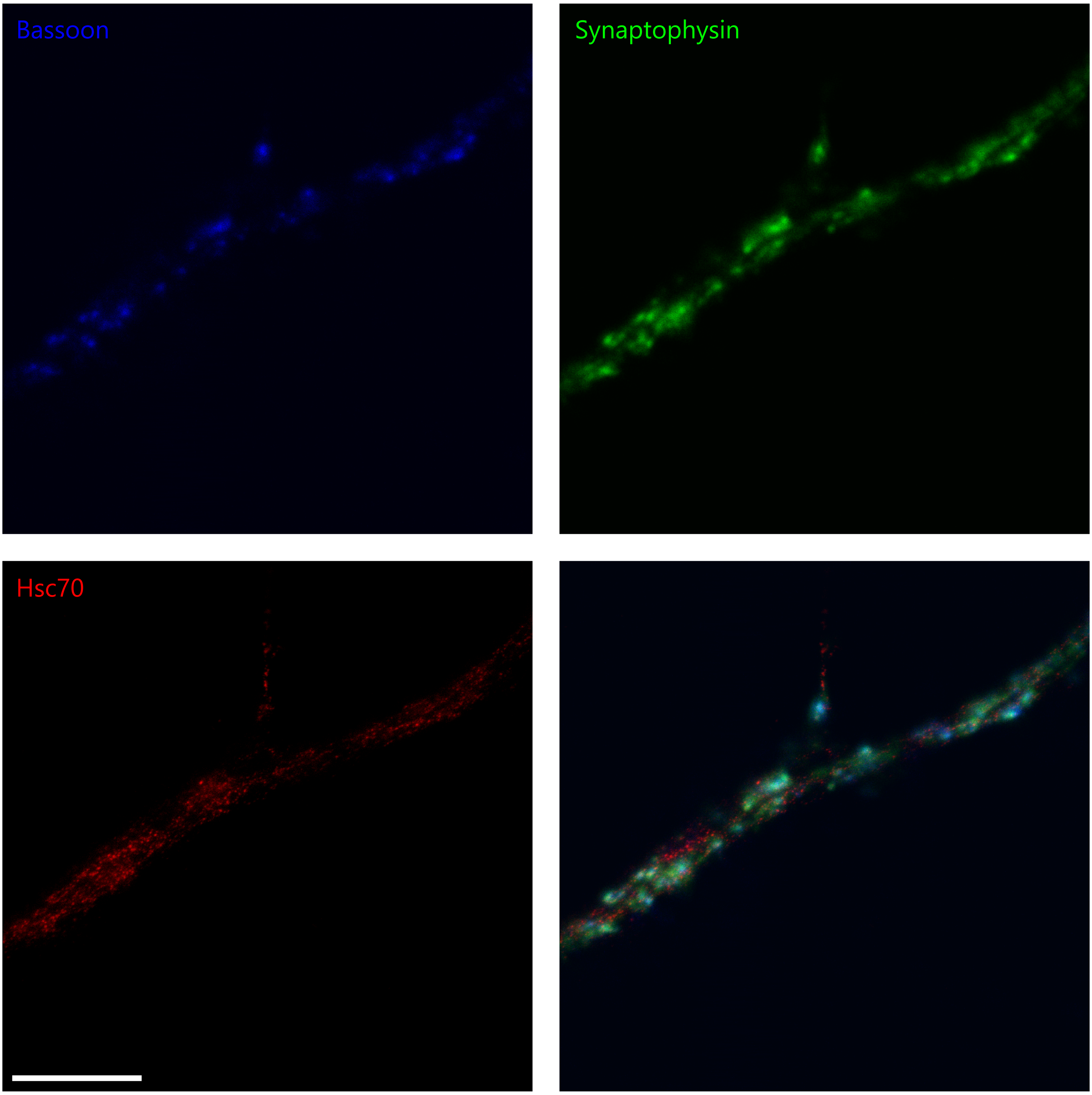

Scale bar: 5 µm

Antibodies used:
Bassoon - Synaptic Systems (Göttingen, Germany), 141 021
Synaptophysin - Synaptic Systems (Göttingen, Germany), 101 011
Hsc70 - Santa Cruz (Heidelberg, Germany), sc-7298

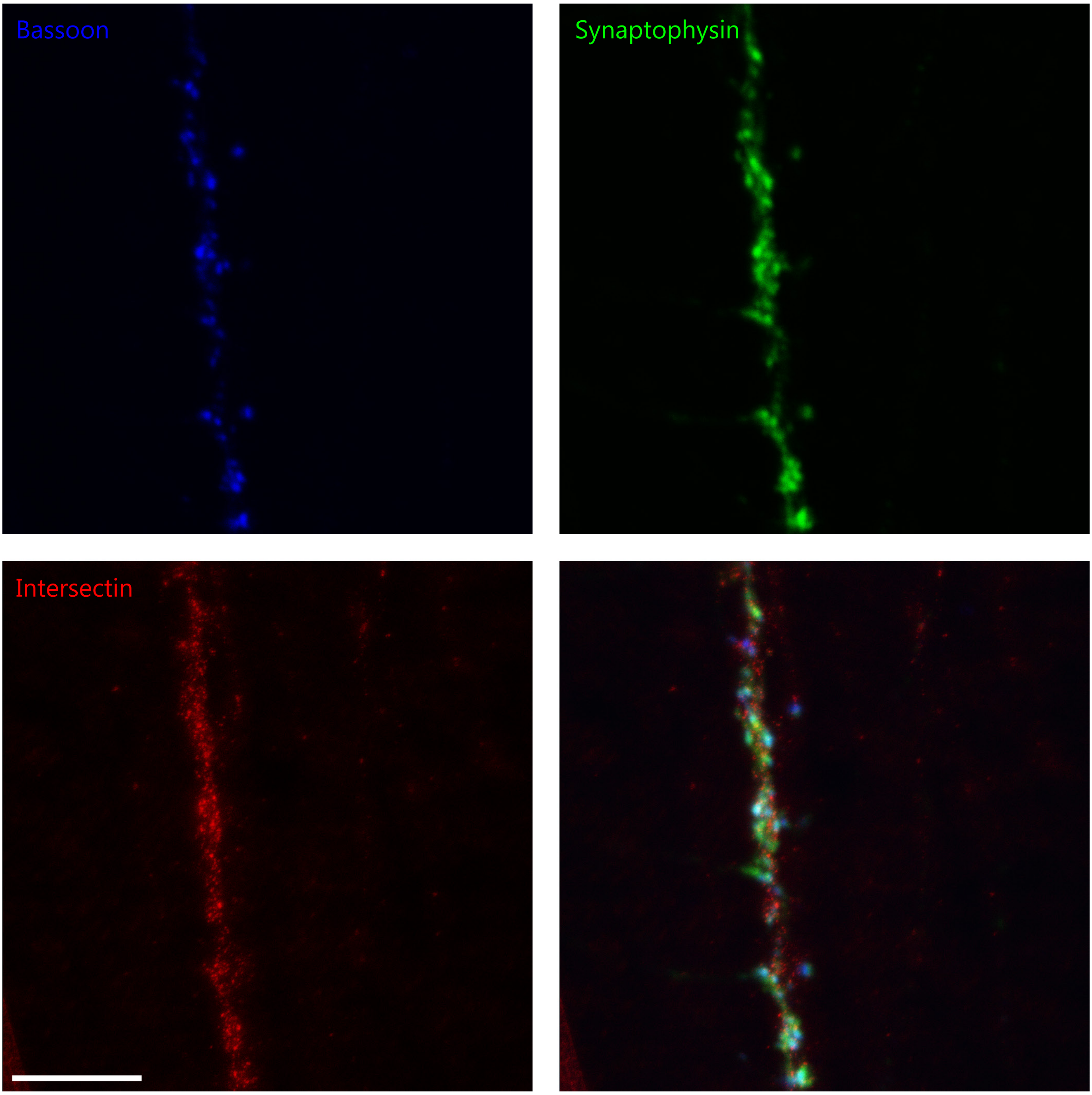

Scale bar: 5 µm

Antibodies used:
Bassoon - Synaptic Systems (Göttingen, Germany), 141 021
Synaptophysin - Synaptic Systems (Göttingen, Germany), 101 011
Intersectin - Volker Haucke (FMP, Berlin, Germany)

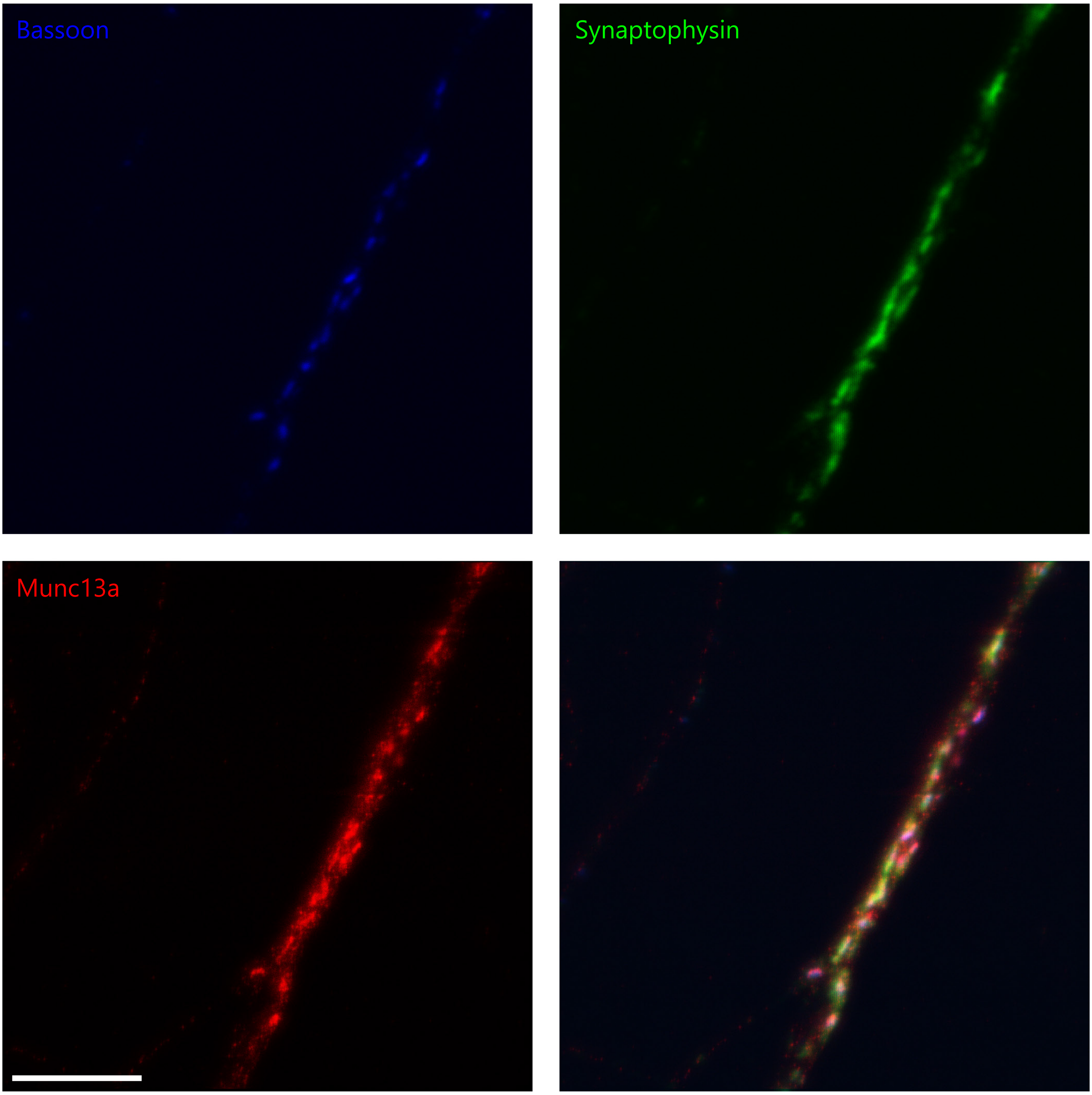

Scale bar: 5 µm

Antibodies used:
Bassoon - Synaptic Systems (Göttingen, Germany), 141 021
Synaptophysin - Synaptic Systems (Göttingen, Germany), 101 011
Munc13a - Antibodies-Online (Aachen, Germany), ABIN571921

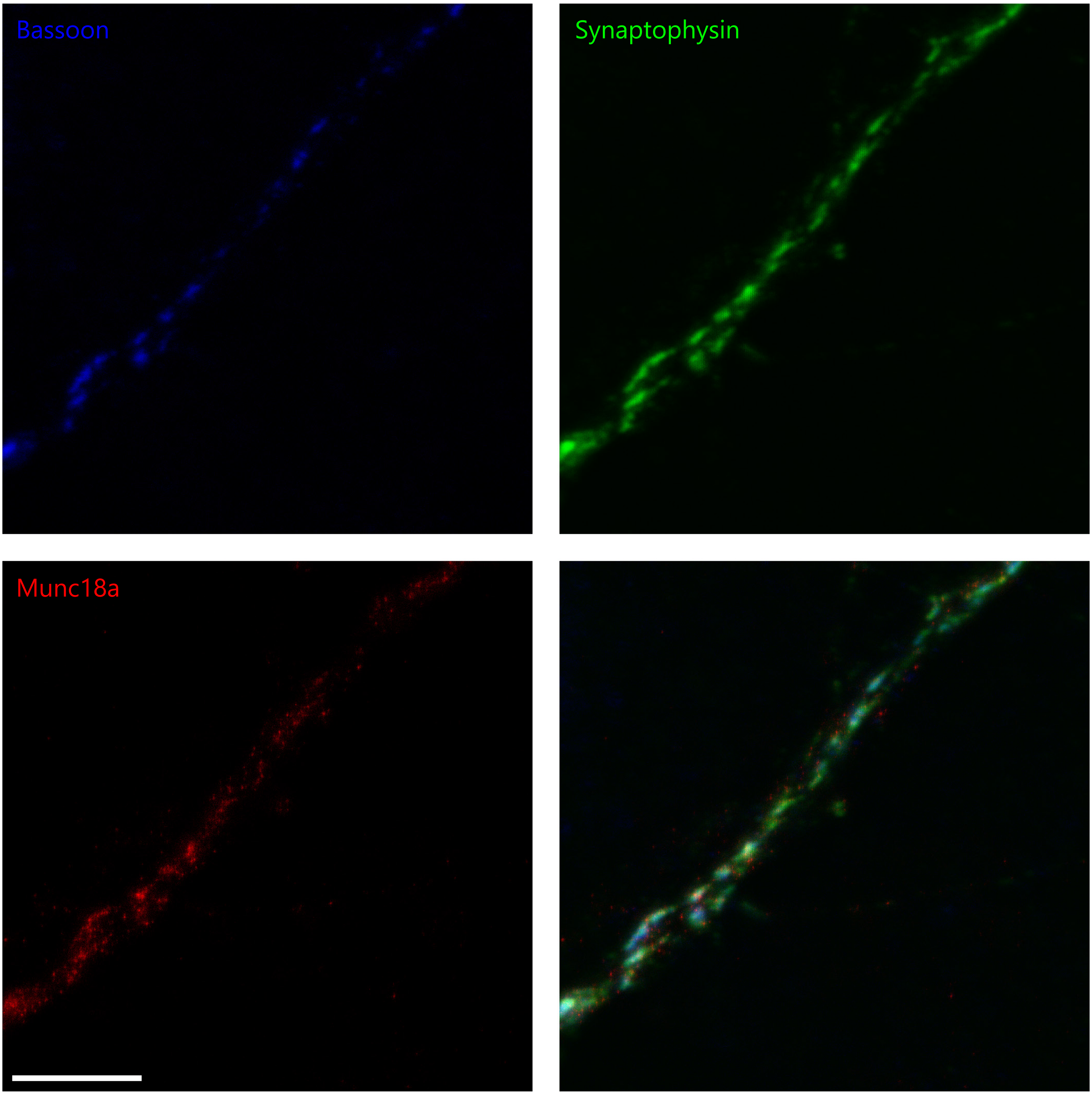

Scale bar: 5 µm

Antibodies used:
Bassoon - Synaptic Systems (Göttingen, Germany), 141 021
Synaptophysin - Synaptic Systems (Göttingen, Germany), 101 011
Munc18a - BD Biosciences (Heidelberg, Germany), 610336

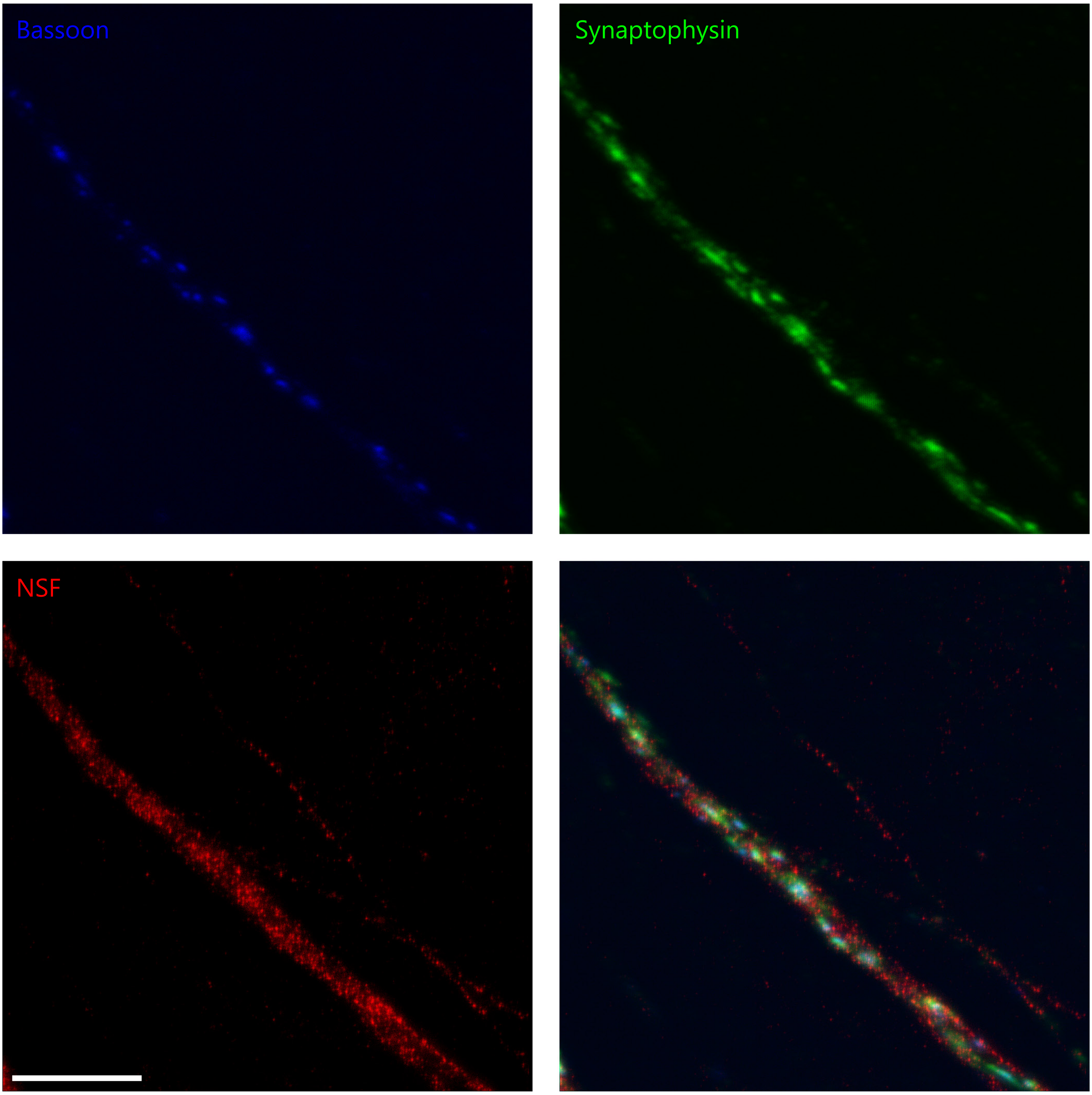

Scale bar: 5 µm

Antibodies used:
Bassoon - Synaptic Systems (Göttingen, Germany), 141 021
Synaptophysin - Synaptic Systems (Göttingen, Germany), 101 011
NSF - Synaptic Systems (Göttingen, Germany), 123 002

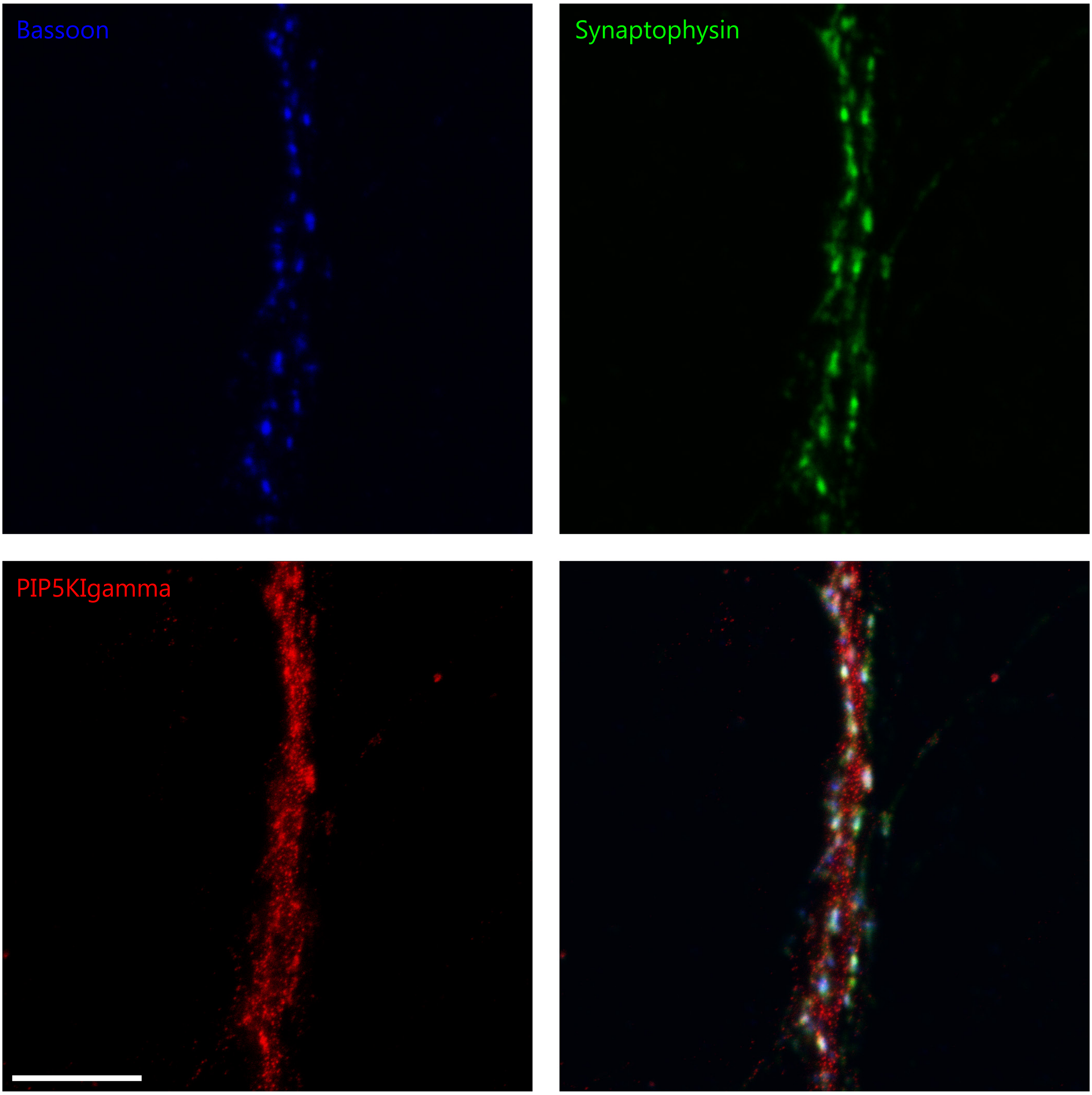

Scale bar: 5 µm

Antibodies used:
Bassoon - Synaptic Systems (Göttingen, Germany), 141 021
Synaptophysin - Synaptic Systems (Göttingen, Germany), 101 011
PIP5KIgamma - Volker Haucke (FMP, Berlin, Germany)

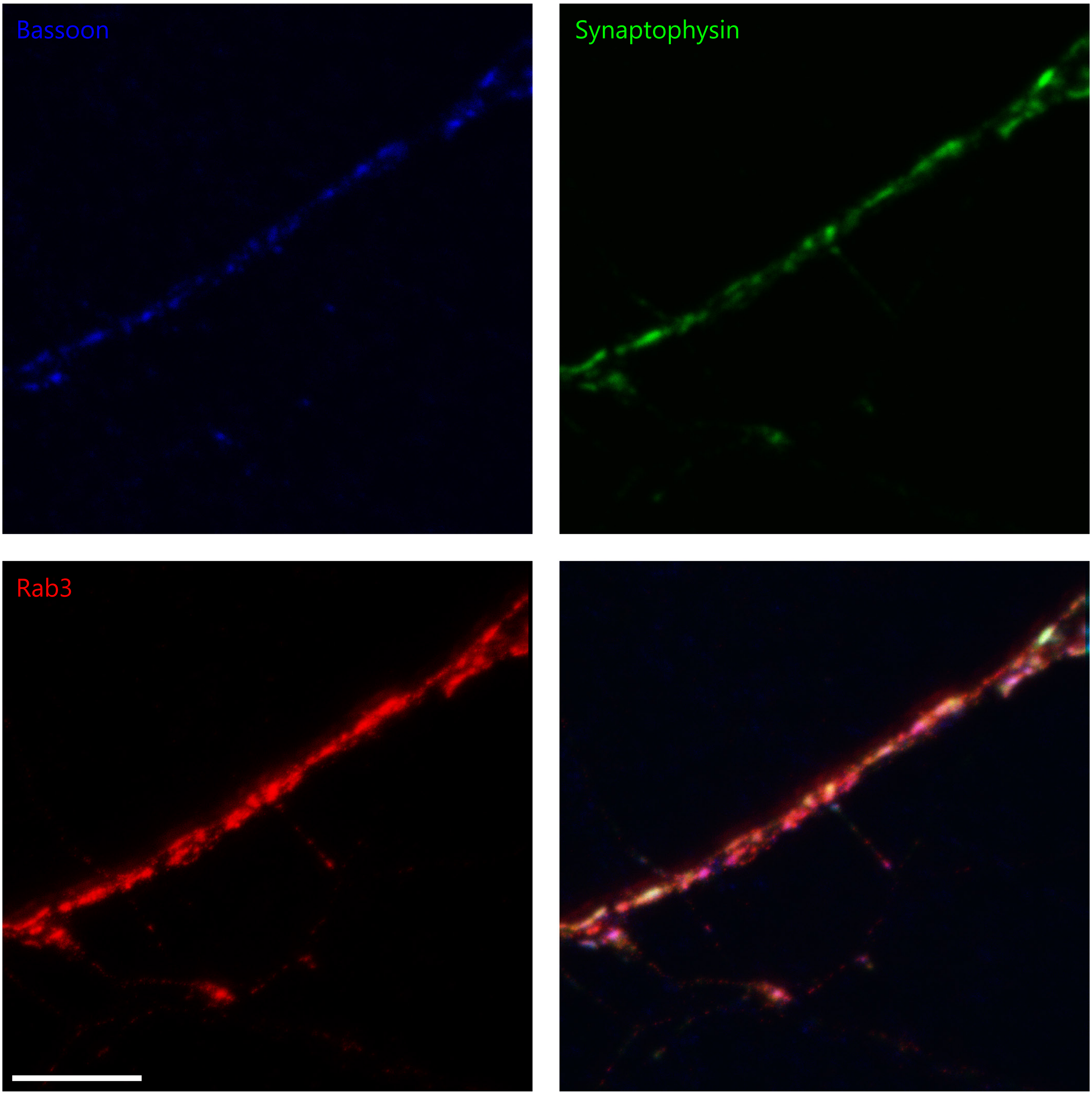

Scale bar: 5 µm

Antibodies used:
Bassoon - Synaptic Systems (Göttingen, Germany), 141 021
Synaptophysin - Synaptic Systems (Göttingen, Germany), 101 011
Rab3 - BD Biosciences (Heidelberg, Germany), 610379

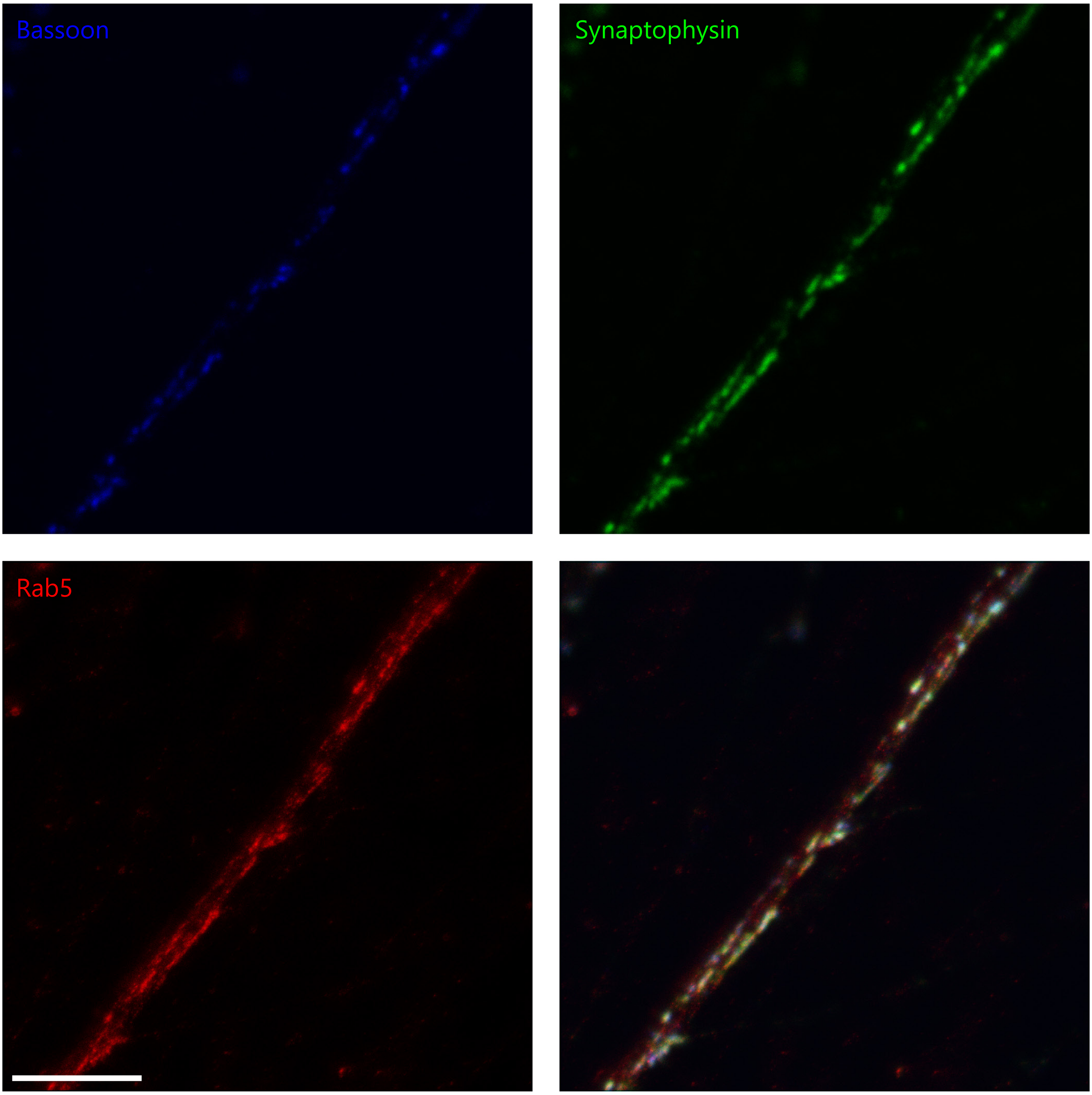

Scale bar: 5 μm

Antibodies used:
Bassoon - Synaptic Systems (Göttingen, Germany), 141 021
Synaptophysin - Synaptic Systems (Göttingen, Germany), 101 011
Rab5 - Cell Signaling Technology (Beverly, Massachusetts, USA), 3547

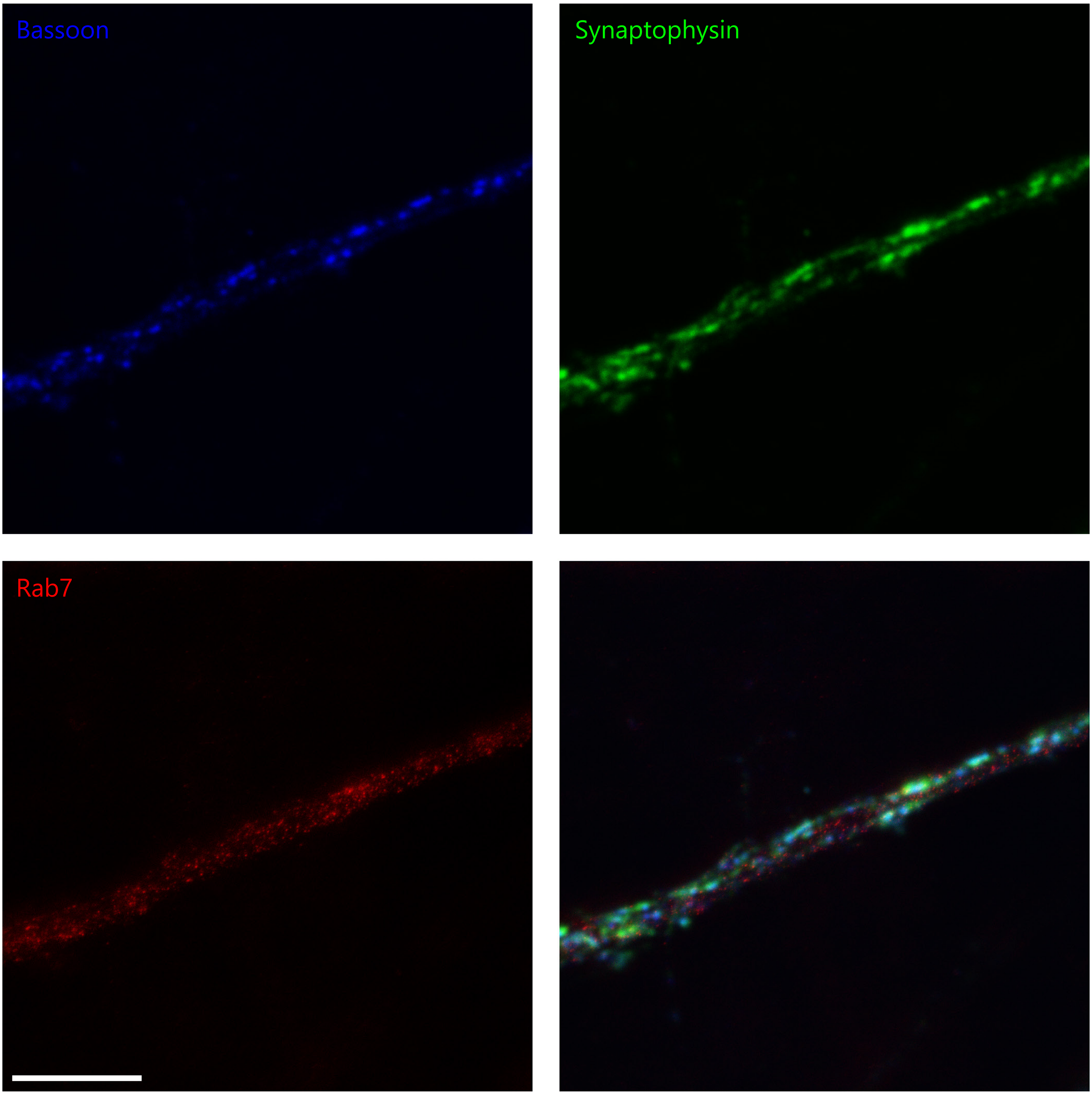

Scale bar: 5 µm

Antibodies used:
Bassoon - Synaptic Systems (Göttingen, Germany), 141 021
Synaptophysin - Synaptic Systems (Göttingen, Germany), 101 011
Rab7 - Novus Biologicals (Littleton, Colorado, USA), NBP1-05048

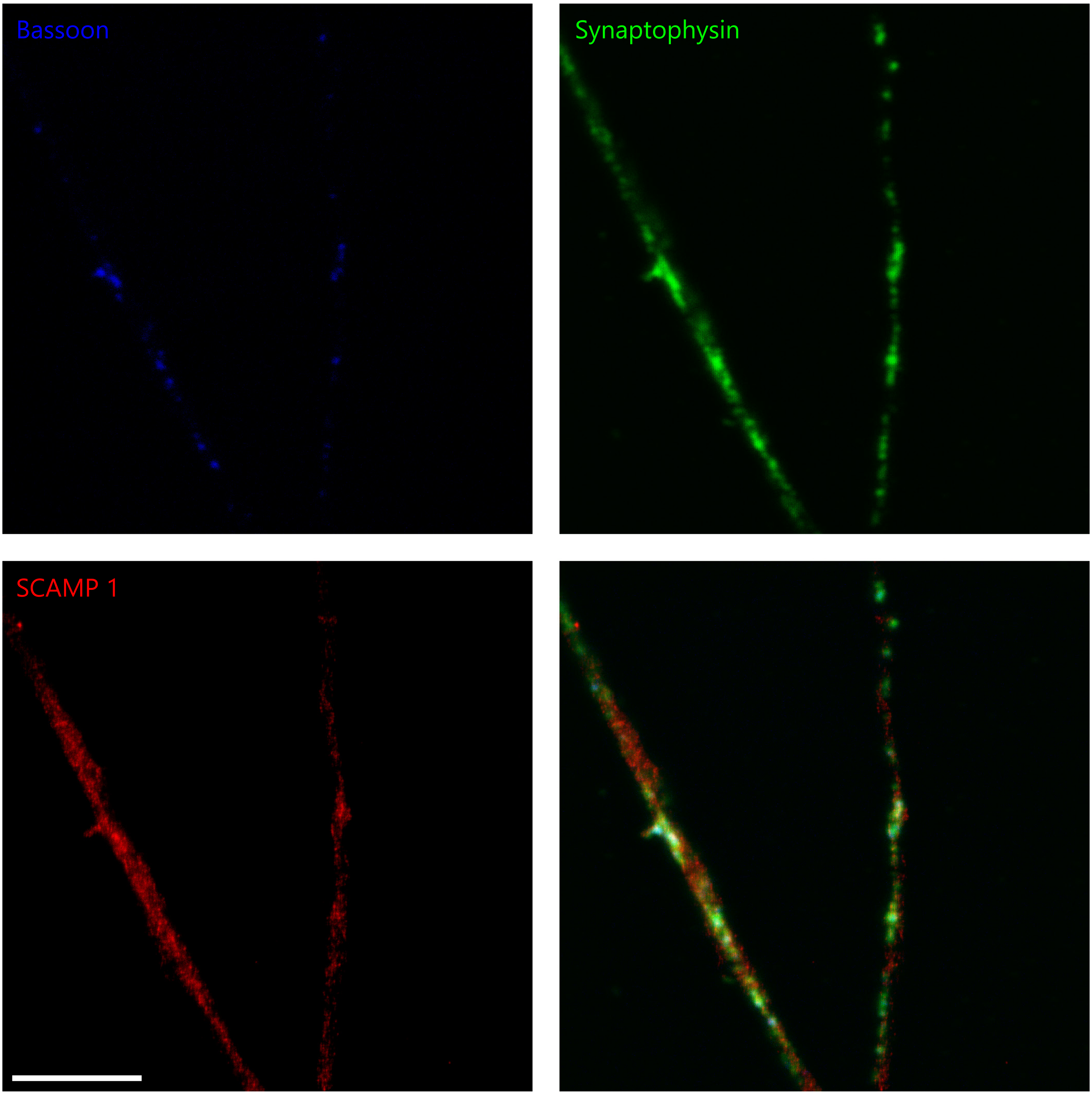

Scale bar: 5 µm

Antibodies used:
Bassoon - Synaptic Systems (Göttingen, Germany), 141 021
Synaptophysin - Synaptic Systems (Göttingen, Germany), 101 011
SCAMP 1 - Synaptic Systems (Göttingen, Germany), 121 001

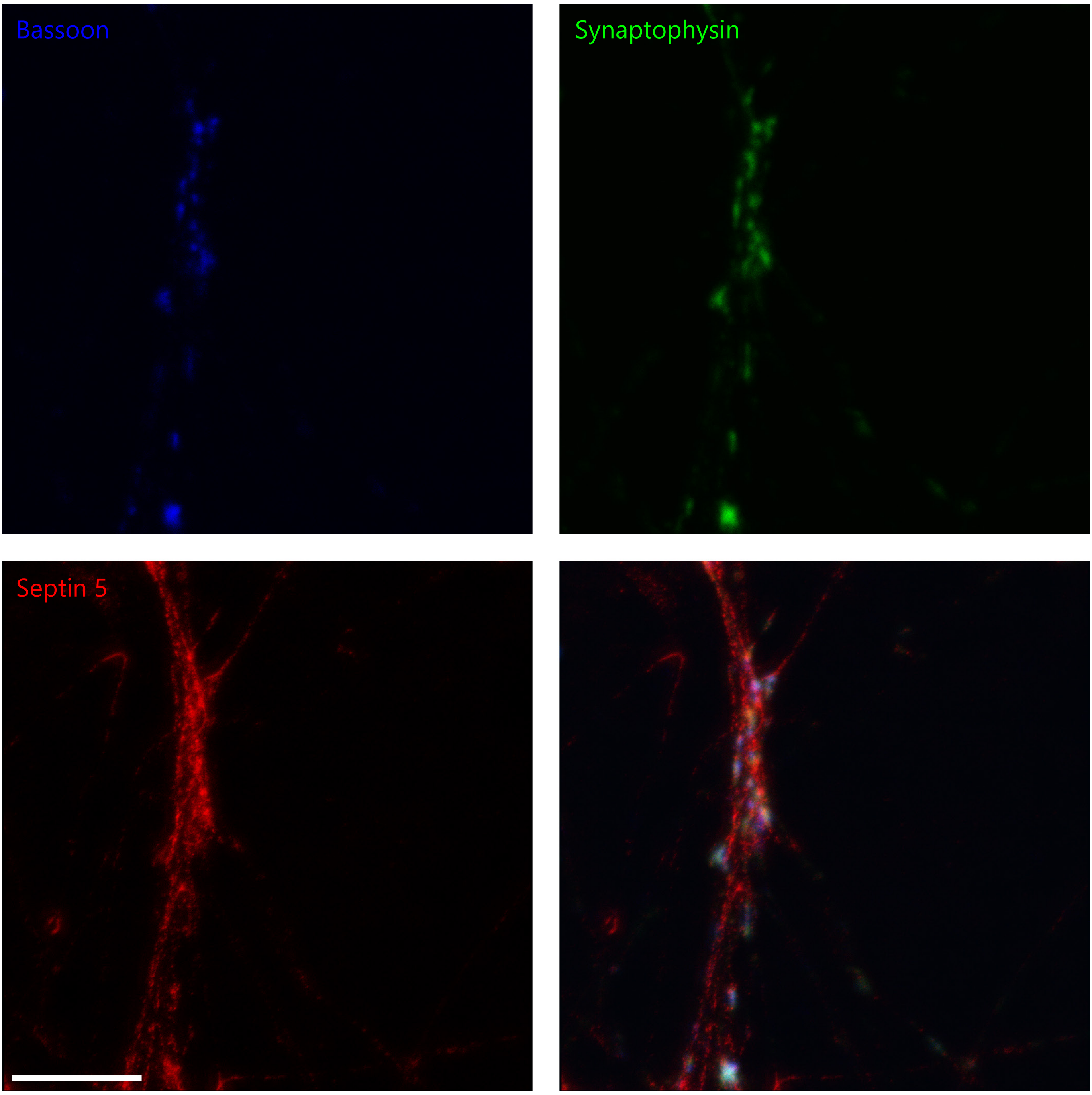

Scale bar: 5 µm

Antibodies used:
Bassoon - Synaptic Systems (Göttingen, Germany), 141 021
Synaptophysin - Synaptic Systems (Göttingen, Germany), 101 011
Septin 5 - Volker Haucke (FMP, Berlin, Germany)

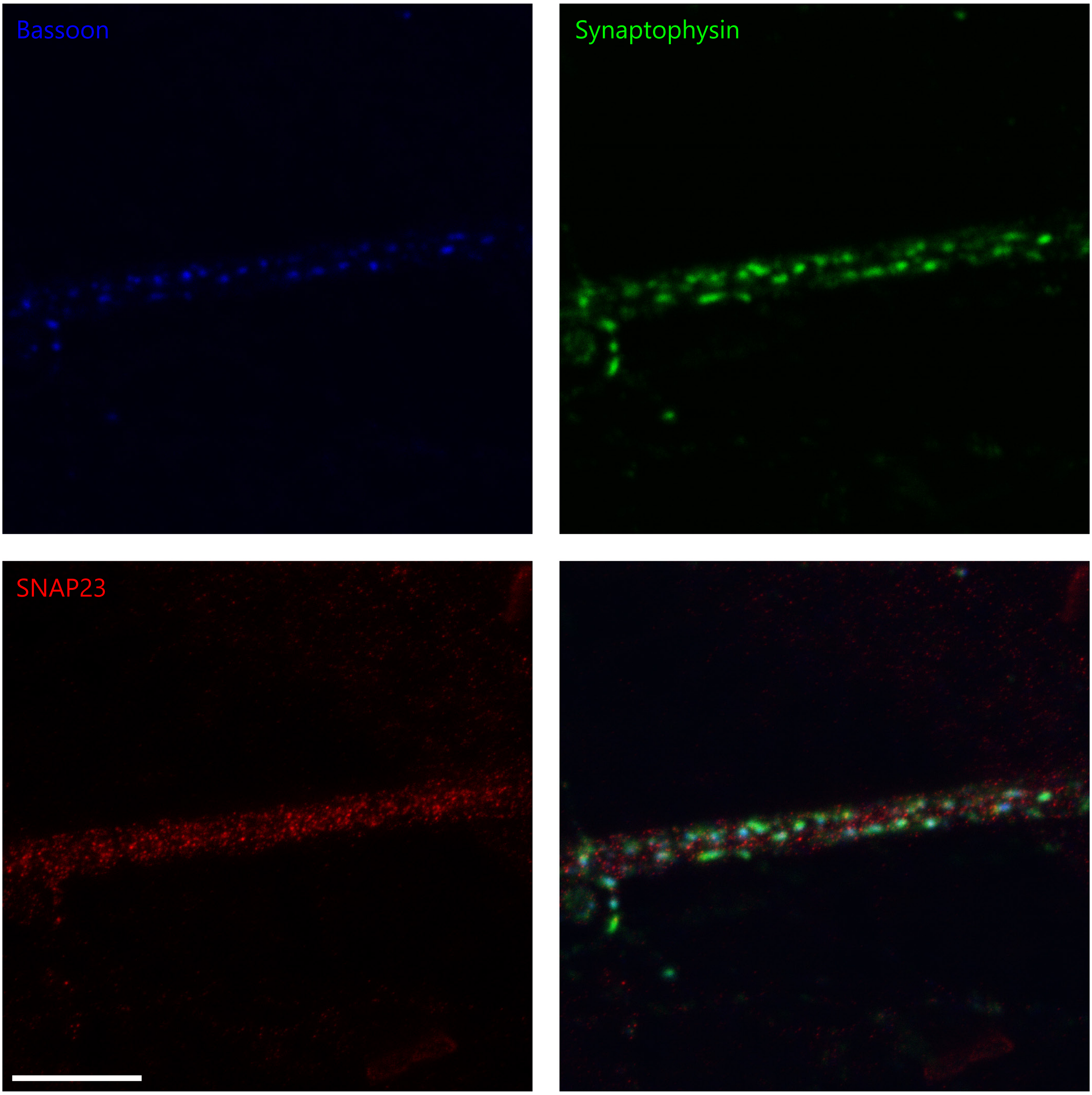

Scale bar: 5 µm

Antibodies used:
Bassoon - Synaptic Systems (Göttingen, Germany), 141 021
Synaptophysin - Synaptic Systems (Göttingen, Germany), 101 011
SNAP23 - Synaptic Systems (Göttingen, Germany), 111 202

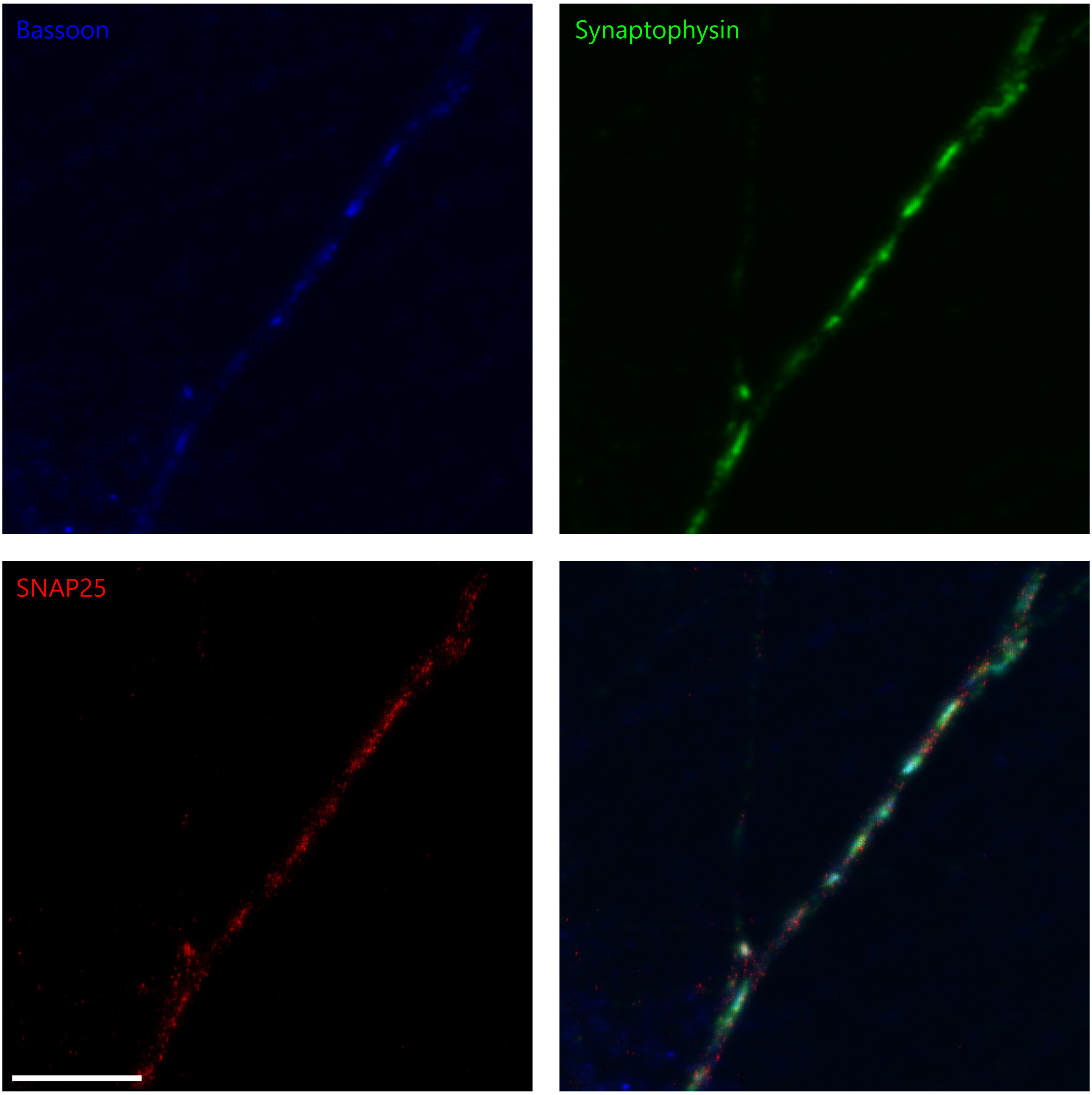

Scale bar: 5 µm

Antibodies used:
Bassoon - Synaptic Systems (Göttingen, Germany), 141 021
Synaptophysin - Synaptic Systems (Göttingen, Germany), 101 011
SNAP25 - Synaptic Systems (Göttingen, Germany), 111 011

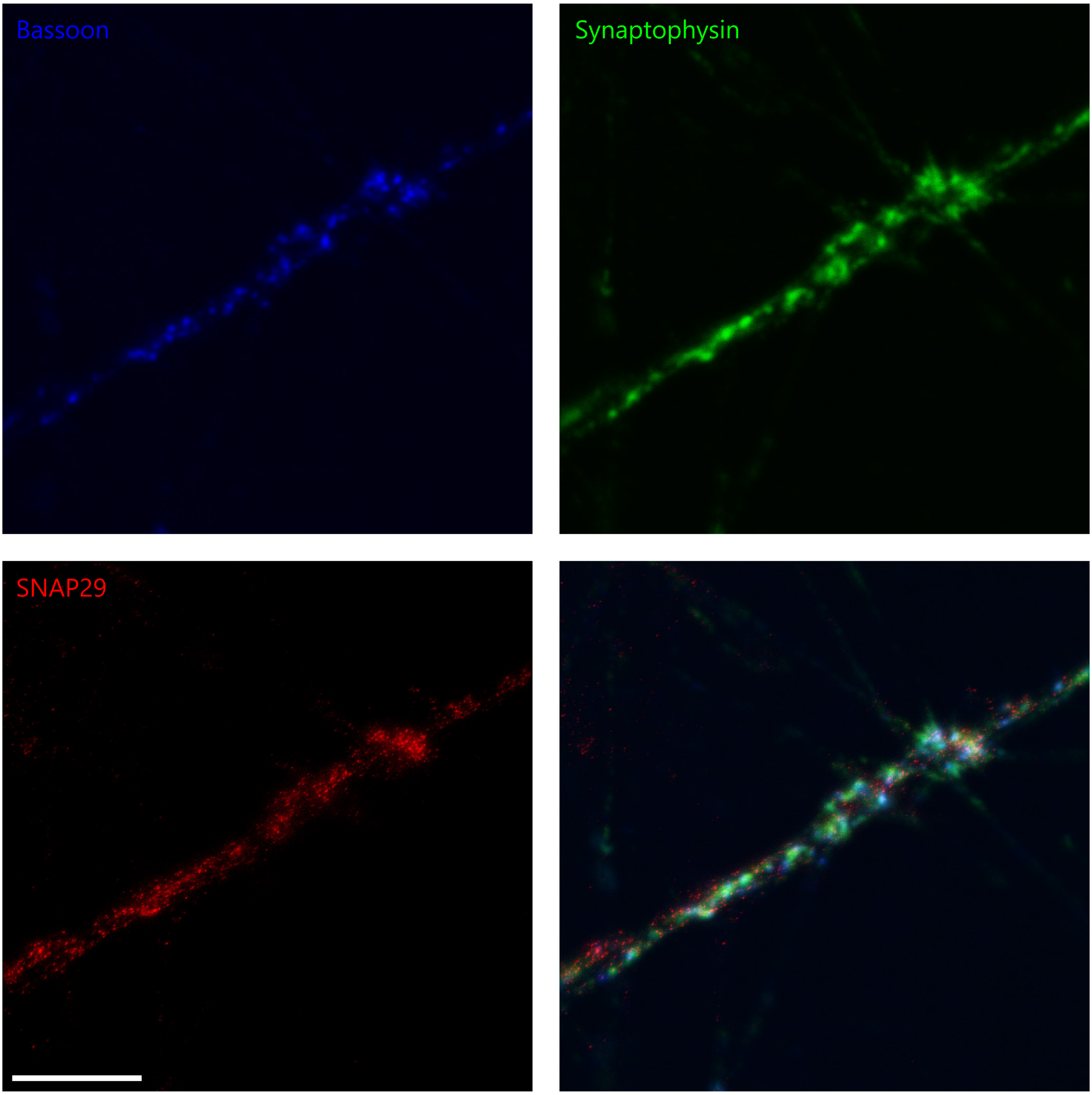

Scale bar: 5 µm

Antibodies used:
Bassoon - Synaptic Systems (Göttingen, Germany), 141 021
Synaptophysin - Synaptic Systems (Göttingen, Germany), 101 011
SNAP29 - Abcam (Cambridge, England), ab68824

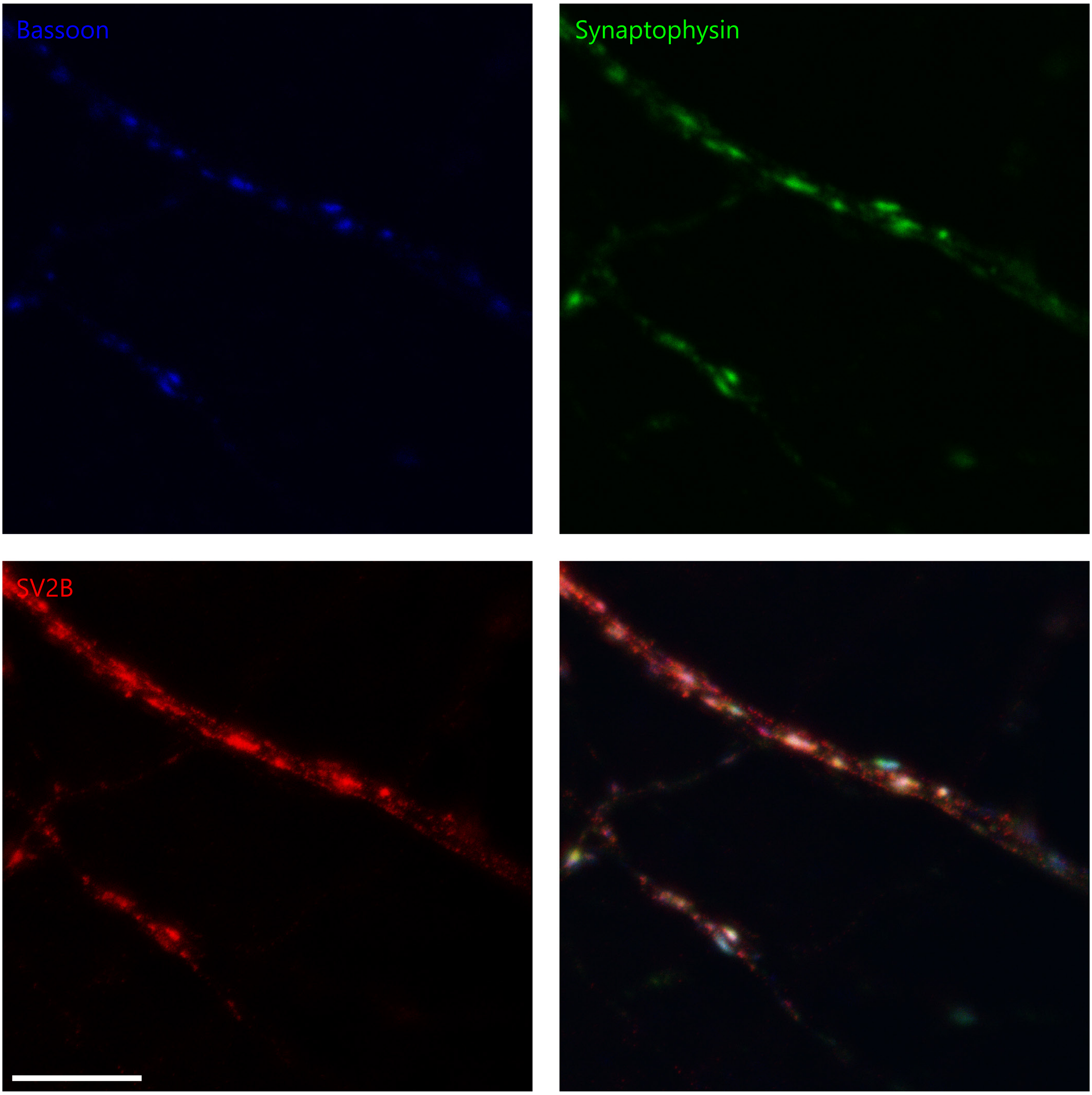

Scale bar: 5 µm

Antibodies used:
Bassoon - Synaptic Systems (Göttingen, Germany), 141 021
Synaptophysin - Synaptic Systems (Göttingen, Germany), 101 011
SV2B - Reinhard Jahn (MPI-BPC, Göttingen, Germany)

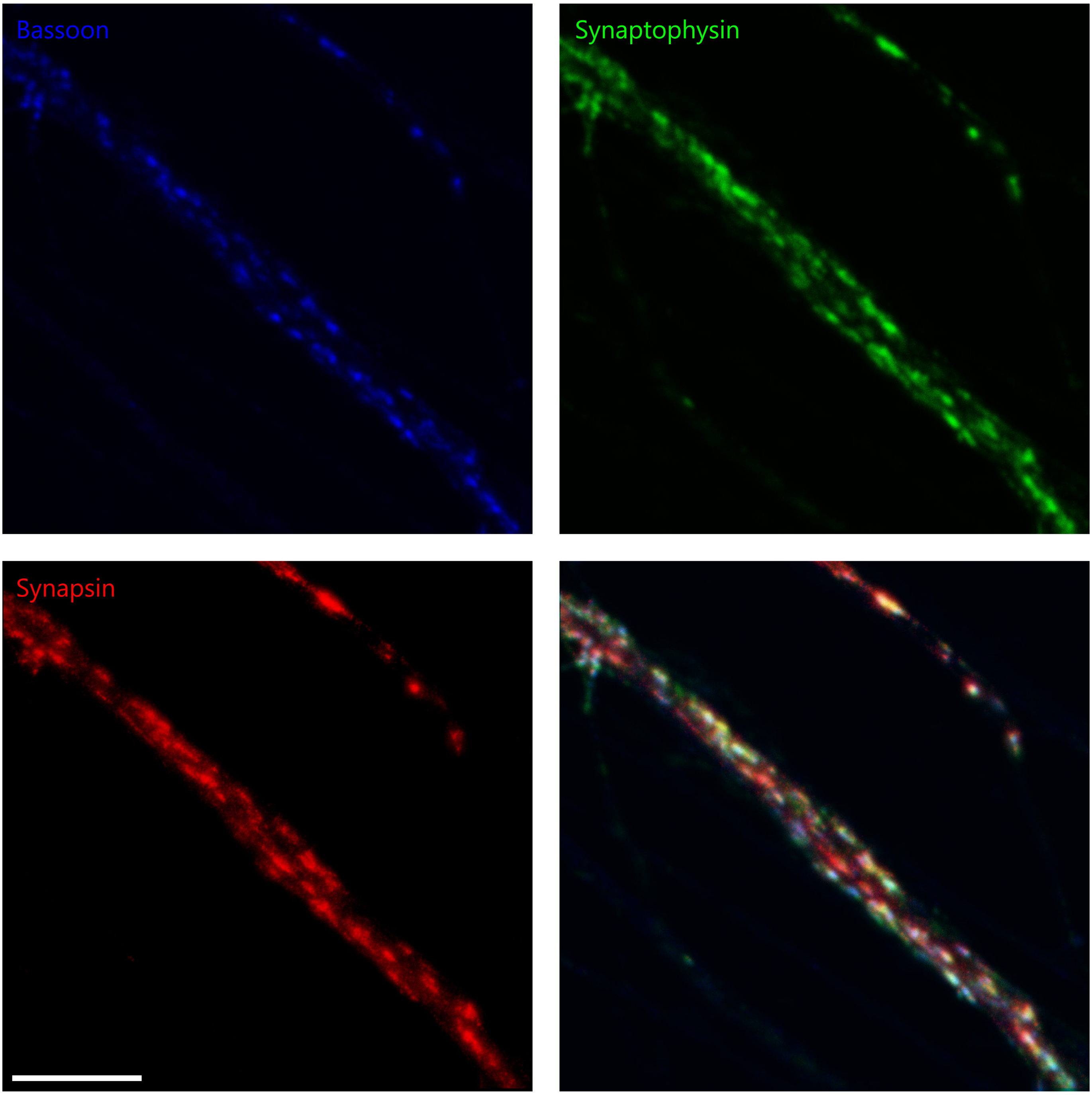

Scale bar: 5 µm

Antibodies used:
Bassoon - Synaptic Systems (Göttingen, Germany), 141 021
Synaptophysin - Synaptic Systems (Göttingen, Germany), 101 011
Synapsin - Reinhard Jahn (MPI-BPC, Göttingen, Germany)

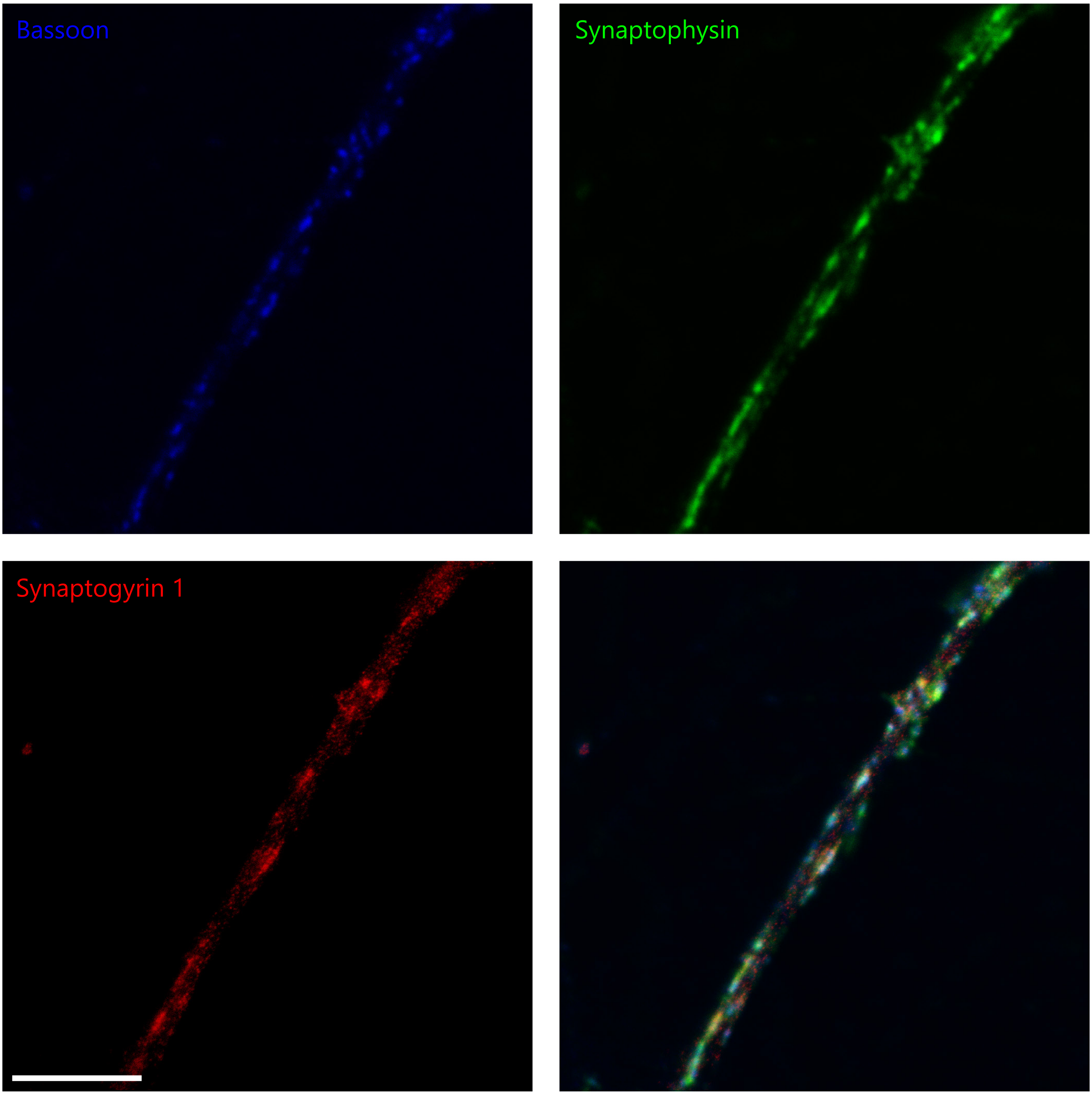

Scale bar: 5 µm

Antibodies used:
Bassoon - Synaptic Systems (Göttingen, Germany), 141 021
Synaptophysin - Synaptic Systems (Göttingen, Germany), 101 011
Synaptogyrin 1 - Synaptic Systems (Göttingen, Germany), 103 002

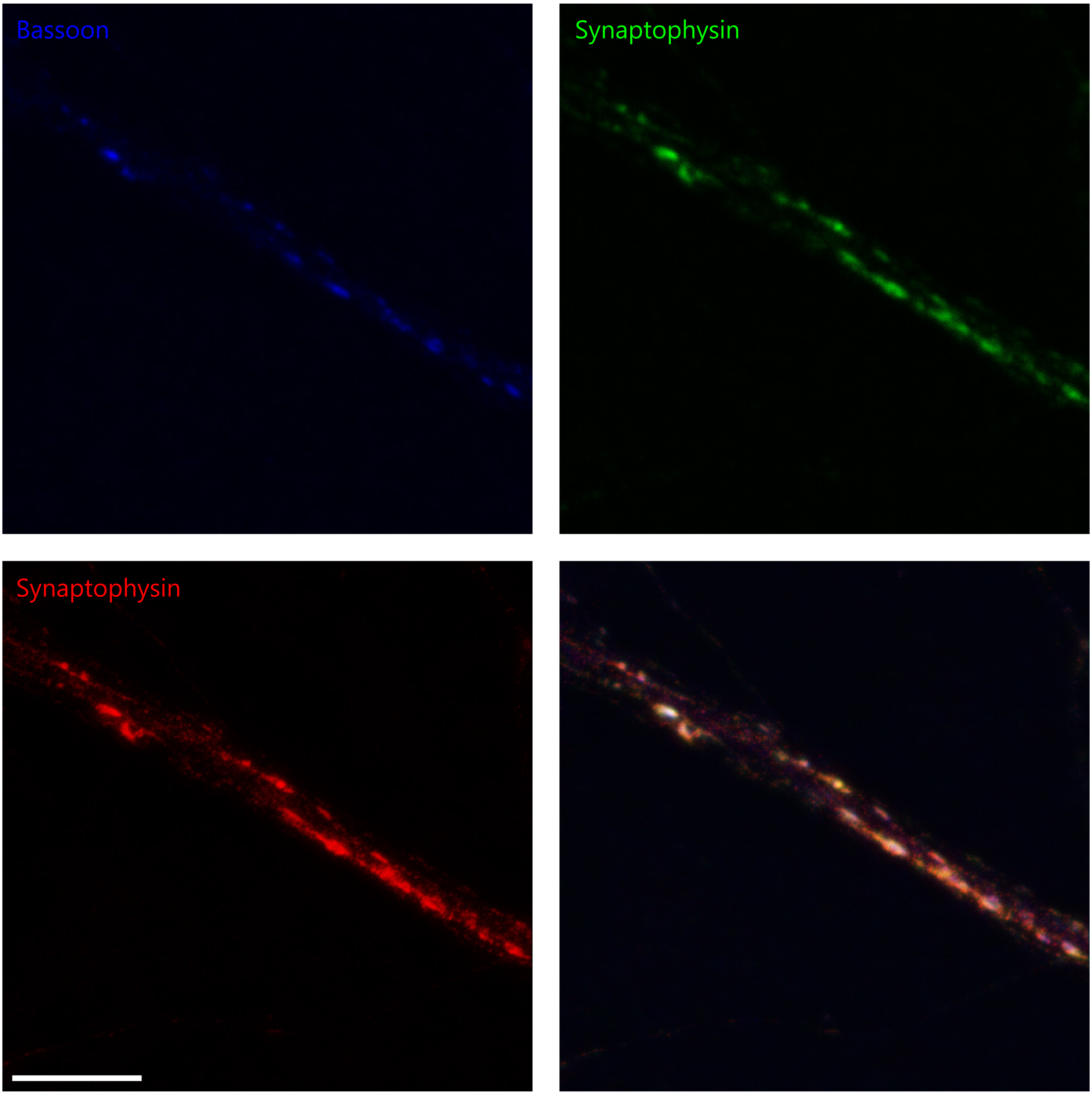

Scale bar: 5 µm

Antibodies used:
Bassoon - Synaptic Systems (Göttingen, Germany), 141 021
Synaptophysin - Synaptic Systems (Göttingen, Germany), 101 011
Synaptophysin - Synaptic Systems (Göttingen, Germany), 101 011

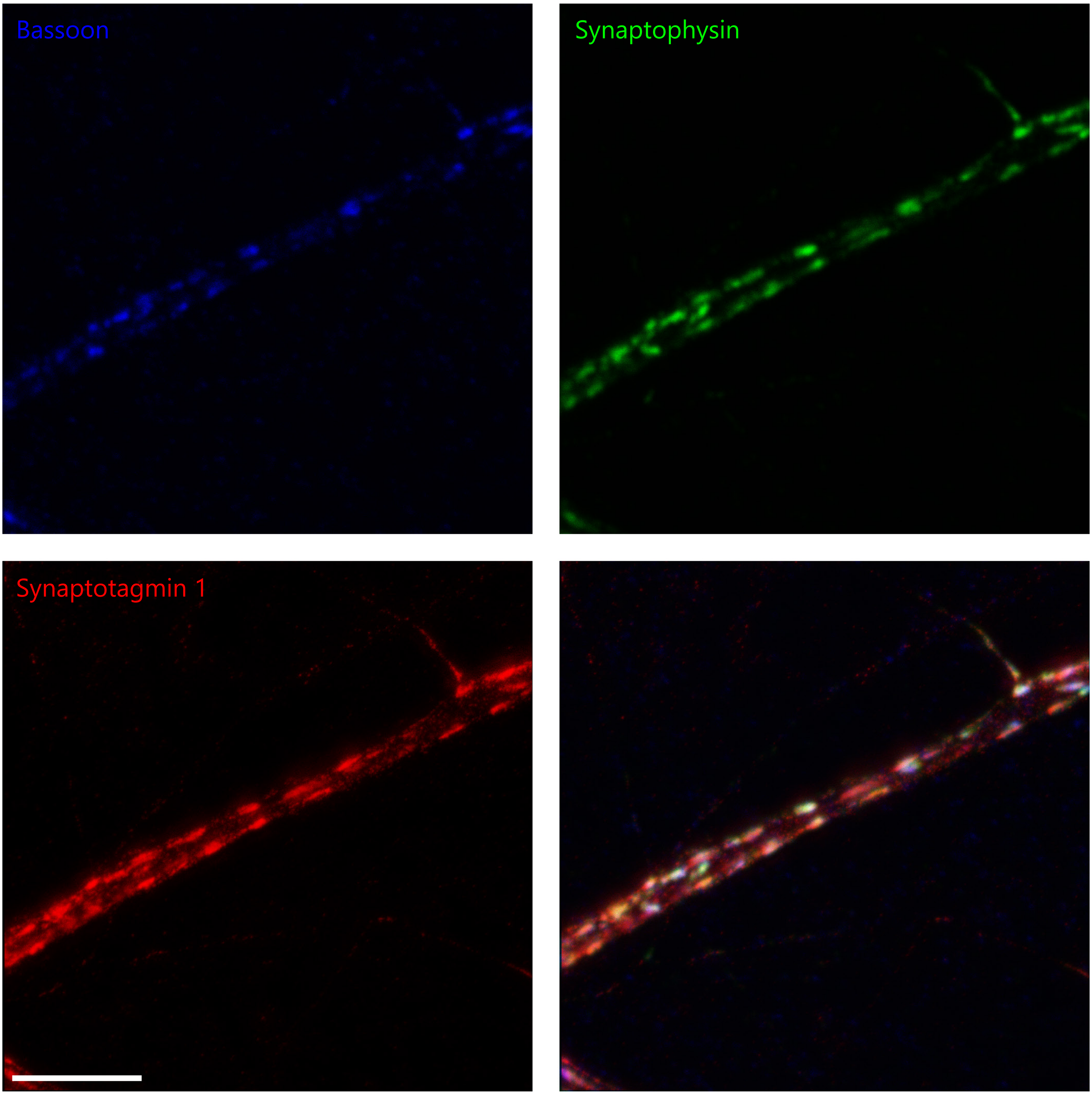

Scale bar: 5 µm

Antibodies used:
Bassoon - Synaptic Systems (Göttingen, Germany), 141 021
Synaptophysin - Synaptic Systems (Göttingen, Germany), 101 011
Synaptotagmin 1 - Synaptic Systems (Göttingen, Germany), 105 311AT1

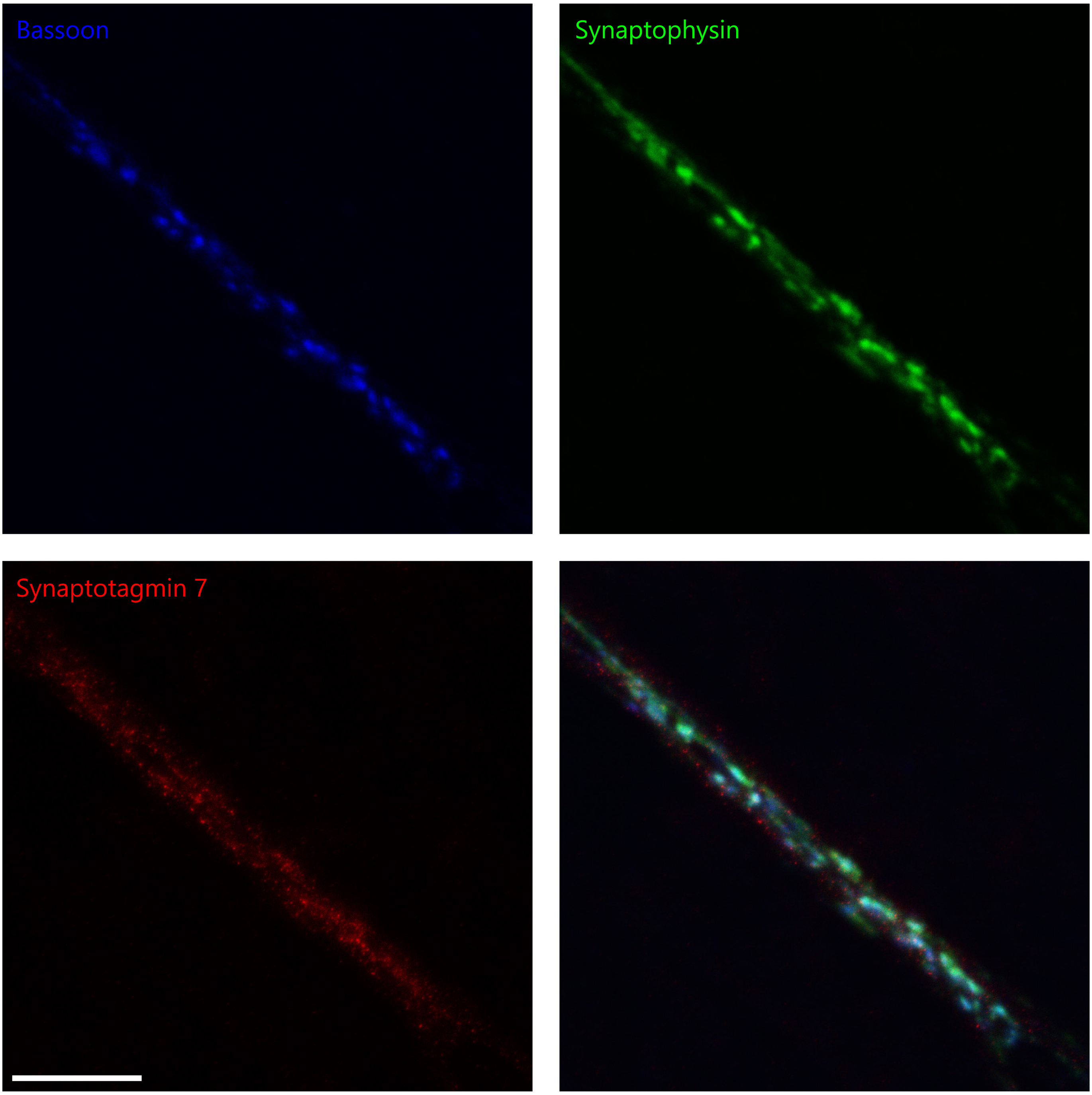

Scale bar: 5 µm

Antibodies used:
Bassoon - Synaptic Systems (Göttingen, Germany), 141 021
Synaptophysin - Synaptic Systems (Göttingen, Germany), 101 011
Synaptotagmin 7 - Synaptic Systems (Göttingen, Germany), 105 173

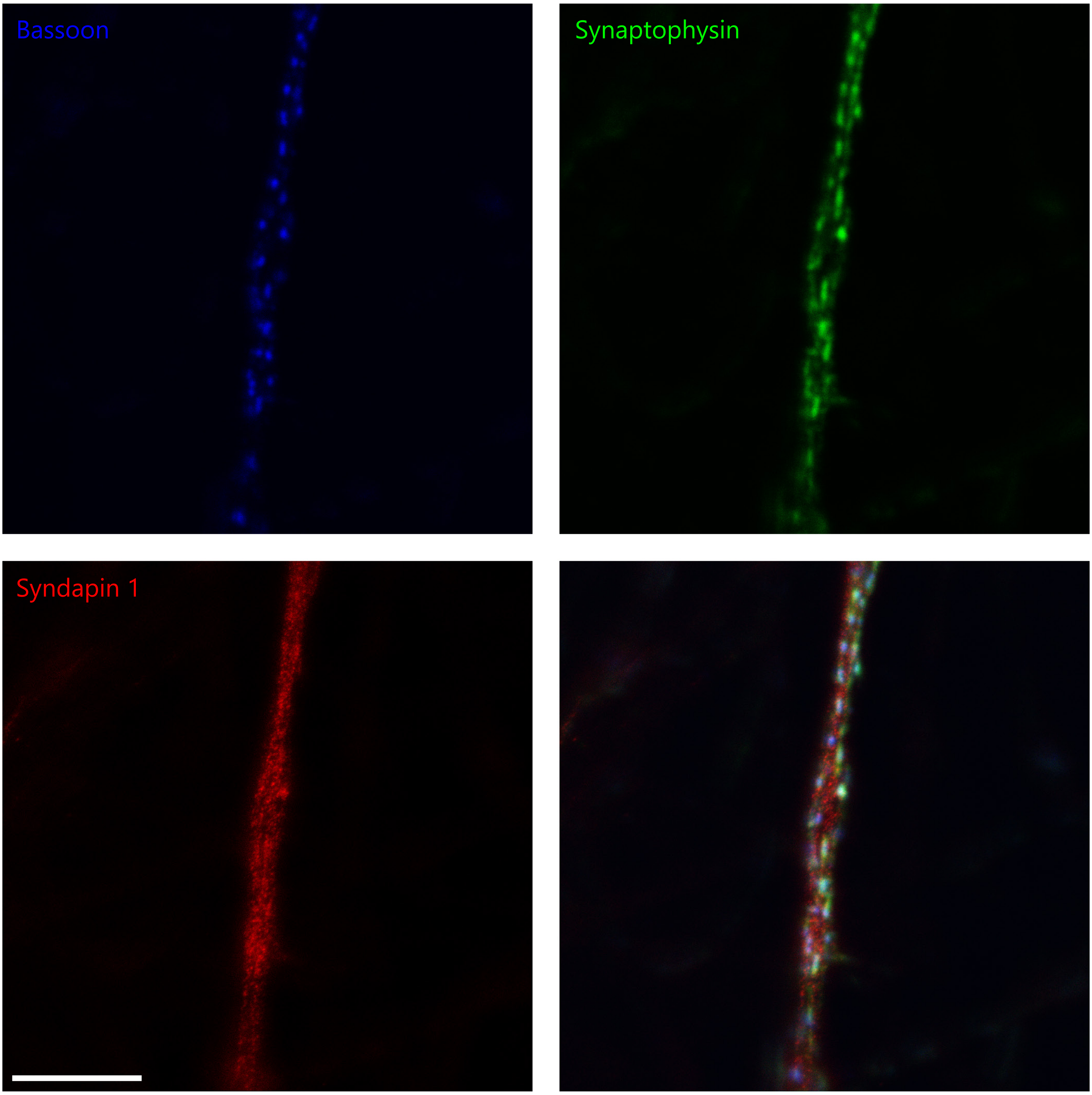

Scale bar: 5 µm

Antibodies used:
Bassoon - Synaptic Systems (Göttingen, Germany), 141 021
Synaptophysin - Synaptic Systems (Göttingen, Germany), 101 011
Syndapin 1 - Novus Biologicals (Littleton, Colorado, USA), H00029993-B01

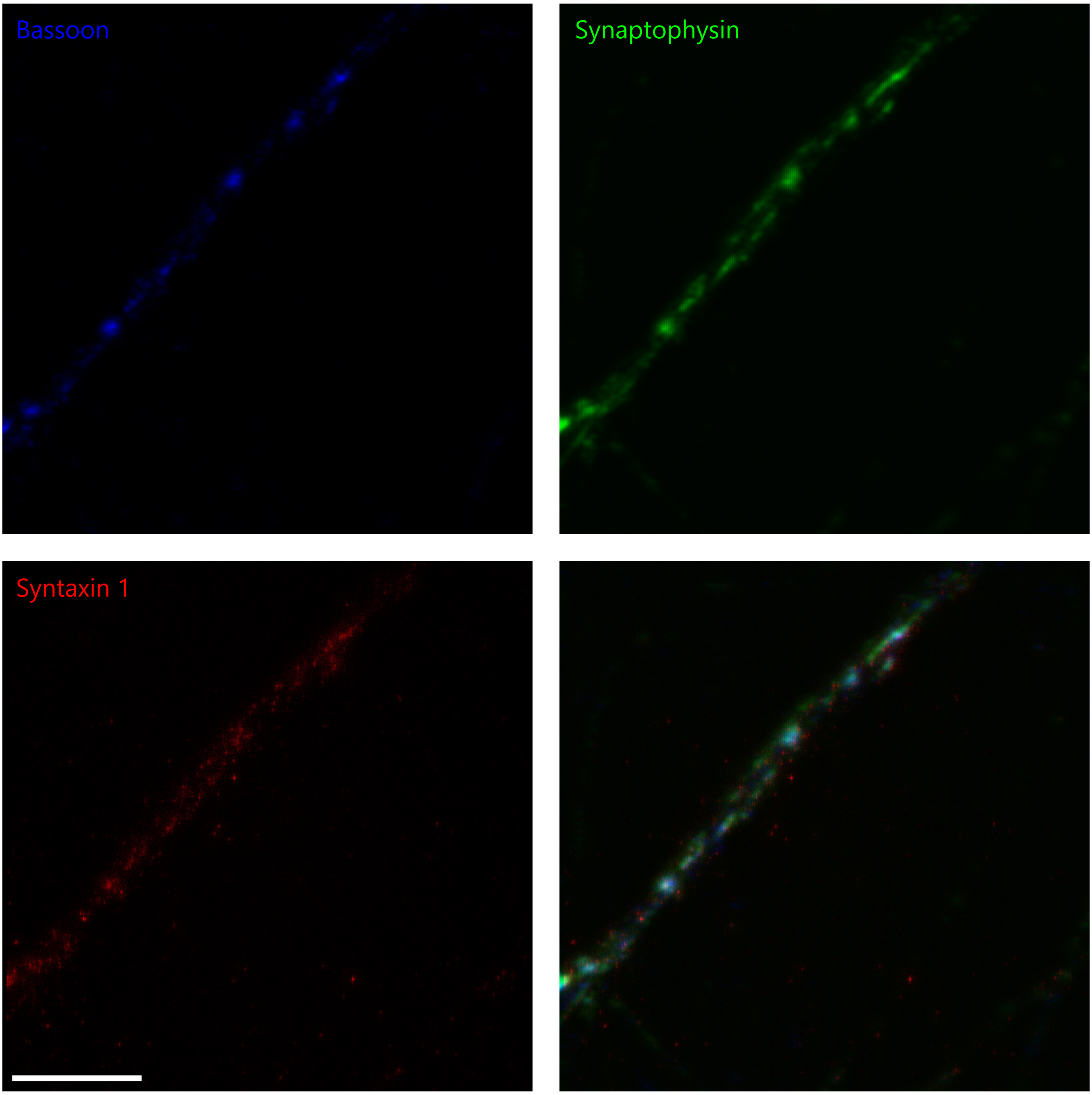

Scale bar: 5 µm

Antibodies used:
Bassoon - Synaptic Systems (Göttingen, Germany), 141 021
Synaptophysin - Synaptic Systems (Göttingen, Germany), 101 011
Syntaxin 1 - Reinhard Jahn (MPI-BPC, Göttingen, Germany)

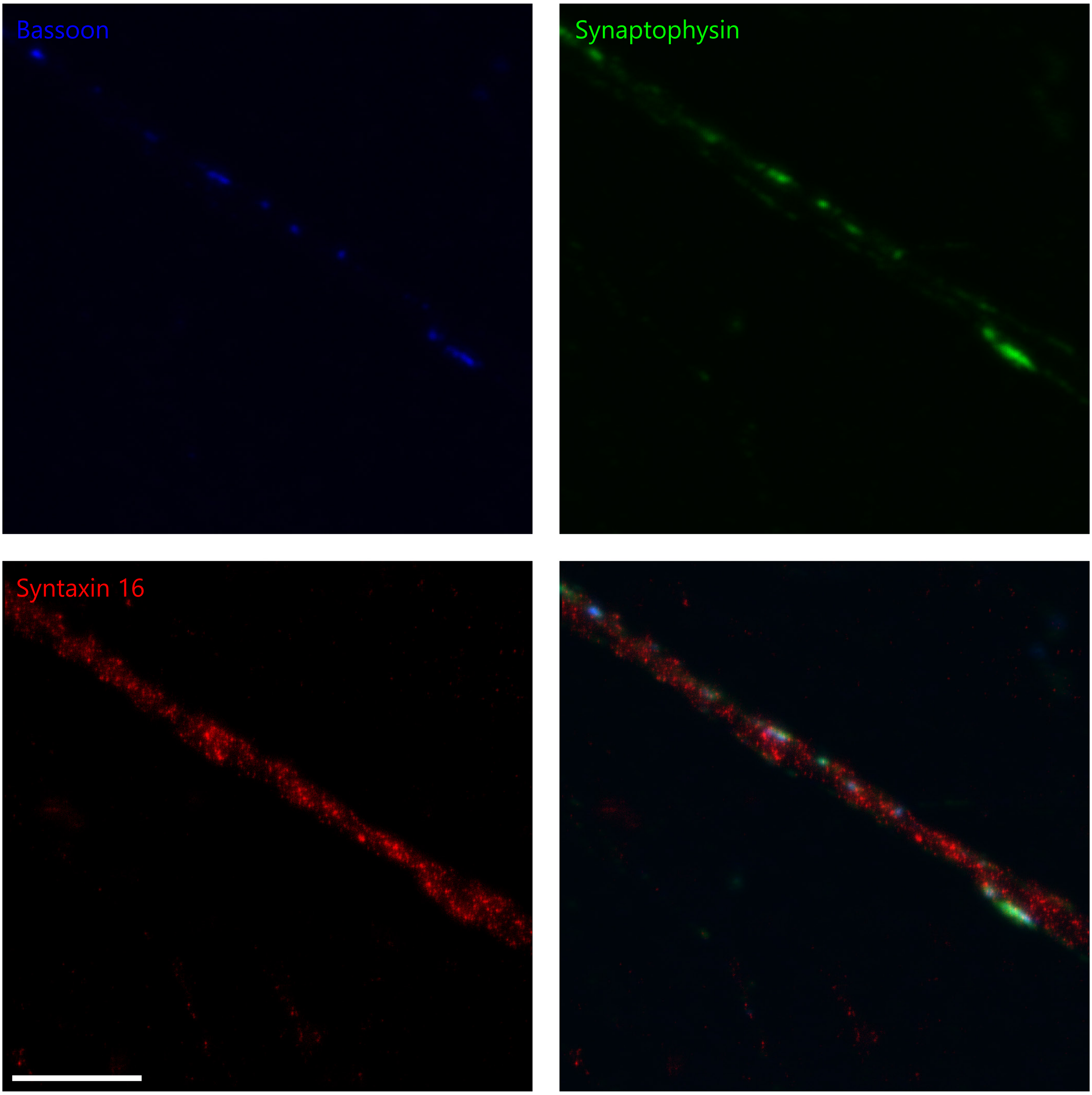

Scale bar: 5 µm

Antibodies used:
Bassoon - Synaptic Systems (Göttingen, Germany), 141 021
Synaptophysin - Synaptic Systems (Göttingen, Germany), 101 011
Syntaxin 16 - Synaptic Systems (Göttingen, Germany), 110 162

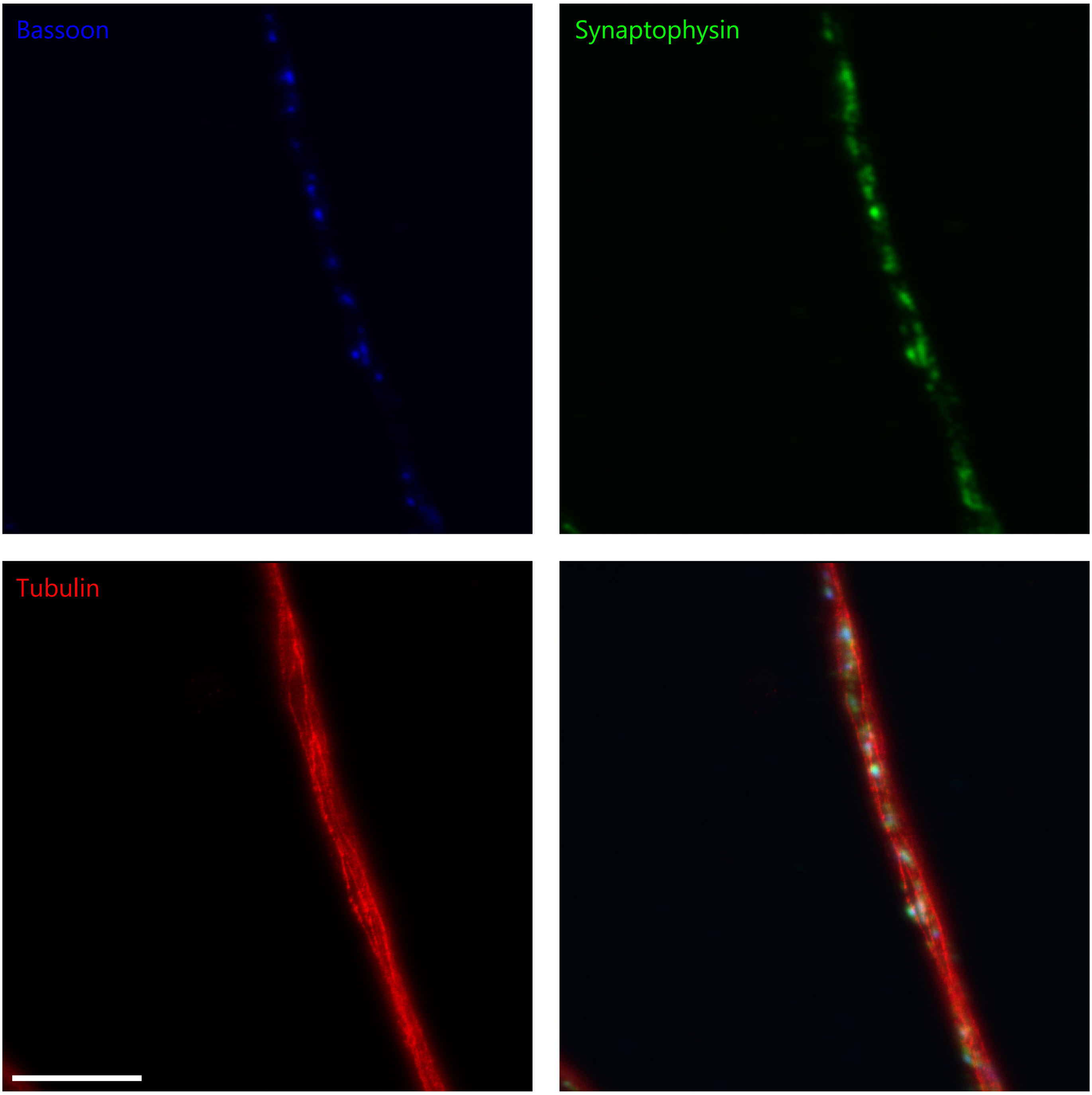

Scale bar: 5 µm

Antibodies used:
Bassoon - Synaptic Systems (Göttingen, Germany), 141 021
Synaptophysin - Synaptic Systems (Göttingen, Germany), 101 011
Tubulin - Synaptic Systems (Göttingen, Germany), 302 203

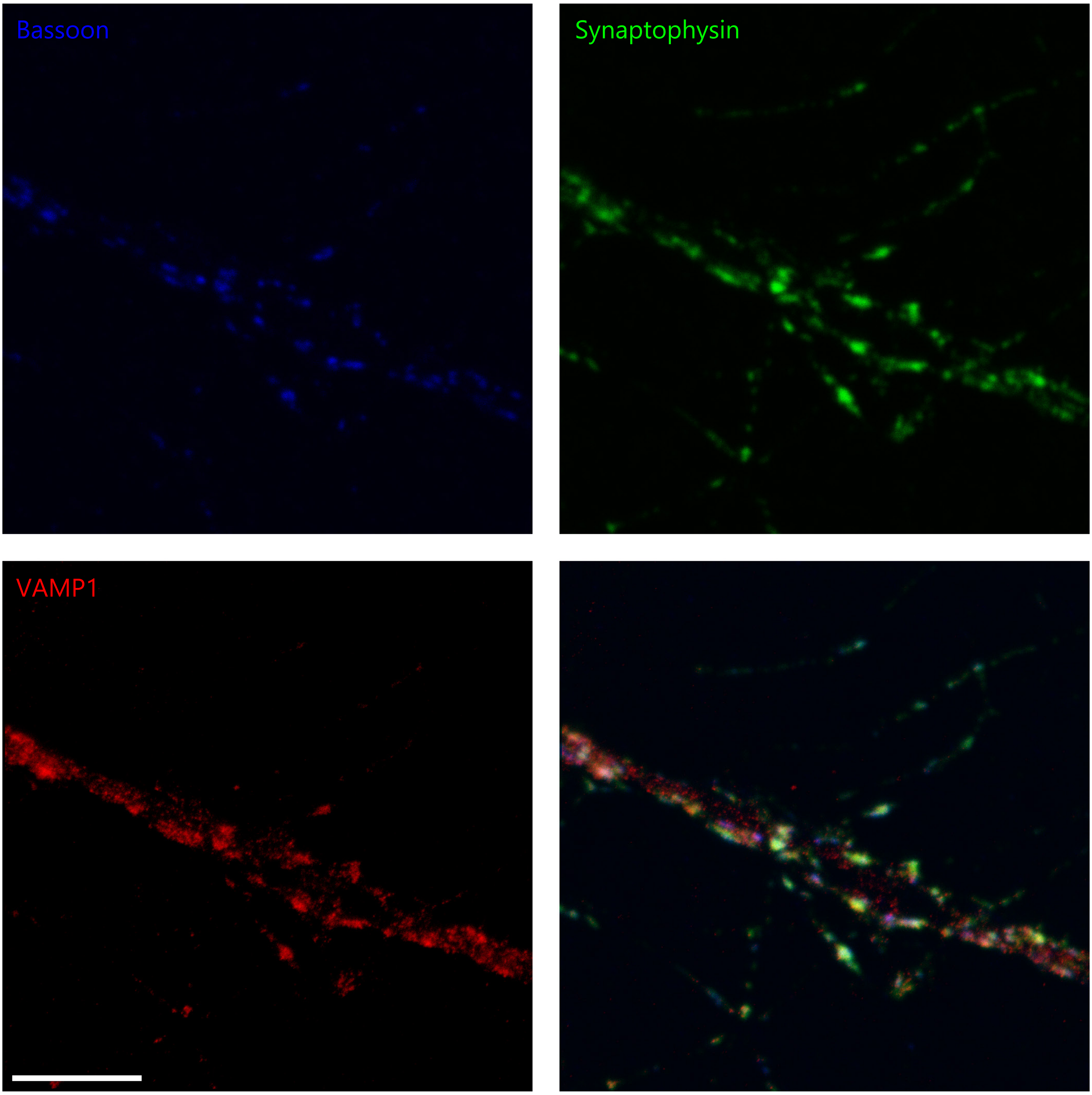

Scale bar: 5 µm

Antibodies used:
Bassoon - Synaptic Systems (Göttingen, Germany), 141 021
Synaptophysin - Synaptic Systems (Göttingen, Germany), 101 011
VAMP1 - Synaptic Systems (Göttingen, Germany), 104 002

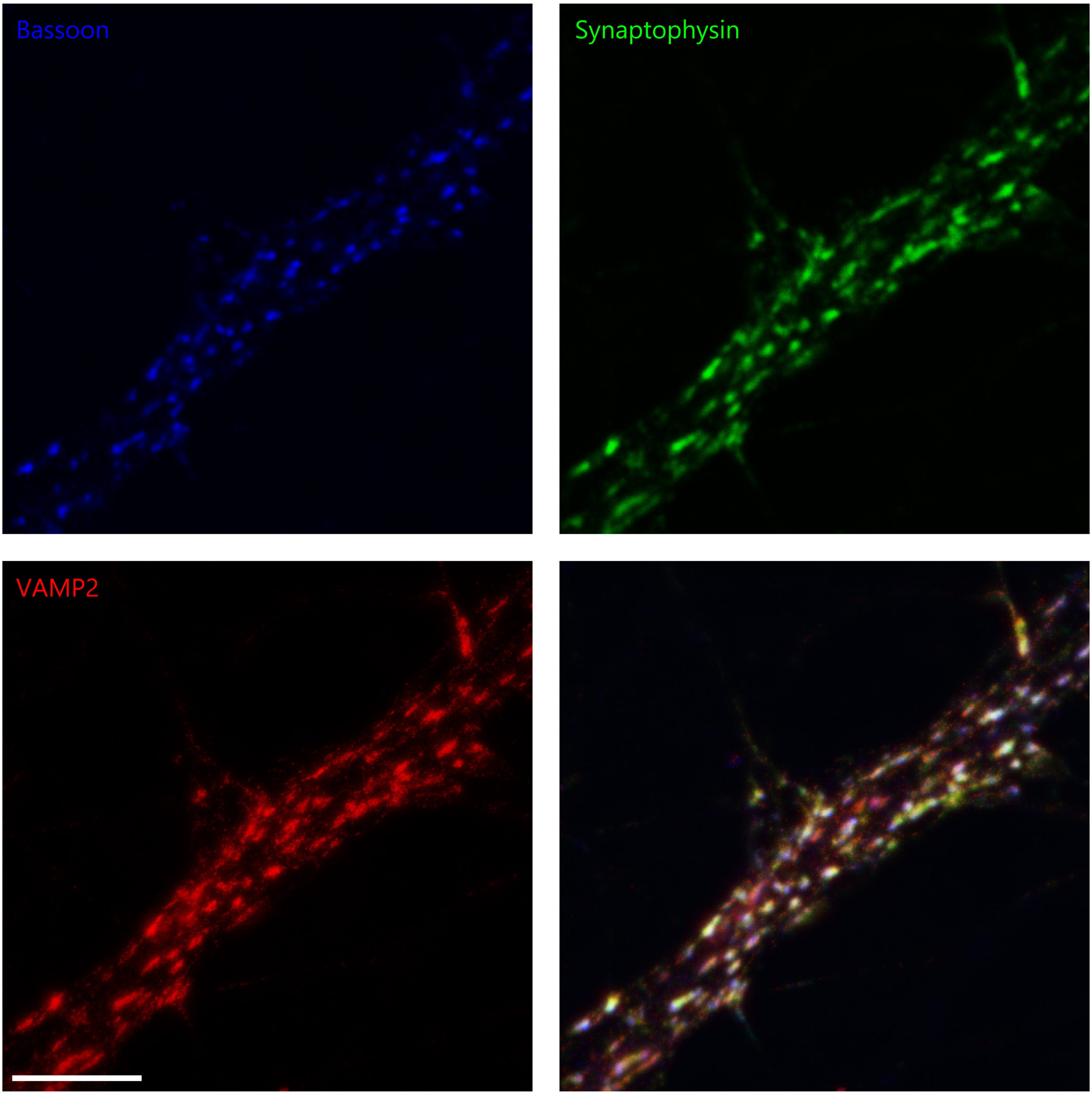

Scale bar: 5 µm

Antibodies used:
Bassoon - Synaptic Systems (Göttingen, Germany), 141 021
Synaptophysin - Synaptic Systems (Göttingen, Germany), 101 011
VAMP2 - Synaptic Systems (Göttingen, Germany), 104 211

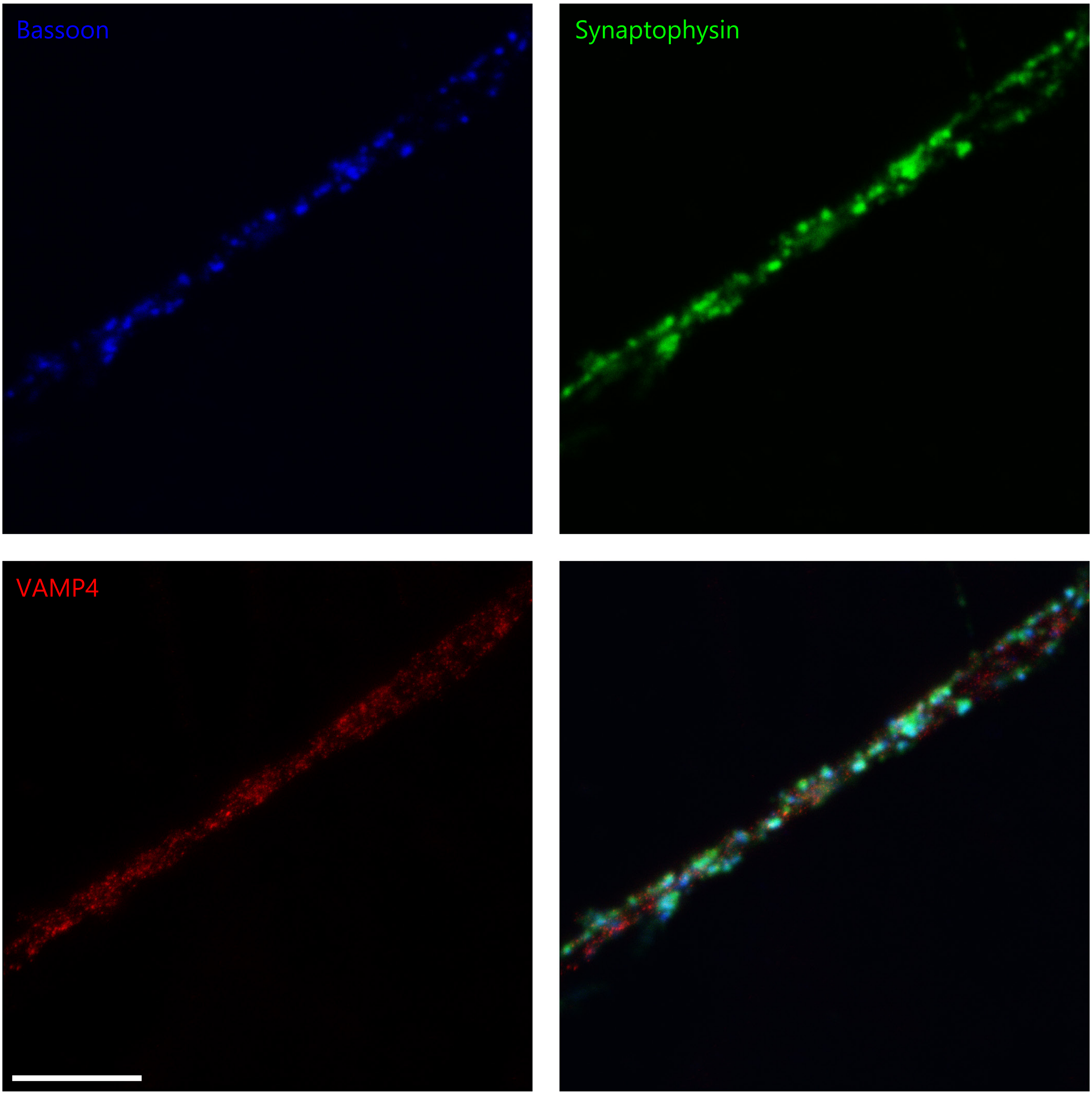

Scale bar: 5 µm

Antibodies used:
Bassoon - Synaptic Systems (Göttingen, Germany), 141 021
Synaptophysin - Synaptic Systems (Göttingen, Germany), 101 011
VAMP4 - Synaptic Systems (Göttingen, Germany), 136 002

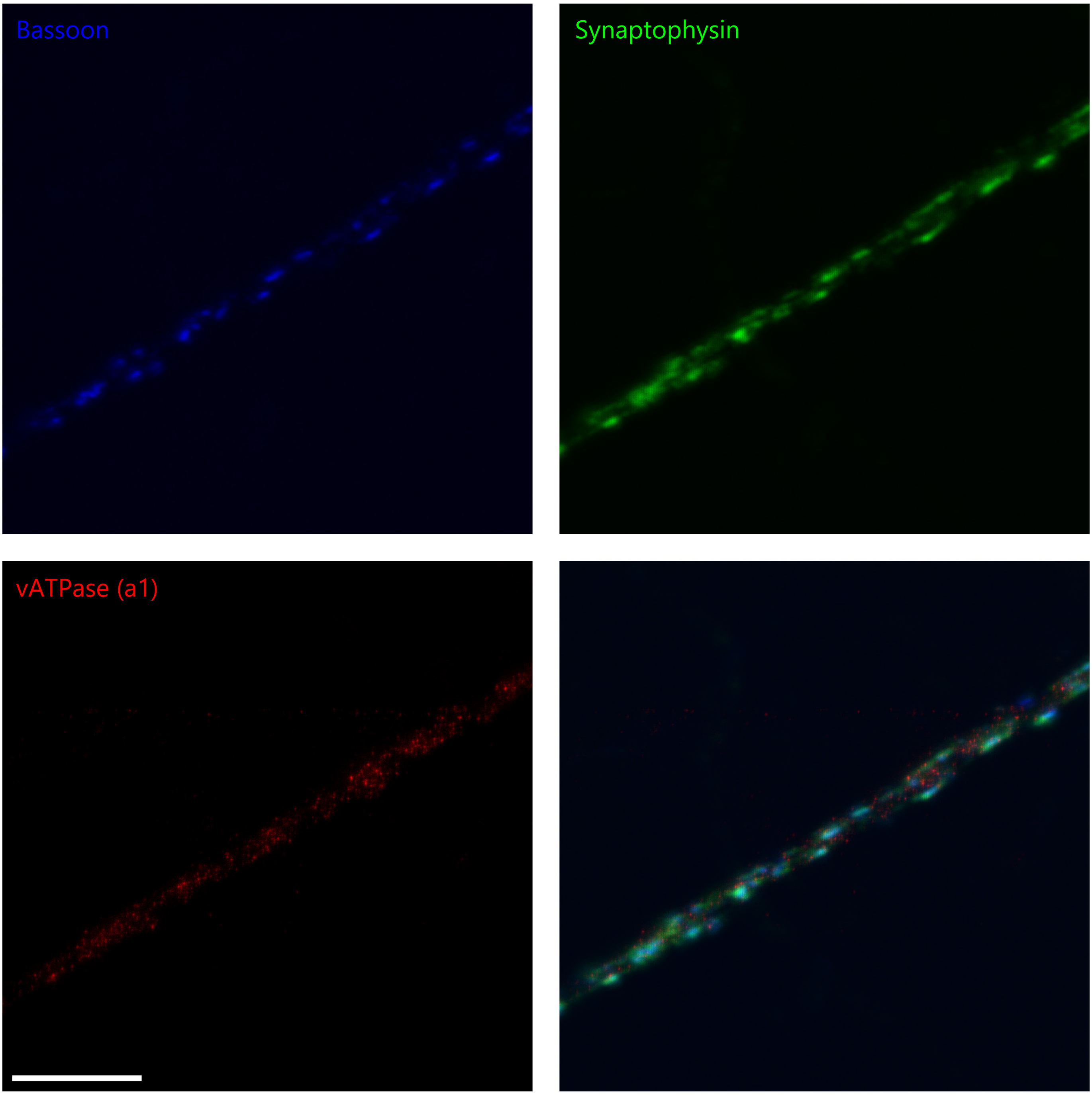

Scale bar: 5 µm

Antibodies used:
Bassoon - Synaptic Systems (Göttingen, Germany), 141 021
Synaptophysin - Synaptic Systems (Göttingen, Germany), 101 011
vATPase (a1) - Synaptic Systems (Göttingen, Germany), 109 002

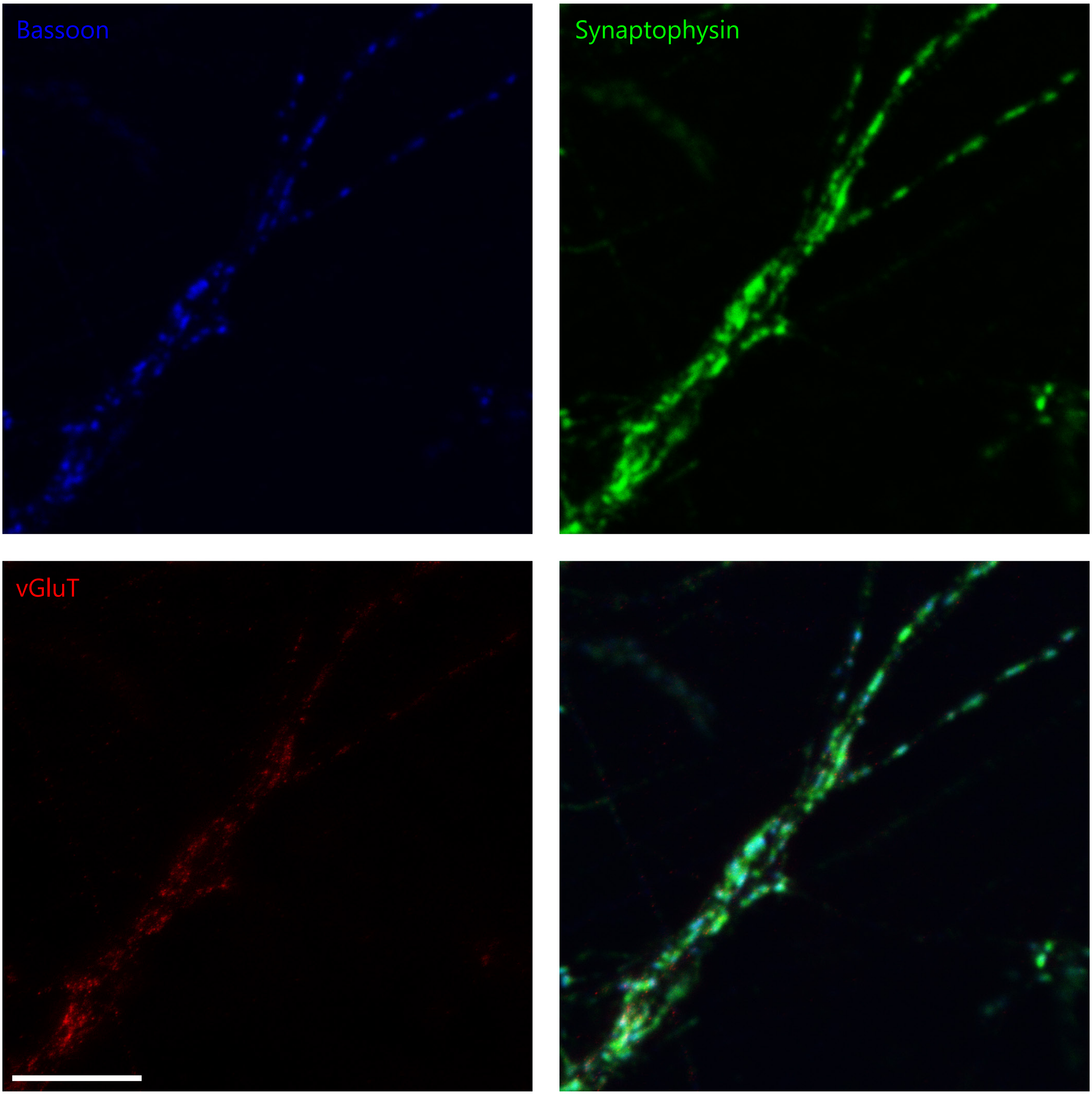

Scale bar: 5 µm

Antibodies used:
Bassoon - Synaptic Systems (Göttingen, Germany), 141 021
Synaptophysin - Synaptic Systems (Göttingen, Germany), 101 011
vGluT - Synaptic Systems (Göttingen, Germany), 135 503

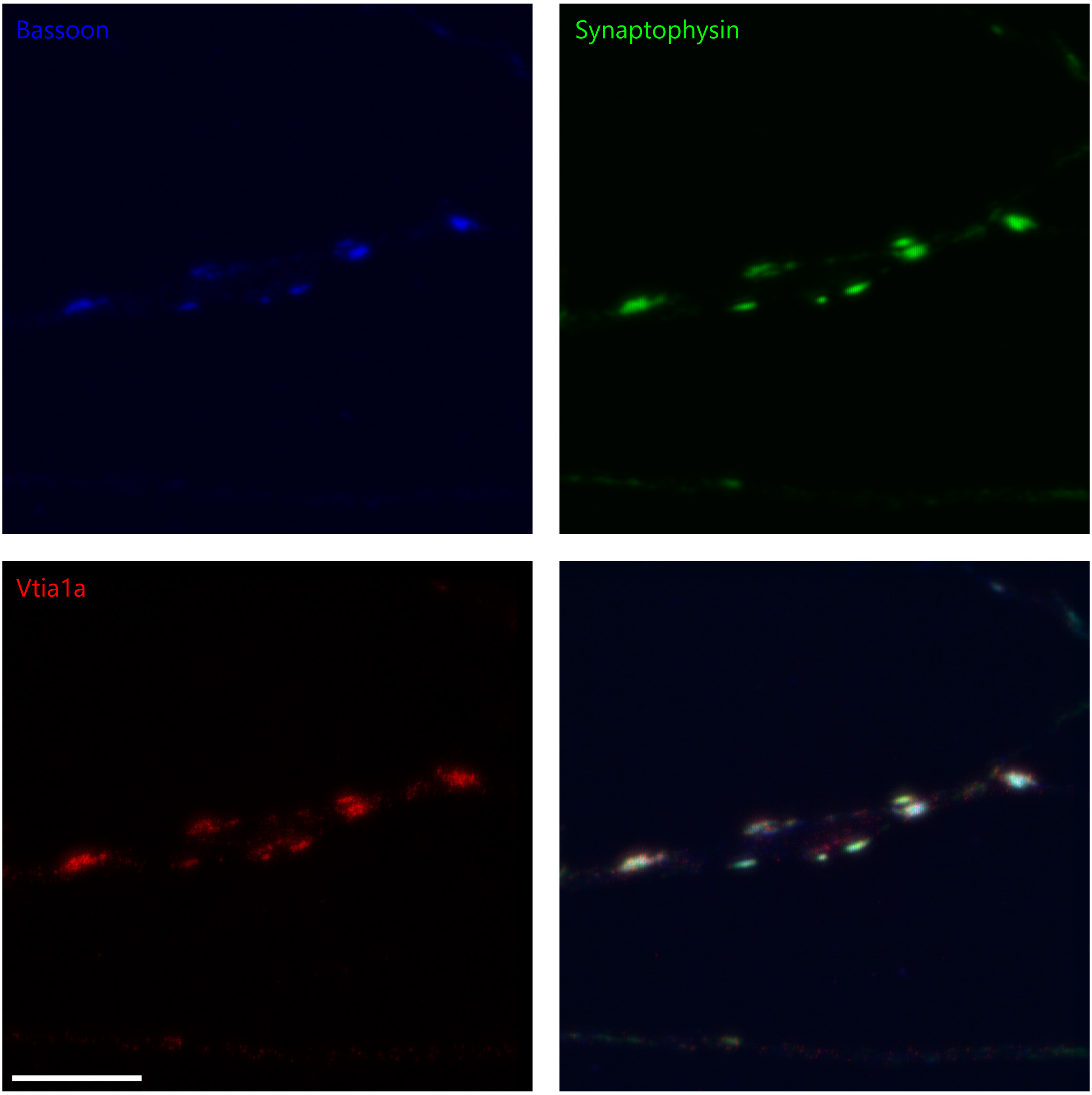

Scale bar: 5 µm

Antibodies used:
Bassoon - Synaptic Systems (Göttingen, Germany), 141 021
Synaptophysin - Synaptic Systems (Göttingen, Germany), 101 011
Vtia1a - BD Biosciences (Heidelberg, Germany), 611220

Multi-page figure

Appendix Fig. S3. Measured FRAP parameters for each individual protein. Please follow the graphical legend presented on the first page of this figure. The box plots are organized as follows: the middle line shows the median; the box edges indicate the 25th percentile; the error bars show the 75th percentile; the symbols show the 90th percentile.

protein name

MW (kDa)	Category	τ_{axon} (s)	$\tau_{synapse}$ (s)	IF_{axon} (%)	$IF_{synapse}$ (%)
molecular weight	category according to localization and function	mean time constant in axon ± SEM	mean time constant in synapse ± SEM	mean immobile fraction in axon ± SEM	mean immobile fraction in synapse ± SEM

-1.7 s	0.5 s	1 s	3 s	10 s	20 s	40 s	82 s

Axon Examplary frames from a typical FRAP experiment in axonal regions. First post-bleach frame is labeled 0.5 s. 8 frames of a total of 76 frames taken in each experiment are shown. Scale bar, 500 nm. The panel is empty if the protein cannot be found in axons.

Synapse Examplary frames from a typical FRAP experiment in synapses. First post-bleach frame is labeled 0.5 s. 8 frames of a total of 76 frames taken in each experiment are shown. Scale bar, 500 nm.

Average FRAP curves recorded in axons (blue) and synapses (green). Shaded area represents SEM.

Box plot of time constants in axons and synapses. Asterisks denote significant differences.

Box plot of immobile fractions in axons and synapses. Asterisks denote significant differences.

Number of FRAP experiments done in axonal regions and synapses. P-values are shown when the difference is significant for FDR = 0.05, using the Benjamini-Hochberg procedure for multiple testing correction.

Tagged protein outline:

Schematic representation of the expressed protein. The relative sizes of the fluorescent tag, linker region and the protein of interest are preserved. The sequence of linker region is shown in one-letter amino acid code. The reference number of the mRNA sequence is shown below the protein of interest.

List of proteins with significantly different values for the time constants or immobile fractions in axons or synapses, in comparison to the protein of interest. Kruskal-Wallis tests were followed by Wilcoxon rank-sum tests, with a Bonferroni multiple test correction; corrected p-values are shown.

References

β-Actin

MW (kDa)	Category	τ_{axon} (s)	$\tau_{synapse}$ (s)	IF_{axon} (%)	$IF_{synapse}$ (%)
41.7	cytoskeletal	1.37 ± 0.11	6.35 ± 0.79	28.40 ± 4.59	13.19 ± 8.88

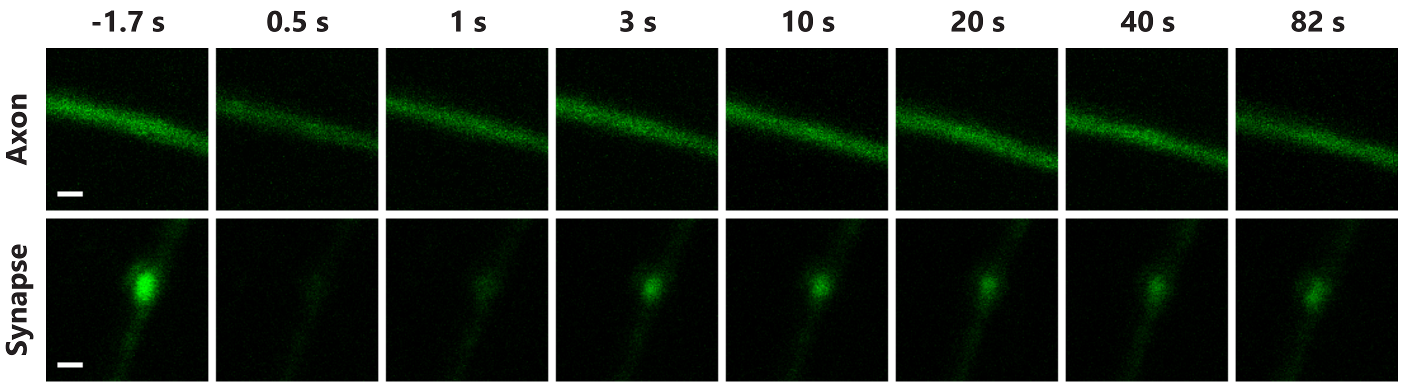

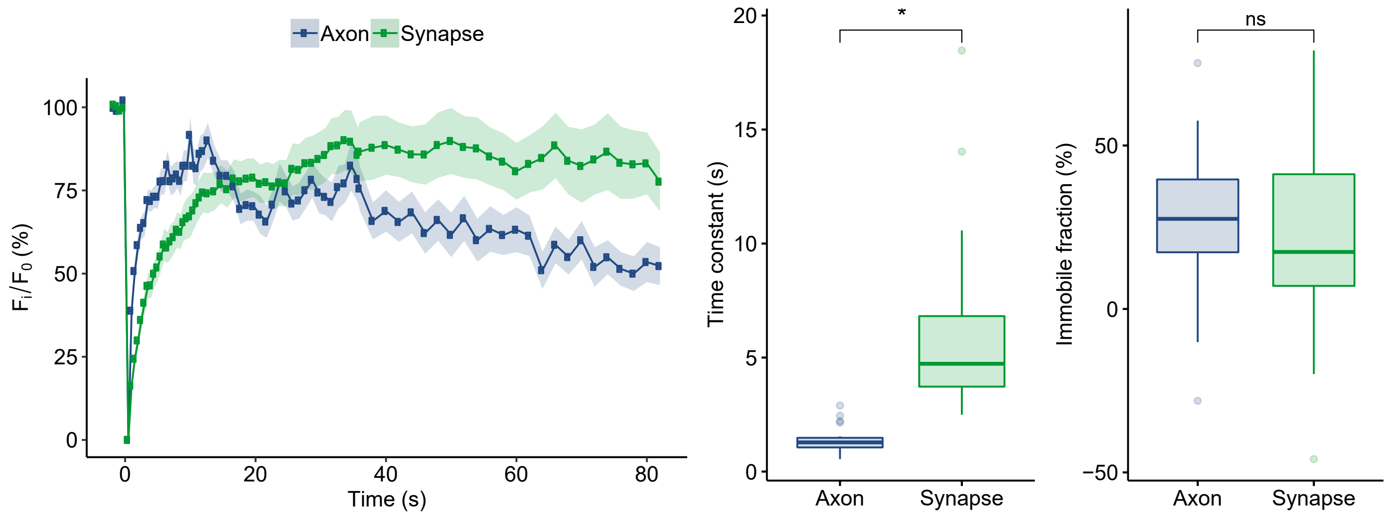

N (axons) = 24, N (synapses) = 26; p (time constant) = 3.03E-09.

Tagged protein outline:

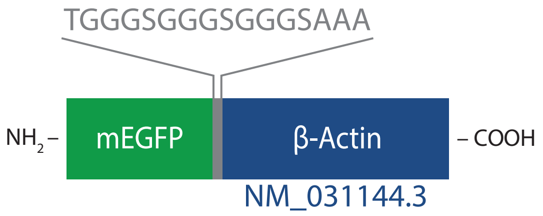

Time constant (axon) is significantly different from time constant (axon) of: Amphiphysin (p = 2.47E-04), Complexin 2 (p = 2.23E-02), Endophilin (p = 1.44E-02), PIPKgamma (p = 2.75E-02), Rab3 (p = 3.28E-03), SNAP23 (p = 6.86E-03), SNAP29 (p = 4.97E-05), Synapsin (p = 2.82E-02), Synaptotagmin 1 (p = 1.97E-03), Synaptotagmin 7 (p = 9.12E-03), Syntaxin 1 (p = 3.88E-04), Syntaxin 16 (p = 4.95E-05), VAMP 1 (p = 1.96E-04).
Immobile fraction (axon) is not significantly different from immobile fraction (axon) of any other proteins.
Time constant (synapse) is significantly different from time constant (synapse) of: Calmodulin (p = 4.78E-02), Hsc70 (p = 1.82E-02), Synapsin (p = 1.04E-02), Synaptogyrin (p = 1.08E-04), Synaptophysin (p = 1.04E-05), Synaptotagmin 1 (p = 4.59E-06), Syntaxin 16 (p = 2.66E-02), VAMP 1 (p = 2.70E-03), mEGFP (p = 5.73E-09), vGlut (p = 6.74E-05).
Immobile fraction (synapse) is significantly different from immobile fraction (synapse) of: SV2B (p = 7.07E-06), Synaptogyrin (p = 3.08E-02), Synaptophysin (p = 1.04E-05), vATPase (p = 4.02E-02), vGlut (p = 1.54E-03).

References

Sankaranarayanan, S., et al. (2003). Nat Neurosci 6, 127-35

α-SNAP

MW (kDa)	Category	τ_{axon} (s)	$\tau_{synapse}$ (s)	IF_{axon} (%)	$IF_{synapse}$ (%)
33.2	soluble, SNARE co-factor	2.40 ± 0.46	5.25 ± 0.47	9.71 ± 5.66	26.74 ± 4.27

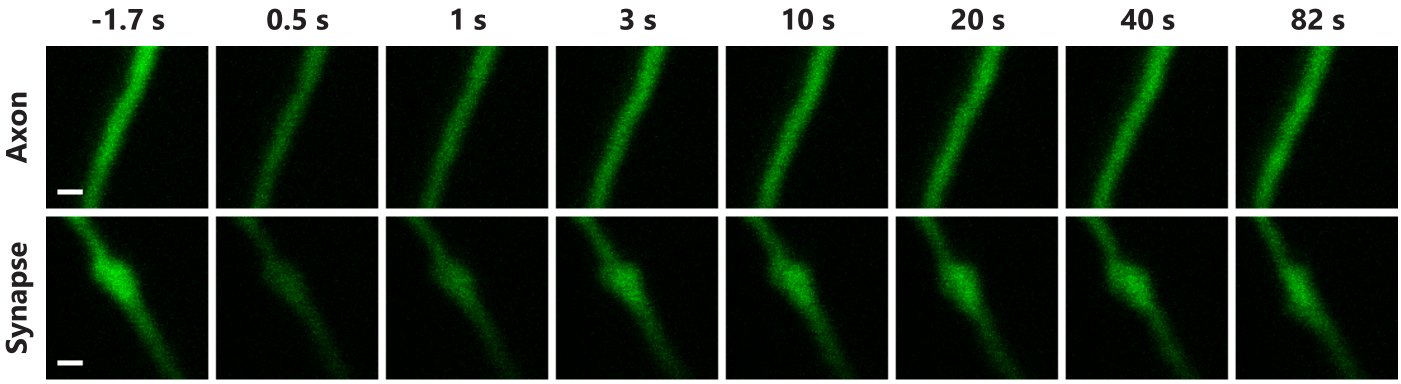

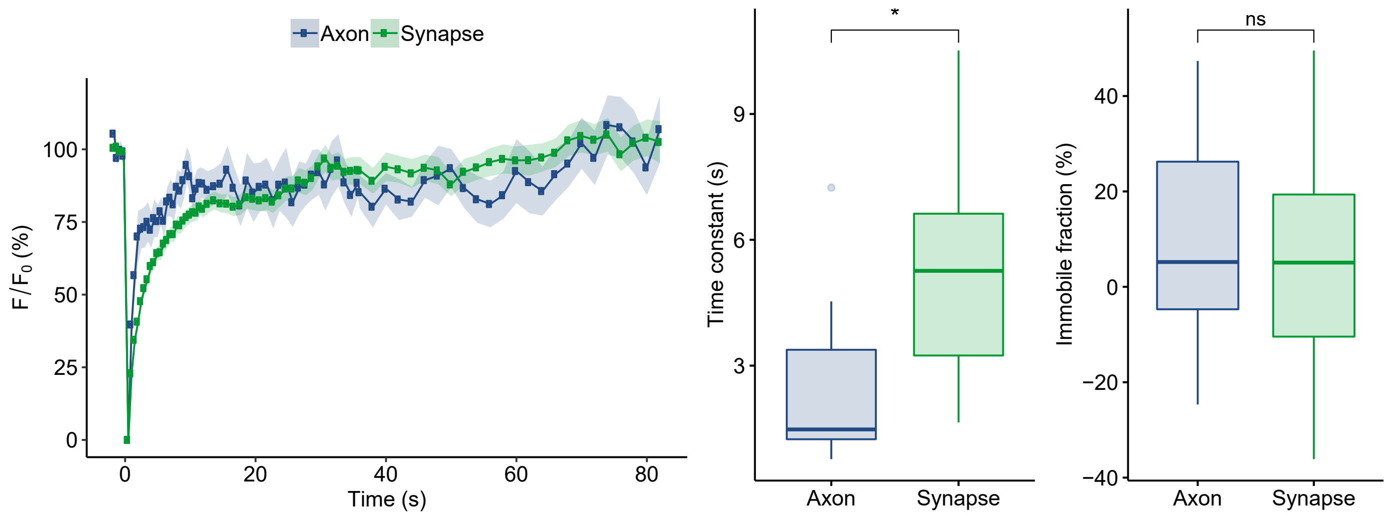

N (axons) = 15, N (synapses) = 25; p (time constant) = 2.26E-04.

Tagged protein outline:

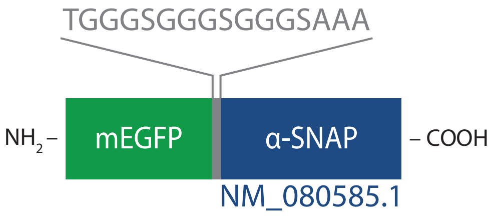

Time constant (axon) is not significantly different from time constant (axon) of any other proteins.
Immobile fraction (axon) is not significantly different from immobile fraction (axon) of any other proteins.
Time constant (synapse) is significantly different from time constant (synapse) of: mEGFP (p = 1.12E-06), SCAMP1 (p = 1.16E-02), Synapsin 1A (p = 4.39E-04), Synaptogyrin (p = 4.97E-05), Synaptophysin (p = 1.47E-06), Synaptotagmin 1 (p = 3.37E-07), Syntaxin 16 (p = 2.97E-03), alpha-Tubulin 1b (p = 3.00E-02), VAMP1 (p = 5.23E-05), vGluT1 (p = 5.86E-06).
Immobile fraction (synapse) is significantly different from immobile fraction (synapse) of: NSF (p = 1.23E-03), SCAMP1 (p = 8.31E-03), SV2B (p = 5.49E-07), Synaptogyrin (p = 7.39E-04), Synaptophysin (p = 2.06E-07), Synaptotagmin 1 (p = 4.68E-04), VAMP2 (p = 3.23E-03), VAMP4 (p = 2.25E-03), vATPase V0a1 (p = 7.38E-05), vGluT1 (p = 1.35E-05).

References

Söllner, T. (1993b). Nature 362, 318-24.
Jahn, R., and Scheller, R.H. (2006). Nat Rev Mol Cell Biol 7, 631-43.

α-synuclein

MW (kDa)	Category	τ_{axon} (s)	$\tau_{synapse}$ (s)	IF_{axon} (%)	$IF_{synapse}$ (%)
14.5	soluble, vesicle tethering	2.41 ± 0.41	6.07 ± 0.72	18.91 ± 4.30	26.74 ± 4.27

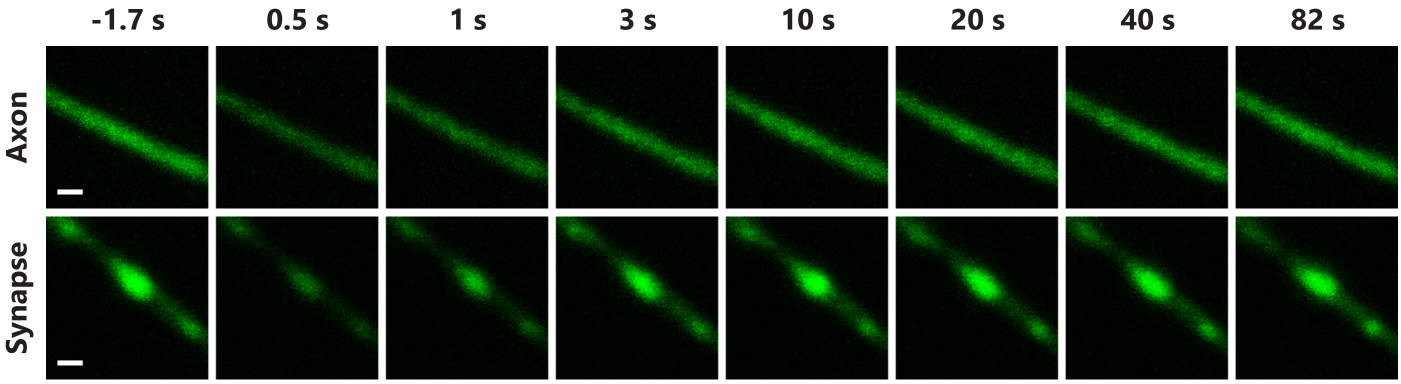

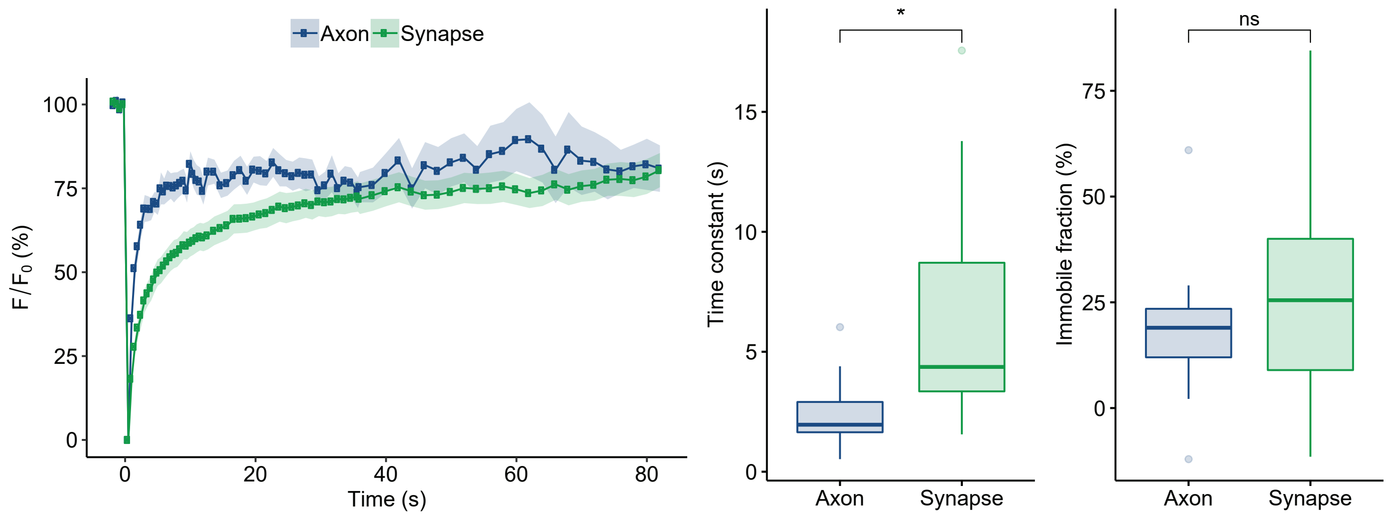

N (axons) = 14, N (synapses) = 31; p (time constant) = 3.28E-04.

Tagged protein outline:

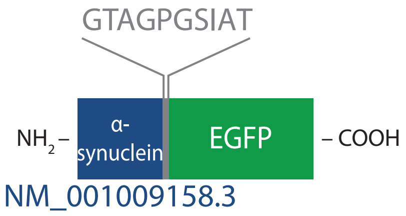

Time constant (axon) is not significantly different from time constant (axon) of any other proteins.
Immobile fraction (axon) is not significantly different from immobile fraction (axon) of any other proteins.
Time constant (synapse) is significantly different from time constant (synapse) of: mEGFP (p = 3.02E-08), Synapsin 1A (p = 4.14E-03), Synaptogyrin (p = 1.85E-05), Synaptophysin (p = 1.13E-06), Synaptotagmin 1 (p = 1.00E-06), Syntaxin 16 (p = 1.06E-02), VAMP1 (p = 1.08E-03), vGluT1 (p = 2.02E-05).
Immobile fraction (synapse) is significantly different from immobile fraction (synapse) of: SV2B (p = 2.43E-06), Synaptogyrin (p = 2.02E-02), Synaptophysin (p = 3.63E-06), vGluT1 (p = 1.69E-03).

References

Scott, D., and Roy, S. (2012). J Neurosci 32, 10129-35.
Marques, O., and Outeiro, T.F. (2012). Cell Death Dis 2, 140-51.

Amphiphysin

MW (kDa)	Category	τ_{axon} (s)	$\tau_{synapse}$ (s)	IF_{axon} (%)	$IF_{synapse}$ (%)
74.88	soluble, endocytic	3.22 ± 0.27	6.77 ± 0.43	18.62 ± 5.77	22.93 ± 6.18

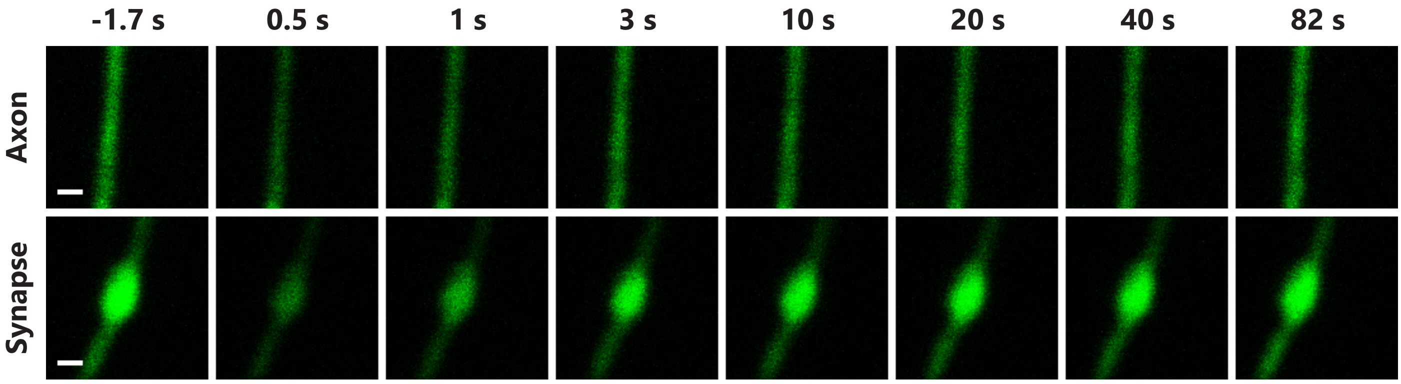

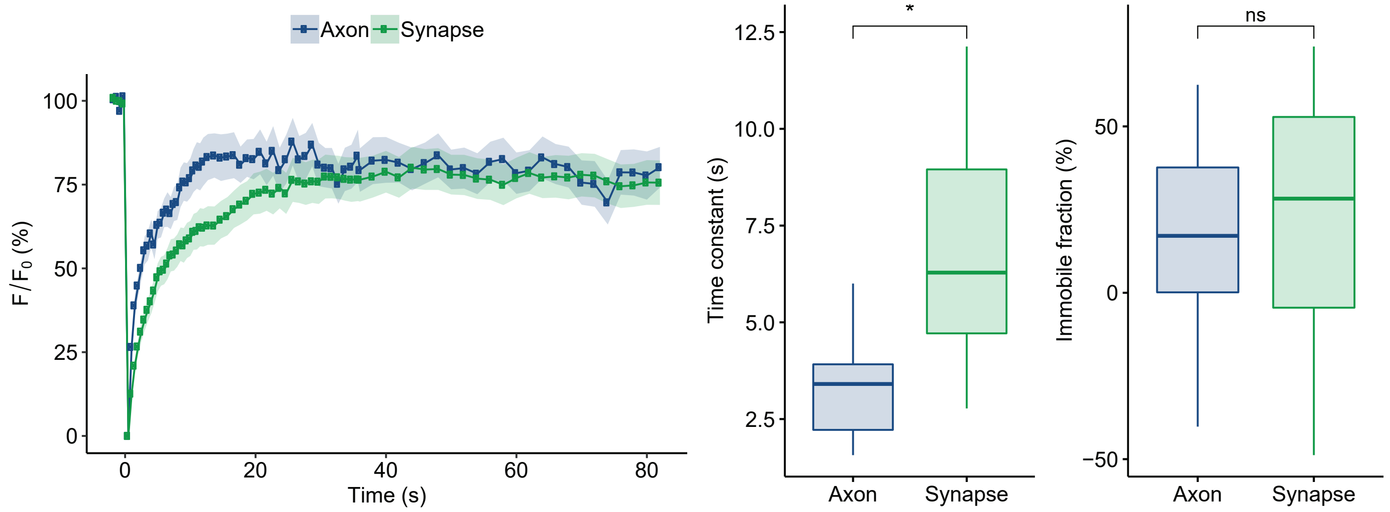

N (axons) = 22, N (synapses) = 35; p (time constant) = 1.49E-07.

Tagged protein outline:

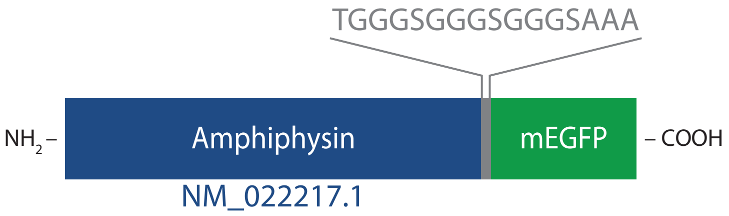

Time constant (axon) is significantly different from time constant (axon) of: Actin (p = 2.47E-04), Clathrin light chain B (p = 4.64E-02), Doc2a (p = 1.16E-02), Munc13 (p = 2.42E-04), Rab5a (p = 1.32E-02), Rab7a (p = 2.79E-02).
Immobile fraction (axon) is not significantly different from immobile fraction (axon) of any other proteins.
Time constant (synapse) is significantly different from time constant (synapse) of: AP2 (p = 2.45E-04), Calmodulin 1 (p = 9.23E-06), Clathrin light chain B (p = 5.44E-05), Complexin 1 (p = 3.63E-03), Endophilin A1 (p = 3.36E-02), Hsc70 (p = 9.52E-07), mEGFP (p = 4.74E-13), Munc13 (p = 1.07E-03), NSF (p = 1.15E-03), Rab3a (p = 3.21E-02), Rab5a (p = 3.19E-04), Rab7a (p = 1.60E-02), Septin 5 (p = 2.64E-02), SNAP23 (p = 2.71E-04), SNAP25 (p = 2.71E-03), Synapsin 1A (p = 1.34E-03), Synaptogyrin (p = 1.88E-05), Synaptophysin (p = 1.85E-07), Synaptotagmin 1 (p = 7.09E-08), VAMP1 (p = 2.45E-04), vGluT1 (p = 6.32E-06).
Immobile fraction (synapse) is significantly different from immobile fraction (synapse) of: SV2B (p = 1.24E-06), Synaptogyrin (p = 2.05E-02), Synaptophysin (p = 1.92E-06), vGluT1 (p = 2.46E-03).

References

Wigge, P., et al. (1997). Mol Biol Cell 8, 2003-15.
Shupliakov, O., et al. (1997). Science 276, 259-63.

AP180

MW (kDa)	Category	τ_{axon} (s)	$\tau_{synapse}$ (s)	IF_{axon} (%)	$IF_{synapse}$ (%)
93.52	soluble, endocytic	1.81 ± 0.21	4.51 ± 0.35	29.24 ± 4.81	7.06 ± 3.69

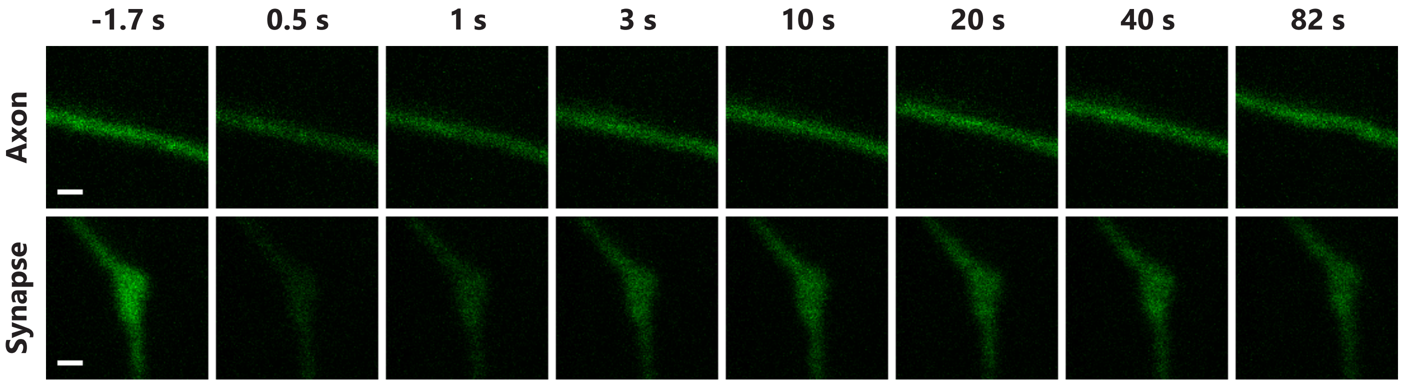

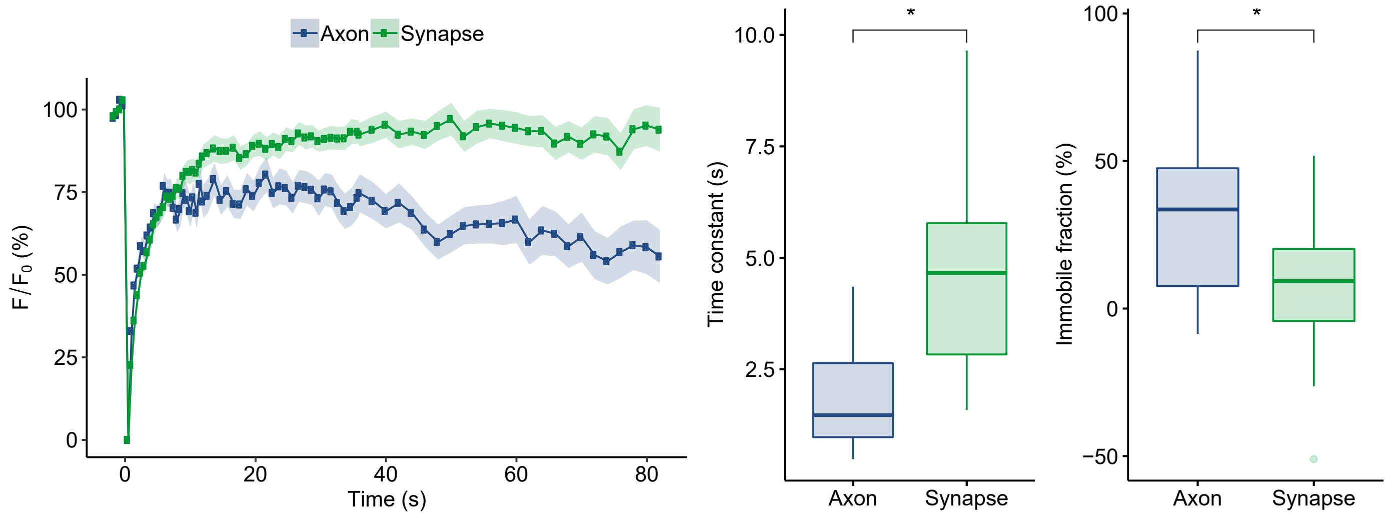

N (axons) = 27, N (synapses) = 33; p (time constant) = 1.83E-07, p (immobile fraction) = 2.44E-03.

Tagged protein outline:

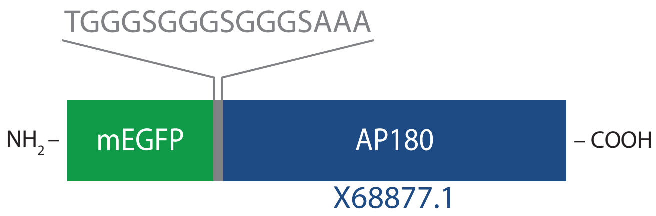

Time constant (axon) is significantly different from time constant (axon) of: SNAP29 (p = 3.55E-02), Synaptotagmin 1 (p = 1.78E-03).
Immobile fraction (axon) is not significantly different from immobile fraction (axon) of any other proteins.
Time constant (synapse) is significantly different from time constant (synapse) of: Dynamin 1 (p = 3.44E-03), mEGFP (p = 5.24E-06), SCAMP1 (p = 1.91E-04), SV2B (p = 9.12E-03), Synapsin 1A (p = 2.84E-05), Synaptogyrin (p = 3.84E-06), Synaptophysin (p = 2.55E-08), Synaptotagmin 1 (p = 6.50E-09), Syntaxin 16 (p = 1.31E-05), alpha-Tubulin 1b (p = 5.43E-04), VAMP1 (p = 1.28E-06), vATPase V0a1 (p = 2.44E-02), vGluT1 (p = 3.07E-08), Vti1a-beta (p = 4.68E-03).
Immobile fraction (synapse) is significantly different from immobile fraction (synapse) of: NSF (p = 1.21E-04), SCAMP1 (p = 3.25E-03), SV2B (p = 1.74E-08), Synaptogyrin (p = 2.45E-04), Synaptophysin (p = 4.81E-09), Synaptotagmin 1 (p = 4.74E-05), VAMP2 (p = 3.40E-04), VAMP4 (p = 6.80E-04), vATPase V0a1 (p = 5.64E-06), vGluT1 (p = 7.37E-07).

References

Morgan, J.R., et al. (2000). J Neurosci 20, 8667-76.
Nonet, M.L., et al. (1999). Mol Biol Cell 10, 2343-60.

AP2

MW (kDa)	Category	τ_{axon} (s)	$\tau_{synapse}$ (s)	IF_{axon} (%)	$IF_{synapse}$ (%)
104.05	soluble, endocytic	2.17 ± 0.17	3.56 ± 0.25	16.49 ± 3.86	7.99 ± 6.05

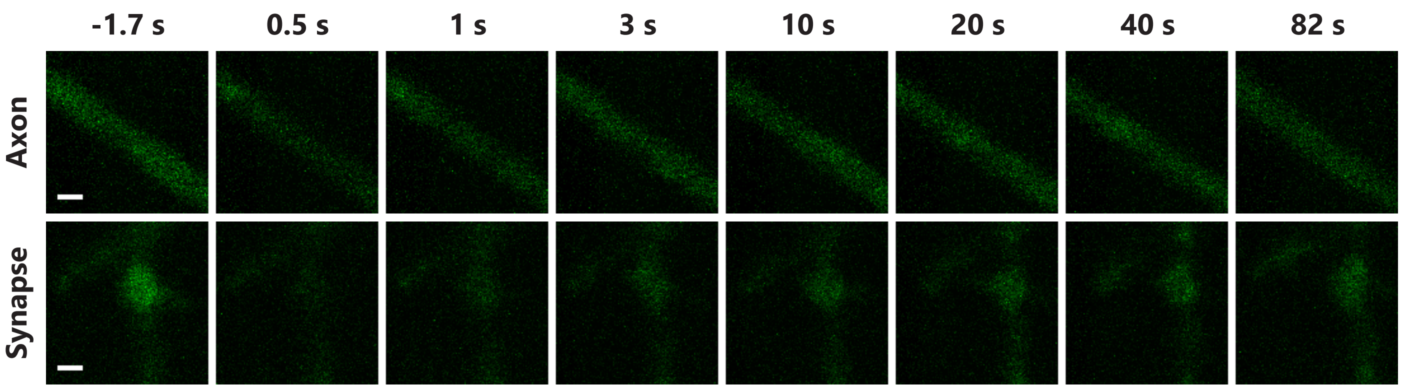

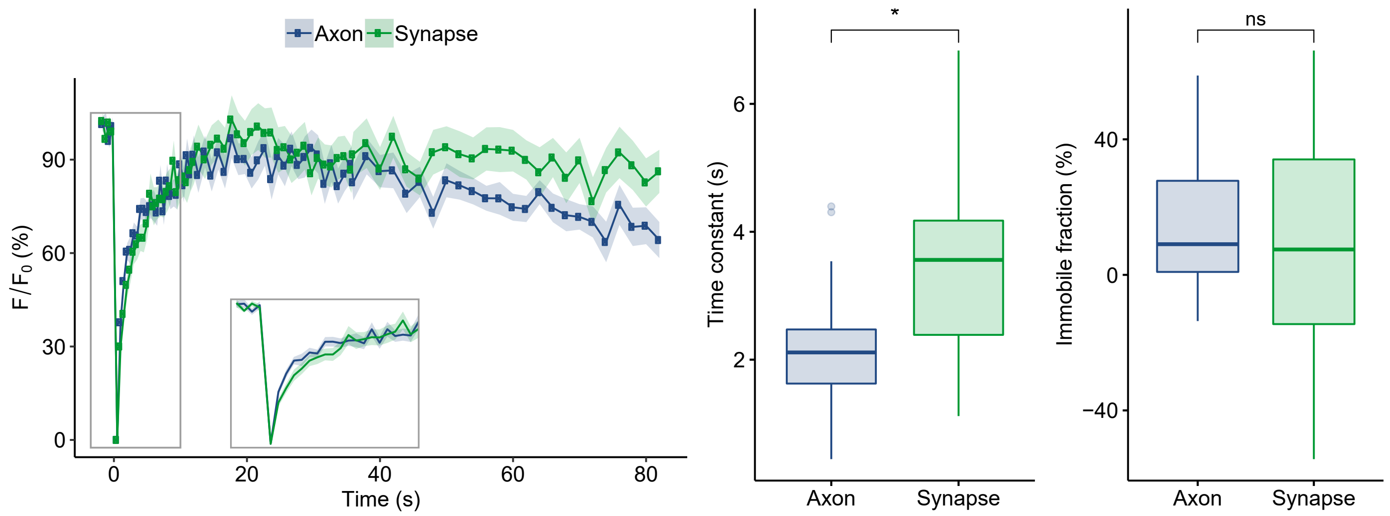

N (axons) = 28, N (synapses) = 28; p (time constant) = 1.49E-04.

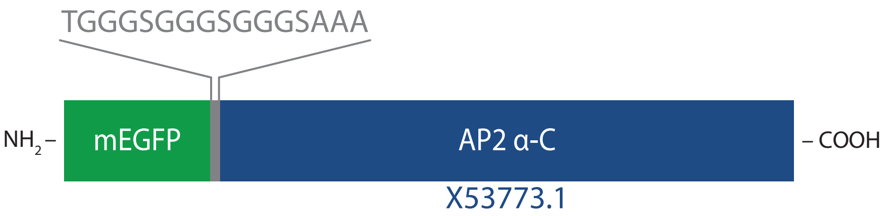

Time constant (axon) is significantly different from time constant (axon) of: Synaptotagmin 1 (p = 2.10E-03).
Immobile fraction (axon) is not significantly different from immobile fraction (axon) of any other proteins.
Time constant (synapse) is significantly different from time constant (synapse) of: Amphiphysin (p = 2.45E-04), Dynamin 1 (p = 3.03E-04), Epsin (p = 1.12E-03), , ITSN 1-L (p = 1.56E-03), mEGFP (p = 2.68E-04), PIP5KI-gamma (p = 1.77E-04), SCAMP1 (p = 2.62E-05), SV2B (p = 6.85E-03), Synapsin 1A (p = 1.07E-05), Synaptogyrin (p = 2.85E-06), Synaptophysin (p = 7.96E-08), Synaptotagmin 1 (p = 3.69E-08), Synaptotagmin 7 (p = 4.55E-03), Syntaxin 1A (p = 4.35E-04), Syntaxin 16 (p = 2.81E-07), alpha-Tubulin 1b (p = 9.69E-05), VAMP1 (p = 1.14E-06), vATPase V0a1 (p = 6.28E-03), vGluT1 (p = 5.50E-08), Vti1a-beta (p = 1.78E-03).
Immobile fraction (synapse) is significantly different from immobile fraction (synapse) of: SCAMP1 (p = 2.74E-02), SV2B (p = 6.71E-07), Synaptogyrin (p = 1.46E-03), Synaptophysin (p = 2.53E-07), Synaptotagmin 1 (p = 2.81E-03), VAMP4 (p = 4.17E-02), vATPase V0a1 (p = 4.23E-04), vGluT1 (p = 6.70E-05).

References

Honing, S., et al. (2005). Mol Cell 18, 519-31. Collins, B.M., et al. (2002). Cell 109, 523-35. Takamori, S., et al. (2006). Cell 127, 831-46.

Calmodulin 1

MW (kDa)	Category	τ_{axon} (s)	$\tau_{synapse}$ (s)	IF_{axon} (%)	$IF_{synapse}$ (%)
16.84	soluble, calcium sensor	2.68 ± 0.25	3.04 ± 0.23	13.43 ± 2.77	20.43 ± 4.83

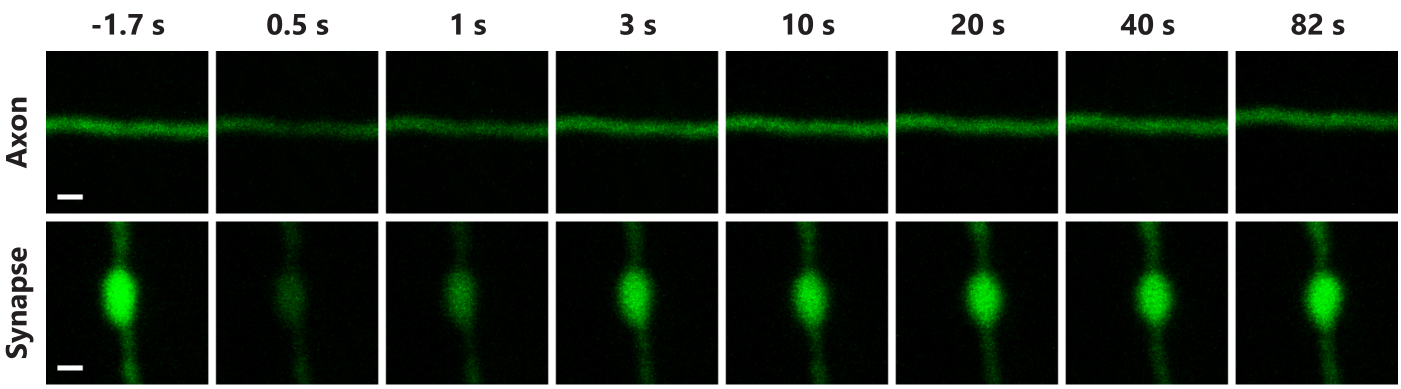

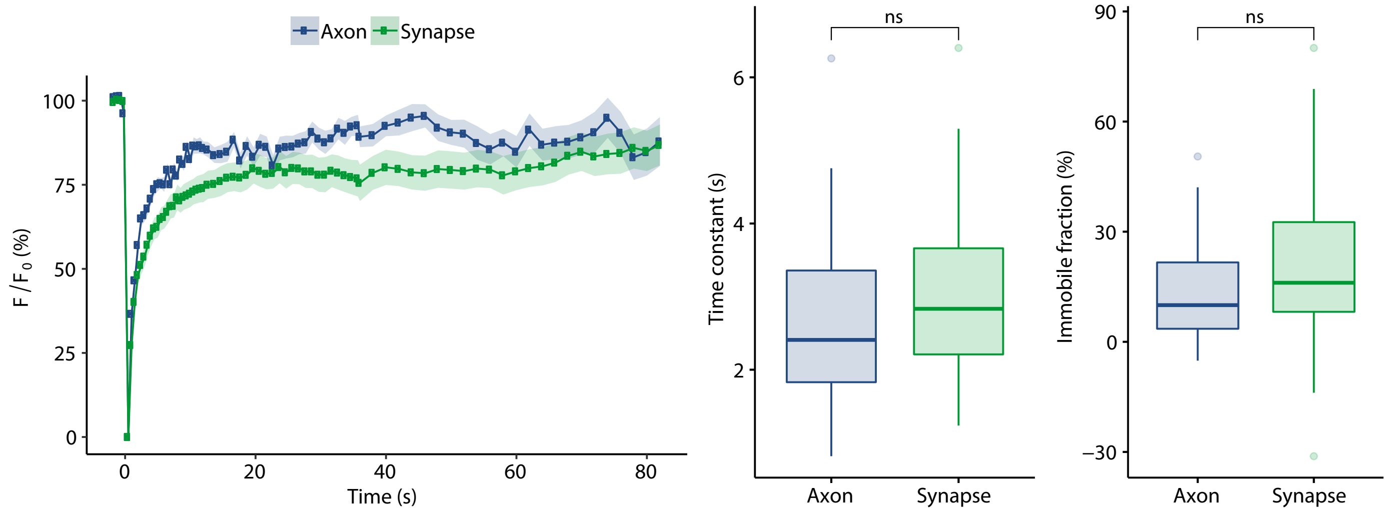

N (axons)=28, N (synapses)=28.

Tagged protein outline:

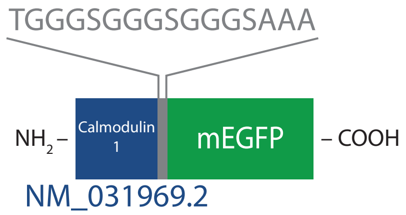

Time constant (axon) is signifi cantly different from time constant (axon) of: Synaptotagmin 1 (p = 2.13E-02). Immobile fraction (axon) is not signifi cantly different from immobile fraction (axon) of any other proteins. Time constant (synapse) is signifi cantly different from time constant (synapse) of: Actin (p = 4.78E-02), Amphiphysin (p = 9.23E06), CSP (p = 6.93E-03), Dynamin 1 (p = 1.55E-04), Epsin (p = 2.58E-04), ITSN 1-L (p = 3.96E-04), PIP5KI-gamma (p = 2.80E-05), SCAMP1 (p = 1.59E-05), SNAP29 (p = 1.67E-03), SV2B (p = 8.46E-04), Synapsin 1A (p = 4.45E-06), Synaptogyrin (p = 1.62E-06), Synaptophysin (p = 4.21E-08), Synaptotagmin 1 (p = 3.69E-08), Synaptotagmin 7 (p = 1.59E-04), Syndapin 1 (p = 1.06E-02), Syntaxin 1A (p = 2.00E-05), Syntaxin 16 (p = 4.02E-08), alpha-Tubulin 1b (p = 3.52E-05), VAMP1 (p = 4.98E-07), vATPase V0a1 (p = 1.96E-03), vGluT1 (p = 4.23E-08), Vti1a-beta (p = 5.24E-04). Immobile fraction (synapse) is signifi cantly different from immobile fraction (synapse) of: SV2B (p = 1.94E-06), Synaptogyrin (p = 1.32E-02), Synaptophysin (p = 1.98E-06), vATPase V0a1 (p = 1.37E-02), vGluT1 (p = 5.13E-04).

References

Chin, D., and Means, A.R. (2000). Trends Cell Biol 10, 322-28.
Quetglas, S., et al. (2002). EMBO J 21, 3970-9.
Igarashi, M., and Watanabe, M. (2007). Neurosci Res 58, 226-33.

Clathrin light chain

MW (kDa)	Category	τ_{axon} (s)	$\tau_{synapse}$ (s)	IF_{axon} (%)	$IF_{synapse}$ (%)
26.98	soluble, endocytic	1.61 ± 0.17	3.39 ± 0.26	23.69 ± 5.29	16.42 ± 6.71

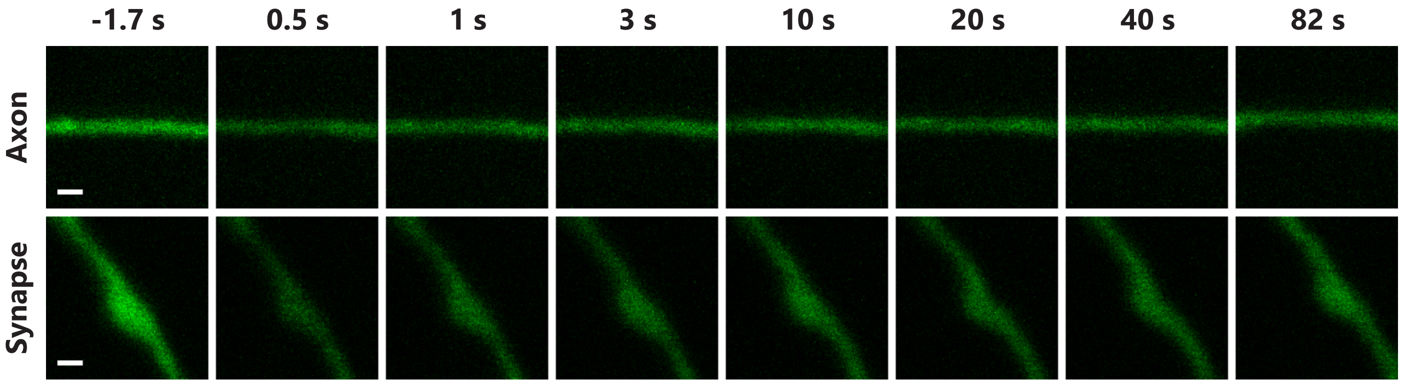

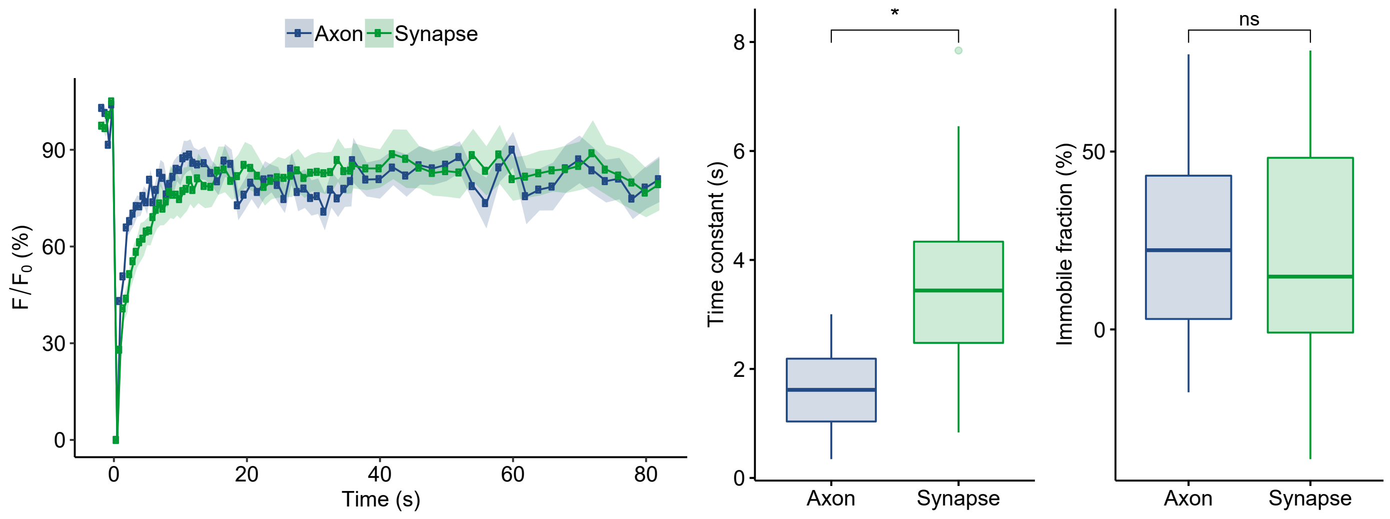

N (axons) = 22, N (synapses) = 33; p (time constant) = 6.86E-06.

Tagged protein outline:

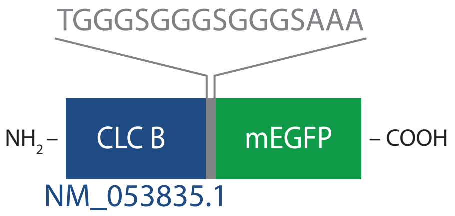

Time constant (axon) is significantly different from time constant (axon) of: Amphiphysin (p = 4.64E-02), SNAP29 (p = 5.30E-03), Synaptotagmin 1 (p = 2.01E-03), Syntaxin 1A (p = 3.07E-03), Syntaxin 16 (p = 1.74E-02), VAMP1 (p = 9.12E-03).
Immobile fraction (axon) is not significantly different from immobile fraction (axon) of any other proteins.
Time constant (synapse) is significantly different from time constant (synapse) of: Amphiphysin (p = 5.44E-05), CSP (p = 3.32E-02), Dynamin 1 (p = 2.81E-04), Epsin (p = 1.29E-03), ITSN 1-L (p = 2.89E-04), mEGFP (p = 4.03E-03), PIP5KI-gamma (p = 4.91E-05), SCAMP1 (p = 1.33E-05), SNAP29 (p = 1.62E-02), SV2B (p = 1.32E-03), Synapsin 1A (p = 3.95E-06), Synaptogyrin (p = 1.05E-06), Synaptophysin (p = 1.15E-08), Synaptotagmin 1 (p = 7.19E-09), Synaptotagmin 7 (p = 2.48E-03), Syntaxin 1A (p = 2.10E-04), Syntaxin 16 (p = 6.33E-08), alpha-Tubulin 1b (p = 3.75E-05), VAMP1 (p = 2.63E-07), vATPase V0a1 (p = 2.62E-03), vGluT1 (p = 2.09E-08), Vti1a-beta (p = 8.35E-04).
Immobile fraction (synapse) is significantly different from immobile fraction (synapse) of: SV2B (p = 6.61E-07), Synaptogyrin (p = 8.30E-03), Synaptophysin (p = 3.64E-06), vATPase V0a1 (p = 4.20E-02), vGluT1 (p = 1.13E-03).

References

Royle, S.J., Lagnado, L. (2010). Traffic 11, 1489-97.
Cheng, Y., et al. (2007). J Mol Biol 365, 892-9.
Wigge, P., et al. (1997). Mol Biol Cell 8, 2003-15.
Musacchio, A., et al. (1999). Mol Cell 3, 761-70.

Complexin 1

MW (kDa)	Category	τ_{axon} (s)	$\tau_{synapse}$ (s)	IF_{axon} (%)	$IF_{synapse}$ (%)
15.12	soluble, SNARE co-factor	2.64 ± 0.39	3.70 ± 0.40	19.24 ± 5.96	23.57 ± 2.89

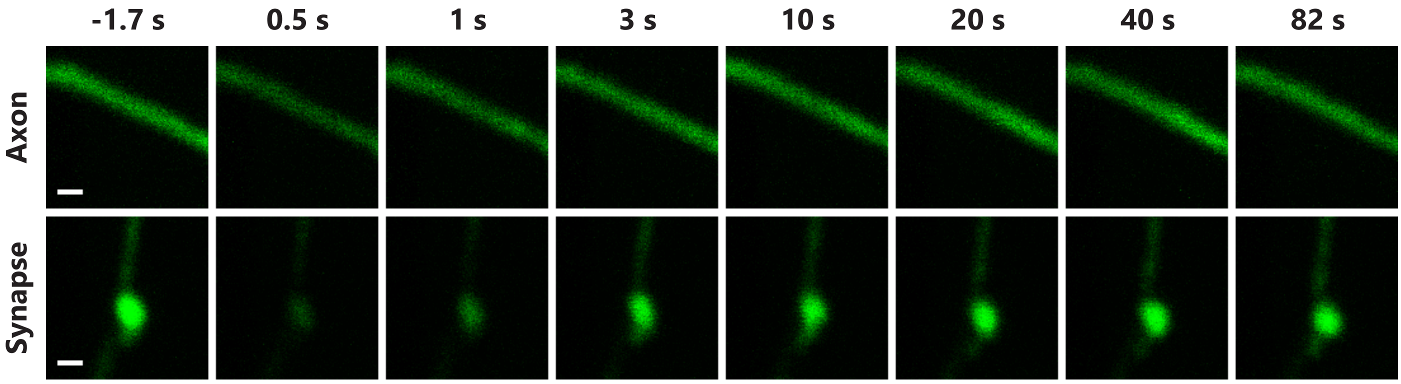

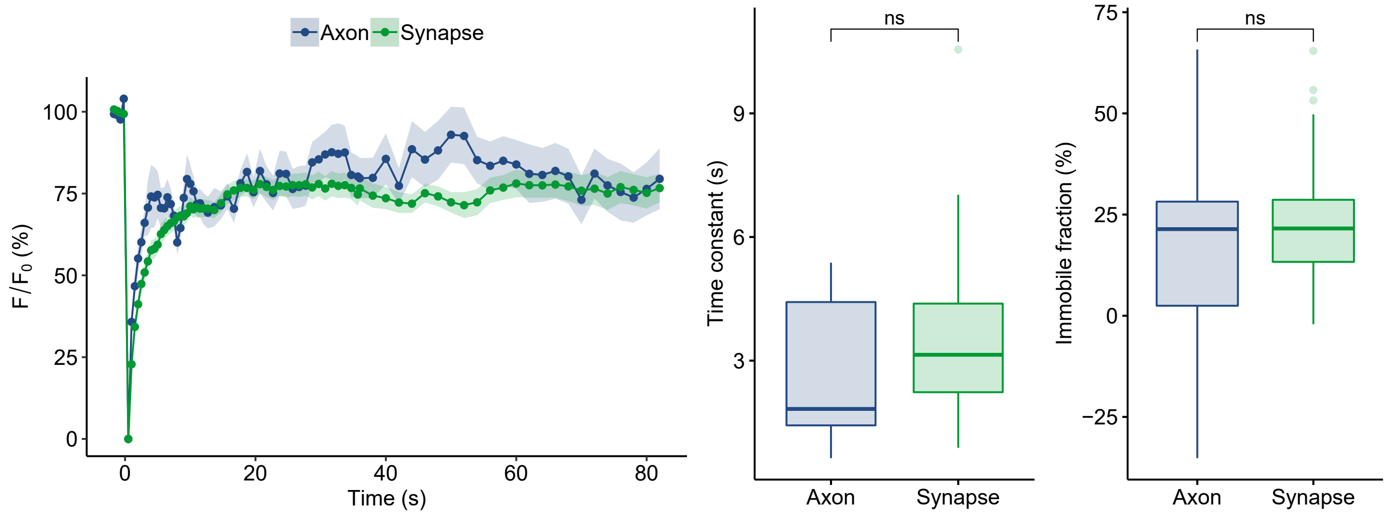

N (axons) = 18, N (synapses) = 30.

Tagged protein outline:

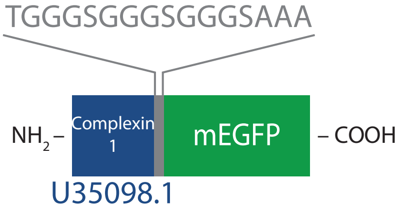

Time constant (axon) is not significantly different from time constant (axon) of any other proteins.
Immobile fraction (axon) is not significantly different from immobile fraction (axon) of any other proteins.
Time constant (synapse) is significantly different from time constant (synapse) of: Amphiphysin (p = 3.63E-03), Dynamin 1 (p = 1.00E-03), Epsin (p = 2.08E-02), ITSN 1-L (p = 4.09E-03), mEGFP (p = 2.66E-02), PIP5KI-gamma (p = 7.51E-03), SCAMP1 (p = 1.25E-04), SV2B (p = 2.04E-03), Synapsin 1A (p = 1.19E-05), Synaptogyrin (p = 2.22E-06), Synaptophysin (p = 3.82E-08), Synaptotagmin 1 (p = 2.12E-08), Syntaxin 1A (p = 1.22E-02), Syntaxin 16 (p = 5.21E-07), alpha-Tubulin 1b (p = 2.33E-04), VAMP1 (p = 1.12E-06), vATPase V0a1 (p = 6.55E-03), vGluT1 (p = 4.48E-08), Vti1a-beta (p = 1.20E-03).
Immobile fraction (synapse) is significantly different from immobile fraction (synapse) of: SV2B (p = 5.41E-07), Synaptogyrin (p = 8.84E-03), Synaptophysin (p = 1.64E-07), Synaptotagmin 1 (p = 9.74E-03), vATPase V0a1 (p = 4.99E-03), vGluT1 (p = 9.48E-05).

References

Chen, X., et al. (2002). Neuron 33, 397-409.
Bracher, A., et al. (2002). J Biol Chem 277, 26517-23.
Denker, A., et al. (2011). Proc Natl Acad Sci U S A 108, 17183-8.
Wragg, R.T., et al. (2013). Neuron 77, 323-34.

Complexin 2

MW (kDa)	Category	τ_{axon} (s)	$\tau_{synapse}$ (s)	IF_{axon} (%)	$IF_{synapse}$ (%)
15.39	soluble, SNARE co-factor	4.03 ± 0.53	5.01 ± 0.39	0.88 ± 6.23	15.44 ± 3.26

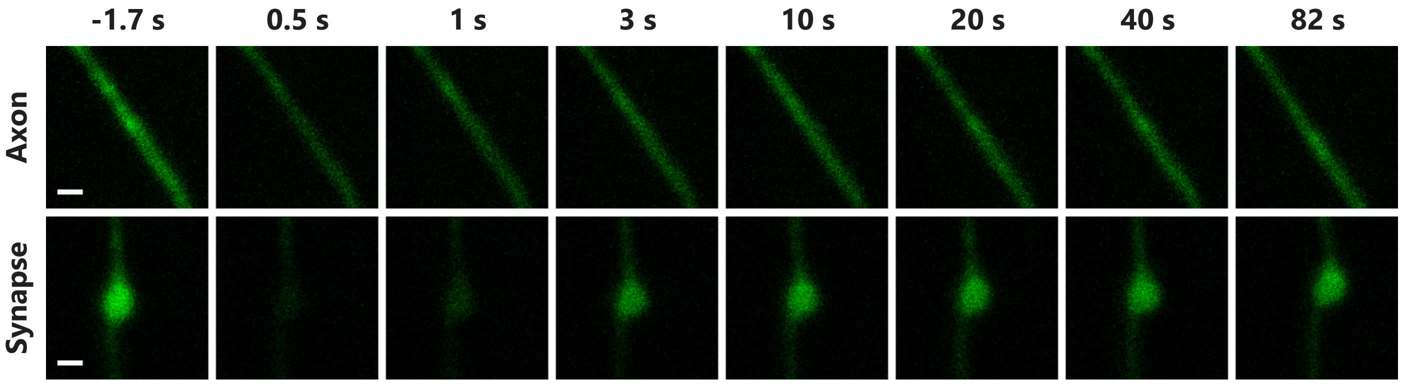

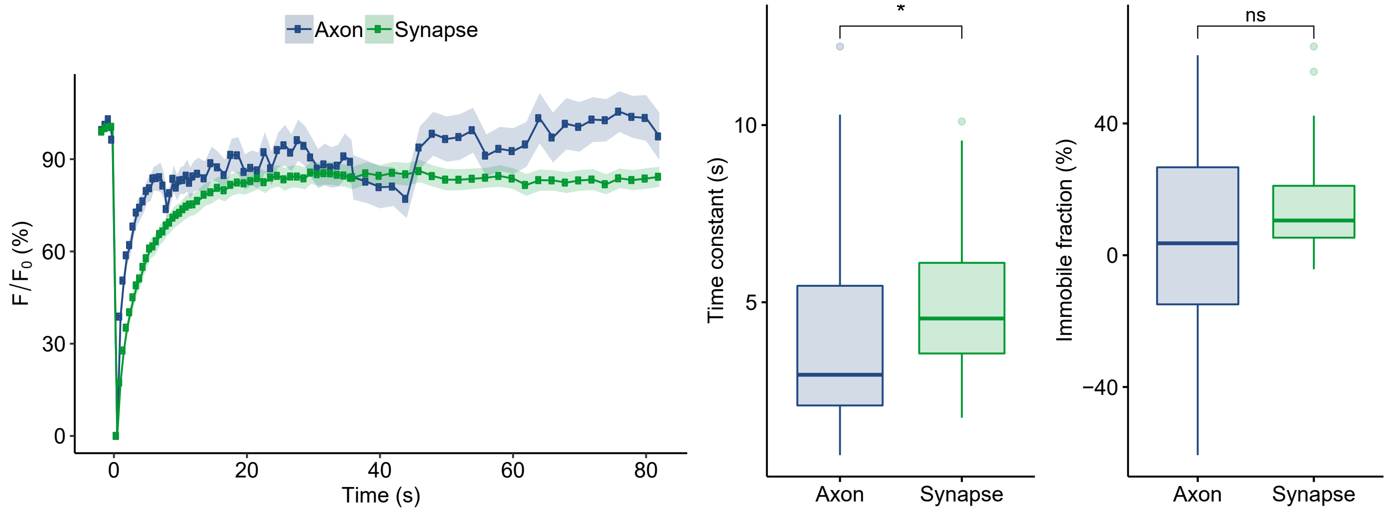

N (axons) = 27, N (synapses) = 28; p (time constant) = 3.54E-02.

Tagged protein outline:

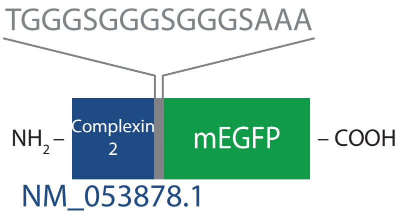

Time constant (axon) is significantly different from time constant (axon) of: Actin (p = 2.23E-02), Doc2a (p = 2.97E-02), Munc13 (p = 2.22E-03).
Immobile fraction (axon) is not significantly different from immobile fraction (axon) of any other proteins.
Time constant (synapse) is significantly different from time constant (synapse) of: Hsc70 (p = 4.69E-02), mEGFP (p = 1.05E-08), SCAMP1 (p = 2.16E-03), Synapsin 1A (p = 1.45E-04), Synaptogyrin (p = 1.48E-05), Synaptophysin (p = 3.29E-07), Synaptotagmin 1 (p = 6.71E-08), Syntaxin 16 (p = 2.41E-04), alpha-Tubulin 1b (p = 6.48E-03), VAMP1 (p = 1.66E-05), vGluT1 (p = 8.22E-07), Vti1a-beta (p = 2.87E-02).
Immobile fraction (synapse) is significantly different from immobile fraction (synapse) of: NSF (p = 5.14E-03), SCAMP1 (p = 3.02E-02), SV2B (p = 2.47E-07), Synaptogyrin (p = 1.60E-03), Synaptophysin (p = 1.04E-07), Synaptotagmin 1 (p = 1.07E-03), VAMP2 (p = 9.68E-03), VAMP4 (p = 2.79E-02), vATPase V0a1 (p = 5.79E-04), vGluT1 (p = 4.02E-05).

References

Chen, X., et al. (2002). Neuron 33, 397-409.
Bracher, A., et al. (2002). J Biol Chem 277, 26517-23.
Denker, A., et al. (2011). Proc Natl Acad Sci U S A 108, 17183-8.
Wragg, R.T., et al. (2013). Neuron 77, 323-34.

CSP

MW (kDa)	Category	τ_{axon} (s)	$\tau_{synapse}$ (s)	IF_{axon} (%)	$IF_{synapse}$ (%)
22.10	membrane-associated, SNARE co-factor	2.87 ± 0.33	8.45 ± 0.90	23.00 ± 4.60	22.05 ± 12.68

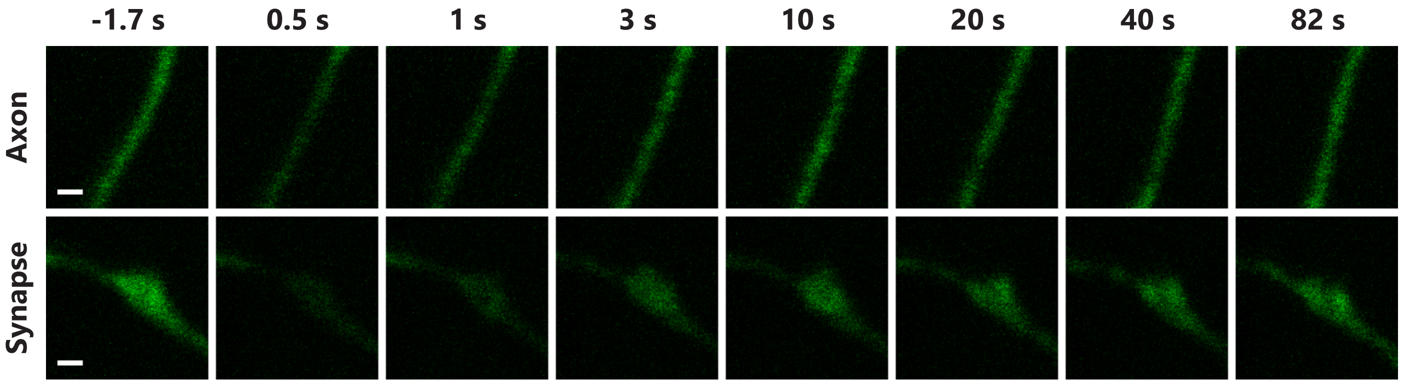

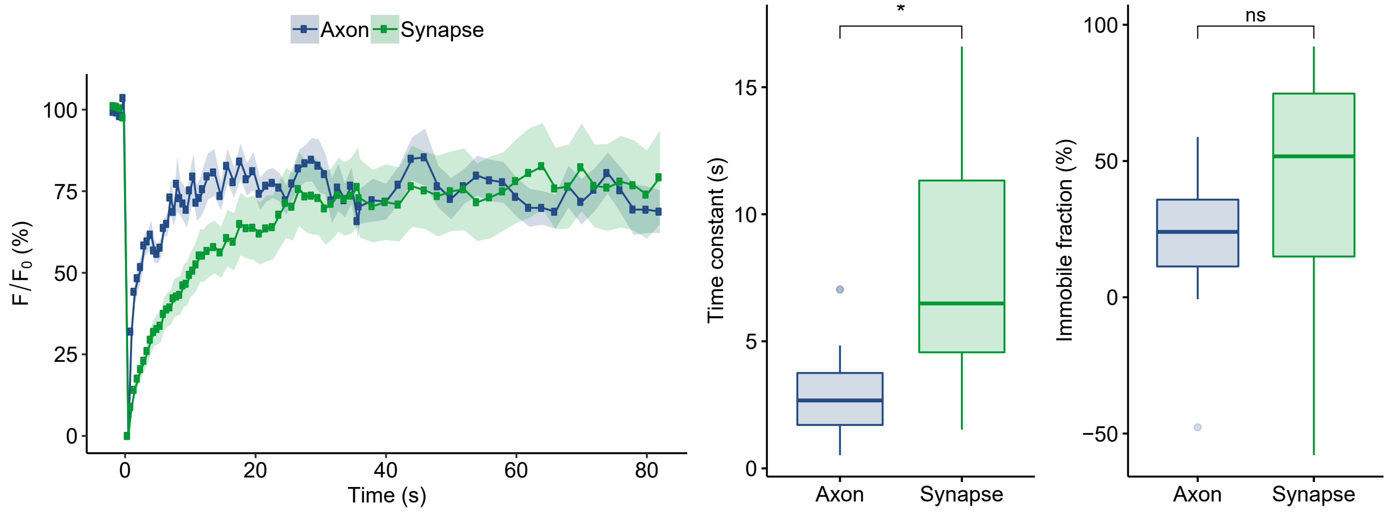

N (axons) = 25, N (synapses) = 28; p (time constant) = 1.06E-05.

Tagged protein outline:

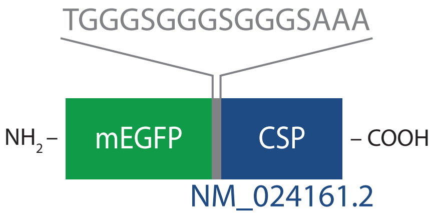

Time constant (axon) is not significantly different from time constant (axon) of any other proteins.
Immobile fraction (axon) is not significantly different from immobile fraction (axon) of any other proteins.
Time constant (synapse) is significantly different from time constant (synapse) of: Calmodulin 1 (p = 6.93E-03), Clathrin light chain B (p = 3.32E-02), Hsc70 (p = 1.48E-03), mEGFP (p = 6.02E-08), Synaptogyrin (p = 6.67E-04), Synaptophysin (p = 1.49E-04), Synaptotagmin 1 (p = 8.25E-05), vGluT1 (p = 4.34E-03).
Immobile fraction (synapse) is not significantly different from immobile fraction (synapse) of ny other proteins.

References

Fernandez-Chacon, R., et al. (2004). Neuron 42, 237-51.
Sharma, M., et al. (2011). Nat Cell Biol 13, 30-9.
Sharma, M., et al. (2011). EMBO J 31, 829-41.
Takamori, S., et al. (2006). Cell 127, 831-46.

Doc2a

MW (kDa)	Category	τ_{axon} (s)	$\tau_{synapse}$ (s)	IF_{axon} (%)	$IF_{synapse}$ (%)
44.59	soluble, calcium sensor	1.64 ± 0.09	5.47 ± 0.53	21.93 ± 2.92	21.00 ± 2.55

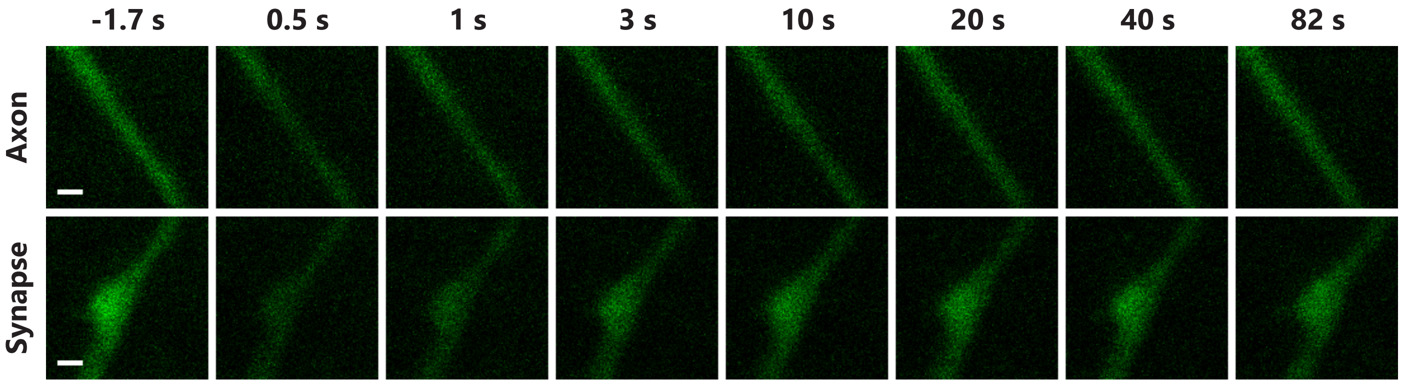

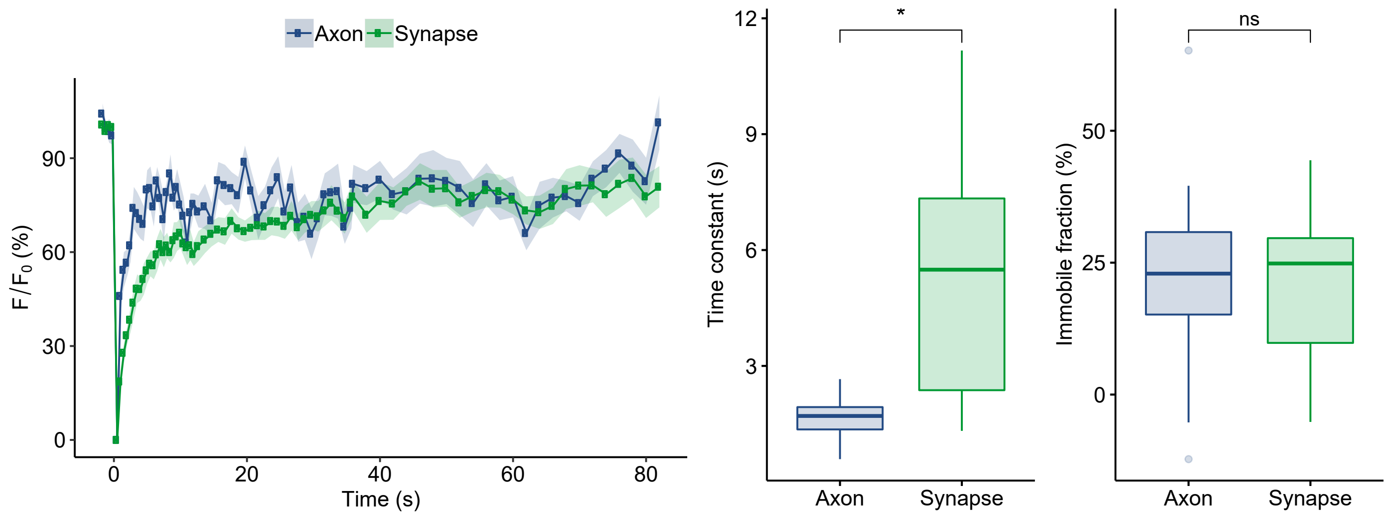

N (axons) = 29, N (synapses) = 30; p (time constant) = 2.91E-08.

Tagged protein outline:

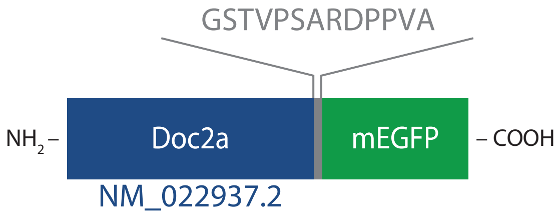

Time constant (axon) is significantly different from time constant (axon) of: Amphiphysin (p = 1.16E-02), Complexin 2 (p = 2.97E-02), SNAP29 (p = 4.27E-05), Synaptotagmin 1 (p = 9.18E-04), Syntaxin 1A (p = 8.24E-05), Syntaxin 16 (p = 5.13E-04), VAMP1 (p = 1.75E-04), Immobile fraction (axon) is not significantly different from immobile fraction (axon) of any other proteins.
Time constant (synapse) is significantly different from time constant (synapse) of: mEGFP (p = 4.57E-05), SV2B (p = 4.00E-02), Synapsin 1A (p = 3.38E-04), Synaptogyrin (p = 3.19E-05), Synaptophysin (p = 3.26E-07), Synaptotagmin 1 (p = 1.03E-07), Syntaxin 16 (p = 2.21E-03), VAMP1 (p = 5.52E-05), vGluT1 (p = 1.67E-06), Vti1a-beta (p = 4.45E-02).
Immobile fraction (synapse) is significantly different from immobile fraction (synapse) of: , SCAMP1 (p = 4.35E-02), SV2B (p = 8.17E-07), Synaptogyrin (p = 5.99E-03), Synaptophysin (p = 1.06E-07), Synaptotagmin 1 (p = 5.01E-03), VAMP4 (p = 3.60E-02), vATPase V0a1 (p = 8.52E-04), vGluT1 (p = 2.37E-05).

References

Groffen, A.J., et al. (2006). J Neurochem 97, 818-33.
Verhage et al. (1997). Neuron 18, 453-61.
Groffen, A.J., et al. (2010). Science 327, 1614-18.
Takamori, S., et al. (2006). Cell 127, 831-46.

Dynamin 1

MW (kDa)	Category	τ_{axon} (s)	$\tau_{synapse}$ (s)	IF_{axon} (%)	$IF_{synapse}$ (%)
97.29	soluble, endocytic	2.51 ± 0.33	10.75 ± 1.20	2.33 ± 5.34	21.01 ± 10.15

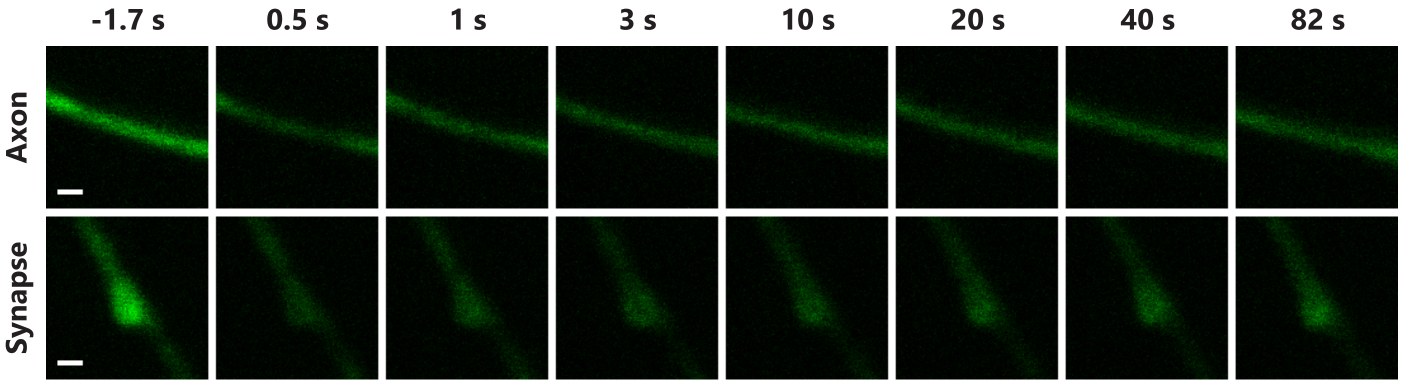

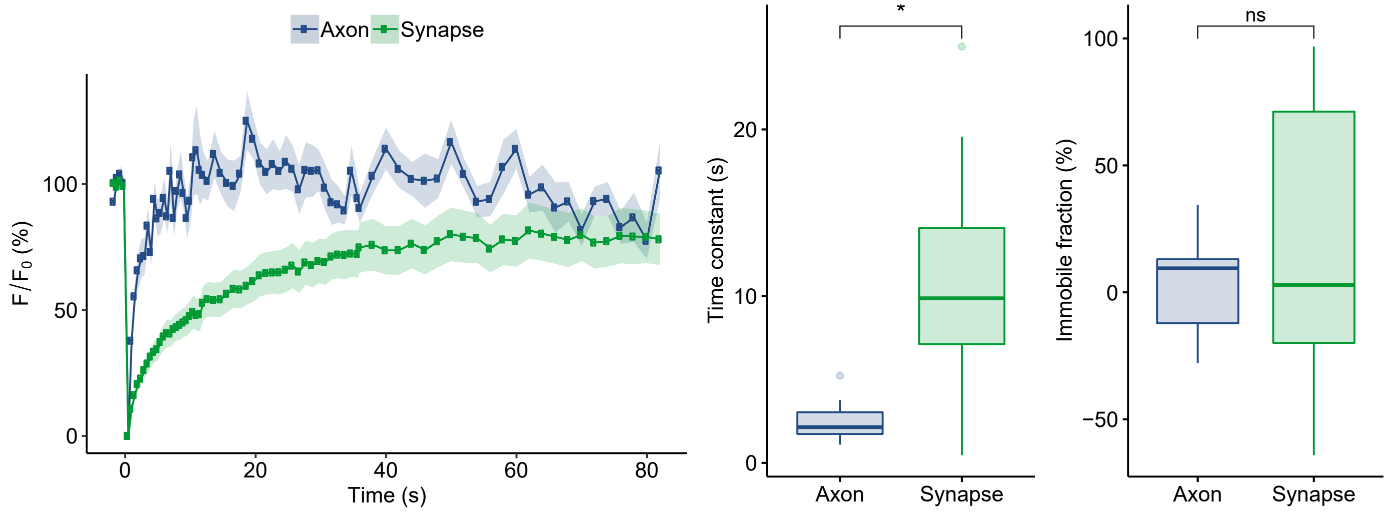

N (axons) = 12, N (synapses) = 25; p (time constant) = 1.99E-05.

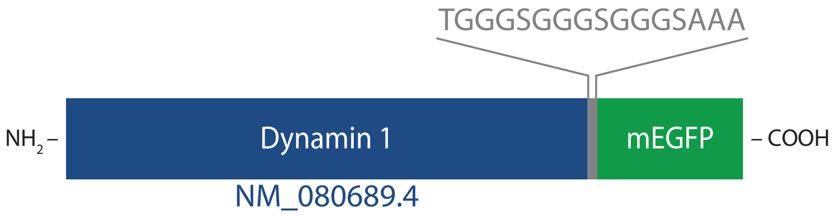

Time constant (axon) is not significantly different from time constant (axon) of any other proteins.
Immobile fraction (axon) is not significantly different from immobile fraction (axon) of any other proteins.
Time constant (synapse) is significantly different from time constant (synapse) of: AP180 (p = 3.44E-03), AP2 (p = 3.03E-04), Calmodulin 1 (p = 1.55E-04), Clathrin light chain B (p = 2.81E-04), Complexin 1 (p = 1.00E-03), Endophilin A1 (p = 3.50E-03), Hsc70 (p = 1.46E-05), mEGFP (p = 4.72E-08), membrane mEGFP (p = 2.18E-02), Munc13 (p = 1.96E-04), Munc18 (p = 3.53E-04), NSF (p = 1.01E-03), Rab3a (p = 1.31E-03), Rab5a (p = 7.14E-05), Rab7a (p = 2.07E-03), Septin 5 (p = 4.22E-03), SNAP23 (p = 1.01E-03), SNAP25 (p = 1.44E-04), Synaptogyrin (p = 7.16E-03), Synaptophysin (p = 2.57E-02), Synaptotagmin 1 (p = 4.71E-02).
Immobile fraction (synapse) is significantly different from immobile fraction (synapse) of: SV2B (p = 4.80E-03).

References

Ferguson, S.M., and De Camili, P. (2012). Nat Rev Mol Cell Biol 13, 75-88.
Roux, A., et al. (2006). Nature 441, 528-31.
Koenig, J.H., and Ikeda, K. (1989). J Neurosci 9, 3844-60.
Takamori, S., et al. (2006). Cell 127, 831-46.

Endophilin

MW (kDa)	Category	τ_{axon} (s)	$\tau_{synapse}$ (s)	IF_{axon} (%)	$IF_{synapse}$ (%)
39.90	soluble, endocytic	2.52 ± 0.21	4.28 ± 0.28	9.87 ± 2.53	17.98 ± 3.68

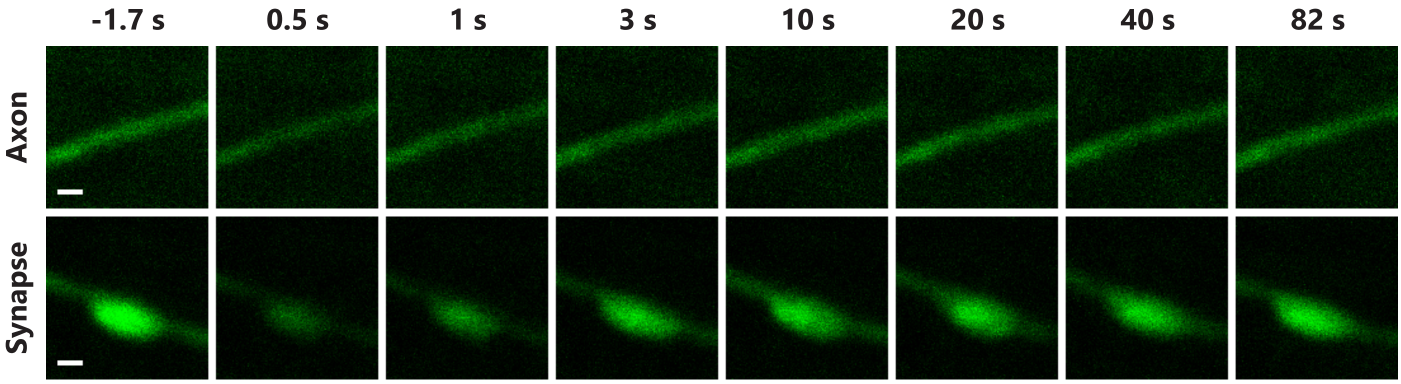

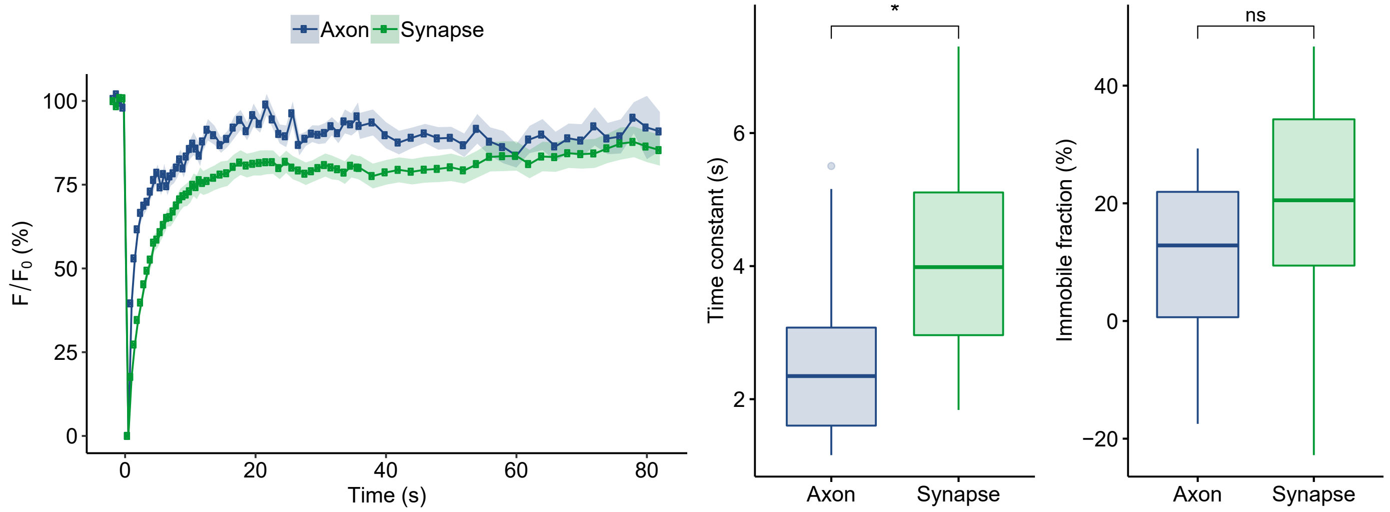

N (axons) = 28 N (synapses) = 28; p (time constant) = 1.26E-05.

Tagged protein outline:

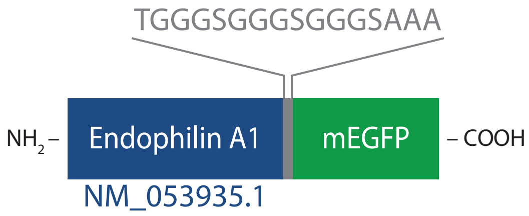

Time constant (axon) is significantly different from time constant (axon) of: Actin (p = 1.44E-02), Munc13 (p = 2.19E-02), Synaptotagmin 1 (p = 8.12E-03).
Immobile fraction (axon) is significantly different from immobile fraction (axon) of: Syndapin 1 (p = 1.12E-02).
Time constant (synapse) is significantly different from time constant (synapse) of: Amphiphysin (p = 3.36E-02), Dynamin 1 (p = 3.50E-03), ITSN 1-L (p = 1.32E-02), mEGFP (p = 9.94E-09), PIP5KI-gamma (p = 2.37E-02), SCAMP1 (p = 1.28E-04), Synapsin 1A (p = 3.45E-05), Synaptogyrin (p = 5.55E-06), Synaptophysin (p = 1.78E-07), Synaptotagmin 1 (p = 3.69E-08), Syntaxin 16 (p = 4.96E-06), alpha-Tubulin 1b (p = 9.35E-04), VAMP1 (p = 2.84E-06), vATPase V0a1 (p = 4.01E-02), vGluT1 (p = 1.42E-07), Vti1a-beta (p = 9.68E-03).
Immobile fraction (synapse) is significantly different from immobile fraction (synapse) of: SCAMP1 (p = 2.49E-02), SV2B (p = 9.59E-07), Synaptogyrin (p = 5.14E-03), Synaptophysin (p = 1.94E-07), Synaptotagmin 1 (p = 4.33E-03), VAMP4 (p = 3.02E-02), vATPase V0a1 (p = 6.16E-04), vGluT1 (p = 3.73E-05).

References

Schuske, K.R., et al. (2003). Nauron 40, 749-62.
Sundborger, A., et al. (2011). J Cell Sci 124, 133-43.
Kjaerulff, O., et al. (2010). Cell Biochem Biophys 60, 137-54.

Epsin

MW (kDa)	Category	τ_{axon} (s)	$\tau_{synapse}$ (s)	IF_{axon} (%)	$IF_{synapse}$ (%)
60.16	soluble, endocytic	2.63 ± 0.32	6.78 ± 0.53	24.51 ± 4.89	17.65 ± 5.35

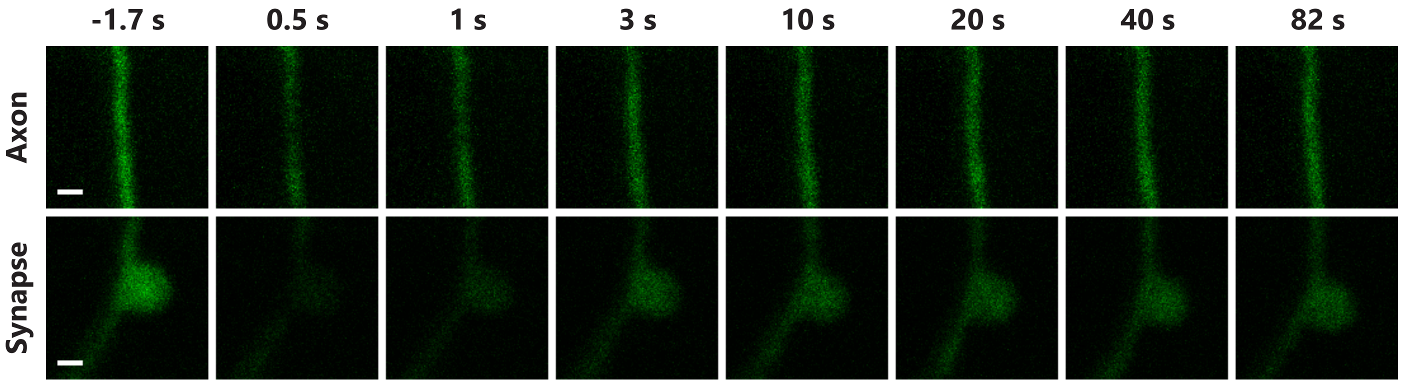

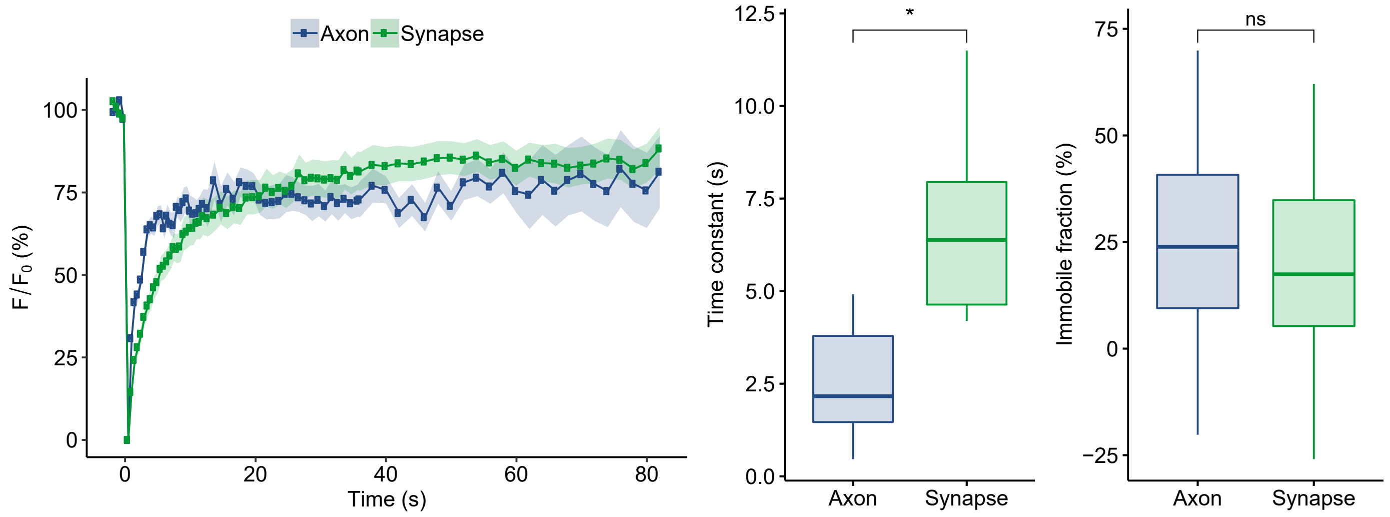

N (axons) = 19, N (synapses) = 19; p (time constant) = 5.97E-07.

Tagged protein outline:

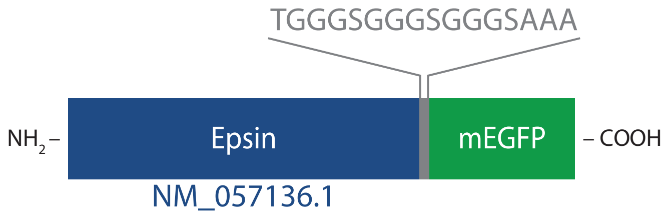

Time constant (axon) is not significantly different from time constant (axon) of any other proteins.
Immobile fraction (axon) is not significantly different from immobile fraction (axon) of any other proteins.
Time constant (synapse) is significantly different from time constant (synapse) of: AP2 (p = 1.12E-03), Calmodulin 1 (p = 2.58E-04), Clathrin light chain B (p = 1.29E-03), Complexin 1 (p = 2.08E-02), Hsc70 (p = 1.19E-04), mEGFP (p = 4.64E-08), Munc13 (p = 1.32E-02), NSF (p = 1.57E-02), Rab5a (p = 5.39E-03), SNAP23 (p = 1.70E-03), Synapsin 1A (p = 3.75E-02), Synaptogyrin (p = 2.17E-03), Synaptophysin (p = 1.15E-04), Synaptotagmin 1 (p = 2.10E-05), VAMP1 (p = 6.83E-03), vGluT1 (p = 8.87E-04).
Immobile fraction (synapse) is significantly different from immobile fraction (synapse) of: SV2B (p = 4.21E-05), Synaptogyrin (p = 3.02E-02), Synaptophysin (p = 2.59E-05), vATPase V0a1 (p = 2.98E-02), vGluT1 (p = 2.68E-03).

References

Horvath, C.A., et al. (2007). Int J Biochem Cell Biol 39, 1765-70.
Ford, M.G., et al. (2002). Nature 419, 361-6.

Hsc70

MW (kDa)	Category	τ_{axon} (s)	$\tau_{synapse}$ (s)	IF_{axon} (%)	$IF_{synapse}$ (%)
70.87	soluble, endocytic	2.63 ± 0.28	2.92 ± 0.24	14.69 ± 4.30	24.41 ± 3.00

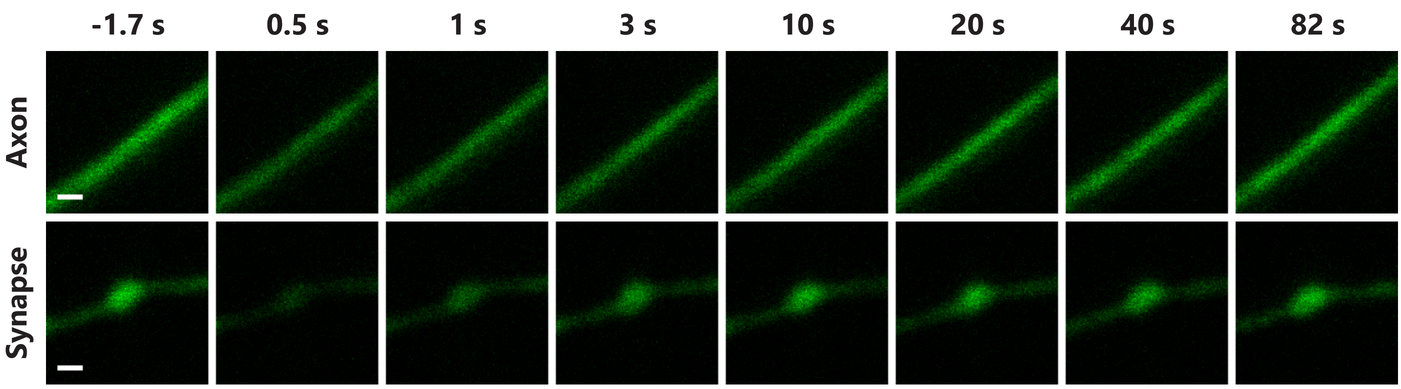

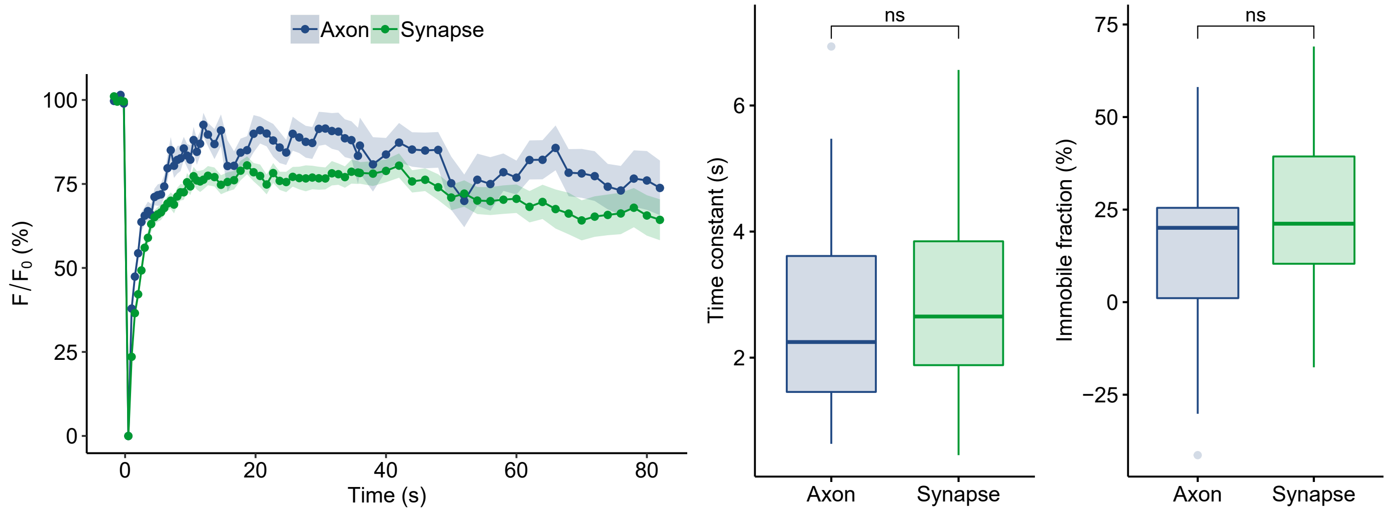

N (axons) = 29, N (synapses) = 40.

Tagged protein outline:

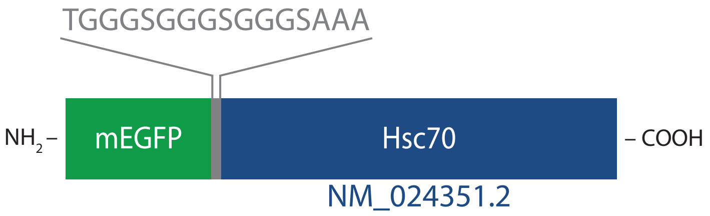

Time constant (axon) is significantly different from time constant (axon) of: Synaptotagmin 1 (p = 3.80E-02).
Immobile fraction (axon) is not significantly different from immobile fraction (axon) of any other proteins.
Time constant (synapse) is significantly different from time constant (synapse) of: Actin (p = 1.82E-02), Amphiphysin (p = 9.52E-07), Complexin 2 (p = 4.69E-02), CSP (p = 1.48E-03), Dynamin 1 (p = 1.46E-05), Epsin (p = 1.19E-04), ITSN 1-L (p = 2.81E-05), Munc18 (p = 1.49E-02), PIP5KI-gamma (p = 7.79E-06), SCAMP1 (p = 1.51E-06), SNAP29 (p = 5.47E-04), SV2B (p = 1.82E-05), Synapsin 1A (p = 3.53E-07), Synaptogyrin (p = 9.92E-08), Synaptophysin (p = 4.40E-10), Synaptotagmin 1 (p = 4.70E-10), Synaptotagmin 7 (p = 7.21E-05), Syndapin 1 (p = 2.09E-03), Syntaxin 1A (p = 5.02E-06), Syntaxin 16 (p = 7.36E-10), alpha-Tubulin 1b (p = 1.60E-06), VAMP1 (p = 1.40E-08), VAMP2 (p = 1.71E-02), vATPase V0a1 (p = 5.56E-05), vGluT1 (p = 3.36E-10), Vti1a-beta (p = 3.39E-05).
Immobile fraction (synapse) is significantly different from immobile fraction (synapse) of: , Rab5a (p = 4.95E-02), SV2B (p = 5.25E-08), Synaptogyrin (p = 2.96E-03), Synaptophysin (p = 1.25E-08), Synaptotagmin 1 (p = 1.12E-02), vATPase V0a1 (p = 2.10E-03), vGluT1 (p = 3.28E-05).

References

Schlossman, D.M., et al. (1984). J Cell Biol 99, 723-33.
Rothnie, A., et al. (2011). Proc Natl Acad Sci U S A 108, 6927-32.
Cremona, O., et al. (1999). Cell 99, 179-88.

Intersectin 1-L

MW (kDa)	Category	τ_{axon} (s)	$\tau_{synapse}$ (s)	IF_{axon} (%)	$IF_{synapse}$ (%)
194.20	soluble, endocytic	1.89 ± 0.23	9.52 ± 0.88	26.14 ± 7.26	40.73 ± 7.23

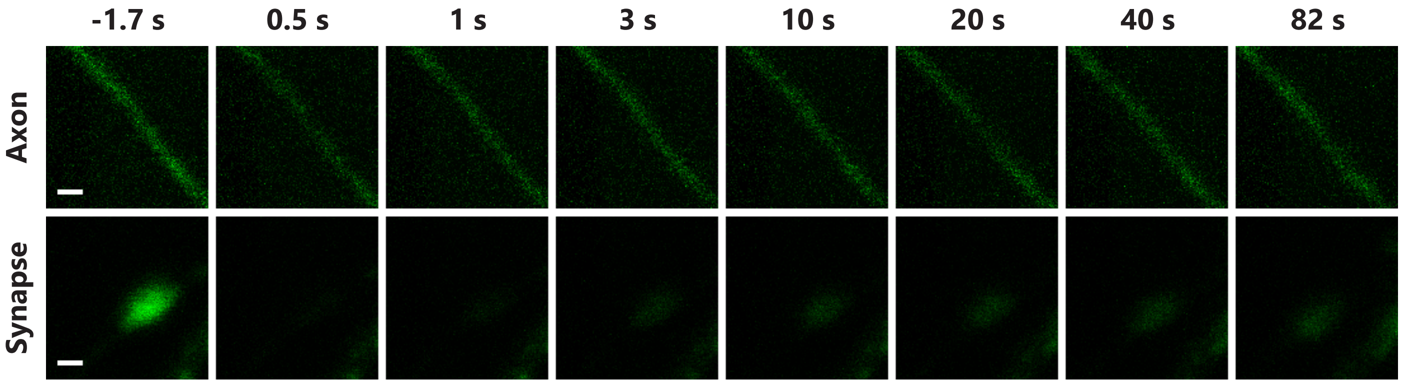

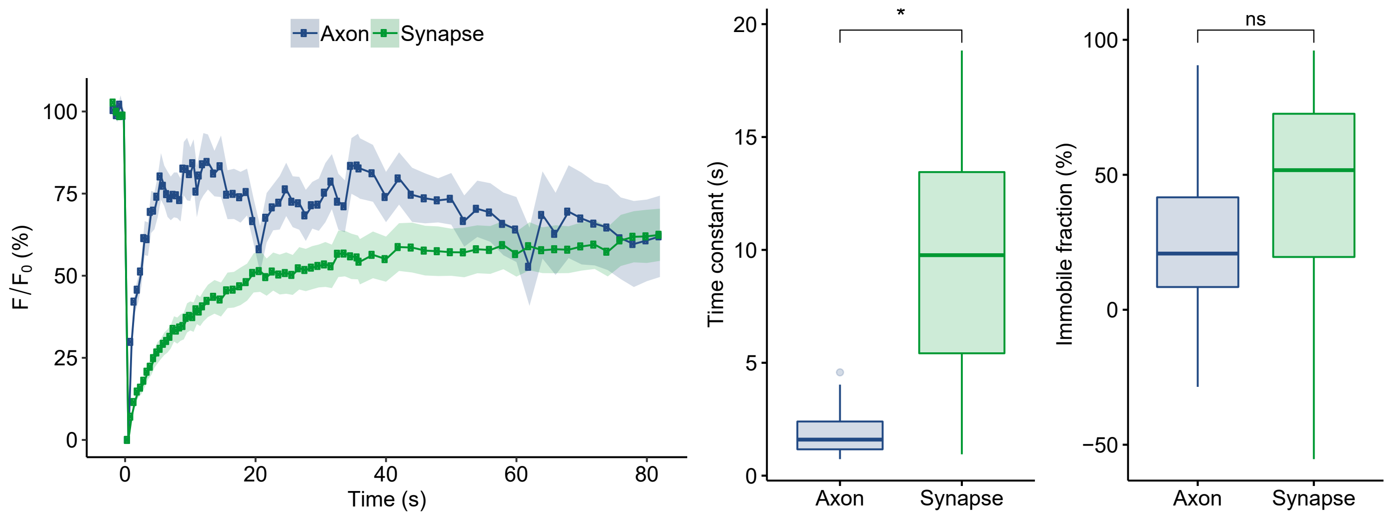

N (axons) = 20, N (synapses) = 32; p (time constant) = 2.97E-07.

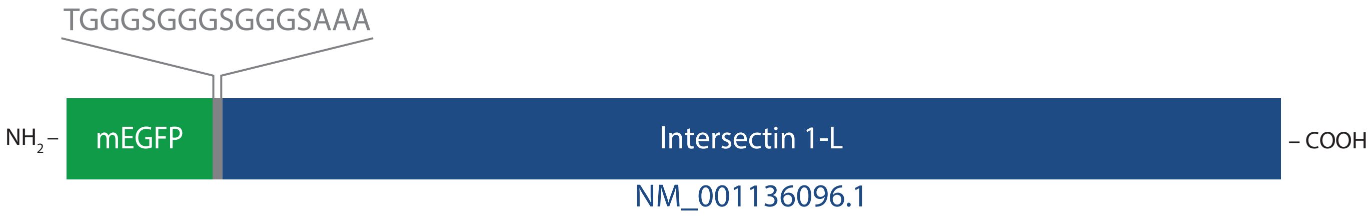

Time constant (axon) is significantly different from time constant (axon) of: SNAP29 (p = 4.56E-02), Synaptotagmin 1 (p = 1.35E-02).
Immobile fraction (axon) is not significantly different from immobile fraction (axon) of any other proteins.
Time constant (synapse) is significantly different from time constant (synapse) of: AP2 (p = 1.56E-03), Calmodulin 1 (p = 3.96E-04), Clathrin light chain B (p = 2.89E-04), Complexin 1 (p = 4.09E-03), Endophilin A1 (p = 1.32E-02), Hsc70 (p = 2.81E-05), mEGFP (p = 4.96E-08), Munc13 (p = 6.76E-04), Munc18 (p = 1.13E-03), NSF (p = 6.07E-03), Rab3a (p = 9.89E-03), Rab5a (p = 3.17E-04), Rab7a (p = 9.36E-03), Septin 5 (p = 6.14E-03), SNAP23 (p = 3.48E-03), SNAP25 (p = 3.49E-04), Synaptogyrin (p = 7.83E-04), Synaptophysin (p = 6.11E-04), Synaptotagmin 1 (p = 1.75E-03).
Immobile fraction (synapse) is significantly different from immobile fraction (synapse) of: Rab5a (p = 3.67E-02), SV2B (p = 1.03E-02).

References

Pechstein, A., et al. (2010a). Proc Natl Acad Sci U S A 107, 4206-11.
Pechstein, A., et al. (2010b). Biochem Soc Trans 38, 181-6.
Gerth, F. et al. Proc. Natl. Acad. Sci. U.S.A. 114, 12057–12062 (2017).

mEGFP

MW (kDa)	Category	τ_{axon} (s)	$\tau_{synapse}$ (s)	IF_{axon} (%)	$IF_{synapse}$ (%)
26.92	soluble, control	1.94 ± 0.12	2.08 ± 0.08	20.47 ± 2.32	18.15 ± 1.65

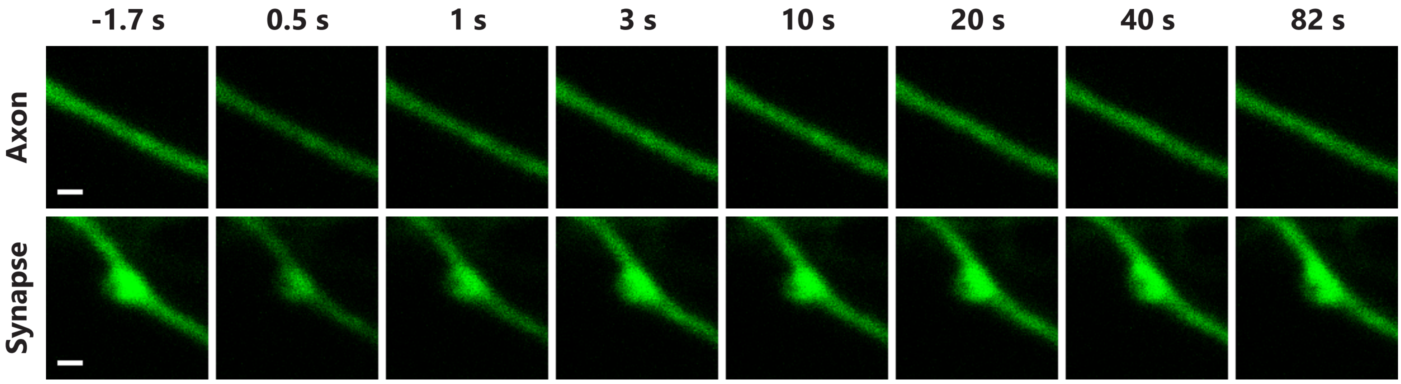

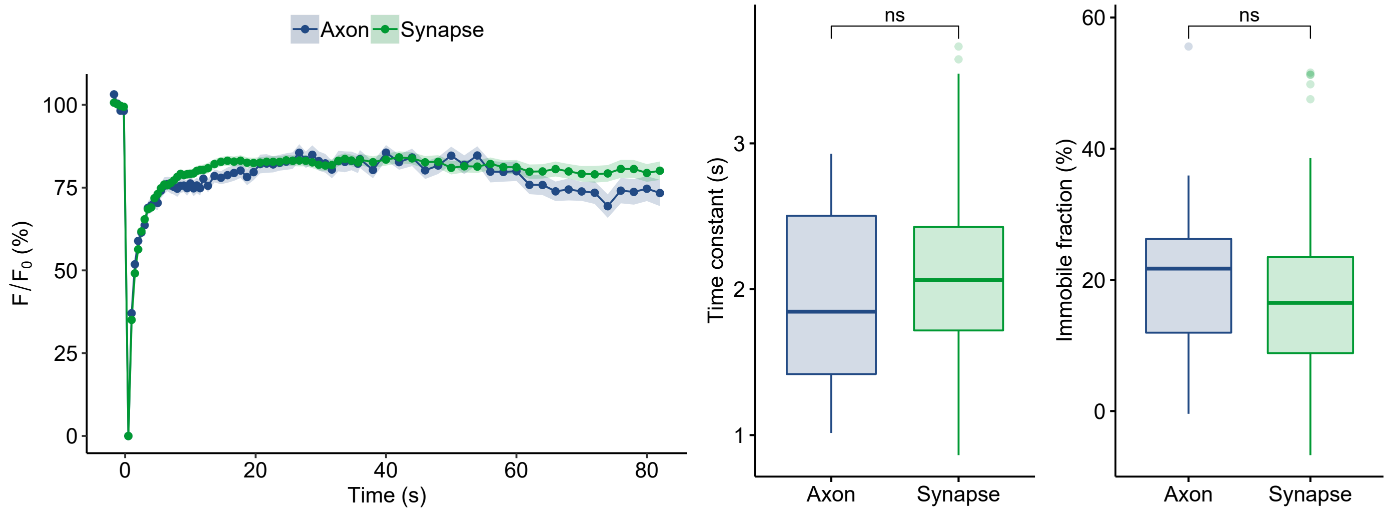

N (axons) = 26, N (synapses) = 65.

Tagged protein outline: mEGFP

Time constant (axon) is significantly different from time constant (axon) of: SNAP29 (p = 5.91E-03), Synaptotagmin 1 (p = 1.50E-03), Syntaxin 1A (p = 1.50E-03), VAMP1 (p = 1.21E-02).
Immobile fraction (axon) is not significantly different from immobile fraction (axon) of any other proteins.
Time constant (synapse) is significantly different from time constant (synapse) of: Actin (p = 5.73E-09), alpha-SNAP (p = 1.12E-06), alpha-synuclein (p = 3.02E-08), Amphiphysin (p = 4.74E-13), AP180 (p = 5.24E-06), AP2 (p = 2.68E-04), Clathrin light chain B (p = 4.03E-03), Complexin 1 (p = 2.66E-02), Complexin 2 (p = 1.05E-08), CSP (p = 6.02E-08), Doc2a (p = 4.57E-05), Dynamin 1 (p = 4.72E-08), Endophilin A1 (p = 9.94E-09), Epsin (p = 4.64E-08), ITSN 1-L (p = 4.96E-08), membrane mEGFP (p = 3.77E-06), Munc13 (p = 3.52E-06), Munc18 (p = 1.96E-12), PIP5KI-gamma (p = 1.63E-09), Rab3a (p = 2.54E-04), Rab5a (p = 2.42E-07), Rab7a (p = 2.24E-04), SCAMP1 (p = 4.64E-08), Septin 5 (p = 1.05E-08), SNAP23 (p = 7.99E-05), SNAP25 (p = 8.10E-13), SNAP29 (p = 8.59E-10), SV2B (p = 2.00E-09), Synapsin 1A (p = 7.77E-10), Synaptogyrin (p = 6.34E-10), Synaptophysin (p = 2.09E-13), Synaptotagmin 1 (p = 1.67E-12), Synaptotagmin 7 (p = 5.55E-11), Syndapin 1 (p = 2.70E-10), Syntaxin 1A (p = 3.71E-11), Syntaxin 16 (p = 2.52E-15), alpha-Tubulin 1b (p = 3.06E-09), VAMP1 (p = 3.48E-11), VAMP2 (p = 2.58E-06), VAMP4 (p = 7.08E-04), vATPase V0a1 (p = 3.66E-08), vGluT1 (p = 9.06E-14), Vti1a-beta (p = 8.41E-08).
Immobile fraction (synapse) is significantly different from immobile fraction (synapse) of: NSF (p = 5.86E-05), SCAMP1 (p = 2.16E-03), SV2B (p = 1.94E-11), Synaptogyrin (p = 3.29E-05), Synaptophysin (p = 2.69E-12), Synaptotagmin 1 (p = 7.13E-06), VAMP2 (p = 1.51E-04), VAMP4 (p = 1.73E-03), vATPase V0a1 (p = 9.46E-07), vGluT1 (p = 7.67E-09).

References

Zacharias D., et al. (2002). Science 296, 913–916.

membrane mEGFP

MW (kDa)	Category	τ_{axon} (s)	$\tau_{synapse}$ (s)	IF_{axon} (%)	$IF_{synapse}$ (%)
36.29	PM-associated, control	2.30 ± 0.29	4.84 ± 0.41	33.99 ± 3.89	26.86 ± 4.53

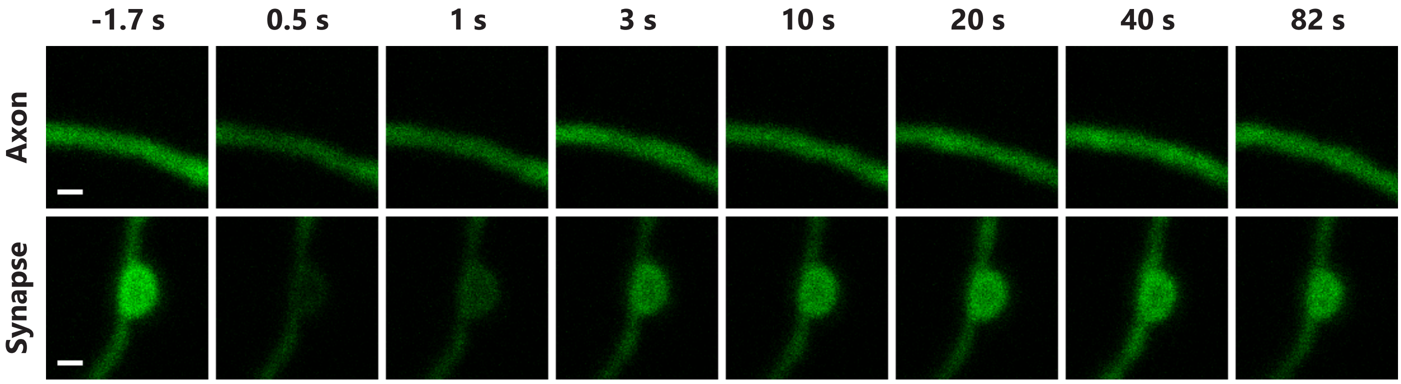

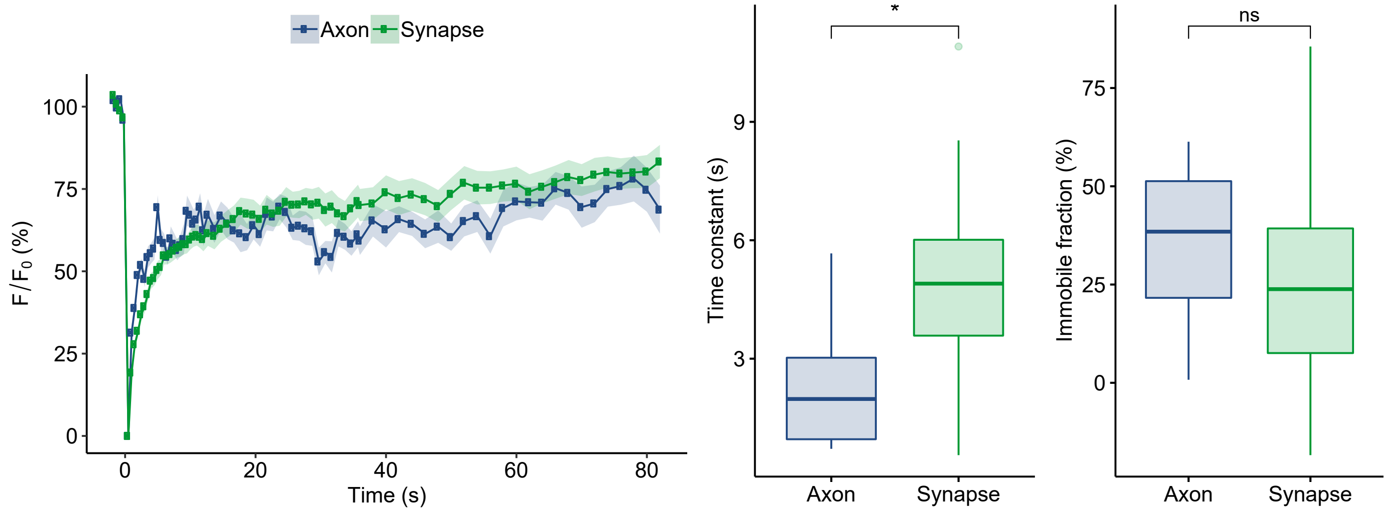

N (axons) = 22, N (synapses) = 29; p (time constant) = 5.31E-05.

Tagged protein outline:

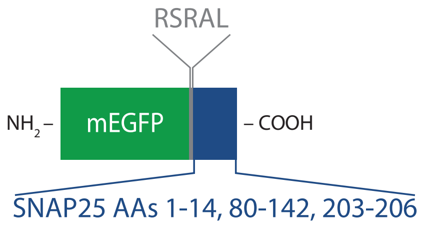

Time constant (axon) is significantly different from time constant (axon) of: Synaptotagmin 1 (p = 2.87E-02).
Immobile fraction (axon) is significantly different from immobile fraction (axon) of: VAMP2 (p = 4.07E-02).
Time constant (synapse) is significantly different from time constant (synapse) of: Dynamin 1 (p = 2.18E-02), mEGFP (p = 3.77E-06), SCAMP1 (p = 1.23E-03), SV2B (p = 4.51E-02), Synapsin 1A (p = 1.91E-04), Synaptogyrin (p = 1.90E-05), Synaptophysin (p = 1.79E-07), Synaptotagmin 1 (p = 3.92E-08), Syntaxin 16 (p = 2.19E-04), alpha-Tubulin 1b (p = 4.21E-03), VAMP1 (p = 6.56E-06), vGluT1 (p = 3.98E-07), Vti1a-beta (p = 2.09E-02).
Immobile fraction (synapse) is significantly different from immobile fraction (synapse) of: SV2B (p = 5.76E-06), Synaptogyrin (p = 4.54E-02), Synaptophysin (p = 8.57E-06), vGluT1 (p = 3.44E-03).

References

Munc13

MW (kDa)	Category	τ_{axon} (s)	$\tau_{synapse}$ (s)	IF_{axon} (%)	$IF_{synapse}$ (%)
19.64	soluble, SNARE co-factor	1.53 ± 0.12	3.95 ± 0.27	21.91 ± 2.41	4.64 ± 4.88

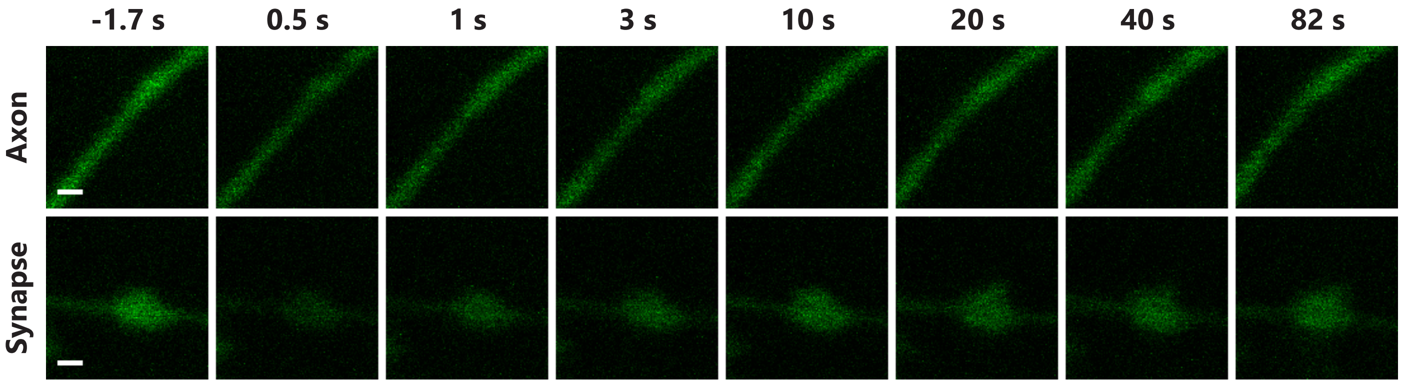

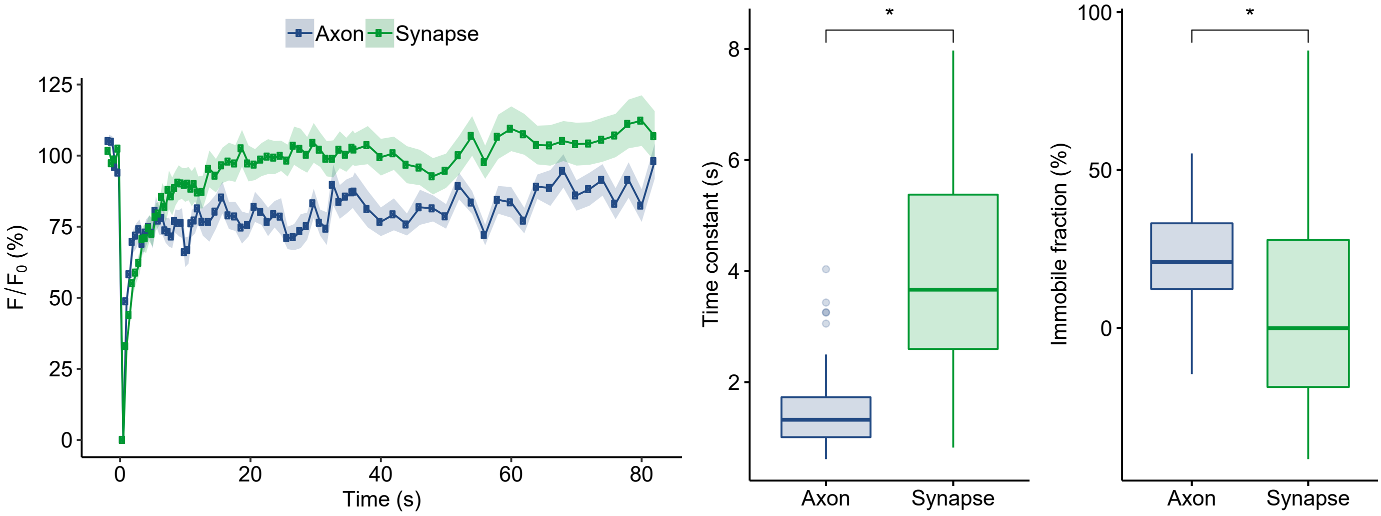

N (axons) = 47, N (synapses) = 45; p (time constant) = 9.18E-12, p (immobile fraction) = 1.10E-03.

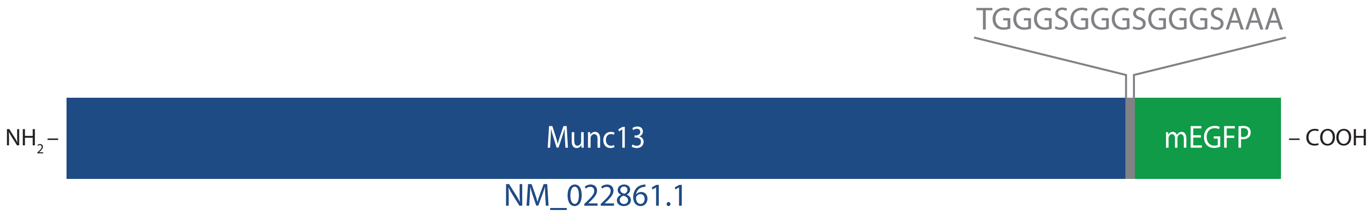

Time constant (axon) is significantly different from time constant (axon) of: Amphiphysin (p = 2.42E-04), Complexin 2 (p = 2.22E-03), Endophilin A1 (p = 2.19E-02), PIP5KI-gamma (p = 2.96E-02), Rab3a (p = 1.48E-03), SNAP23 (p = 5.20E-03), SNAP29 (p = 8.64E-06), Synapsin 1A (p = 2.47E-02), Synaptotagmin 1 (p = 8.17E-05), Synaptotagmin 7 (p = 8.65E-03), Syntaxin 1A (p = 2.36E-04), Syntaxin 16 (p = 4.07E-06), VAMP1 (p = 4.30E-05), VAMP2 (p = 4.64E-02).
Immobile fraction (axon) is not significantly different from immobile fraction (axon) of any other proteins.
Time constant (synapse) is significantly different from time constant (synapse) of: Amphiphysin (p = 1.07E-03), Dynamin 1 (p = 1.96E-04), Epsin (p = 1.32E-02), ITSN 1-L (p = 6.76E-04), mEGFP (p = 3.52E-06), PIP5KI-gamma (p = 1.92E-03), SCAMP1 (p = 1.20E-05), SV2B (p = 7.96E-04), Synapsin 1A (p = 9.74E-07), Synaptogyrin (p = 1.09E-07), Synaptophysin (p = 4.08E-10), Synaptotagmin 1 (p = 1.14E-10), Syntaxin 1A (p = 7.17E-03), Syntaxin 16 (p = 1.42E-08), alpha-Tubulin 1b (p = 1.50E-05), VAMP1 (p = 2.71E-08), vATPase V0a1 (p = 9.04E-04), vGluT1 (p = 3.44E-10), Vti1a-beta (p = 3.07E-04).
Immobile fraction (synapse) is significantly different from immobile fraction (synapse) of: , NSF (p = 1.05E-02), SCAMP1 (p = 2.46E-03), SV2B (p = 5.12E-09), Synaptogyrin (p = 8.53E-05), Synaptophysin (p = 6.77E-09), Synaptotagmin 1 (p = 1.72E-04), VAMP2 (p = 1.01E-02), VAMP4 (p = 1.02E-03), vATPase V0a1 (p = 4.95E-06), vGluT1 (p = 5.82E-07).

References

Deng, L., et al. (2011). Neuron 69, 317-31.
Betz, A., et al. (1997). J Biol Chem 272, 2520-6.
Varoqueaux, F., et al. (2002). Proc Natl Acad Sci U S A 99, 9037-42.

Munc18

MW (kDa)	Category	τ_{axon} (s)	$\tau_{synapse}$ (s)	IF_{axon} (%)	$IF_{synapse}$ (%)
67.57	soluble, SNARE co-factor	2.37 ± 0.33	4.66 ± 0.25	21.50 ± 4.41	18.24 ± 1.55

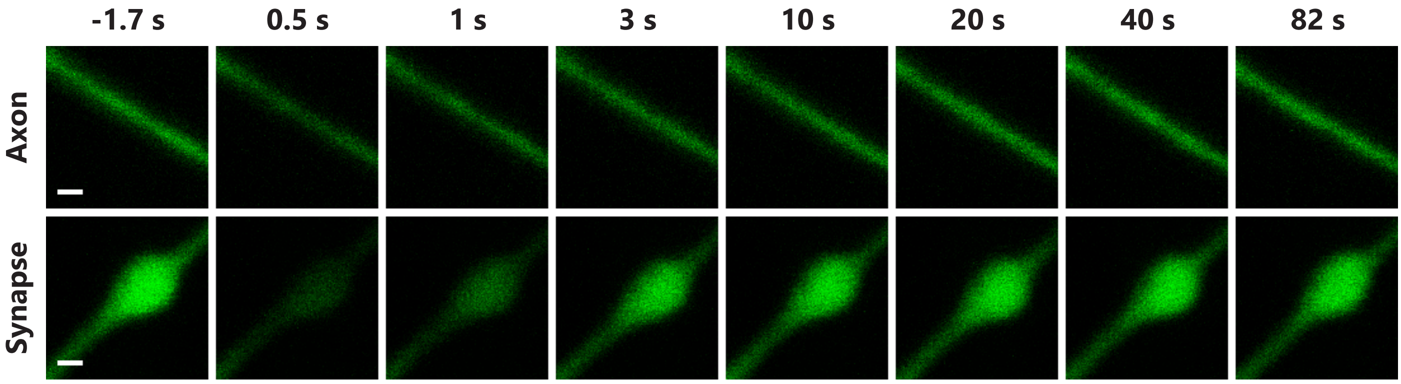

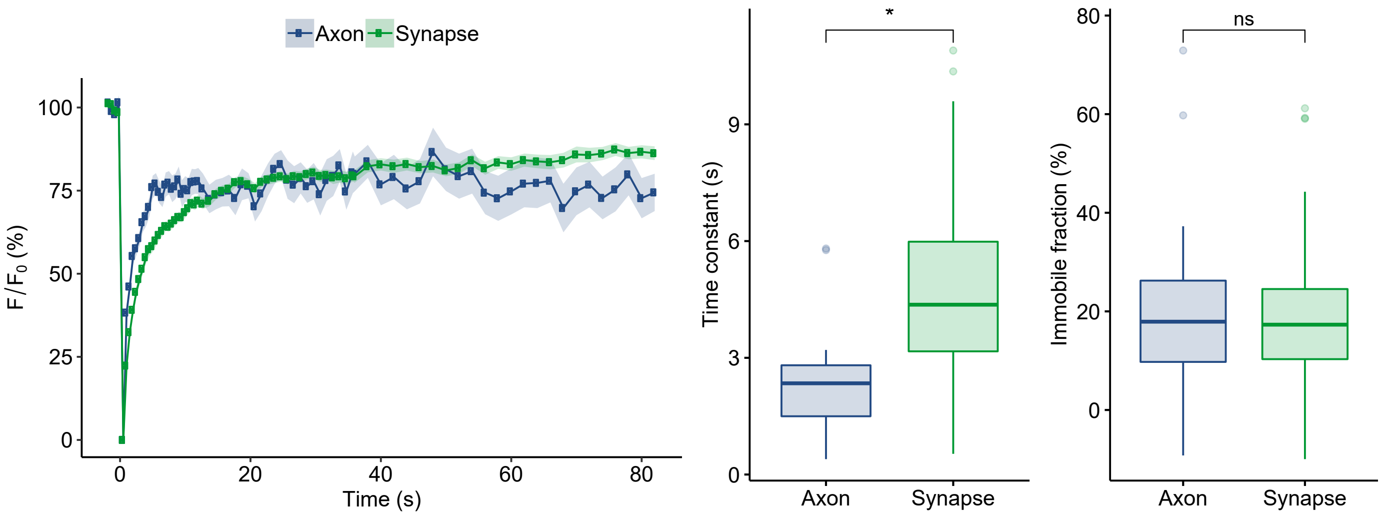

N (axons) = 19, N (synapses) = 78; p (time constant) = 1.02E-05.

Tagged protein outline:

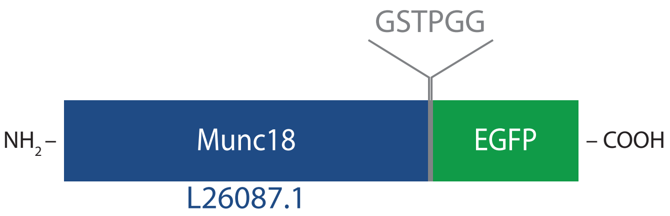

Time constant (axon) is not significantly different from time constant (axon) of any other proteins.
Immobile fraction (axon) is not significantly different from immobile fraction (axon) of any other proteins.
Time constant (synapse) is significantly different from time constant (synapse) of: , Dynamin 1 (p = 3.53E-04), Hsc70 (p = 1.49E-02), ITSN 1-L (p = 1.13E-03), mEGFP (p = 1.96E-12), PIP5KI-gamma (p = 4.29E-02), SCAMP1 (p = 1.29E-05), SV2B (p = 3.27E-04), Synapsin 1A (p = 1.88E-07), Synaptogyrin (p = 1.01E-08), Synaptophysin (p = 1.66E-12), Synaptotagmin 1 (p = 4.96E-13), Syntaxin 16 (p = 7.39E-09), alpha-Tubulin 1b (p = 1.70E-05), VAMP1 (p = 2.01E-09), vATPase V0a1 (p = 5.85E-04), vGluT1 (p = 4.59E-12), Vti1a-beta (p = 1.22E-04).
Immobile fraction (synapse) is significantly different from immobile fraction (synapse) of: , NSF (p = 2.30E-05), SCAMP1 (p = 2.58E-03), SV2B (p = 3.07E-12), Synaptogyrin (p = 2.52E-05), Synaptophysin (p = 1.92E-13), Synaptotagmin 1 (p = 2.69E-06), VAMP2 (p = 7.20E-05), VAMP4 (p = 9.24E-04), vATPase V0a1 (p = 1.89E-07), vGluT1 (p = 2.15E-09).

References

Burgoyne, R.D., et al. (2009). Ann N Y Acad Sci 1152, 76-86.
Takamori, S., et al. (2006). Cell 127, 831-46.
Verhage, M., et al. (2000). Science 287, 864-69.

NSF

MW (kDa)	Category	τ_{axon} (s)	$\tau_{synapse}$ (s)	IF_{axon} (%)	$IF_{synapse}$ (%)
82.65	soluble, SNARE co-factor	1.69 ± 0.24	3.40 ± 0.34	27.68 ± 5.49	42.49 ± 4.00

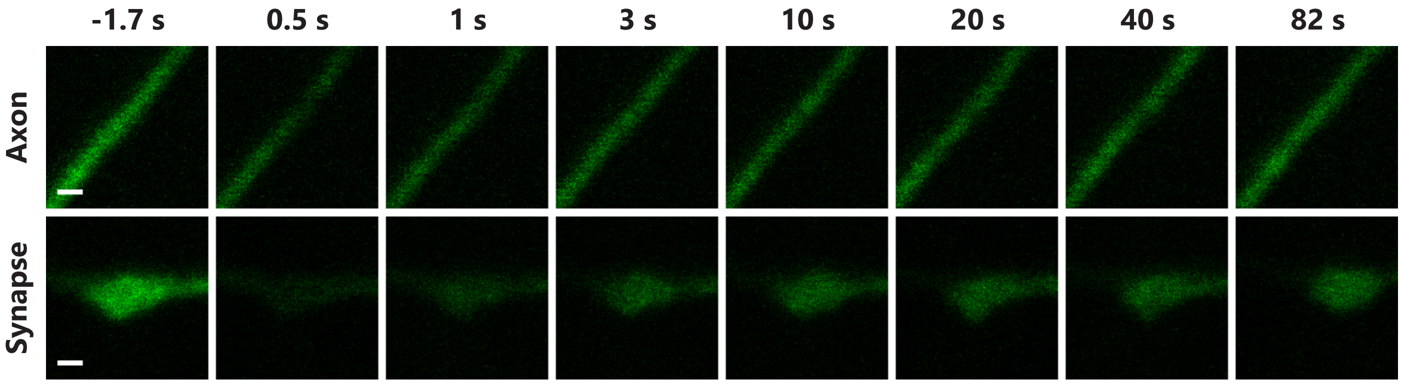

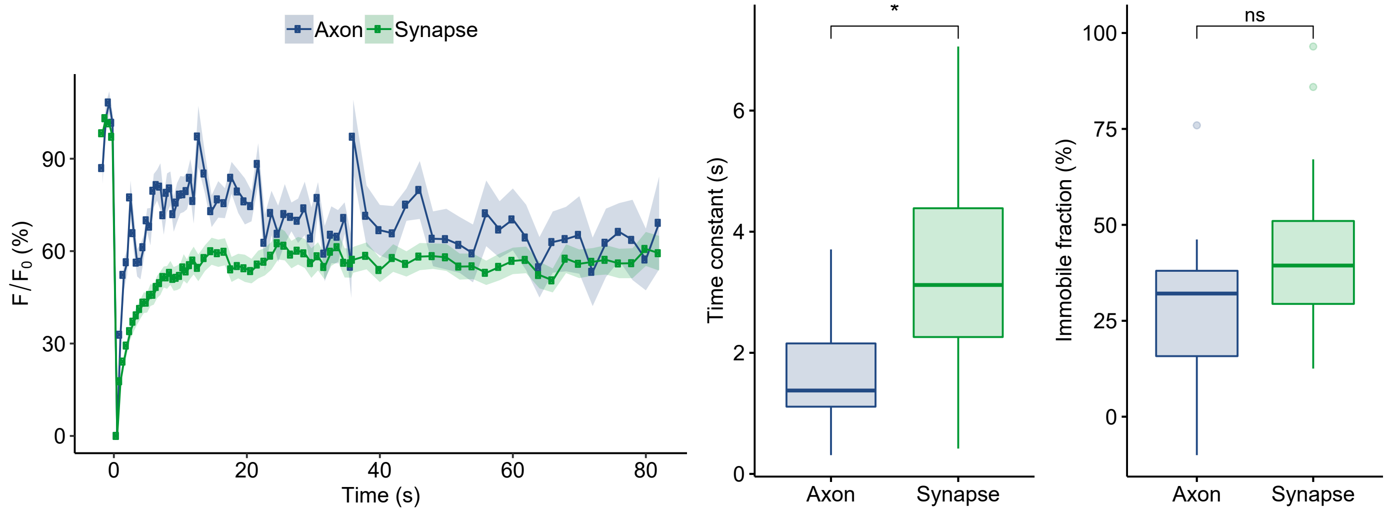

N (axons) = 15, N (synapses) = 24; p (time constant) = 5.04E-04.

Tagged protein outline:

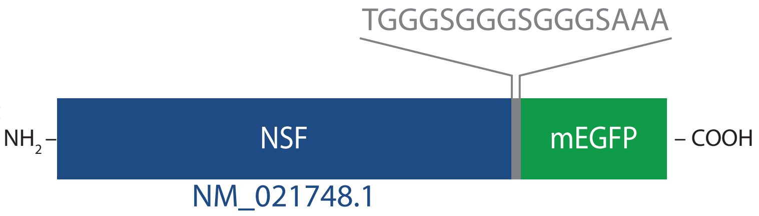

Time constant (axon) is significantly different from time constant (axon) of: Synaptotagmin 1 (p = 2.19E-02).
Immobile fraction (axon) is not significantly different from immobile fraction (axon) of any other proteins.
Time constant (synapse) is significantly different from time constant (synapse) of: Amphiphysin (p = 1.15E-03), Dynamin 1 (p = 1.01E-03), Epsin (p = 1.57E-02), ITSN 1-L (p = 6.07E-03), PIP5KI-gamma (p = 1.63E-03), SCAMP1 (p = 1.15E-04), SV2B (p = 5.79E-03), Synapsin 1A (p = 3.83E-05), Synaptogyrin (p = 1.16E-05), Synaptophysin (p = 4.40E-07), Synaptotagmin 1 (p = 2.39E-07), Synaptotagmin 7 (p = 2.43E-02), Syntaxin 1A (p = 2.52E-03), Syntaxin 16 (p = 2.63E-06), alpha-Tubulin 1b (p = 3.19E-04), VAMP1 (p = 4.45E-06), vATPase V0a1 (p = 1.49E-02), vGluT1 (p = 4.12E-07), Vti1a-beta (p = 3.58E-03).
Immobile fraction (synapse) is significantly different from immobile fraction (synapse) of: alpha-SNAP (p = 1.23E-03), AP180 (p = 1.21E-04), Complexin 2 (p = 5.14E-03), mEGFP (p = 5.86E-05), Munc13 (p = 1.05E-02), Munc18 (p = 2.30E-05), Rab3a (p = 1.62E-02), Rab5a (p = 1.44E-06), Rab7a (p = 1.00E-03), Munc13 (p = 1.05E-02), SNAP29 (p = 1.46E-03), SV2B (p = 5.79E-03), Synaptophysin (p = 6.85E-03), Synaptotagmin 7 (p = 1.27E-02), Syndapin 1 (p = 1.82E-02), Syntaxin 1A (p = 3.28E-02).

References

Söllner, T., et al. (1997a). Cell 75, 409-18.
Jahn, R., and Scheller, R.H. (2006) Nat Rev Mol Cell Biol 7, 631-43.
Takamori, S., et al. (2006). Cell 127, 831-46.

PIP5KIγ

MW (kDa)	Category	τ_{axon} (s)	$\tau_{synapse}$ (s)	IF_{axon} (%)	$IF_{synapse}$ (%)
75.60	soluble, endocytic	3.77 ± 0.64	7.16 ± 0.56	32.76 ± 5.13	16.15 ± 5.98

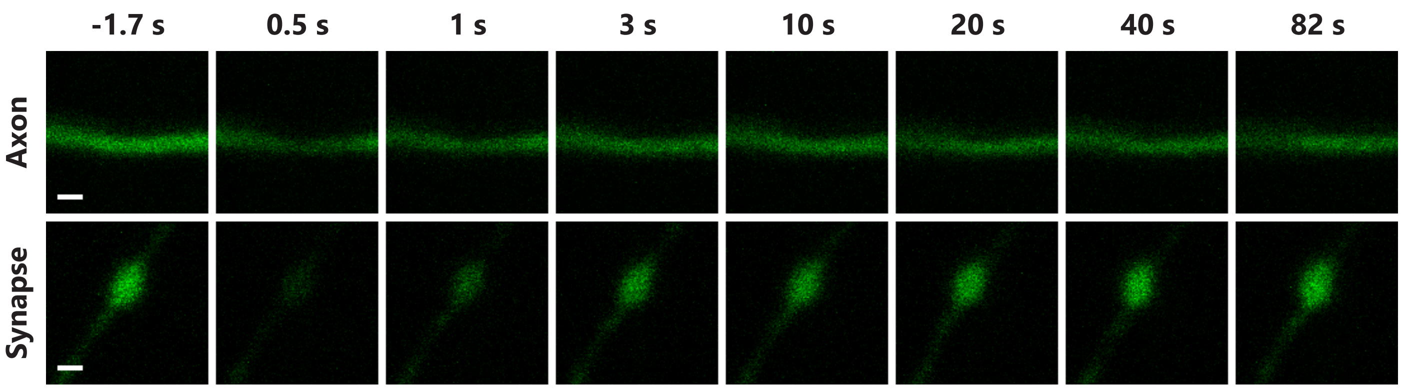

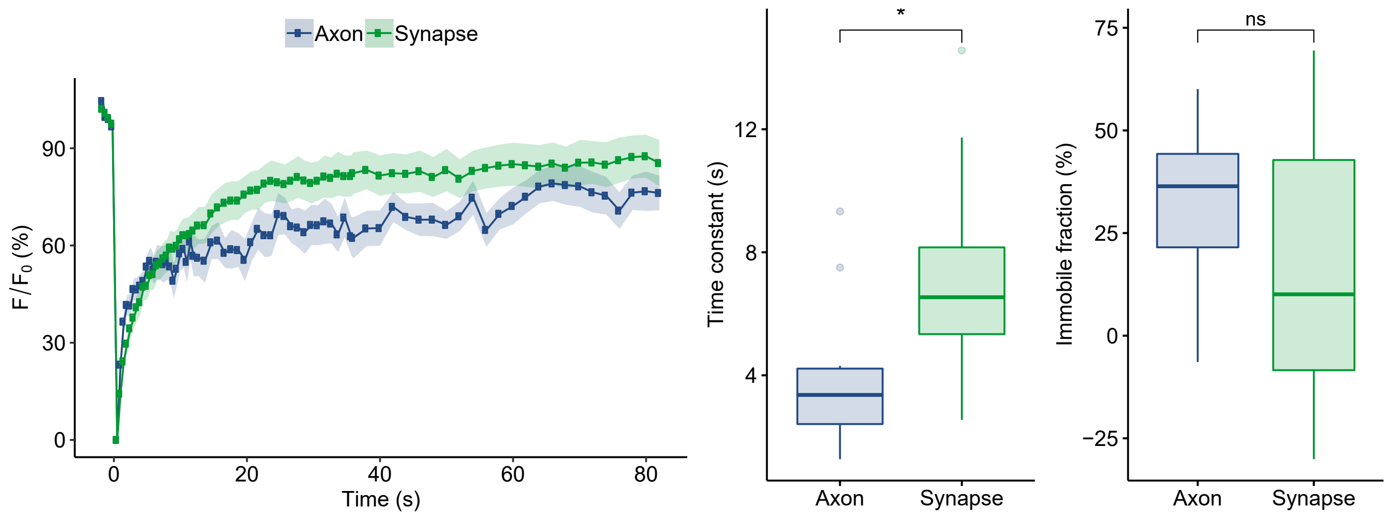

N (axons) = 13, N (synapses) = 24; p (time constant) = 3.45E-04.

Tagged protein outline:

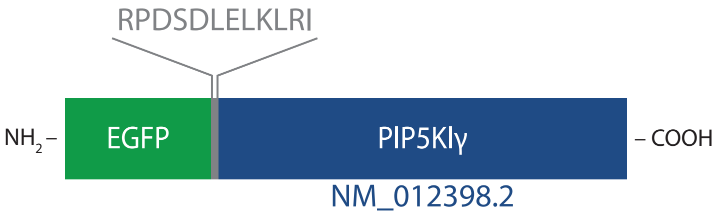

Time constant (axon) is significantly different from time constant (axon) of: Actin (p = 2.75E-02), Munc13 (p = 2.96E-02).
Immobile fraction (axon) is not significantly different from immobile fraction (axon) of any other proteins.
Time constant (synapse) is significantly different from time constant (synapse) of: AP2 (p = 1.77E-04), Calmodulin 1 (p = 2.80E-05), Clathrin light chain B (p = 4.91E-05), Complexin 1 (p = 7.51E-03), Endophilin A1 (p = 2.37E-02), Hsc70 (p = 7.79E-06), mEGFP (p = 1.63E-09), Munc13 (p = 1.92E-03), Munc18 (p = 4.29E-02), NSF (p = 1.63E-03), Rab3a (p = 1.27E-02), Rab5a (p = 4.09E-04), Rab7a (p = 1.57E-02), SNAP23 (p = 5.10E-04), SNAP25 (p = 1.57E-03), Synapsin 1A (p = 4.70E-02), Synaptogyrin (p = 6.32E-04), Synaptophysin (p = 2.97E-05), Synaptotagmin 1 (p = 5.34E-06), VAMP1 (p = 4.31E-03), vGluT1 (p = 4.85E-04).
Immobile fraction (synapse) is significantly different from immobile fraction (synapse) of: SV2B (p = 9.59E-06), Synaptogyrin (p = 1.18E-02), Synaptophysin (p = 6.05E-06), vATPase V0a1 (p = 9.68E-03), vGluT1 (p = 1.28E-03).

References

Giudici, M.L., et al. (2004). Biochem J 379, 489-96.
Krauss, M., et al. (2003). J Cell Biol 162, 113-24.

Rab3a

MW (kDa)	Category	τ_{axon} (s)	$\tau_{synapse}$ (s)	IF_{axon} (%)	$IF_{synapse}$ (%)
24.97	vesicle-associated, SNARE co-factor	3.24 ± 0.33	4.00 ± 0.32	8.31 ± 3.79	14.31 ± 3.58

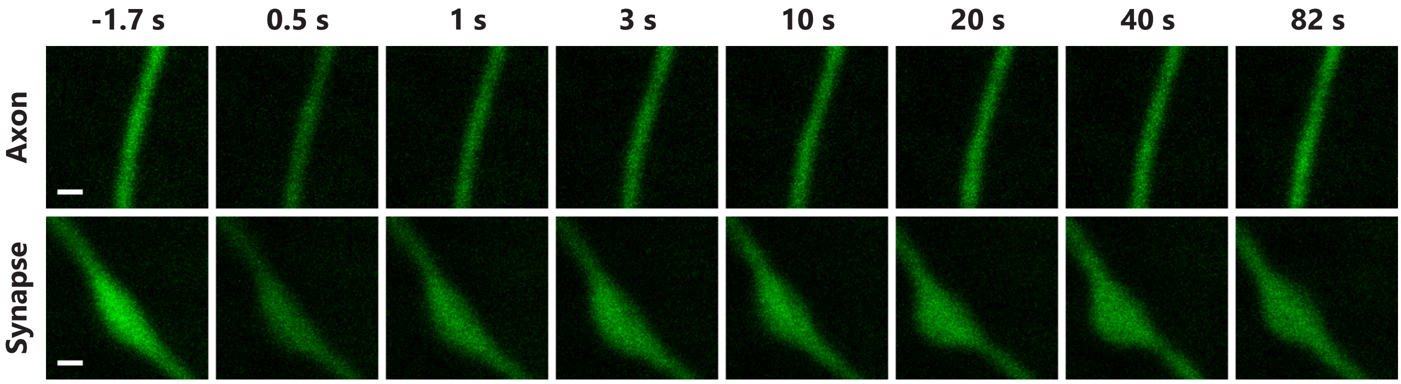

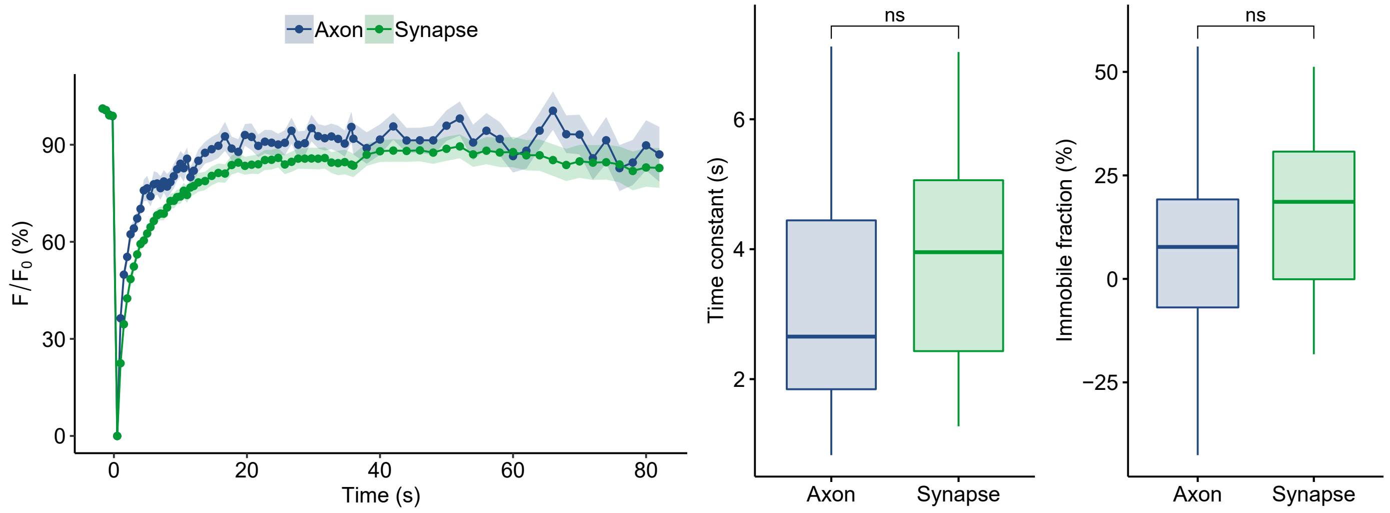

N (axons) = 29, N (synapses) = 29.

Tagged protein outline:

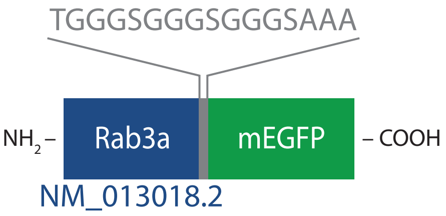

Time constant (axon) is significantly different from time constant (axon) of: Actin (p = 3.28E-03), Munc13 (p = 1.48E-03).
Immobile fraction (axon) is not significantly different from immobile fraction (axon) of any other proteins.
Time constant (synapse) is significantly different from time constant (synapse) of: Amphiphysin (p = 3.21E-02), Dynamin 1 (p = 1.31E-03), ITSN 1-L (p = 9.89E-03), mEGFP (p = 2.54E-04), PIP5KI-gamma (p = 1.27E-02), SCAMP1 (p = 6.65E-05), SV2B (p = 6.95E-03), Synapsin 1A (p = 3.19E-05), Synaptogyrin (p = 5.36E-06), Synaptophysin (p = 6.84E-08), Synaptotagmin 1 (p = 2.40E-08), Syntaxin 1A (p = 2.49E-02), Syntaxin 16 (p = 5.62E-06), alpha-Tubulin 1b (p = 3.45E-04), VAMP1 (p = 1.02E-06), vATPase V0a1 (p = 1.62E-02), vGluT1 (p = 7.59E-08), Vti1a-beta (p = 3.67E-03).
Immobile fraction (synapse) is significantly different from immobile fraction (synapse) of: NSF (p = 1.62E-02), SCAMP1 (p = 1.16E-02), SV2B (p = 4.42E-07), Synaptogyrin (p = 1.62E-03), Synaptophysin (p = 8.15E-08), Synaptotagmin 1 (p = 1.21E-03), VAMP2 (p = 2.18E-02), VAMP4 (p = 5.05E-03), vATPase V0a1 (p = 2.83E-04), vGluT1 (p = 9.77E-06).

References

Schlüter, O.M., et al. (2004). J Biol Chem 277, 40919-29.
Fische von Mollard, G., et al. (1991), Nature 349, 79-81.
Takamori, S., et al. (2006). Cell 127, 831-46.

Rab5a

MW (kDa)	Category	τ_{axon} (s)	$\tau_{synapse}$ (s)	IF_{axon} (%)	$IF_{synapse}$ (%)
23.62	endosome-associated	1.54 ± 0.13	3.82 ± 0.23	21.48 ± 3.46	8.23 ± 2.31

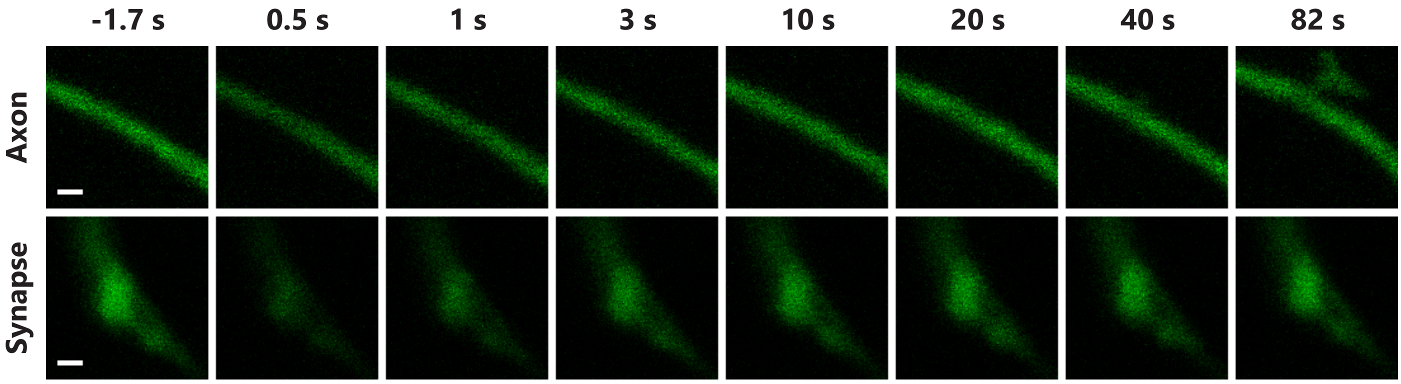

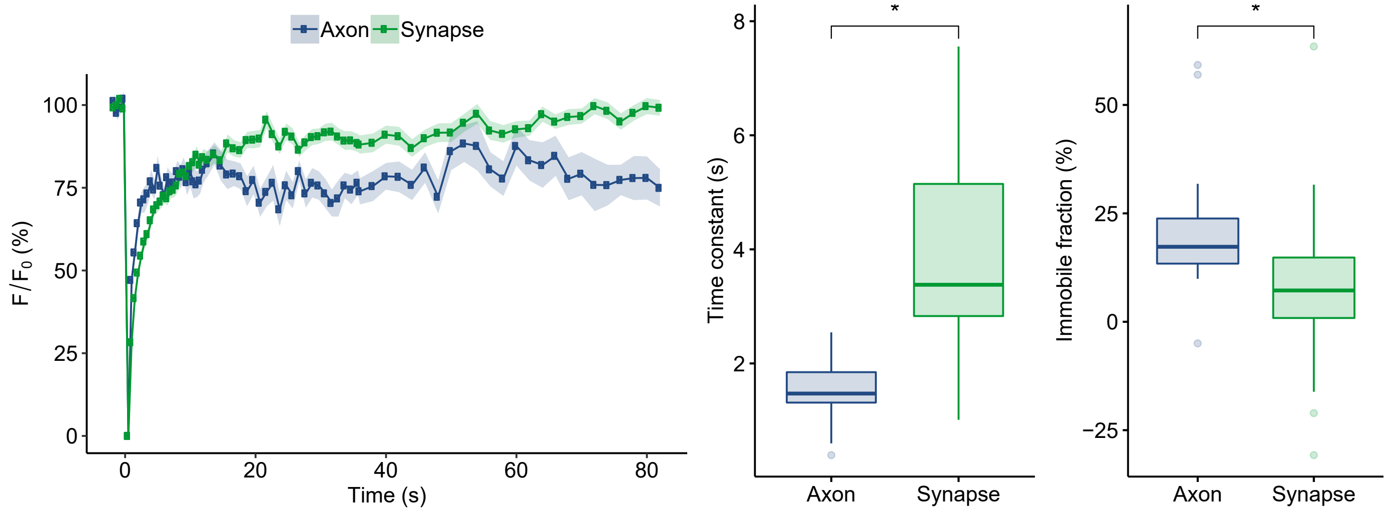

N (axons) = 19, N (synapses) = 45; p (time constant) = 3.58E-08, p (immobile fraction) = 6.52E-04.

Tagged protein outline:

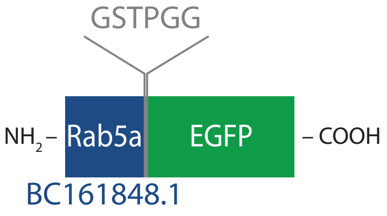

Time constant (axon) is significantly different from time constant (axon) of: Amphiphysin (p = 1.32E-02), SNAP29 (p = 8.11E-04), Synaptotagmin 1 (p = 5.57E-03), Syntaxin 1A (p = 1.15E-03), Syntaxin 16 (p = 3.71E-03), VAMP1 (p = 2.70E-03).
Immobile fraction (axon) is not significantly different from immobile fraction (axon) of any other proteins.
Time constant (synapse) is significantly different from time constant (synapse) of: Amphiphysin (p = 3.19E-04), Dynamin 1 (p = 7.14E-05), Epsin (p = 5.39E-03), ITSN 1-L (p = 3.17E-04), mEGFP (p = 2.42E-07), PIP5KI-gamma (p = 4.09E-04), SCAMP1 (p = 3.21E-06), SV2B (p = 7.56E-04), Synapsin 1A (p = 8.31E-07), Synaptogyrin (p = 1.28E-07), Synaptophysin (p = 3.31E-10), Synaptotagmin 1 (p = 1.14E-10), Synaptotagmin 7 (p = 3.81E-02), Syntaxin 1A (p = 6.81E-04), Syntaxin 16 (p = 4.98E-09), alpha-Tubulin 1b (p = 6.29E-06), VAMP1 (p = 1.15E-08), vATPase V0a1 (p = 7.59E-04), vGluT1 (p = 2.45E-10), Vti1a-beta (p = 1.80E-04).
Immobile fraction (synapse) is significantly different from immobile fraction (synapse) of: Hsc70 (p = 4.95E-02), ITSN 1-L (p = 3.67E-02), NSF (p = 1.44E-06), SCAMP1 (p = 1.27E-03), SNAP23 (p = 2.47E-02), SV2B (p = 1.73E-10), Synaptogyrin (p = 1.64E-05), Synaptophysin (p = 5.12E-11), Synaptotagmin 1 (p = 1.58E-06), VAMP1 (p = 2.04E-02), VAMP2 (p = 9.24E-06), VAMP4 (p = 7.42E-05), vATPase V0a1 (p = 3.70E-07), vGluT1 (p = 2.10E-08), Vti1a-beta (p = 2.58E-02).

References

Stenmark, H., et al. (1994). EMBO J 13, 1287-96.
Stenmark, H., et al. (1995). Cell 83, 423-32.
Takamori, S., et al. (2006). Cell 127, 831-46.

Rab7a

MW (kDa)	Category	τ_{axon} (s)	$\tau_{synapse}$ (s)	IF_{axon} (%)	$IF_{synapse}$ (%)
23.50	endosome-associated	1.74 ± 0.18	3.91 ± 0.34	24.83 ± 3.71	3.26 ± 4.68

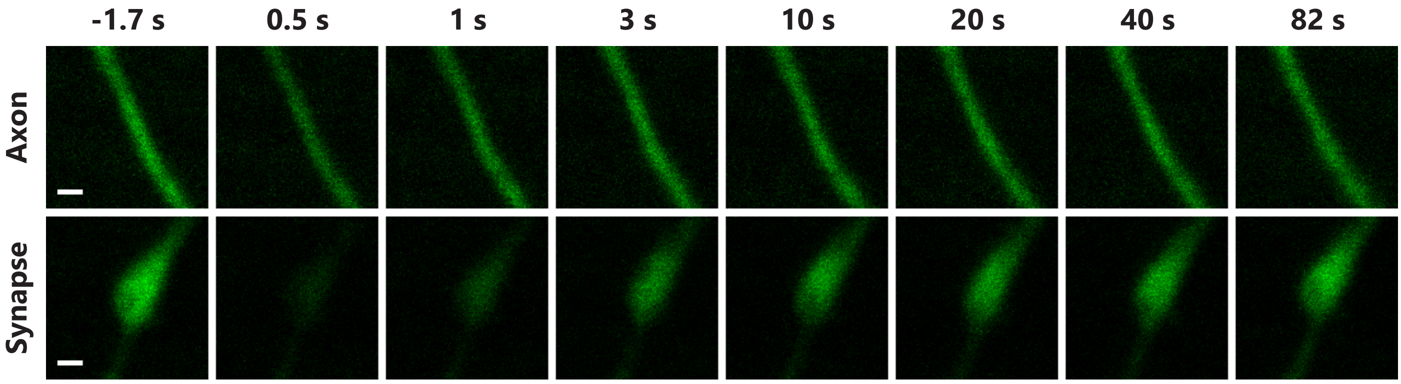

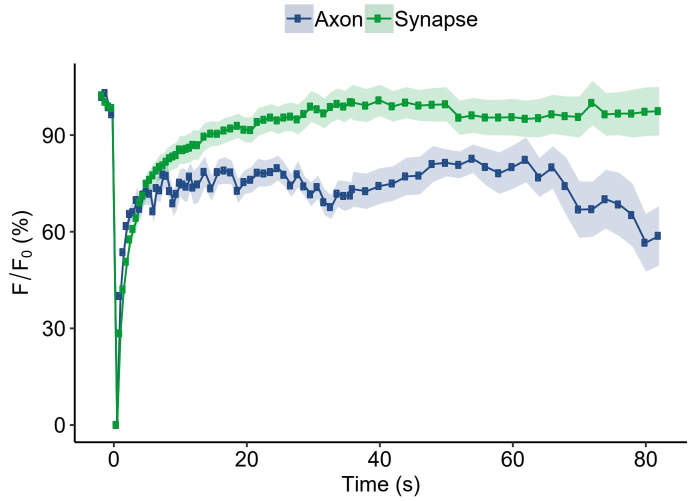

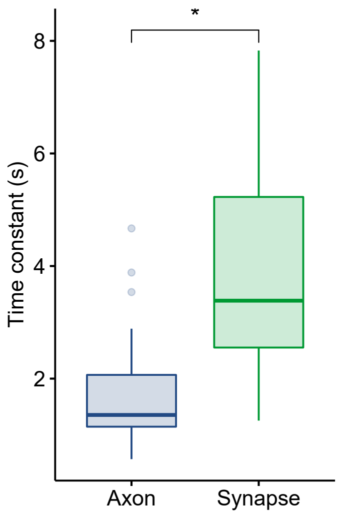

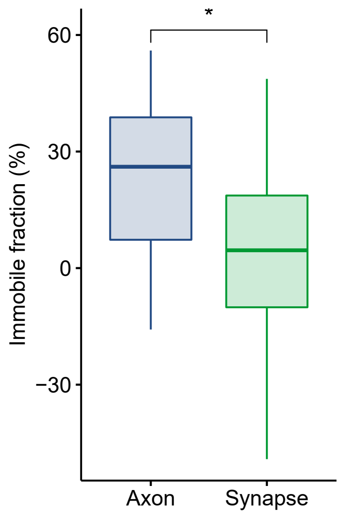

N (axons) = 29, N (synapses) = 28; p (time constant) = 4.37E-07, p (immobile fraction) = 1.71E-03.

Tagged protein outline:

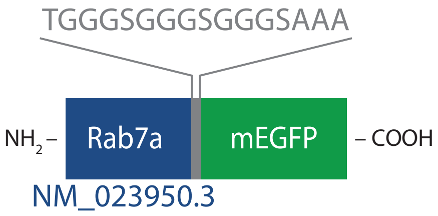

Time constant (axon) is significantly different from time constant (axon) of: Amphiphysin (p = 2.79E-02), SNAP29 (p = 1.56E-03), Synaptotagmin 1 (p = 1.74E-03), Syntaxin 1A (p = 7.74E-03), Syntaxin 16 (p = 5.21E-03), VAMP1 (p = 6.19E-03).
Immobile fraction (axon) is not significantly different from immobile fraction (axon) of any other proteins.
Time constant (synapse) is significantly different from time constant (synapse) of: Amphiphysin (p = 1.60E-02), Dynamin 1 (p = 2.07E-03), ITSN 1-L (p = 9.36E-03), mEGFP (p = 2.24E-04), PIP5KI-gamma (p = 1.57E-02), SCAMP1 (p = 1.44E-04), SV2B (p = 6.85E-03), Synapsin 1A (p = 2.26E-05), Synaptogyrin (p = 5.55E-06), Synaptophysin (p = 1.04E-07), Synaptotagmin 1 (p = 3.69E-08), Syntaxin 1A (p = 2.34E-02), Syntaxin 16 (p = 3.01E-06), alpha-Tubulin 1b (p = 2.58E-04), VAMP1 (p = 1.40E-06), vATPase V0a1 (p = 1.53E-02), vGluT1 (p = 1.30E-07), Vti1a-beta (p = 3.61E-03).
Immobile fraction (synapse) is significantly different from immobile fraction (synapse) of: NSF (p = 1.00E-03), SCAMP1 (p = 2.97E-03), SV2B (p = 1.56E-07), Synaptogyrin (p = 2.16E-04), Synaptophysin (p = 5.06E-08), Synaptotagmin 1 (p = 1.65E-04), VAMP2 (p = 1.33E-03), VAMP4 (p = 1.21E-03), vATPase V0a1 (p = 1.75E-05), vGluT1 (p = 4.09E-06).

References

Takamori, S., et al. (2006). Cell 127, 831-86.
Bucci, C., et al. (2000). Mol Biol Cell 11, 467-80.

SCAMP1

MW (kDa)	Category	τ_{axon} (s)	$\tau_{synapse}$ (s)	IF_{axon} (%)	$IF_{synapse}$ (%)
37.99	plasma membrane	-	12.57 ± 1.77	-	57.63 ± 6.92

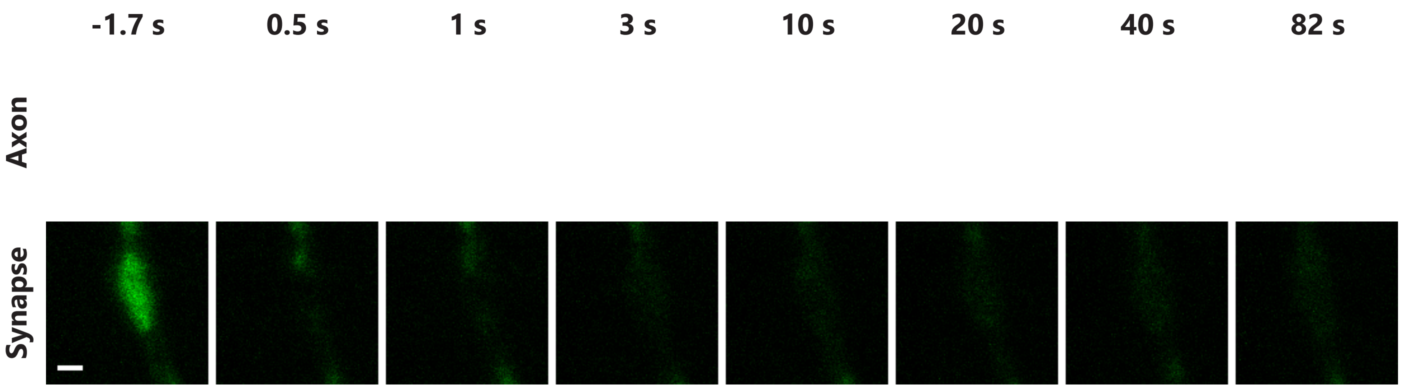

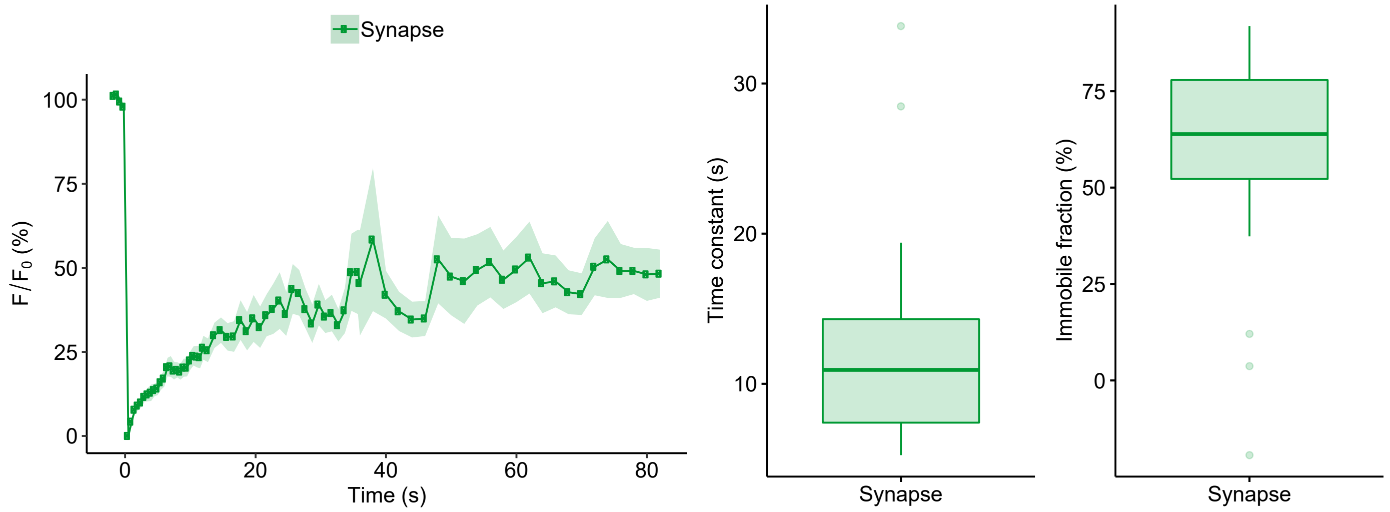

N (axons) = 0, N (synapses) = 19.

Tagged protein outline:

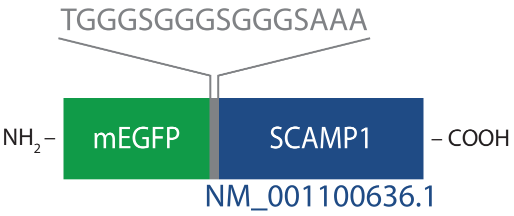

Time constant (synapse) is significantly different from time constant (synapse) of: alpha-SNAP (p = 1.16E-02), AP180 (p = 1.91E-04), AP2 (p = 2.62E-05), Calmodulin 1 (p = 1.59E-05), Clathrin light chain B (p = 1.33E-05), Complexin 1 (p = 1.25E-04), Complexin 2 (p = 2.16E-03), Endophilin A1 (p = 1.28E-04), Hsc70 (p = 1.51E-06), mEGFP (p = 4.64E-08), membrane mEGFP (p = 1.23E-03), Munc13 (p = 1.20E-05), Munc18 (p = 1.29E-05), NSF (p = 1.15E-04), Rab3a (p = 6.65E-05), Rab5a (p = 3.21E-06), Rab7a (p = 1.44E-04), Septin 5 (p = 1.91E-04), SNAP23 (p = 4.43E-05), SNAP25 (p = 3.72E-06), SNAP29 (p = 4.07E-03), Synaptotagmin 7 (p = 1.71E-02), Syndapin 1 (p = 4.52E-03), VAMP2 (p = 3.73E-02), VAMP4 (p = 7.09E-03).
Immobile fraction (synapse) is significantly different from immobile fraction (synapse) of: , alpha-SNAP (p = 8.31E-03), AP180 (p = 3.25E-03), AP2 (p = 2.74E-02), Complexin 2 (p = 3.02E-02), Doc2a (p = 4.35E-02), Endophilin A1 (p = 2.49E-02), mEGFP (p = 2.16E-03), Munc13 (p = 2.46E-03), Munc18 (p = 2.58E-03), Rab3a (p = 1.16E-02), Rab5a (p = 1.27E-03), Rab7a (p = 2.97E-03), Munc13 (p = 2.46E-03), SNAP29 (p = 6.83E-03), Synaptotagmin 7 (p = 7.84E-03), Syndapin 1 (p = 1.90E-02), Syntaxin 1A (p = 1.38E-02), Syntaxin 16 (p = 1.97E-02).

References

Castle, A., and Castle, D. (2005). J Cell Sci 118, 3769-80.
Takamori, S., et al. (2006). Cell 127, 831-846.

Septin 5

MW (kDa)	Category	τ_{axon} (s)	$\tau_{synapse}$ (s)	IF_{axon} (%)	$IF_{synapse}$ (%)
42.85	cytoskeletal	2.72 ± 0.35	4.40 ± 0.33	21.18 ± 4.78	21.23 ± 4.87

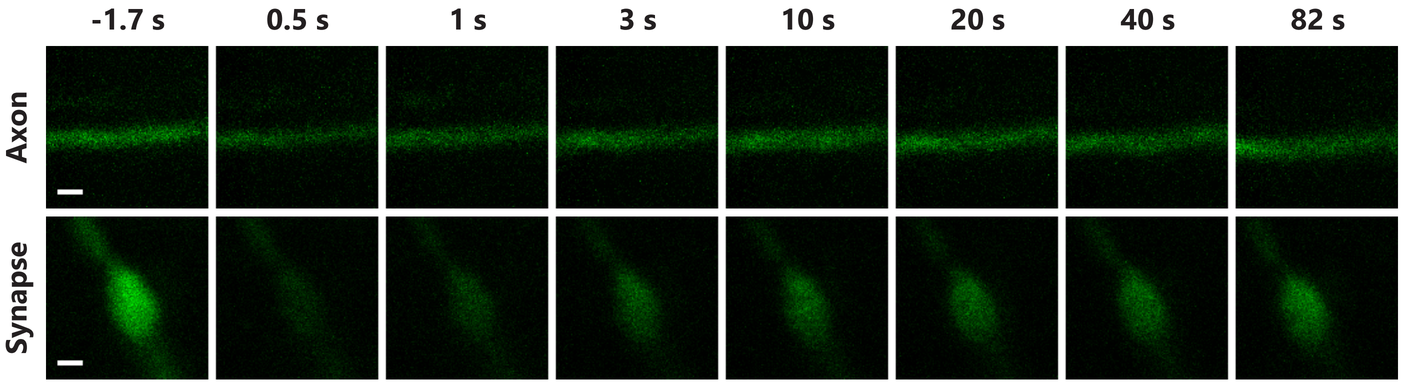

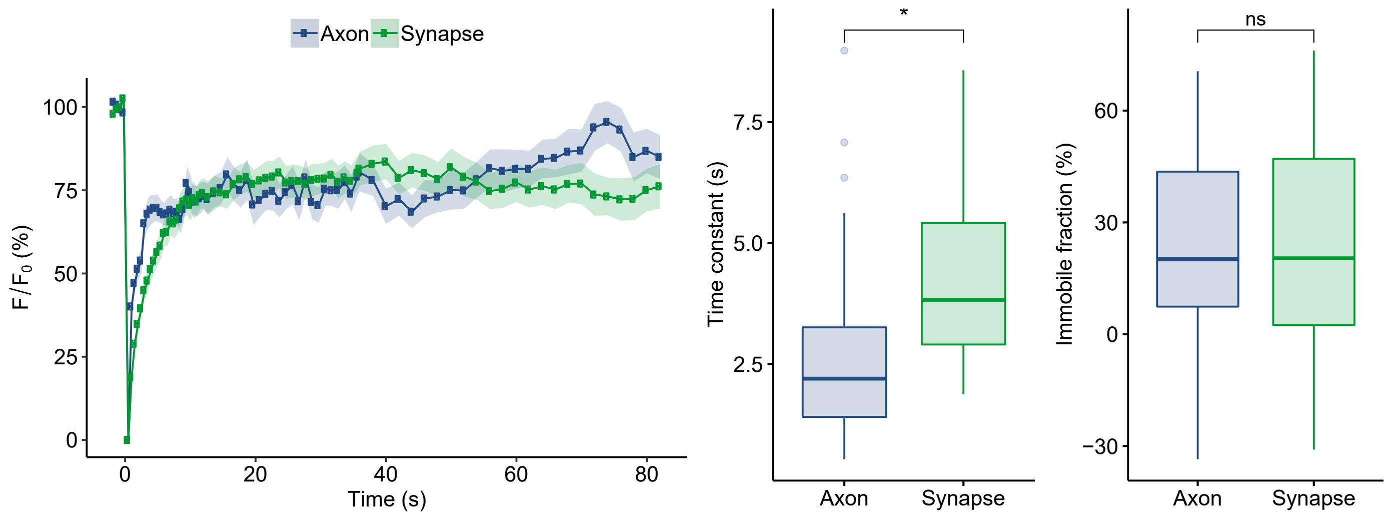

N (axons) = 32, N (synapses) = 37; p (time constant) = 8.98E-05.

Tagged protein outline:

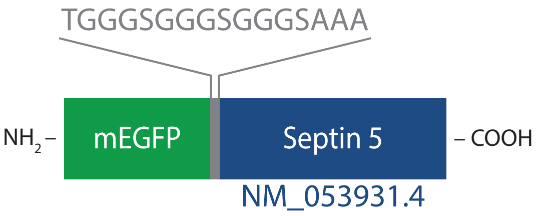

Time constant (axon) is not significantly different from time constant (axon) of any other proteins.
Immobile fraction (axon) is not significantly different from immobile fraction (axon) of any other proteins.
Time constant (synapse) is significantly different from time constant (synapse) of: Amphiphysin (p = 2.64E-02), Dynamin 1 (p = 4.22E-03), ITSN 1-L (p = 6.14E-03), mEGFP (p = 1.05E-08), SCAMP1 (p = 1.91E-04), SV2B (p = 9.04E-03), Synapsin 1A (p = 4.86E-06), Synaptogyrin (p = 6.51E-07), Synaptophysin (p = 8.36E-09), Synaptotagmin 1 (p = 1.72E-09), Syntaxin 16 (p = 8.21E-07), alpha-Tubulin 1b (p = 3.37E-04), VAMP1 (p = 2.55E-07), vATPase V0a1 (p = 1.99E-02), vGluT1 (p = 2.10E-08), Vti1a-beta (p = 1.93E-03).
Immobile fraction (synapse) is significantly different from immobile fraction (synapse) of: SV2B (p = 1.66E-07), Synaptogyrin (p = 4.69E-03), Synaptophysin (p = 1.54E-07), Synaptotagmin 1 (p = 4.16E-02), vATPase V0a1 (p = 7.34E-03), vGluT1 (p = 2.36E-04).

References

Mostowy, S., and Cossart, P. (2012). Nat Rev Mol Cell Biol 13, 183-94.
Beites, C.L., et al. (1999). Nat Neurosci 2, 434-9.
Amin, N.D., et al. (2008). J Neurosci 28, 3631-43.

SNAP23

MW (kDa)	Category	τ_{axon} (s)	$\tau_{synapse}$ (s)	IF_{axon} (%)	$IF_{synapse}$ (%)
23.23	plasma membrane-associated	2.79 ± 0.22	2.54 ± 0.23	16.58 ± 4.48	25.56 ± 3.09

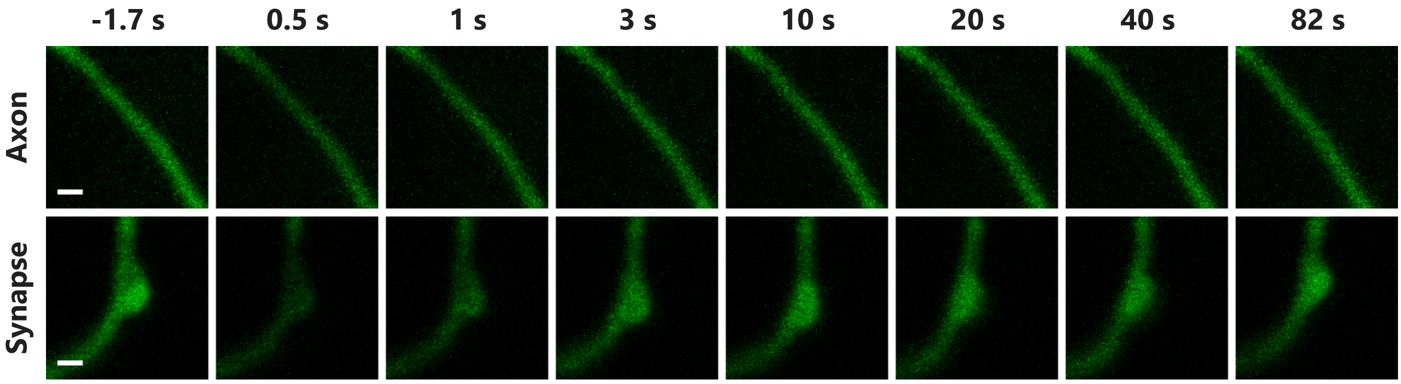

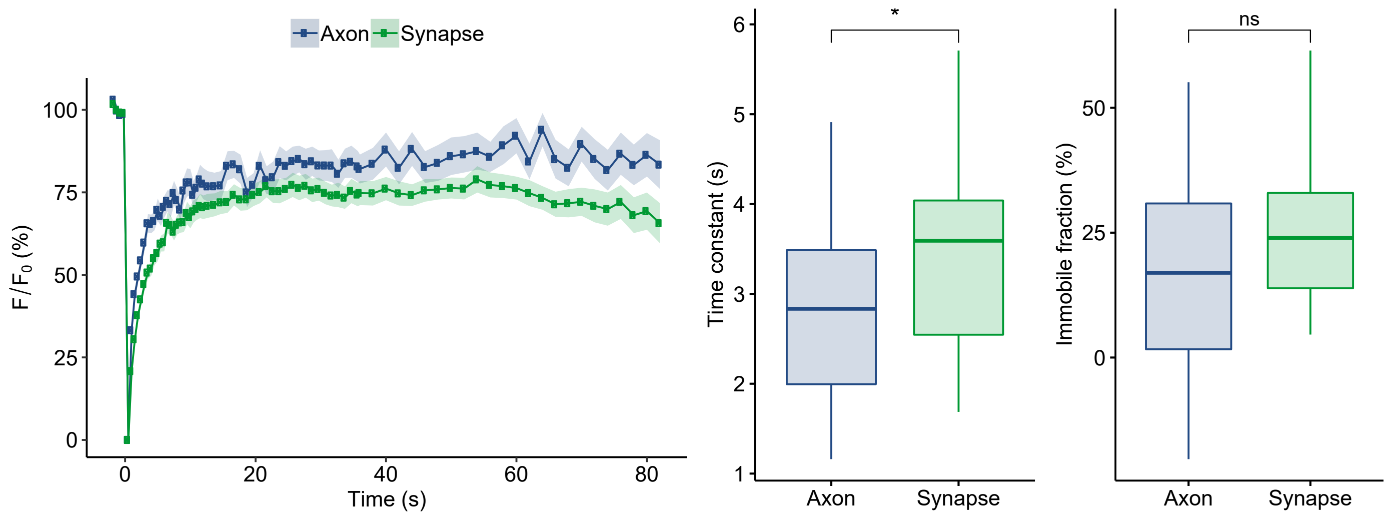

N (axons) = 22, N (synapses) = 24; p (time constant) = 2.56E-02.

Tagged protein outline:

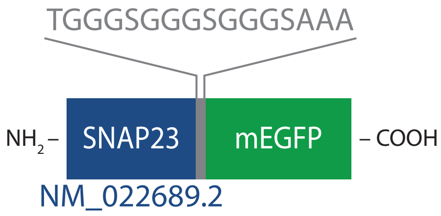

Time constant (axon) is significantly different from time constant (axon) of: Actin (p = 6.86E-03), Munc13 (p = 5.20E-03), Synaptotagmin 1 (p = 2.87E-02).
Immobile fraction (axon) is not significantly different from immobile fraction (axon) of any other proteins.
Time constant (synapse) is significantly different from time constant (synapse) of: Amphiphysin (p = 2.71E-04), Dynamin 1 (p = 1.01E-03), Epsin (p = 1.70E-03), ITSN 1-L (p = 3.48E-03), mEGFP (p = 7.99E-05), PIP5KI-gamma (p = 5.10E-04), SCAMP1 (p = 4.43E-05), SV2B (p = 2.15E-02), Synapsin 1A (p = 4.31E-05), Synaptogyrin (p = 8.06E-06), Synaptophysin (p = 4.85E-07), Synaptotagmin 1 (p = 2.39E-07), Synaptotagmin 7 (p = 7.72E-03), Syntaxin 1A (p = 1.00E-03), Syntaxin 16 (p = 1.38E-06), alpha-Tubulin 1b (p = 1.95E-04), VAMP1 (p = 3.98E-06), vATPase V0a1 (p = 2.01E-02), vGluT1 (p = 5.44E-07), Vti1a-beta (p = 4.64E-03).
Immobile fraction (synapse) is significantly different from immobile fraction (synapse) of: Rab5a (p = 2.47E-02), SV2B (p = 9.59E-06), Synaptogyrin (p = 2.74E-02), Synaptophysin (p = 2.91E-06), vATPase V0a1 (p = 3.05E-02), vGluT1 (p = 8.84E-04).

References

Jahn, R., and Scheller, R.H. (2006). Nat Rev Mol Cell Biol 7, 631-43.
Takamori, S., et al. (2006). Cell 127, 831-46.
Sorensen, J.B., et al. (2003). Cell 114, 75-86.

SNAP25

MW (kDa)	Category	τ_{axon} (s)	$\tau_{synapse}$ (s)	IF_{axon} (%)	$IF_{synapse}$ (%)
23.31	plasma membrane-associated	1.89 ± 0.17	4.38 ± 0.24	17.90 ± 3.31	19.49 ± 3.39

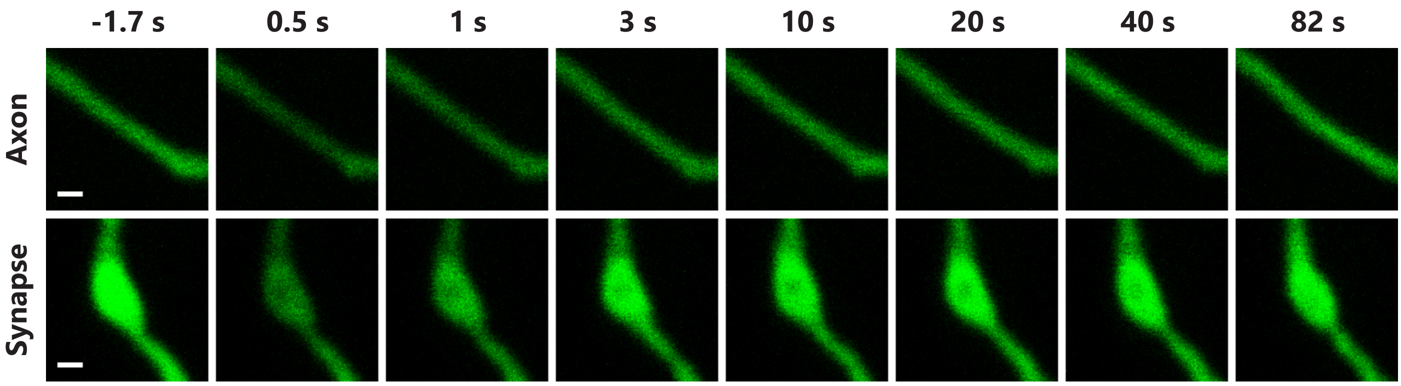

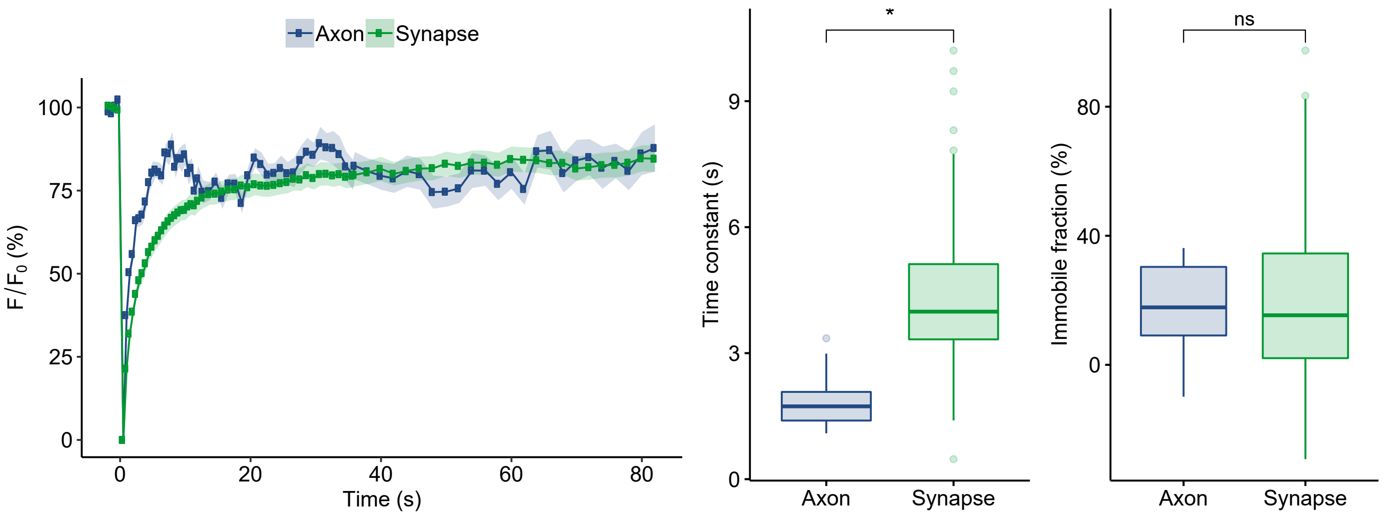

N (axons) = 16 N (synapses) = 65; p (time constant) = 1.18E-07.

Tagged protein outline:

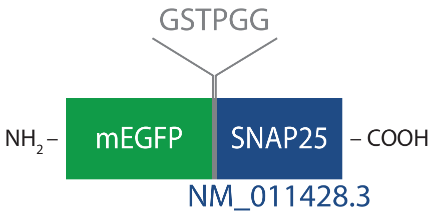

Time constant (axon) is significantly different from time constant (axon) of: , Synaptotagmin 1 (p = 2.17E-02), Syntaxin 1A (p = 4.78E-02).
Immobile fraction (axon) is not significantly different from immobile fraction (axon) of any other proteins.
Time constant (synapse) is significantly different from time constant (synapse) of: Amphiphysin (p = 2.71E-03), Dynamin 1 (p = 1.44E-04), ITSN 1-L (p = 3.49E-04), mEGFP (p = 8.10E-13), PIP5KI-gamma (p = 1.57E-03), SCAMP1 (p = 3.72E-06), SV2B (p = 6.10E-04), Synapsin 1A (p = 2.03E-07), Synaptogyrin (p = 1.16E-08), Synaptophysin (p = 6.30E-12), Synaptotagmin 1 (p = 3.10E-12), Syntaxin 1A (p = 4.43E-03), Syntaxin 16 (p = 4.90E-09), alpha-Tubulin 1b (p = 5.16E-06), VAMP1 (p = 3.44E-09), vATPase V0a1 (p = 7.65E-04), vGluT1 (p = 2.19E-11), Vti1a-beta (p = 9.96E-05).
Immobile fraction (synapse) is significantly different from immobile fraction (synapse) of: , SCAMP1 (p = 3.21E-02), SV2B (p = 1.17E-09), Synaptogyrin (p = 5.57E-04), Synaptophysin (p = 1.44E-09), Synaptotagmin 1 (p = 1.29E-03), VAMP4 (p = 2.92E-02), vATPase V0a1 (p = 9.08E-05), vGluT1 (p = 2.12E-06).

References

Walch-Solimena C., et. al. (1995). J Cell Biol 128, 637-45.
Takamori, S., et al. (2006). Cell 127, 831-46.
Bar-On, D., et. al. (2012). J Biol Chem 287, 27158-67.

SNAP29

MW (kDa)	Category	τ_{axon} (s)	$\tau_{synapse}$ (s)	IF_{axon} (%)	$IF_{synapse}$ (%)
29.07	plasma membrane-associated	3.65 ± 0.31	5.41 ± 0.32	6.58 ± 4.51	10.64 ± 3.28

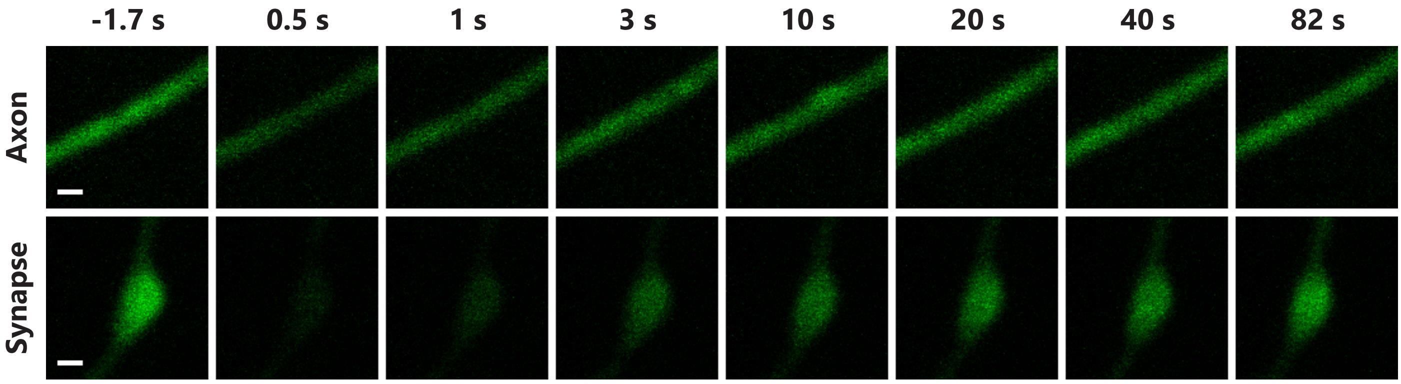

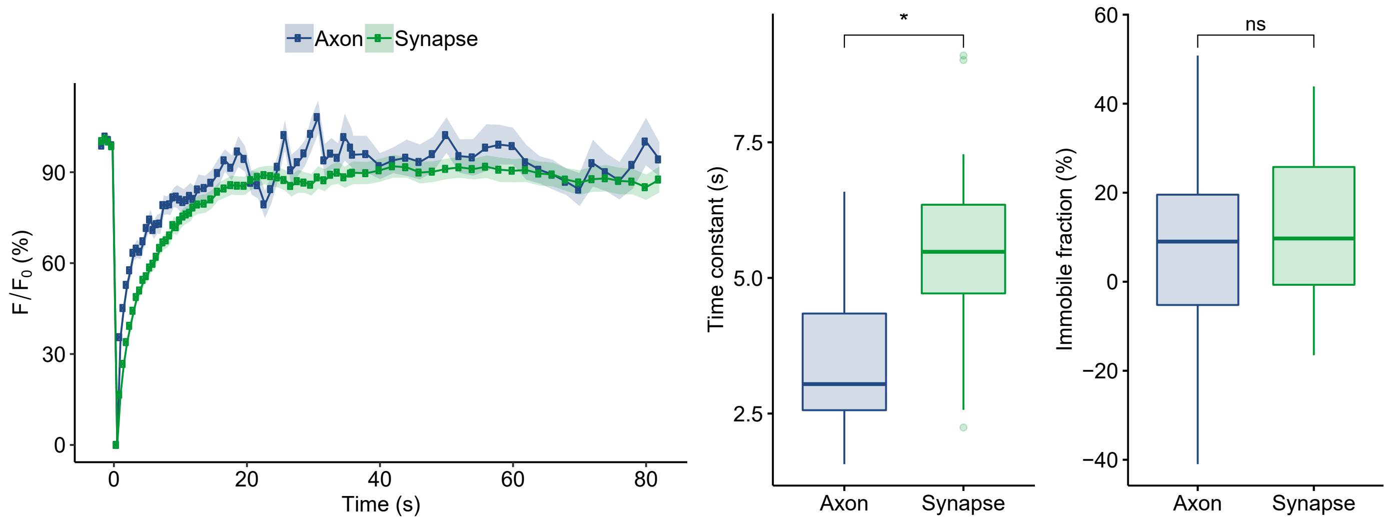

N (axons) = 25, N (synapses) = 35; p (time constant) = 5.29E-04.

Tagged protein outline:

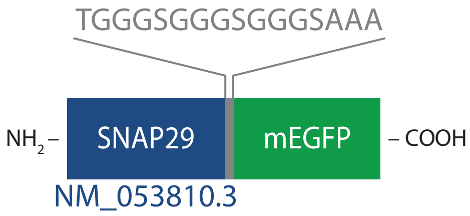

Time constant (axon) is significantly different from time constant (axon) of: Actin (p = 4.97E-05), AP180 (p = 3.55E-02), Clathrin light chain B (p = 5.30E-03), Doc2a (p = 4.27E-05), ITSN 1-L (p = 4.56E-02), mEGFP (p = 5.91E-03), Munc13 (p = 8.64E-06), Rab5a (p = 8.11E-04), Rab7a (p = 1.56E-03), Syndapin 1 (p = 1.16E-02).
Immobile fraction (axon) is not significantly different from immobile fraction (axon) of any other proteins.
Time constant (synapse) is significantly different from time constant (synapse) of: Calmodulin 1 (p = 1.67E-03), Clathrin light chain B (p = 1.62E-02), Hsc70 (p = 5.47E-04), mEGFP (p = 8.59E-10), SCAMP1 (p = 4.07E-03), Synapsin 1A (p = 6.27E-04), Synaptogyrin (p = 5.23E-05), Synaptophysin (p = 3.92E-07), Synaptotagmin 1 (p = 6.71E-08), Syntaxin 16 (p = 2.55E-03), alpha-Tubulin 1b (p = 1.74E-02), VAMP1 (p = 1.37E-05), vGluT1 (p = 9.68E-07).
Immobile fraction (synapse) is significantly different from immobile fraction (synapse) of: NSF (p = 1.46E-03), SCAMP1 (p = 6.83E-03), SV2B (p = 2.25E-07), Synaptogyrin (p = 6.89E-04), Synaptophysin (p = 5.06E-08), Synaptotagmin 1 (p = 3.38E-04), VAMP2 (p = 2.47E-03), VAMP4 (p = 1.46E-03), vATPase V0a1 (p = 6.41E-05), vGluT1 (p = 7.63E-06).

References

Peng, J., et al. (2004). J Biol Chem 279, 21003-11.
Takamori, S., et al. (2006). Cell 127, 831-846.

SV2B

MW (kDa)	Category	τ_{axon} (s)	$\tau_{synapse}$ (s)	IF_{axon} (%)	$IF_{synapse}$ (%)
77.50	vesicular	6.85 ± 1.45	17.35 ± 2.88	28.68 ± 10.33	75.35 ± 5.19

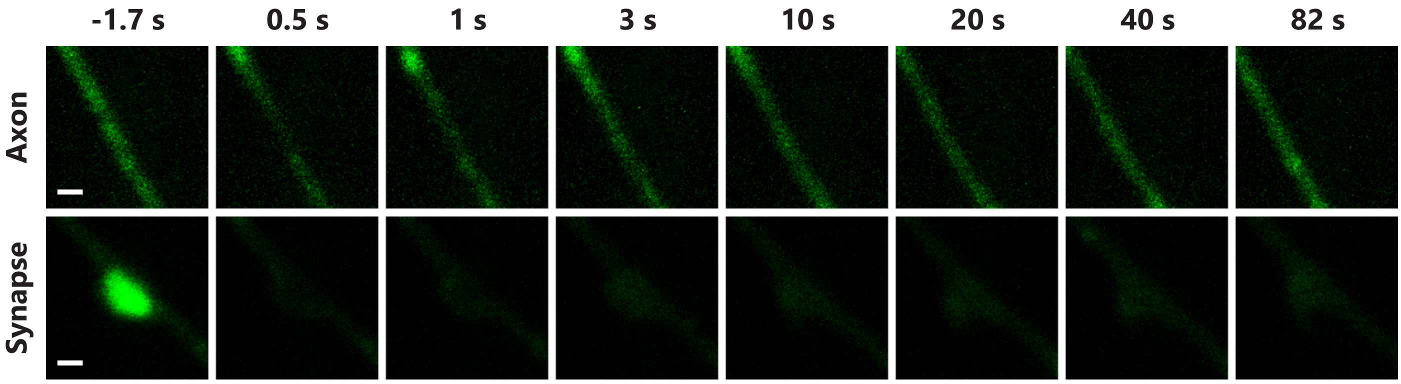

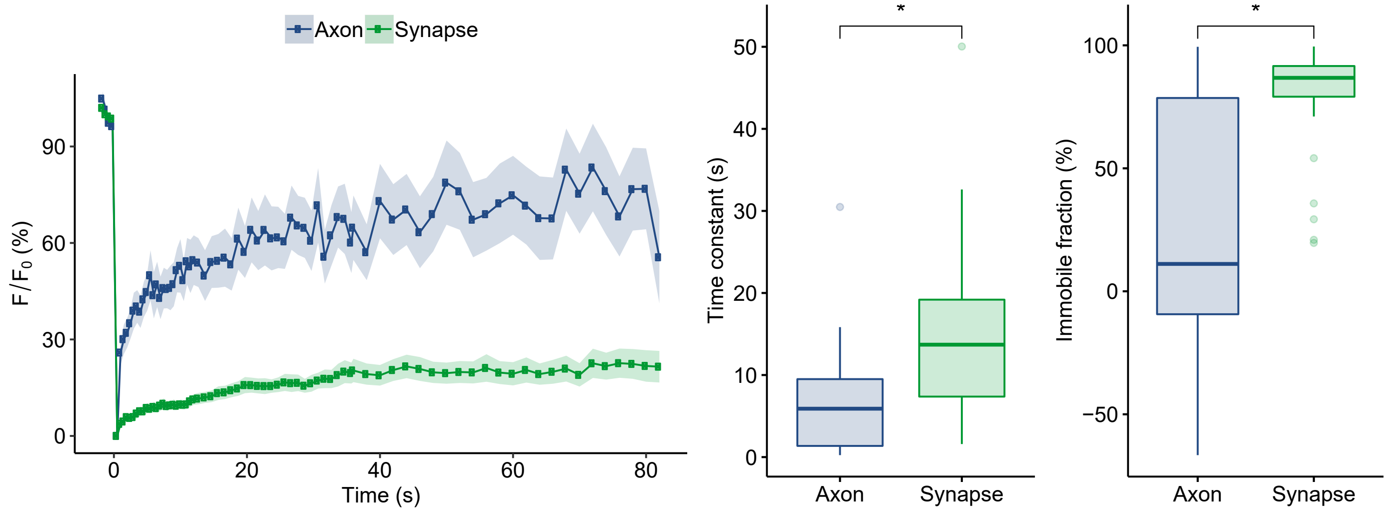

N (axons) = 23, N (synapses) = 35; p (time constant) = 7.83E-04, p (immobile fraction) = 1.66E-04.

Tagged protein outline:

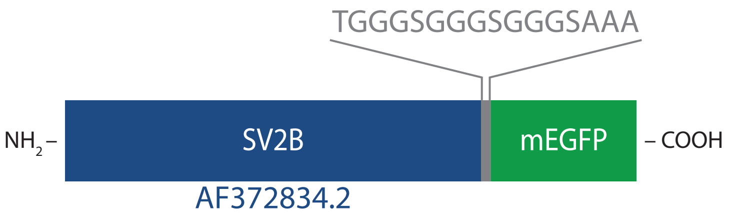

Time constant (axon) is not significantly different from time constant (axon) of any other proteins.
Immobile fraction (axon) is not significantly different from immobile fraction (axon) of any other proteins.
Time constant (synapse) is significantly different from time constant (synapse) of: AP180 (p = 9.12E-03), AP2 (p = 6.85E-03), Calmodulin 1 (p = 8.46E-04), Clathrin light chain B (p = 1.32E-03), Complexin 1 (p = 2.04E-03), Doc2a (p = 4.00E-02), Hsc70 (p = 1.82E-05), mEGFP (p = 2.00E-09), membrane mEGFP (p = 4.51E-02), Munc13 (p = 7.96E-04), Munc18 (p = 3.27E-04), NSF (p = 5.79E-03), Rab3a (p = 6.95E-03), Rab5a (p = 7.56E-04), Rab7a (p = 6.85E-03), Septin 5 (p = 9.04E-03), SNAP23 (p = 2.15E-02), SNAP25 (p = 6.10E-04).
Immobile fraction (synapse) is significantly different from immobile fraction (synapse) of: Actin (p = 7.07E-06), alpha-SNAP (p = 5.49E-07), alpha-synuclein (p = 2.43E-06), Amphiphysin (p = 1.24E-06), AP180 (p = 1.74E-08), AP2 (p = 6.71E-07), Calmodulin 1 (p = 1.94E-06), Clathrin light chain B (p = 6.61E-07), Complexin 1 (p = 5.41E-07), Complexin 2 (p = 2.47E-07), Doc2a (p = 8.17E-07), Dynamin 1 (p = 4.80E-03), Endophilin A1 (p = 9.59E-07), Epsin (p = 4.21E-05), Hsc70 (p = 5.25E-08), ITSN 1-L (p = 1.03E-02), mEGFP (p = 1.94E-11), membrane mEGFP (p = 5.76E-06), Munc13 (p = 5.12E-09), Munc18 (p = 3.07E-12), NSF (p = 5.79E-03), PIP5KI-gamma (p = 9.59E-06), Rab3a (p = 4.42E-07), Rab5a (p = 1.73E-10), Rab7a (p = 1.56E-07), Septin 5 (p = 1.66E-07), SNAP23 (p = 9.59E-06), Munc13 (p = 5.12E-09), SNAP29 (p = 2.25E-07), Synapsin 1A (p = 9.19E-05), Synaptotagmin 1 (p = 2.35E-03), Synaptotagmin 7 (p = 4.05E-07), Syndapin 1 (p = 3.22E-07), Syntaxin 1A (p = 3.90E-07), Syntaxin 16 (p = 5.17E-08), alpha-Tubulin 1b (p = 4.89E-03), VAMP1 (p = 2.88E-05), VAMP2 (p = 3.74E-04), Vti1a-beta (p = 2.40E-03).

References

Buckley, K., and Kelly, R.B. (1985). J Cell Biol 100, 1284-94.
Bajjalieh, S.M., et al. (1992). Science 257, 1271-3.
Takamori, S., et al. (2006). Cell 127, 831-46.

Synapsin 1A

MW (kDa)	Category	τ_{axon} (s)	$\tau_{synapse}$ (s)	IF_{axon} (%)	$IF_{synapse}$ (%)
73.99	soluble, vesicle tethering	5.84 ± 1.03	13.38 ± 1.19	19.74 ± 8.70	23.51 ± 8.84

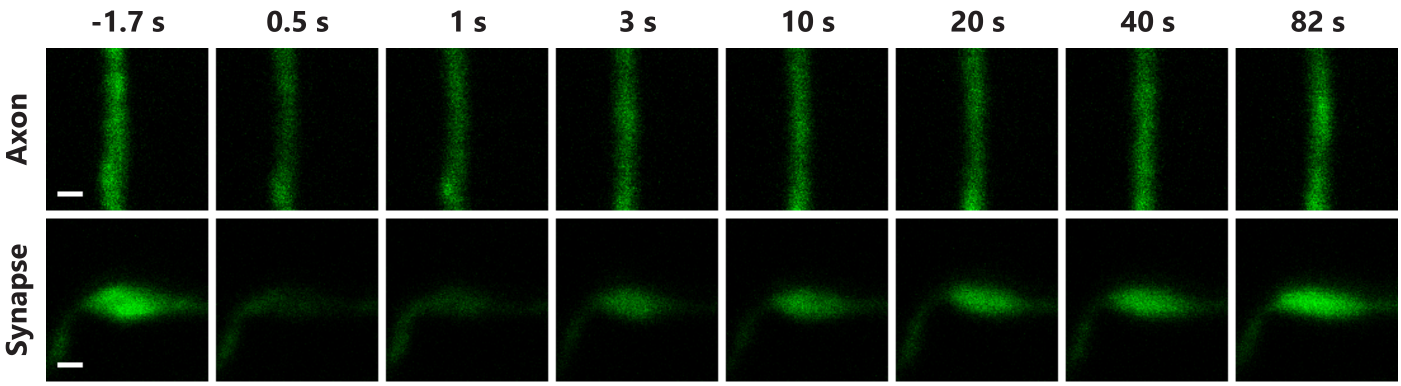

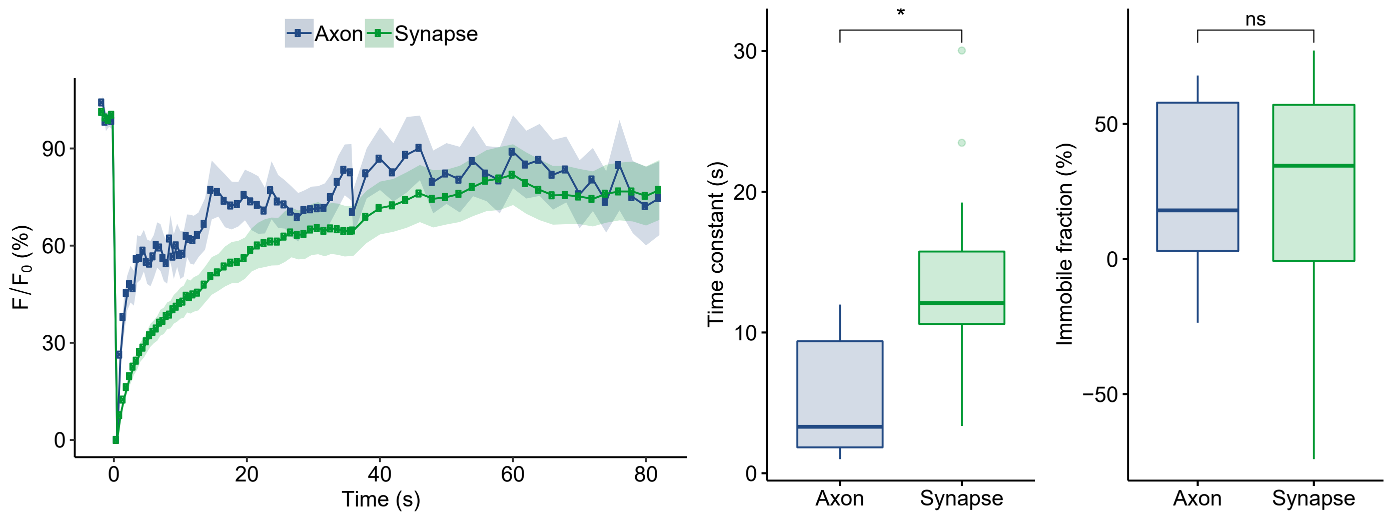

N (axons) = 17, N (synapses) = 24; p (time constant) = 4.66E-05.

Tagged protein outline:

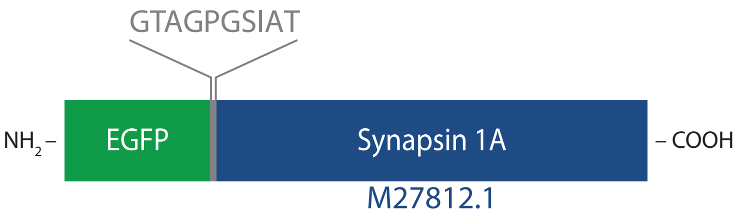

Time constant (axon) is significantly different from time constant (axon) of: Actin (p = 2.82E-02), Munc13 (p = 2.47E-02).
Immobile fraction (axon) is not significantly different from immobile fraction (axon) of any other proteins.
Time constant (synapse) is significantly different from time constant (synapse) of: Actin (p = 1.04E-02), alpha-SNAP (p = 4.39E-04), alpha-synuclein (p = 4.14E-03), Amphiphysin (p = 1.34E-03), AP180 (p = 2.84E-05), AP2 (p = 1.07E-05), Calmodulin 1 (p = 4.45E-06), Clathrin light chain B (p = 3.95E-06), Complexin 1 (p = 1.19E-05), Complexin 2 (p = 1.45E-04), Doc2a (p = 3.38E-04), Endophilin A1 (p = 3.45E-05), Epsin (p = 3.75E-02), Hsc70 (p = 3.53E-07), mEGFP (p = 7.77E-10), membrane mEGFP (p = 1.91E-04), Munc13 (p = 9.74E-07), Munc18 (p = 1.88E-07), NSF (p = 3.83E-05), PIP5KI-gamma (p = 4.70E-02), Rab3a (p = 3.19E-05), Rab5a (p = 8.31E-07), Rab7a (p = 2.26E-05), Septin 5 (p = 4.86E-06), SNAP23 (p = 4.31E-05), SNAP25 (p = 2.03E-07), SNAP29 (p = 6.27E-04), Synaptotagmin 7 (p = 1.48E-03), Syndapin 1 (p = 1.54E-04), Syntaxin 1A (p = 2.76E-03), VAMP2 (p = 8.19E-04), VAMP4 (p = 3.30E-04).
Immobile fraction (synapse) is significantly different from immobile fraction (synapse) of: SV2B (p = 9.19E-05), Synaptophysin (p = 4.95E-04).

References

Cesca, F., et al. (2010). Prog Neurobiol 91, 313-48.
Siksou, L., et al. (2007). J Neurosci 27, 6868-77.
Hirokawa, N., et al. (1989). J Cell Biol 108, 111-26.
Takamori, S., et al. (2006). Cell 127, 831-46.

Synaptogyrin

MW (kDa)	Category	τ_{axon} (s)	$\tau_{synapse}$ (s)	IF_{axon} (%)	$IF_{synapse}$ (%)
25.67	vesicular	-	33.65 ± 4.19	-	65.35 ± 6.54

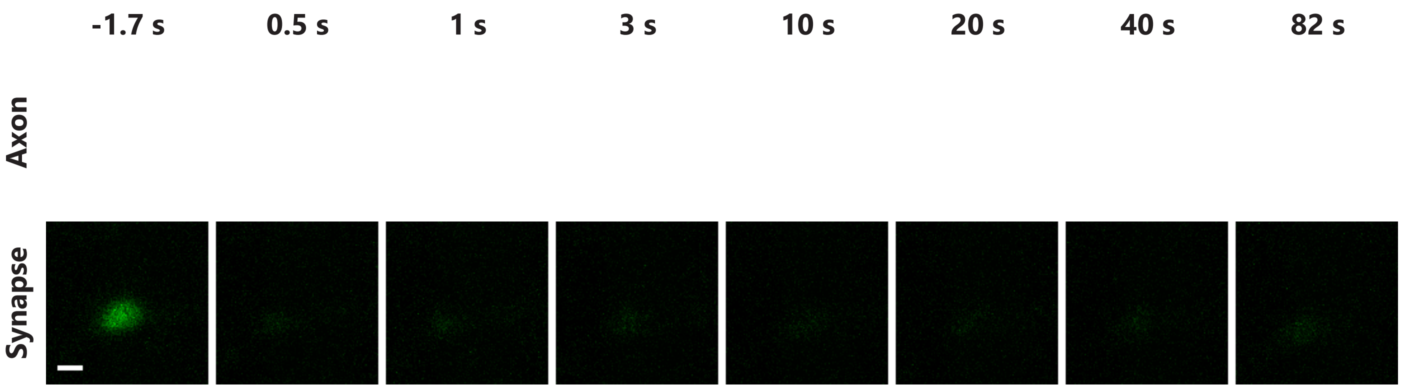

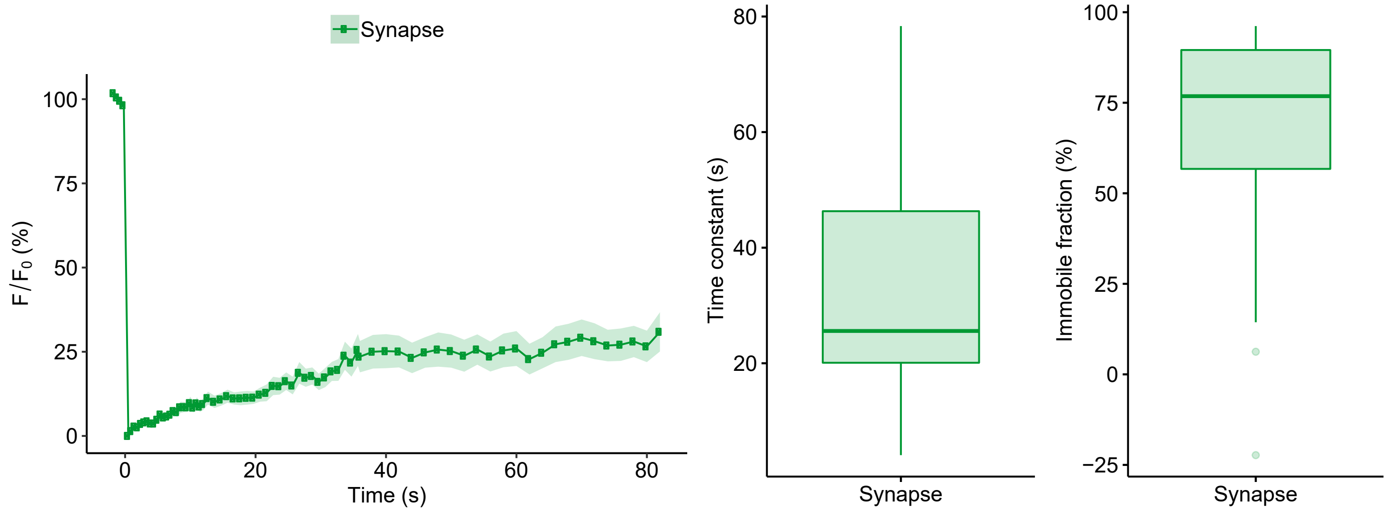

N (axons) = 0, N (synapses) = 24.

Tagged protein outline:

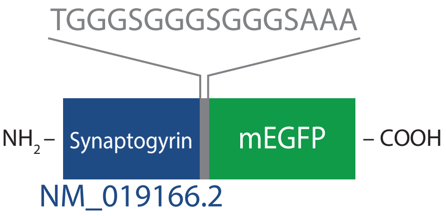

Time constant (synapse) is significantly different from time constant (synapse) of: Actin (p = 1.08E-04), alpha-SNAP (p = 4.97E-05), alpha-synuclein (p = 1.85E-05), Amphiphysin (p = 1.88E-05), AP180 (p = 3.84E-06), AP2 (p = 2.85E-06), Calmodulin 1 (p = 1.62E-06), Clathrin light chain B (p = 1.05E-06), Complexin 1 (p = 2.22E-06), Complexin 2 (p = 1.48E-05), CSP (p = 6.67E-04), Doc2a (p = 3.19E-05), Dynamin 1 (p = 7.16E-03), Endophilin A1 (p = 5.55E-06), Epsin (p = 2.17E-03), Hsc70 (p = 9.92E-08), ITSN 1-L (p = 7.83E-04), mEGFP (p = 6.34E-10), membrane mEGFP (p = 1.90E-05), Munc13 (p = 1.09E-07), Munc18 (p = 1.01E-08), NSF (p = 1.16E-05), PIP5KI-gamma (p = 6.32E-04), Rab3a (p = 5.36E-06), Rab5a (p = 1.28E-07), Rab7a (p = 5.55E-06), Septin 5 (p = 6.51E-07), SNAP23 (p = 8.06E-06), SNAP25 (p = 1.16E-08), SNAP29 (p = 5.23E-05), Synaptotagmin 7 (p = 4.32E-05), Syndapin 1 (p = 2.18E-05), Syntaxin 1A (p = 8.74E-05), Syntaxin 16 (p = 5.38E-03), alpha-Tubulin 1b (p = 4.71E-03), VAMP2 (p = 1.08E-04), VAMP4 (p = 6.11E-05), vATPase V0a1 (p = 1.58E-02).
Immobile fraction (synapse) is significantly different from immobile fraction (synapse) of: Actin (p = 3.08E-02), alpha-SNAP (p = 7.39E-04), alpha-synuclein (p = 2.02E-02), Amphiphysin (p = 2.05E-02), AP180 (p = 2.45E-04), AP2 (p = 1.46E-03), Calmodulin 1 (p = 1.32E-02), Clathrin light chain B (p = 8.30E-03), Complexin 1 (p = 8.84E-03), Complexin 2 (p = 1.60E-03), Doc2a (p = 5.99E-03), Endophilin A1 (p = 5.14E-03), Epsin (p = 3.02E-02), Hsc70 (p = 2.96E-03), mEGFP (p = 3.29E-05), membrane mEGFP (p = 4.54E-02), Munc13 (p = 8.53E-05), Munc18 (p = 2.52E-05), PIP5KI-gamma (p = 1.18E-02), Rab3a (p = 1.62E-03), Rab5a (p = 1.64E-05), Rab7a (p = 2.16E-04), Septin 5 (p = 4.69E-03), SNAP23 (p = 2.74E-02), Munc13 (p = 8.53E-05), SNAP29 (p = 6.89E-04), Synaptotagmin 7 (p = 7.84E-04), Syndapin 1 (p = 2.13E-03), Syntaxin 1A (p = 1.10E-03), Syntaxin 16 (p = 6.27E-04),

References

Baumert, M., et al. (1990). J Cell Biol 110, 1285-94.
Jahn, R., et al. (1985). Proc Natl Acad Sci U S A 82, 4127-41.
Takamori, S., et al. (2006). Cell 127, 831-46.

Synaptophysin

MW (kDa)	Category	τ_{axon} (s)	$\tau_{synapse}$ (s)	IF_{axon} (%)	$IF_{synapse}$ (%)
33.31	vesicular	3.08 ± 0.64	24.60 ± 3.53	22.26 ± 7.20	72.59 ± 2.99

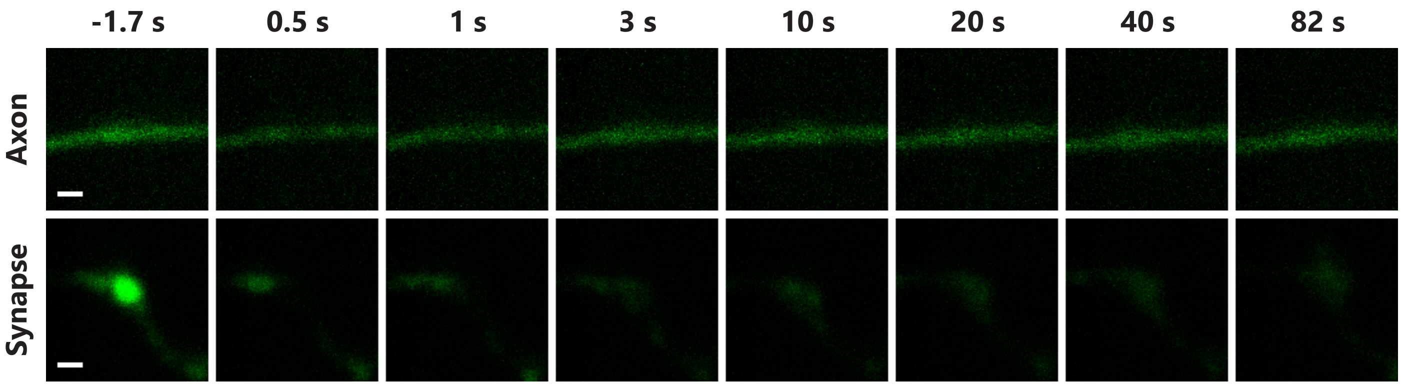

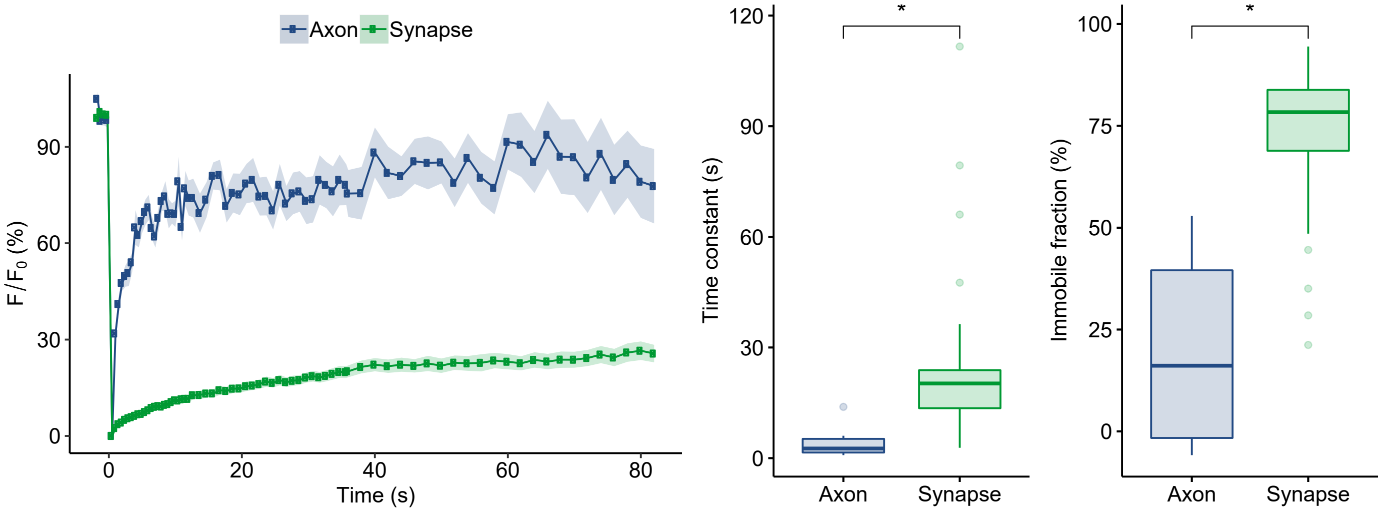

N (axons) = 10, N (synapses) = 36; p (time constant) = 4.53E-06, p (immobile fraction) = 7.29E-06.

Tagged protein outline:

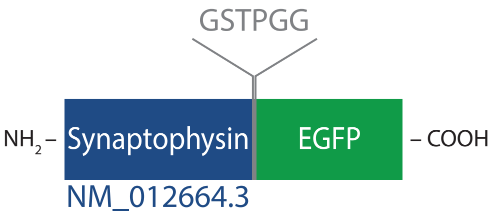

Time constant (axon) is not significantly different from time constant (axon) of any other proteins.
Immobile fraction (axon) is not significantly different from immobile fraction (axon) of any other proteins.
Time constant (synapse) is significantly different from time constant (synapse) of: Actin (p = 1.04E-05), alpha-SNAP (p = 1.47E-06), alpha-synuclein (p = 1.13E-06), Amphiphysin (p = 1.85E-07), AP180 (p = 2.55E-08), AP2 (p = 7.96E-08), Calmodulin 1 (p = 4.21E-08), Clathrin light chain B (p = 1.15E-08), Complexin 1 (p = 3.82E-08), Complexin 2 (p = 3.29E-07), CSP (p = 1.49E-04), Doc2a (p = 3.26E-07), Dynamin 1 (p = 2.57E-02), Endophilin A1 (p = 1.78E-07), Epsin (p = 1.15E-04), Hsc70 (p = 4.40E-10), ITSN 1-L (p = 6.11E-04), mEGFP (p = 2.09E-13), membrane mEGFP (p = 1.79E-07), Munc13 (p = 4.08E-10), Munc18 (p = 1.66E-12), NSF (p = 4.40E-07), PIP5KI-gamma (p = 2.97E-05), Rab3a (p = 6.84E-08), Rab5a (p = 3.31E-10), Rab7a (p = 1.04E-07), Septin 5 (p = 8.36E-09), SNAP23 (p = 4.85E-07), SNAP25 (p = 6.30E-12), SNAP29 (p = 3.92E-07), Synaptotagmin 7 (p = 5.41E-07), Syndapin 1 (p = 2.11E-07), Syntaxin 1A (p = 1.30E-06), alpha-Tubulin 1b (p = 1.10E-02), VAMP2 (p = 2.76E-06), VAMP4 (p = 1.83E-06).
Immobile fraction (synapse) is significantly different from immobile fraction (synapse) of: Actin (p = 1.04E-05), alpha-SNAP (p = 2.06E-07), alpha-synuclein (p = 3.63E-06), Amphiphysin (p = 1.92E-06), AP180 (p = 4.81E-09), AP2 (p = 2.53E-07), Calmodulin 1 (p = 1.98E-06), Clathrin light chain B (p = 3.64E-06), Complexin 1 (p = 1.64E-07), Complexin 2 (p = 1.04E-07), Doc2a (p = 1.06E-07), Endophilin A1 (p = 1.94E-07), Epsin (p = 2.59E-05), Hsc70 (p = 1.25E-08), mEGFP (p = 2.69E-12), membrane mEGFP (p = 8.57E-06), Munc13 (p = 6.77E-09), Munc18 (p = 1.92E-13), NSF (p = 6.85E-03), PIP5KI-gamma (p = 6.05E-06), Rab3a (p = 8.15E-08), Rab5a (p = 5.12E-11), Rab7a (p = 5.06E-08), Septin 5 (p = 1.54E-07), SNAP23 (p = 2.91E-06), Munc13 (p = 6.77E-09), SNAP29 (p = 5.06E-08), Synapsin 1A (p = 4.95E-04), Synaptotagmin 7 (p = 7.47E-08), Syndapin 1 (p = 5.89E-08), Syntaxin 1A (p = 1.14E-07), Syntaxin 16 (p = 3.97E-08), alpha-Tubulin 1b (p = 2.16E-02), VAMP1 (p = 3.85E-05), VAMP2 (p = 3.03E-04), Vti1a-beta (p = 3.66E-02).

References

Jahn, R., et al. (1985). Proc Natl Acad Sci U S A 82, 4137-41.
Reisinger, C., et al. (2004). J Neurochem 90, 1-8.
Takamori, S., et al. (2006). Cell 127, 831-846.

Synaptotagmin 1

MW (kDa)	Category	τ_{axon} (s)	$\tau_{synapse}$ (s)	IF_{axon} (%)	$IF_{synapse}$ (%)
47.40	vesicular	6.48 ± 0.96	25.36 ± 3.28	6.77 ± 7.34	26.66 ± 13.46

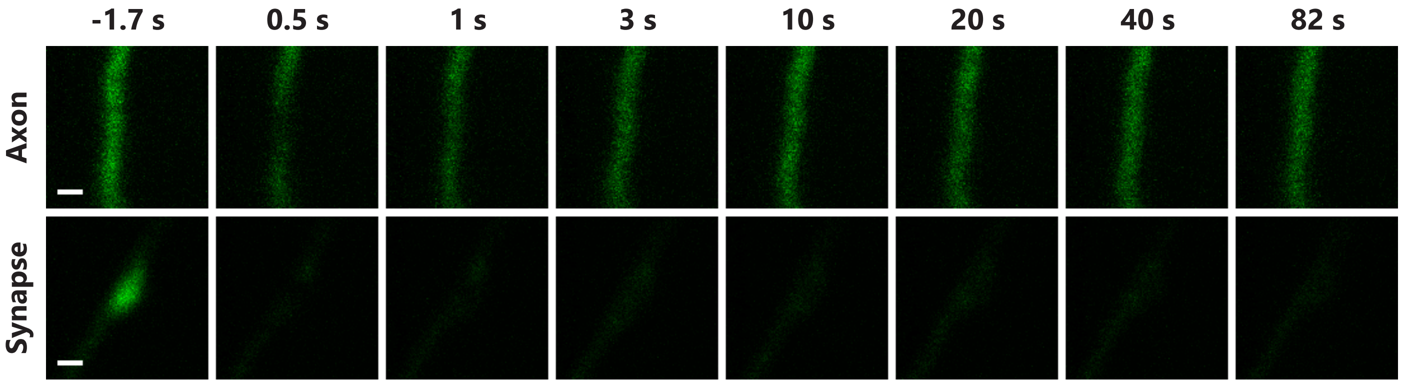

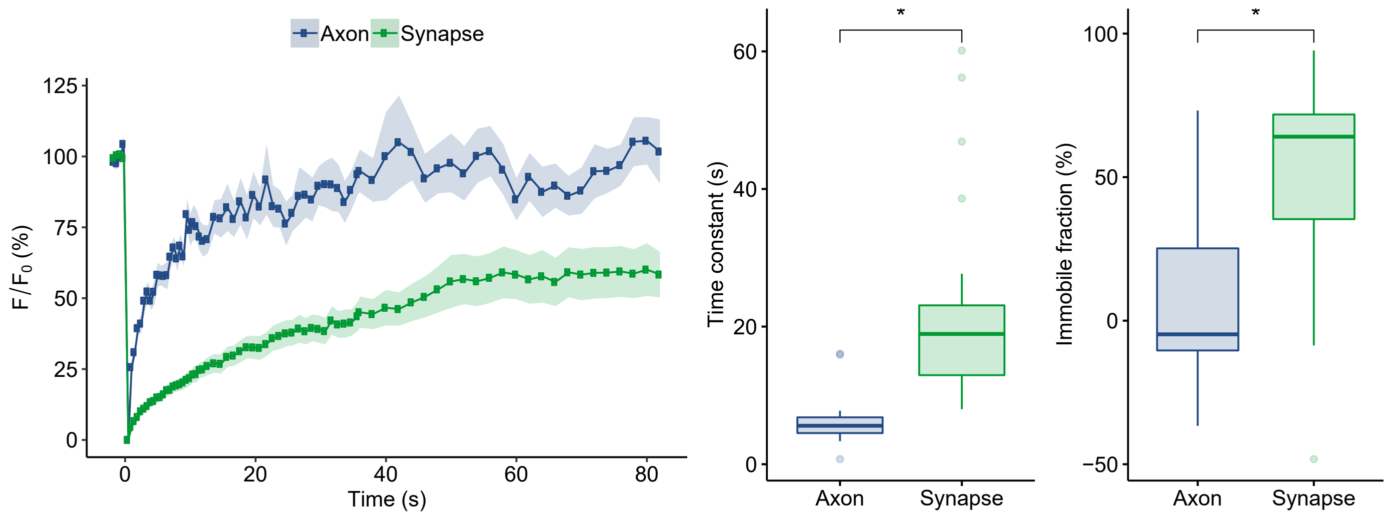

N (axons) = 17, N (synapses) = 35; p (time constant) = 2.52E-07, p (immobile fraction) = 6.88E-05.

Tagged protein outline:

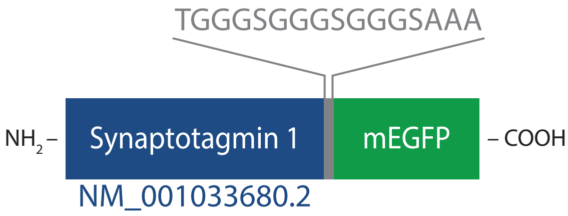

Time constant (axon) is significantly different from time constant (axon) of: Actin (p = 1.97E-03), AP180 (p = 1.78E-03), AP2 (p = 2.10E-03), Calmodulin 1 (p = 2.13E-02), Clathrin light chain B (p = 2.01E-03), Doc2a (p = 9.18E-04), Endophilin A1 (p = 8.12E-03), Hsc70 (p = 3.80E-02), ITSN 1-L (p = 1.35E-02), mEGFP (p = 1.50E-03), membrane mEGFP (p = 2.87E-02), Munc13 (p = 8.17E-05), NSF (p = 2.19E-02), Rab5a (p = 5.57E-03), Rab7a (p = 1.74E-03), SNAP23 (p = 2.87E-02), SNAP25 (p = 2.17E-02), Synaptotagmin 7 (p = 2.82E-02), Syndapin 1 (p = 1.38E-03).
Immobile fraction (axon) is not significantly different from immobile fraction (axon) of any other proteins.
Time constant (synapse) is significantly different from time constant (synapse) of: Actin (p = 4.59E-06), alpha-SNAP (p = 3.37E-07), alpha-synuclein (p = 1.00E-06), Amphiphysin (p = 7.09E-08), AP180 (p = 6.50E-09), AP2 (p = 3.69E-08), Calmodulin 1 (p = 3.69E-08), Clathrin light chain B (p = 7.19E-09), Complexin 1 (p = 2.12E-08), Complexin 2 (p = 6.71E-08), CSP (p = 8.25E-05), Doc2a (p = 1.03E-07), Dynamin 1 (p = 4.71E-02), Endophilin A1 (p = 3.69E-08), Epsin (p = 2.10E-05), Hsc70 (p = 4.70E-10), ITSN 1-L (p = 1.75E-03), mEGFP (p = 1.67E-12), membrane mEGFP (p = 3.92E-08), Munc13 (p = 1.14E-10), Munc18 (p = 4.96E-13), NSF (p = 2.39E-07), PIP5KI-gamma (p = 5.34E-06), Rab3a (p = 2.40E-08), Rab5a (p = 1.14E-10), Rab7a (p = 3.69E-08), Septin 5 (p = 1.72E-09), SNAP23 (p = 2.39E-07), SNAP25 (p = 3.10E-12), SNAP29 (p = 6.71E-08), Synaptotagmin 7 (p = 7.74E-08), Syndapin 1 (p = 3.43E-08), Syntaxin 1A (p = 2.39E-07), alpha-Tubulin 1b (p = 2.41E-02), VAMP2 (p = 2.74E-07), VAMP4 (p = 3.29E-07).
Immobile fraction (synapse) is significantly different from immobile fraction (synapse) of: alpha-SNAP (p = 4.68E-04), AP180 (p = 4.74E-05), AP2 (p = 2.81E-03), Complexin 1 (p = 9.74E-03), Complexin 2 (p = 1.07E-03), Doc2a (p = 5.01E-03), Endophilin A1 (p = 4.33E-03), Hsc70 (p = 1.12E-02), mEGFP (p = 7.13E-06), Munc13 (p = 1.72E-04), Munc18 (p = 2.69E-06), Rab3a (p = 1.21E-03), Rab5a (p = 1.58E-06), Rab7a (p = 1.65E-04), Septin 5 (p = 4.16E-02), Munc13 (p = 1.72E-04), SNAP29 (p = 3.38E-04), SV2B (p = 2.35E-03), Synaptotagmin 7 (p = 7.20E-04), Syndapin 1 (p = 2.05E-03), Syntaxin 1A (p = 1.25E-03), Syntaxin 16 (p = 1.70E-02),

References

Opazo, F., et al. (2010). Traffic 11, 800-12.
Takamori, S., et al. (2006). Cell 127, 831-46.

Synaptotagmin 7

MW (kDa)	Category	τ_{axon} (s)	$\tau_{synapse}$ (s)	IF_{axon} (%)	$IF_{synapse}$ (%)
45.48	vesicular	2.66 ± 0.29	5.90 ± 0.39	19.51 ± 2.66	8.21 ± 5.15

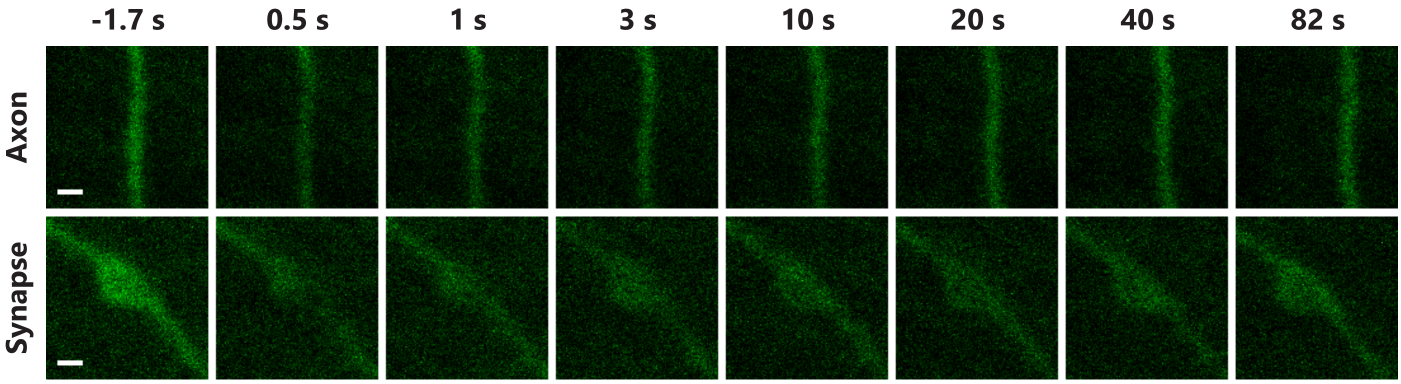

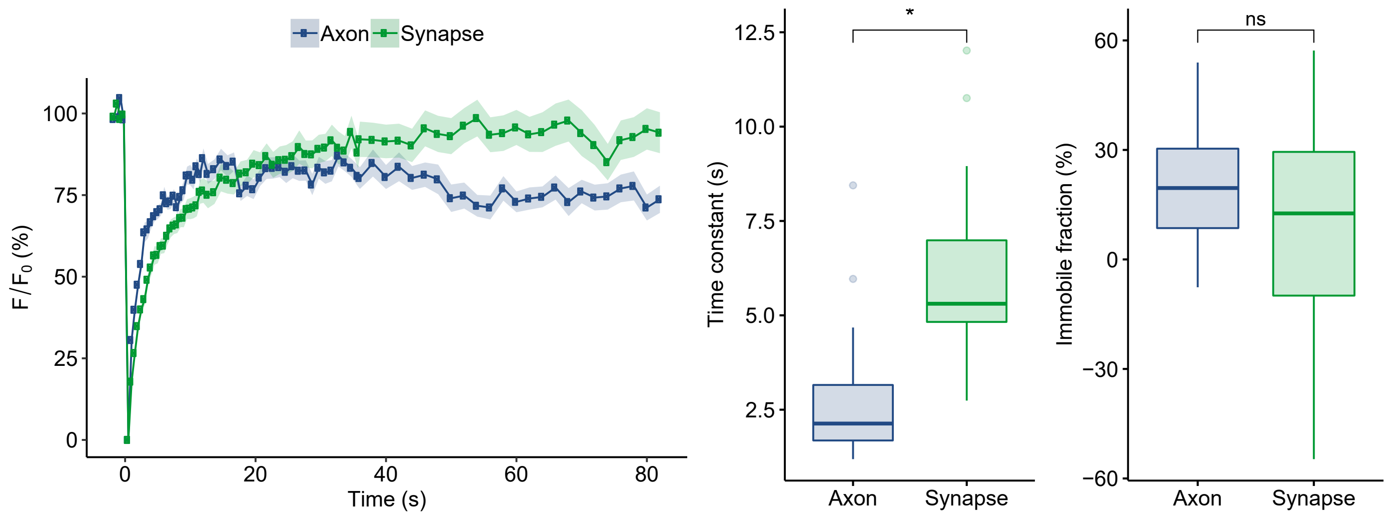

N (axons) = 29, N (synapses) = 29; p (time constant) = 3.09E-08.

Tagged protein outline:

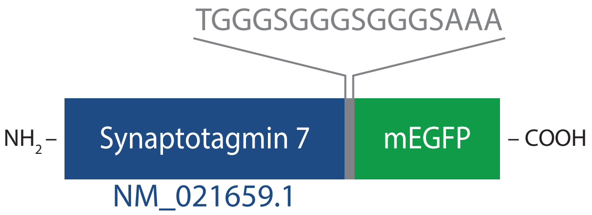

Time constant (axon) is significantly different from time constant (axon) of: Actin (p = 9.12E-03), Munc13 (p = 8.65E-03), Synaptotagmin 1 (p = 2.82E-02).
Immobile fraction (axon) is not significantly different from immobile fraction (axon) of any other proteins.
Time constant (synapse) is significantly different from time constant (synapse) of: AP2 (p = 4.55E-03), Calmodulin 1 (p = 1.59E-04), Clathrin light chain B (p = 2.48E-03), Hsc70 (p = 7.21E-05), mEGFP (p = 5.55E-11), NSF (p = 2.43E-02), Rab5a (p = 3.81E-02), SCAMP1 (p = 1.71E-02), SNAP23 (p = 7.72E-03), Synapsin 1A (p = 1.48E-03), Synaptogyrin (p = 4.32E-05), Synaptophysin (p = 5.41E-07), Synaptotagmin 1 (p = 7.74E-08), Syntaxin 16 (p = 9.27E-03), VAMP1 (p = 4.22E-05), vGluT1 (p = 2.47E-06).
Immobile fraction (synapse) is significantly different from immobile fraction (synapse) of: NSF (p = 1.27E-02), SCAMP1 (p = 7.84E-03), SV2B (p = 4.05E-07), Synaptogyrin (p = 7.84E-04), Synaptophysin (p = 7.47E-08), Synaptotagmin 1 (p = 7.20E-04), VAMP2 (p = 1.16E-02), VAMP4 (p = 5.50E-03), vATPase V0a1 (p = 9.62E-05), vGluT1 (p = 7.23E-06).

References

Fernandez, I., et al. (2001). Neuron 32, 1057-69.
Takamori, S., et al. (2006). Cell 127, 831-46.

Syndapin 1

MW (kDa)	Category	τ_{axon} (s)	$\tau_{synapse}$ (s)	IF_{axon} (%)	$IF_{synapse}$ (%)
50.45	soluble, endocytic	2.00 ± 0.14	5.30 ± 0.36	28.10 ± 2.78	16.17 ± 3.59

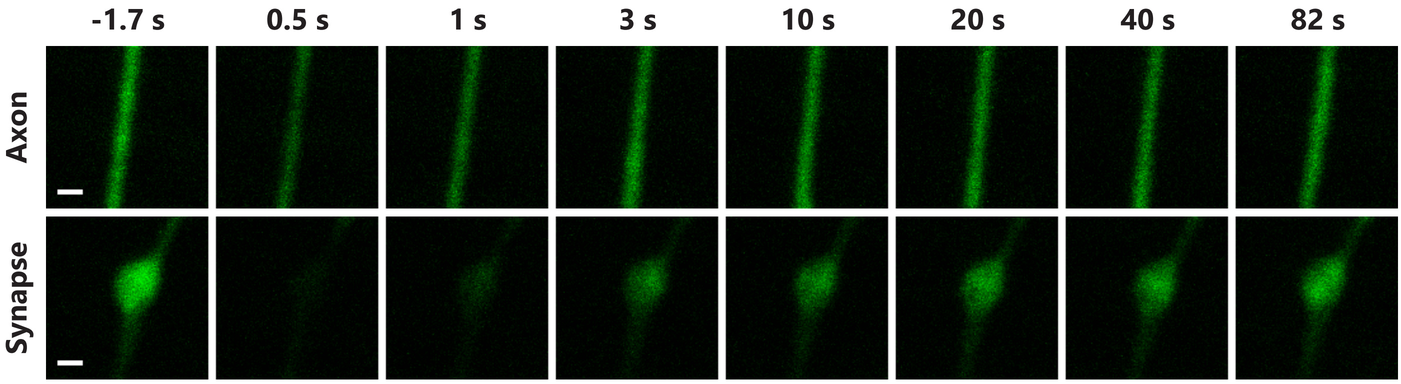

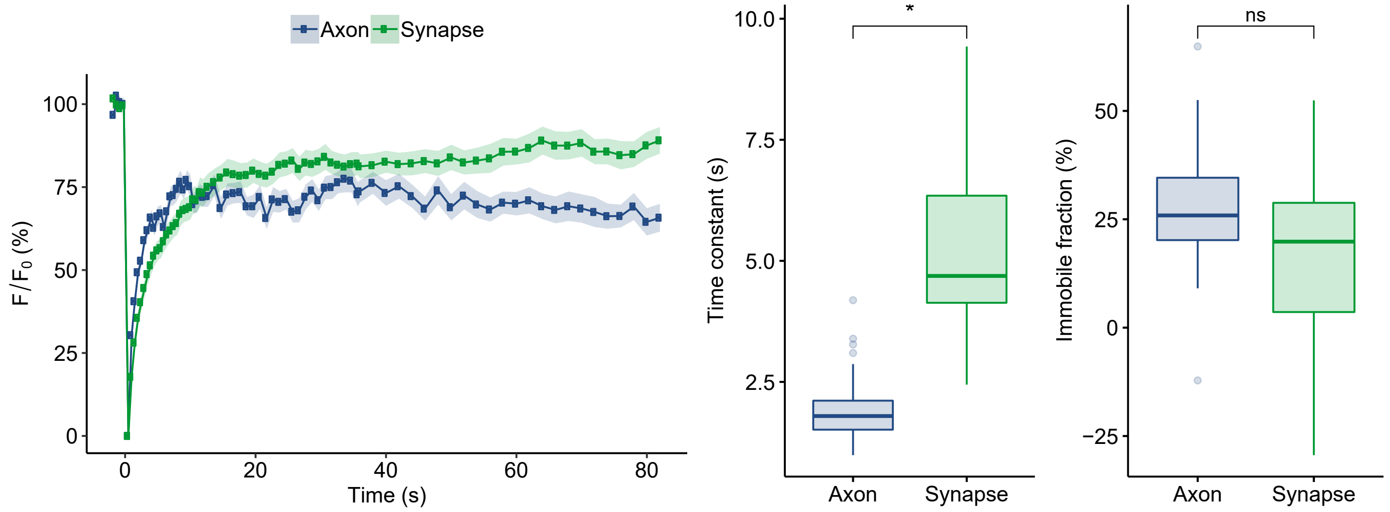

N (axons) = 29, N (synapses) = 30; p (time constant) = 7.84E-10.

Tagged protein outline:

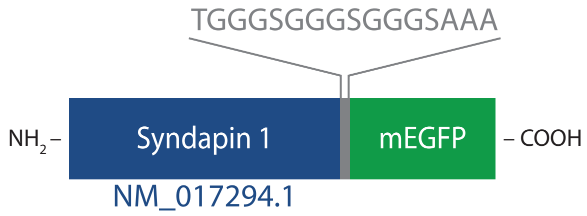

Time constant (axon) is significantly different from time constant (axon) of: SNAP29 (p = 1.16E-02), Synaptotagmin 1 (p = 1.38E-03), Syntaxin 1A (p = 9.67E-03), VAMP1 (p = 2.41E-02).
Immobile fraction (axon) is significantly different from immobile fraction (axon) of: Endophilin A1 (p = 1.12E-02), VAMP2 (p = 4.91E-02).
Time constant (synapse) is significantly different from time constant (synapse) of: Calmodulin 1 (p = 1.06E-02), Hsc70 (p = 2.09E-03), mEGFP (p = 2.70E-10), SCAMP1 (p = 4.52E-03), Synapsin 1A (p = 1.54E-04), Synaptogyrin (p = 2.18E-05), Synaptophysin (p = 2.11E-07), Synaptotagmin 1 (p = 3.43E-08), Syntaxin 16 (p = 5.78E-04), alpha-Tubulin 1b (p = 9.82E-03), VAMP1 (p = 1.42E-05), vGluT1 (p = 5.53E-07), Vti1a-beta (p = 4.84E-02).
Immobile fraction (synapse) is significantly different from immobile fraction (synapse) of: NSF (p = 1.82E-02), SCAMP1 (p = 1.90E-02), SV2B (p = 3.22E-07), Synaptogyrin (p = 2.13E-03), Synaptophysin (p = 5.89E-08), Synaptotagmin 1 (p = 2.05E-03), VAMP2 (p = 1.73E-02), VAMP4 (p = 1.82E-02), vATPase V0a1 (p = 2.77E-04), vGluT1 (p = 1.42E-05).

References

Kessels, M.M., and Qualmann, B. (2004). J Cell Sci 117, 3077-86.
Anggono, V., et al. (2006). Nat Neurosci 9, 752-60.
Kessels, M.M., and Qualmann, B. (2006). J Biol Chem 281. 13285-99.

Syntaxin 1A

MW (kDa)	Category	τ_{axon} (s)	$\tau_{synapse}$ (s)	IF_{axon} (%)	$IF_{synapse}$ (%)
33.07	plasma membrane	3.42 ± 0.20	6.56 ± 0.42	11.94 ± 4.61	12.66 ± 4.08

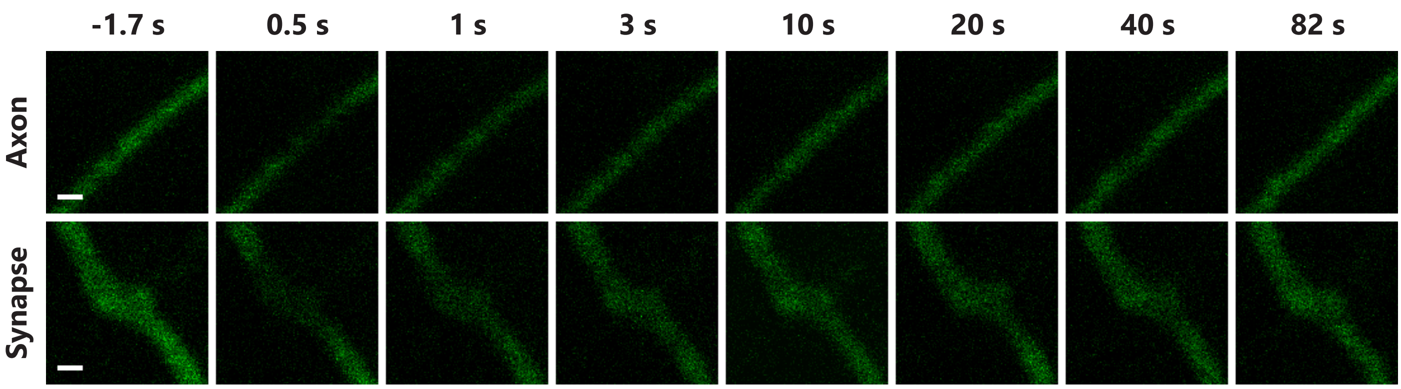

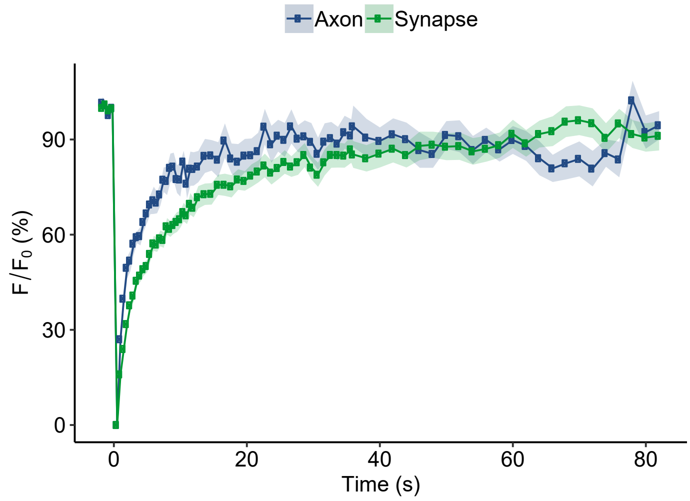

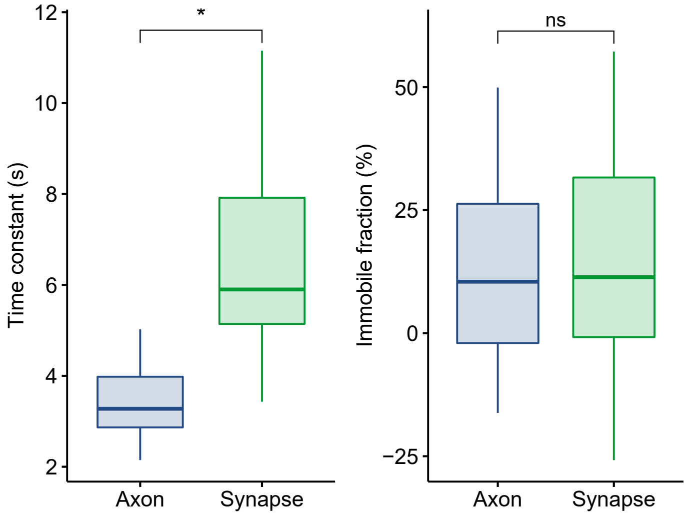

N (axons) = 16, N (synapses) = 28; p (time constant) = 1.13E-06.

Tagged protein outline:

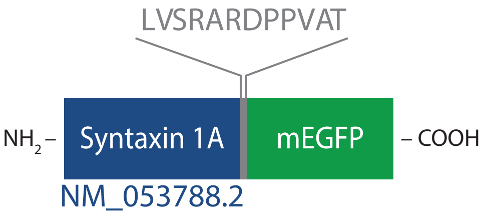

Time constant (axon) is significantly different from time constant (axon) of: Actin (p = 3.88E-04), Clathrin light chain B (p = 3.07E-03), Doc2a (p = 8.24E-05), mEGFP (p = 1.50E-03), Munc13 (p = 2.36E-04), Rab5a (p = 1.15E-03), Rab7a (p = 7.74E-03), SNAP25 (p = 4.78E-02), Syndapin 1 (p = 9.67E-03).
Immobile fraction (axon) is not significantly different from immobile fraction (axon) of any other proteins.
Time constant (synapse) is significantly different from time constant (synapse) of: AP2 (p = 4.35E-04), Calmodulin 1 (p = 2.00E-05), Clathrin light chain B (p = 2.10E-04), Complexin 1 (p = 1.22E-02), Hsc70 (p = 5.02E-06), mEGFP (p = 3.71E-11), Munc13 (p = 7.17E-03), NSF (p = 2.52E-03), Rab3a (p = 2.49E-02), Rab5a (p = 6.81E-04), Rab7a (p = 2.34E-02), SNAP23 (p = 1.00E-03), SNAP25 (p = 4.43E-03), Synapsin 1A (p = 2.76E-03), Synaptogyrin (p = 8.74E-05), Synaptophysin (p = 1.30E-06), Synaptotagmin 1 (p = 2.39E-07), VAMP1 (p = 3.99E-04), vGluT1 (p = 1.52E-05).
Immobile fraction (synapse) is significantly different from immobile fraction (synapse) of: NSF (p = 3.28E-02), SCAMP1 (p = 1.38E-02), SV2B (p = 3.90E-07), Synaptogyrin (p = 1.10E-03), Synaptophysin (p = 1.14E-07), Synaptotagmin 1 (p = 1.25E-03), VAMP2 (p = 2.02E-02), VAMP4 (p = 1.22E-02), vATPase V0a1 (p = 2.54E-04), vGluT1 (p = 2.39E-05).

References

Bennett, M.K., et al. (2004). Neuron 41, 495-511.
Sieber, J.J., et al. (2007). Science 317, 1072-6.
Takamori, S., et al. (2006). Cell 127, 831-46.

Syntaxin 16

MW (kDa)	Category	τ_{axon} (s)	$\tau_{synapse}$ (s)	IF_{axon} (%)	$IF_{synapse}$ (%)
35.44	endosomal	3.42 ± 0.26	12.63 ± 1.08	2.40 ± 5.16	9.14 ± 6.72

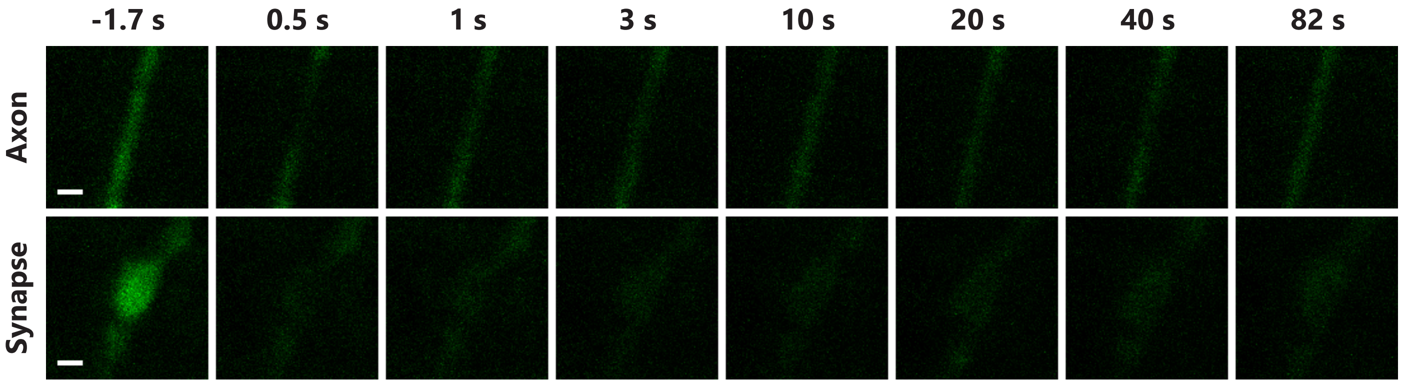

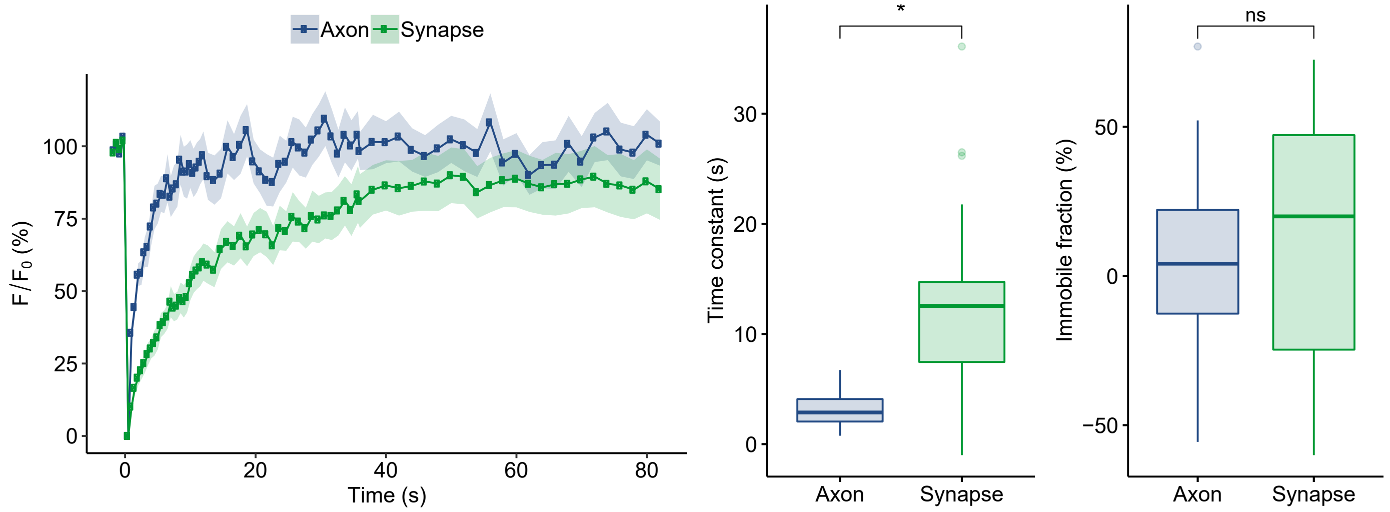

N (axons) = 42, N (synapses) = 44; p (time constant) = 1.29E-12.

Tagged protein outline:

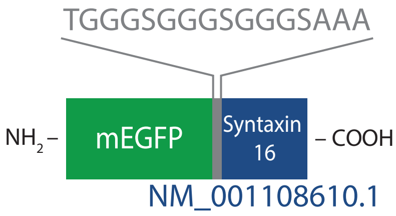

Time constant (axon) is significantly different from time constant (axon) of: Actin (p = 4.95E-05), Clathrin light chain B (p = 1.74E-02), Doc2a (p = 5.13E-04), Munc13 (p = 4.07E-06), Rab5a (p = 3.71E-03), Rab7a (p = 5.21E-03).
Immobile fraction (axon) is not significantly different from immobile fraction (axon) of any other proteins.
Time constant (synapse) is significantly different from time constant (synapse) of: Actin (p = 2.66E-02), alpha-SNAP (p = 2.97E-03), alpha-synuclein (p = 1.06E-02), AP180 (p = 1.31E-05), AP2 (p = 2.81E-07), Calmodulin 1 (p = 4.02E-08), Clathrin light chain B (p = 6.33E-08), Complexin 1 (p = 5.21E-07), Complexin 2 (p = 2.41E-04), Doc2a (p = 2.21E-03), Endophilin A1 (p = 4.96E-06), Hsc70 (p = 7.36E-10), mEGFP (p = 2.52E-15), membrane mEGFP (p = 2.19E-04), Munc13 (p = 1.42E-08), Munc18 (p = 7.39E-09), NSF (p = 2.63E-06), Rab3a (p = 5.62E-06), Rab5a (p = 4.98E-09), Rab7a (p = 3.01E-06), Septin 5 (p = 8.21E-07), SNAP23 (p = 1.38E-06), SNAP25 (p = 4.90E-09), SNAP29 (p = 2.55E-03), Synaptogyrin (p = 5.38E-03), Synaptotagmin 7 (p = 9.27E-03), Syndapin 1 (p = 5.78E-04), VAMP2 (p = 4.85E-03), VAMP4 (p = 6.27E-04).
Immobile fraction (synapse) is significantly different from immobile fraction (synapse) of: , SCAMP1 (p = 1.97E-02), SV2B (p = 5.17E-08), Synaptogyrin (p = 6.27E-04), Synaptophysin (p = 3.97E-08), Synaptotagmin 1 (p = 1.70E-02), vATPase V0a1 (p = 1.65E-03), vGluT1 (p = 4.59E-05).

References

Simonsen, A., et al. (1998). Eur J Cell Biol 75, 223-31.
Chen, Y., et al. (2010). J Cell Physiol 225, 326-32.
Takamori, S., et al. (2006). Cell 127, 831-46.

α-Tubulin

MW (kDa)	Category	τ_{axon} (s)	$\tau_{synapse}$ (s)	IF_{axon} (%)	$IF_{synapse}$ (%)
50.15	cytoskeletal	7.04 ± 1.89	10.61 ± 0.93	45.48 ± 11.49	35.23 ± 6.40

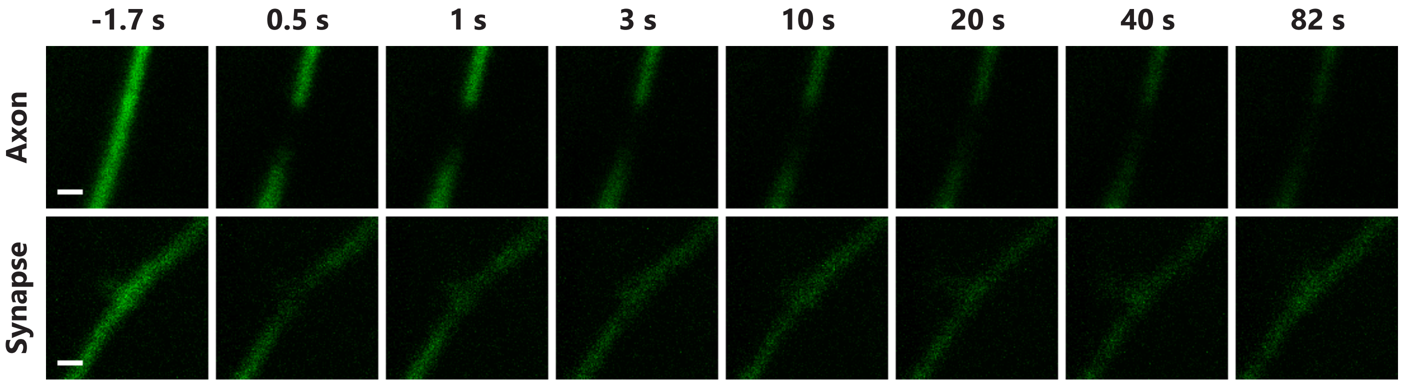

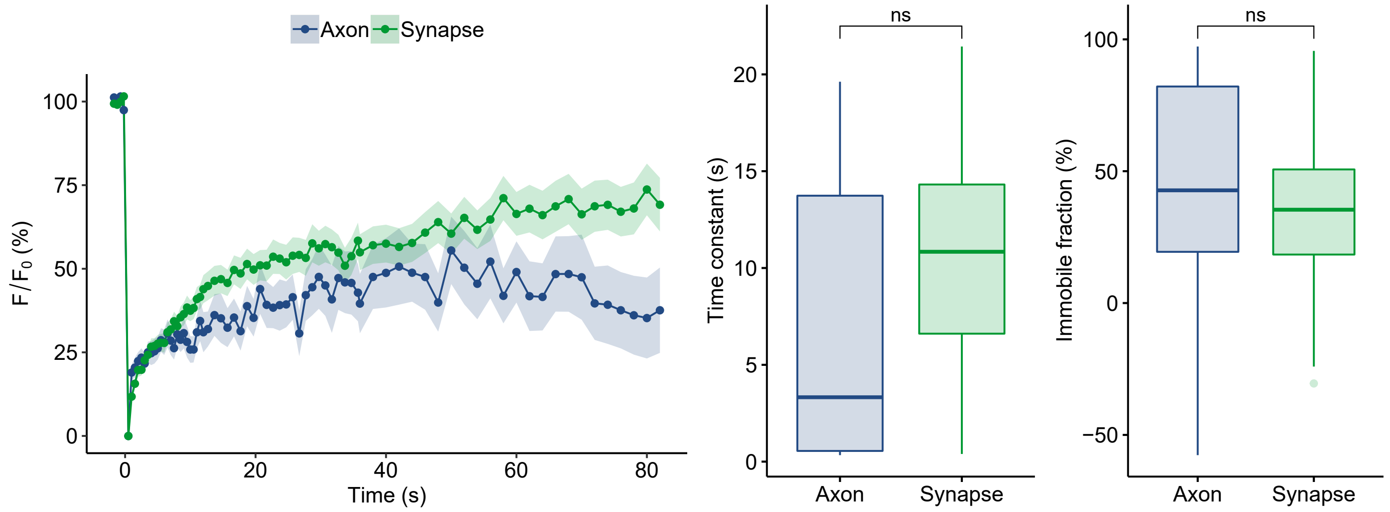

N (axons) = 15, N (synapses) = 28.

Tagged protein outline:

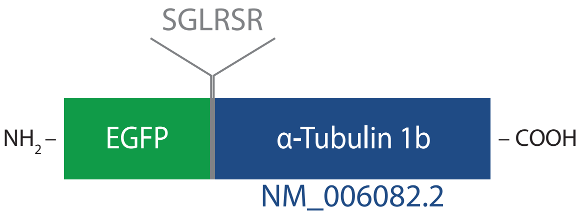

Time constant (axon) is not significantly different from time constant (axon) of any other proteins.
Immobile fraction (axon) is not significantly different from immobile fraction (axon) of any other proteins.
Time constant (synapse) is significantly different from time constant (synapse) of: alpha-SNAP (p = 3.00E-02), AP180 (p = 5.43E-04), AP2 (p = 9.69E-05), Calmodulin 1 (p = 3.52E-05), Clathrin light chain B (p = 3.75E-05), Complexin 1 (p = 2.33E-04), Complexin 2 (p = 6.48E-03), Endophilin A1 (p = 9.35E-04), Hsc70 (p = 1.60E-06), mEGFP (p = 3.06E-09), membrane mEGFP (p = 4.21E-03), Munc13 (p = 1.50E-05), Munc18 (p = 1.70E-05), NSF (p = 3.19E-04), Rab3a (p = 3.45E-04), Rab5a (p = 6.29E-06), Rab7a (p = 2.58E-04), Septin 5 (p = 3.37E-04), SNAP23 (p = 1.95E-04), SNAP25 (p = 5.16E-06), SNAP29 (p = 1.74E-02), Synaptogyrin (p = 4.71E-03), Synaptophysin (p = 1.10E-02), Synaptotagmin 1 (p = 2.41E-02), Syndapin 1 (p = 9.82E-03), VAMP4 (p = 1.32E-02).
Immobile fraction (synapse) is significantly different from immobile fraction (synapse) of: , SV2B (p = 4.89E-03), Synaptophysin (p = 2.16E-02).

References

Hirokawa, N., et al. (1989). J Cell Biol 108, 111-26.
Conde, C. and Caceres, A. (2009). Nat Rev Neurosci 10, 319-32.

VAMP1

MW (kDa)	Category	τ_{axon} (s)	$\tau_{synapse}$ (s)	IF_{axon} (%)	$IF_{synapse}$ (%)
12.80	vesicular	3.38 ± 0.25	13.64 ± 1.01	9.68 ± 3.65	32.25 ± 4.39

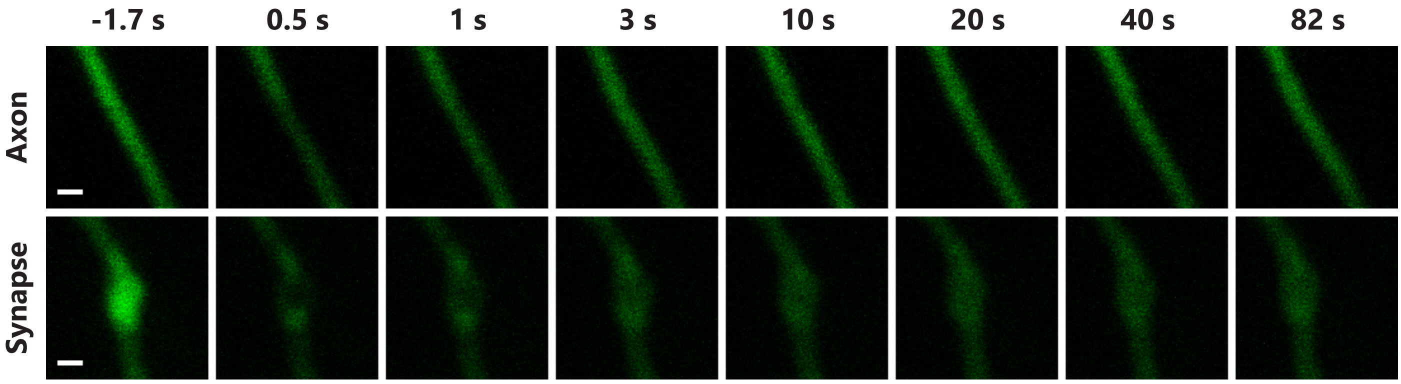

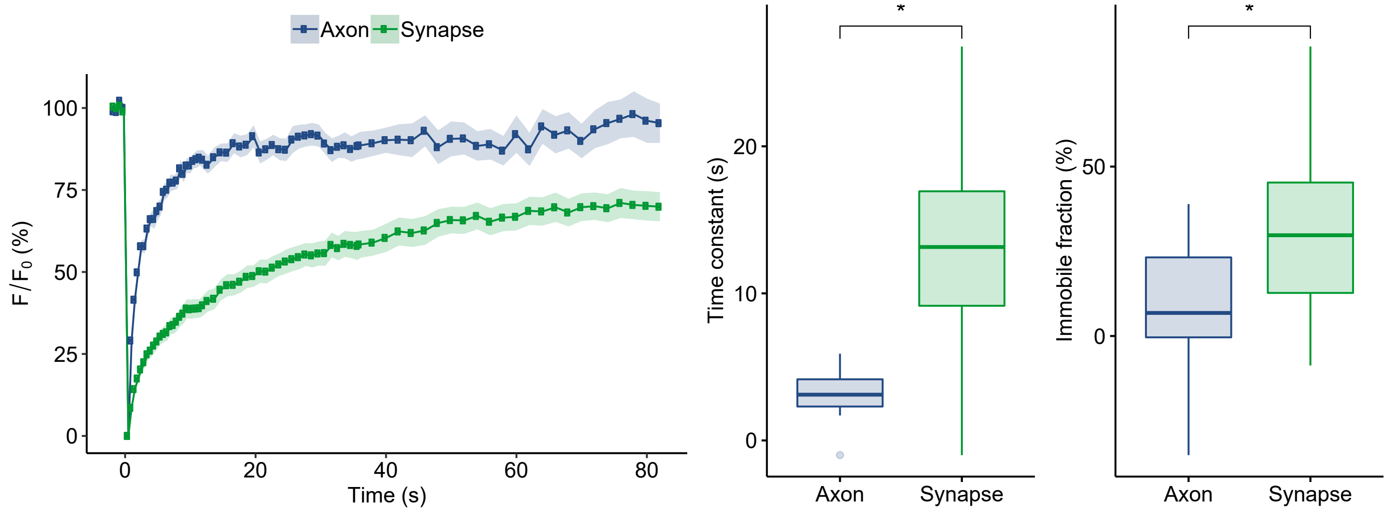

N (axons) = 21, N (synapses) = 28; p (time constant) = 1.15E-08, p (immobile fraction) = 1.10E-03.

Tagged protein outline:

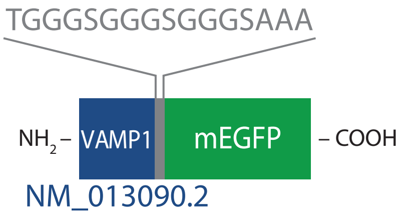

Time constant (axon) is significantly different from time constant (axon) of: Actin (p = 1.96E-04), Clathrin light chain B (p = 9.12E-03), Doc2a (p = 1.75E-04), mEGFP (p = 1.21E-02), Munc13 (p = 4.30E-05), Rab5a (p = 2.70E-03), Rab7a (p = 6.19E-03), Syndapin 1 (p = 2.41E-02).
Immobile fraction (axon) is not significantly different from immobile fraction (axon) of any other proteins.
Time constant (synapse) is significantly different from time constant (synapse) of: Actin (p = 2.70E-03), alpha-SNAP (p = 5.23E-05), alpha-synuclein (p = 1.08E-03), Amphiphysin (p = 2.45E-04), AP180 (p = 1.28E-06), AP2 (p = 1.14E-06), Calmodulin 1 (p = 4.98E-07), Clathrin light chain B (p = 2.63E-07), Complexin 1 (p = 1.12E-06), Complexin 2 (p = 1.66E-05), Doc2a (p = 5.52E-05), Endophilin A1 (p = 2.84E-06), Epsin (p = 6.83E-03), Hsc70 (p = 1.40E-08), mEGFP (p = 3.48E-11), membrane mEGFP (p = 6.56E-06), Munc13 (p = 2.71E-08), Munc18 (p = 2.01E-09), NSF (p = 4.45E-06), PIP5KI-gamma (p = 4.31E-03), Rab3a (p = 1.02E-06), Rab5a (p = 1.15E-08), Rab7a (p = 1.40E-06), Septin 5 (p = 2.55E-07), SNAP23 (p = 3.98E-06), SNAP25 (p = 3.44E-09), SNAP29 (p = 1.37E-05), Synaptotagmin 7 (p = 4.22E-05), Syndapin 1 (p = 1.42E-05), Syntaxin 1A (p = 3.99E-04), VAMP2 (p = 1.16E-04), VAMP4 (p = 2.80E-05).
Immobile fraction (synapse) is significantly different from immobile fraction (synapse) of: Rab5a (p = 2.04E-02), SV2B (p = 2.88E-05), Synaptophysin (p = 3.85E-05), vGluT1 (p = 1.48E-02).

References

Trimble, W.S., et al. (1998). Proc Natl Acad Sci U S A 85, 4528-42.
Raptis, A., et al. (2005). J Chem Neuroanat 30, 201-11.
Takamori, S., et al. (2006). Cell 127, 831-46.

VAMP2

MW (kDa)	Category	τ_{axon} (s)	$\tau_{synapse}$ (s)	IF_{axon} (%)	$IF_{synapse}$ (%)
12.69	vesicular	2.64 ± 0.20	5.53 ± 0.45	-11.95 ± 7.29	41.67 ± 4.07

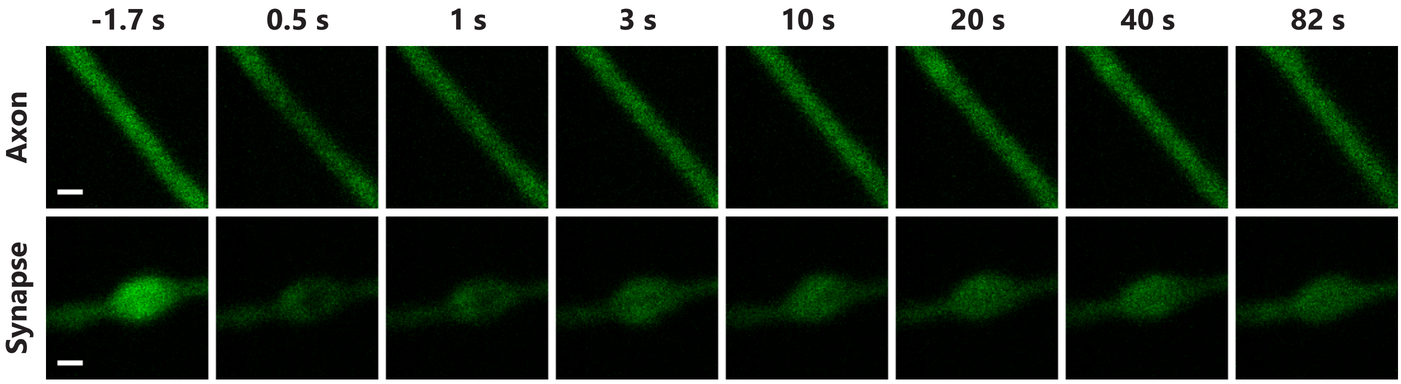

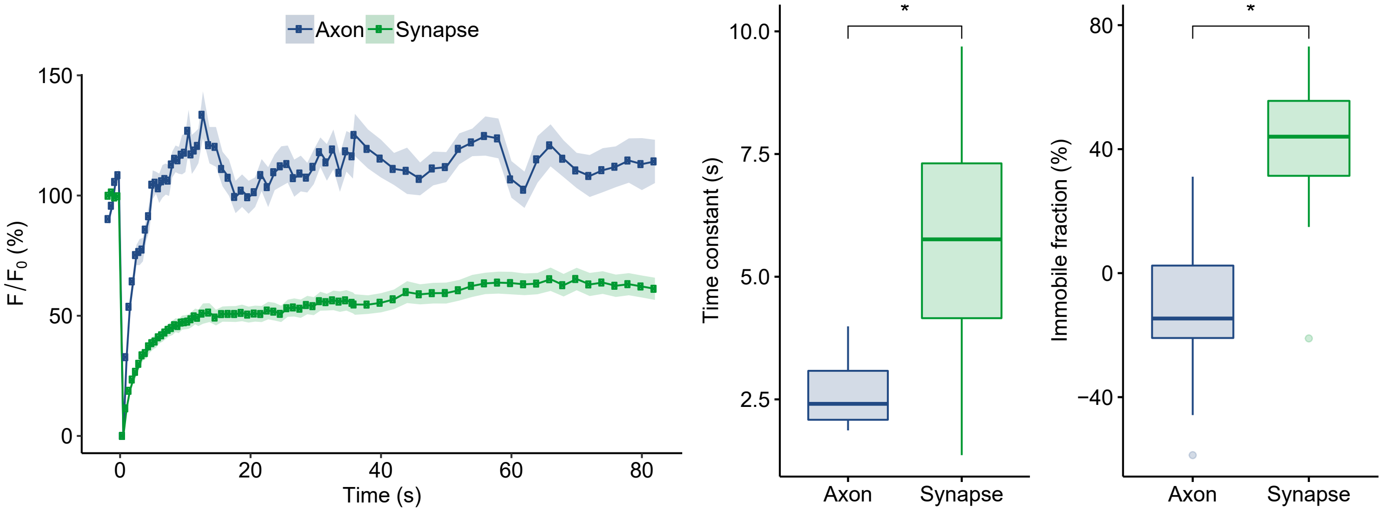

N (axons) = 12, N (synapses) = 25; p (time constant) = 5.50E-04, p (immobile fraction) = 1.28E-05.

Tagged protein outline:

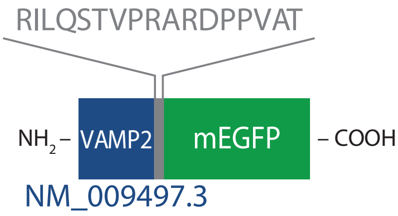

Time constant (axon) is significantly different from time constant (axon) of: Munc13 (p = 4.64E-02).
Immobile fraction (axon) is significantly different from immobile fraction (axon) of: membrane mEGFP (p = 4.07E-02), Syndapin 1 (p = 4.91E-02).
Time constant (synapse) is significantly different from time constant (synapse) of: Hsc70 (p = 1.71E-02), mEGFP (p = 2.58E-06), SCAMP1 (p = 3.73E-02), Synapsin 1A (p = 8.19E-04), Synaptogyrin (p = 1.08E-04), Synaptophysin (p = 2.76E-06), Synaptotagmin 1 (p = 2.74E-07), Syntaxin 16 (p = 4.85E-03), VAMP1 (p = 1.16E-04), vGluT1 (p = 3.23E-06).
Immobile fraction (synapse) is significantly different from immobile fraction (synapse) of: alpha-SNAP (p = 3.23E-03), AP180 (p = 3.40E-04), Complexin 2 (p = 9.68E-03), mEGFP (p = 1.51E-04), Munc13 (p = 1.01E-02), Munc18 (p = 7.20E-05), Rab3a (p = 2.18E-02), Rab5a (p = 9.24E-06), Rab7a (p = 1.33E-03), Munc13 (p = 1.01E-02), SNAP29 (p = 2.47E-03), SV2B (p = 3.74E-04), Synaptophysin (p = 3.03E-04), Synaptotagmin 7 (p = 1.16E-02), Syndapin 1 (p = 1.73E-02), Syntaxin 1A (p = 2.02E-02).

References

Baumert, M., et al. (1989). J Cell Biol 110, 1285-94.
Takamori, S., et al. (2006). Cell 127, 831-46.

VAMP4

MW (kDa)	Category	τ_{axon} (s)	$\tau_{synapse}$ (s)	IF_{axon} (%)	$IF_{synapse}$ (%)
16.35	endosomal	14.74 ± 3.91	4.71 ± 0.47	1.61 ± 16.31	51.38 ± 5.68

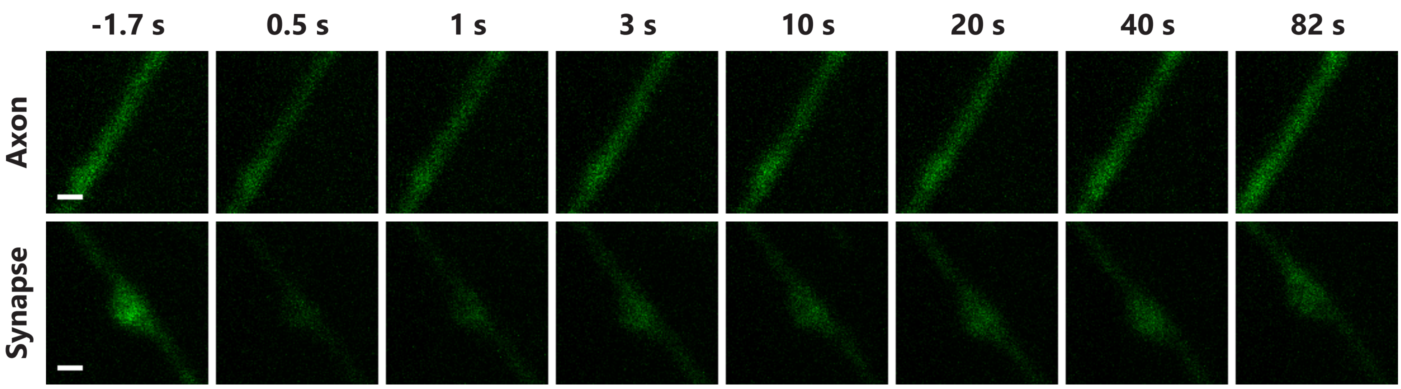

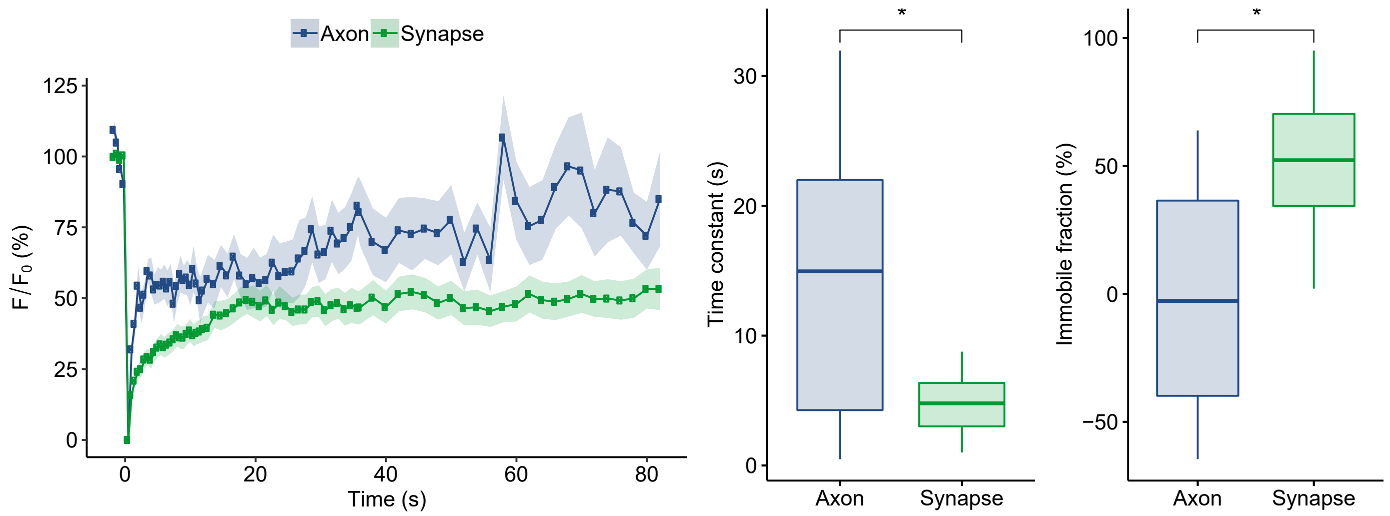

N (axons) = 9, N (synapses) = 24; p (time constant) = 4.99E-02, p (immobile fraction) = 1.03E-02.

Tagged protein outline:

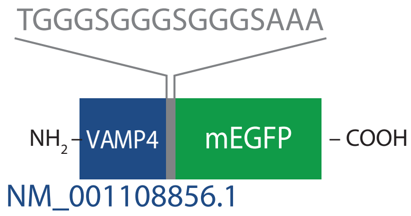

Time constant (axon) is not significantly different from time constant (axon) of any other proteins.
Immobile fraction (axon) is not significantly different from immobile fraction (axon) of any other proteins.
Time constant (synapse) is significantly different from time constant (synapse) of: mEGFP (p = 7.08E-04), SCAMP1 (p = 7.09E-03), Synapsin 1A (p = 3.30E-04), Synaptogyrin (p = 6.11E-05), Synaptophysin (p = 1.83E-06), Synaptotagmin 1 (p = 3.29E-07), Syntaxin 16 (p = 6.27E-04), alpha-Tubulin 1b (p = 1.32E-02), VAMP1 (p = 2.80E-05), vGluT1 (p = 3.29E-06), Vti1a-beta (p = 4.91E-02).
Immobile fraction (synapse) is significantly different from immobile fraction (synapse) of: alpha-SNAP (p = 2.25E-03), AP180 (p = 6.80E-04), AP2 (p = 4.17E-02), Complexin 2 (p = 2.79E-02), Doc2a (p = 3.60E-02), Endophilin A1 (p = 3.02E-02), mEGFP (p = 1.73E-03), Munc13 (p = 1.02E-03), Munc18 (p = 9.24E-04), Rab3a (p = 5.05E-03), Rab5a (p = 7.42E-05), Rab7a (p = 1.21E-03), Munc13 (p = 1.02E-03), SNAP29 (p = 1.46E-03), Synaptotagmin 7 (p = 5.50E-03), Syndapin 1 (p = 1.82E-02), Syntaxin 1A (p = 1.22E-02).

References

Steegmaier, M., et al. (1999). Mol Biol Cell 10, 1957-72.
Mallard, F., et al. (2002). J Cell Biol 156, 653-64.
Raingo, J., et al. (2012). Nat Neurosci 15, 738-45.
Takamori, S., et al. (2006). Cell 127, 831-46.

vATPase

MW (kDa)	Category	τ_{axon} (s)	$\tau_{synapse}$ (s)	IF_{axon} (%)	$IF_{synapse}$ (%)
95.66	vesicular	3.13 ± 0.71	13.56 ± 1.99	20.97 ± 5.90	54.99 ± 4.56

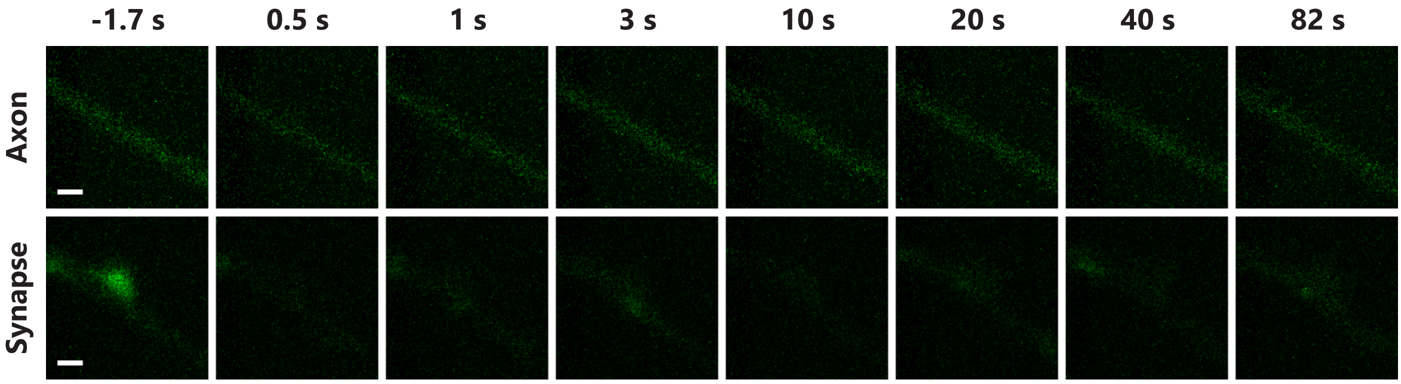

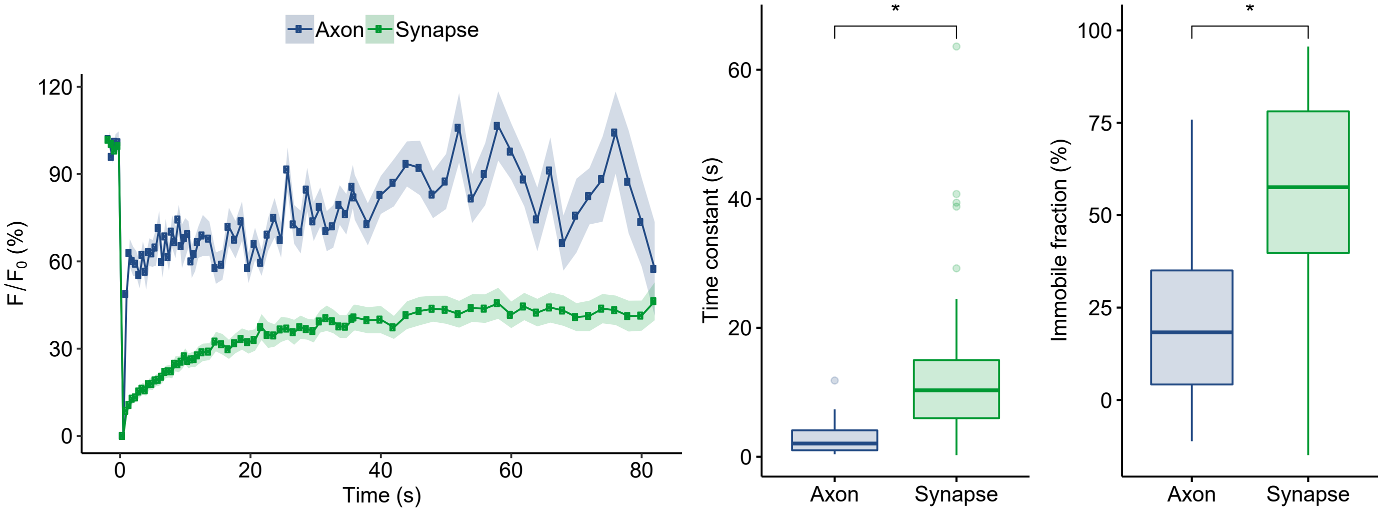

N (axons) = 18, N (synapses) = 42; p (time constant) = 1.98E-05, p (immobile fraction) = 1.28E-04.

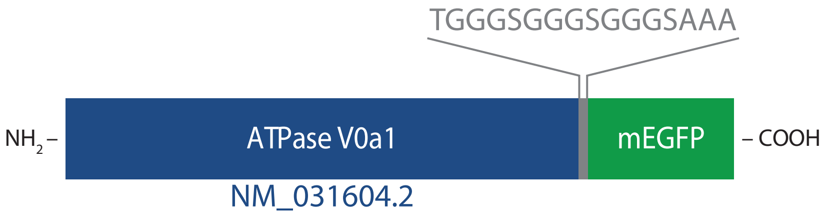

Time constant (axon) is not significantly different from time constant (axon) of any other proteins.
Immobile fraction (axon) is not significantly different from immobile fraction (axon) of any other proteins.
Time constant (synapse) is significantly different from time constant (synapse) of: AP180 (p = 2.44E-02), AP2 (p = 6.28E-03), Calmodulin 1 (p = 1.96E-03), Clathrin light chain B (p = 2.62E-03), Complexin 1 (p = 6.55E-03), Endophilin A1 (p = 4.01E-02), Hsc70 (p = 5.56E-05), mEGFP (p = 3.66E-08), Munc13 (p = 9.04E-04), Munc18 (p = 5.85E-04), NSF (p = 1.49E-02), Rab3a (p = 1.62E-02), Rab5a (p = 7.59E-04), Rab7a (p = 1.53E-02), Septin 5 (p = 1.99E-02), SNAP23 (p = 2.01E-02), SNAP25 (p = 7.65E-04), Synaptogyrin (p = 1.58E-02).
Immobile fraction (synapse) is significantly different from immobile fraction (synapse) of: Actin (p = 4.02E-02), alpha-SNAP (p = 7.38E-05), AP180 (p = 5.64E-06), AP2 (p = 4.23E-04), Calmodulin 1 (p = 1.37E-02), Clathrin light chain B (p = 4.20E-02), Complexin 1 (p = 4.99E-03), Complexin 2 (p = 5.79E-04), Doc2a (p = 8.52E-04), Endophilin A1 (p = 6.16E-04), Epsin (p = 2.98E-02), Hsc70 (p = 2.10E-03), mEGFP (p = 9.46E-07), Munc13 (p = 4.95E-06), Munc18 (p = 1.89E-07), PIP5KI-gamma (p = 9.68E-03), Rab3a (p = 2.83E-04), Rab5a (p = 3.70E-07), Rab7a (p = 1.75E-05), Septin 5 (p = 7.34E-03), SNAP23 (p = 3.05E-02), Munc13 (p = 4.95E-06), SNAP29 (p = 6.41E-05), Synaptotagmin 7 (p = 9.62E-05), Syndapin 1 (p = 2.77E-04), Syntaxin 1A (p = 2.54E-04), Syntaxin 16 (p = 1.65E-03).

References

Perin, M.S., et al. (1991). J Biol Chem 266, 3877-81.
Takamori, S., et al. (2006). Cell 127, 831-46.
Oot, R.A., et al. (2012). Structure 20, 1881-92.
Kitagawa, N., et al. (2008). J Biol Chem 283, 3329-37.

vGluT1

MW (kDa)	Category	τ_{axon} (s)	$\tau_{synapse}$ (s)	IF_{axon} (%)	$IF_{synapse}$ (%)
61.66	vesicular	-	23.53 ± 3.20	-	63.24 ± 4.86

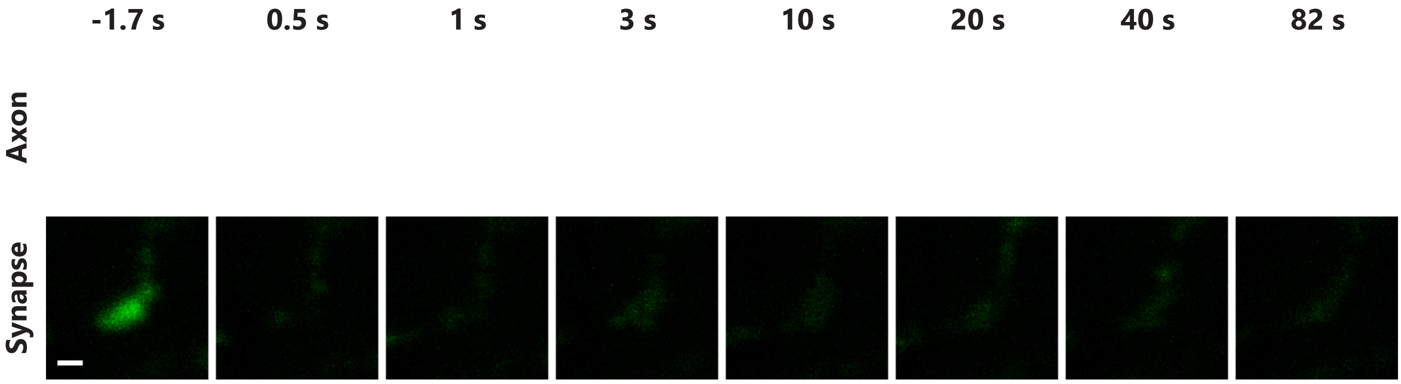

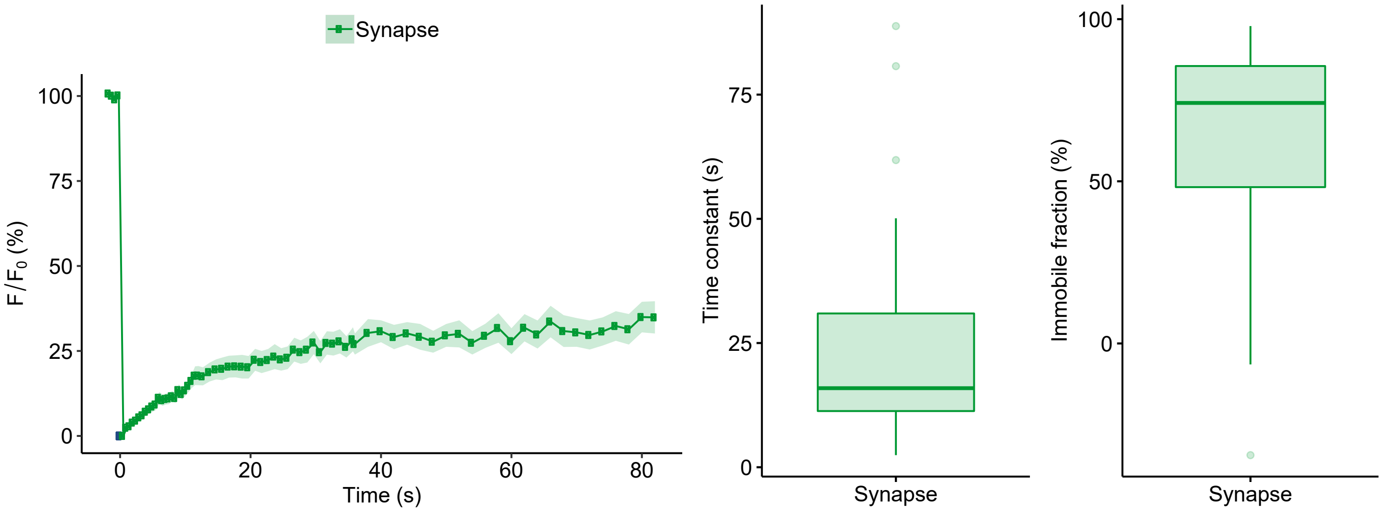

N (synapses) = 38.

Tagged protein outline:

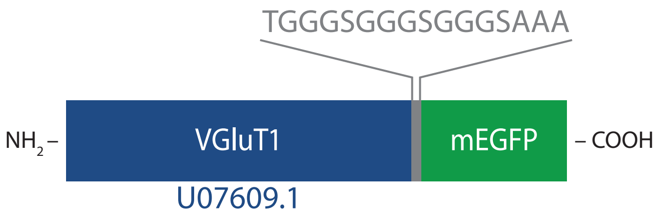

Time constant (synapse) is significantly different from time constant (synapse) of: Actin (p = 6.74E-05), alpha-SNAP (p = 5.86E-06), alpha-synuclein (p = 2.02E-05), Amphiphysin (p = 6.32E-06), AP180 (p = 3.07E-08), AP2 (p = 5.50E-08), Calmodulin 1 (p = 4.23E-08), Clathrin light chain B (p = 2.09E-08), Complexin 1 (p = 4.48E-08), Complexin 2 (p = 8.22E-07), CSP (p = 4.34E-03), Doc2a (p = 1.67E-06), Endophilin A1 (p = 1.42E-07), Epsin (p = 8.87E-04), Hsc70 (p = 3.36E-10), mEGFP (p = 9.06E-14), membrane mEGFP (p = 3.98E-07), Munc13 (p = 3.44E-10), Munc18 (p = 4.59E-12), NSF (p = 4.12E-07), PIP5KI-gamma (p = 4.85E-04), Rab3a (p = 7.59E-08), Rab5a (p = 2.45E-10), Rab7a (p = 1.30E-07), Septin 5 (p = 2.10E-08), SNAP23 (p = 5.44E-07), SNAP25 (p = 2.19E-11), SNAP29 (p = 9.68E-07), Synaptotagmin 7 (p = 2.47E-06), Syndapin 1 (p = 5.53E-07), Syntaxin 1A (p = 1.52E-05), VAMP2 (p = 3.23E-06), VAMP4 (p = 3.29E-06),.Immobile fraction (synapse) is significantly different from immobile fraction (synapse) of: Actin (p = 1.54E-03), alpha-SNAP (p = 1.35E-05), alpha-synuclein (p = 1.69E-03), Amphiphysin (p = 2.46E-03), AP180 (p = 7.37E-07), AP2 (p = 6.70E-05), Calmodulin 1 (p = 5.13E-04), Clathrin light chain B (p = 1.13E-03), Complexin 1 (p = 9.48E-05), Complexin 2 (p = 4.02E-05), Doc2a (p = 2.37E-05), Endophilin A1 (p = 3.73E-05), Epsin (p = 2.68E-03), Hsc70 (p = 3.28E-05), mEGFP (p = 7.67E-09), membrane mEGFP (p = 3.44E-03), Munc13 (p = 5.82E-07), Munc18 (p = 2.15E-09), PIP5KI-gamma (p = 1.28E-03), Rab3a (p = 9.77E-06), Rab5a (p = 2.10E-08), Rab7a (p = 4.09E-06), Septin 5 (p = 2.36E-04), SNAP23 (p = 8.84E-04), Munc13 (p = 5.82E-07), SNAP29 (p = 7.63E-06), Synaptotagmin 7 (p = 7.23E-06), Syndapin 1 (p = 1.42E-05), Syntaxin 1A (p = 2.39E-05), Syntaxin 16 (p = 4.59E-05), VAMP1 (p = 1.48E-02),

References

Bellocchio, F., et al. (2000). Science 289, 957-60.
Takamori, S., et al. (2006). Cell 127, 831-46.

Vti1a-β

MW (kDa)	Category	τ_{axon} (s)	$\tau_{synapse}$ (s)	IF_{axon} (%)	$IF_{synapse}$ (%)
26.04	endosomal	4.53 ± 0.95	21.24 ± 4.33	11.82 ± 8.78	38.28 ± 7.12

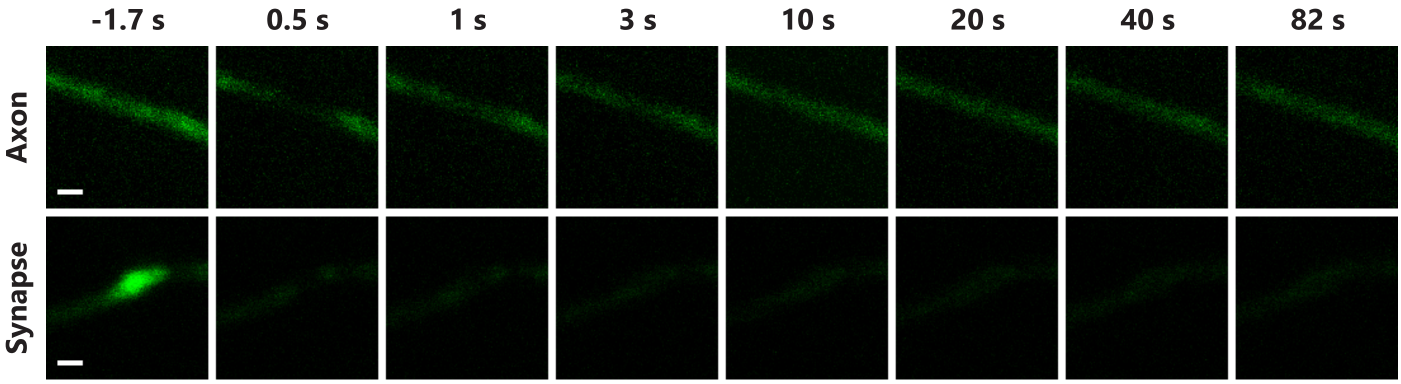

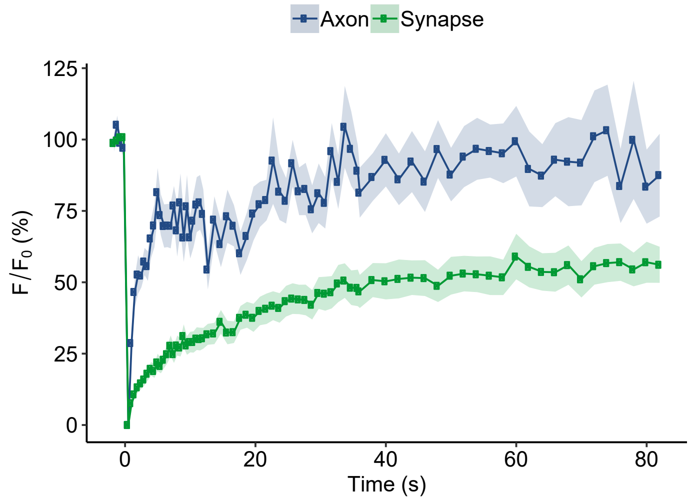

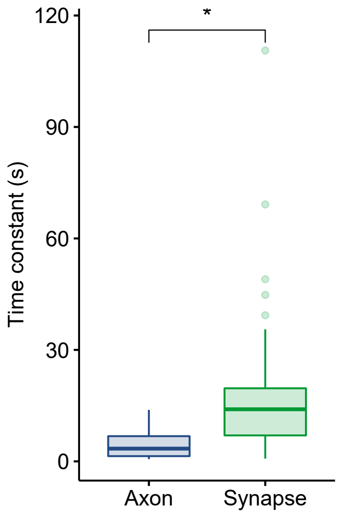

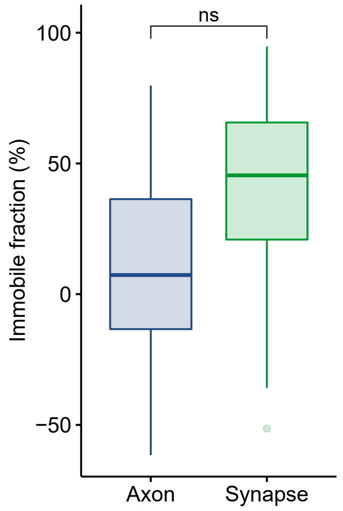

N (axons) = 18, N (synapses) = 29; p (time constant) = 1.03E-04.

Tagged protein outline:

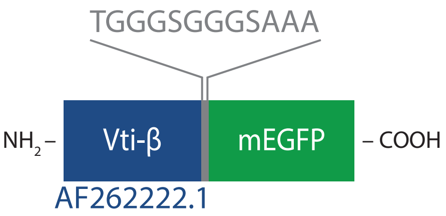

Time constant (axon) is not significantly different from time constant (axon) of any other proteins.
Immobile fraction (axon) is not significantly different from immobile fraction (axon) of any other proteins.
Time constant (synapse) is significantly different from time constant (synapse) of: , AP180 (p = 4.68E-03), AP2 (p = 1.78E-03), Calmodulin 1 (p = 5.24E-04), Clathrin light chain B (p = 8.35E-04), Complexin 1 (p = 1.20E-03), Complexin 2 (p = 2.87E-02), Doc2a (p = 4.45E-02), Endophilin A1 (p = 9.68E-03), Hsc70 (p = 3.39E-05), mEGFP (p = 8.41E-08), membrane mEGFP (p = 2.09E-02), Munc13 (p = 3.07E-04), Munc18 (p = 1.22E-04), NSF (p = 3.58E-03), Rab3a (p = 3.67E-03), Rab5a (p = 1.80E-04), Rab7a (p = 3.61E-03), Septin 5 (p = 1.93E-03), SNAP23 (p = 4.64E-03), SNAP25 (p = 9.96E-05), Syndapin 1 (p = 4.84E-02), VAMP4 (p = 4.91E-02).
Immobile fraction (synapse) is significantly different from immobile fraction (synapse) of: , Rab5a (p = 2.58E-02), SV2B (p = 2.40E-03), Synaptophysin (p = 3.66E-02).

References

Mallard, F., et al. (2002). J Cell Biol 156, 653-64.
Kreykenbohm, V., et al. (2002). Eur J Cell Biol 81, 273-80.
Takamori, S., et al. (2006). Cell 127, 831-846.

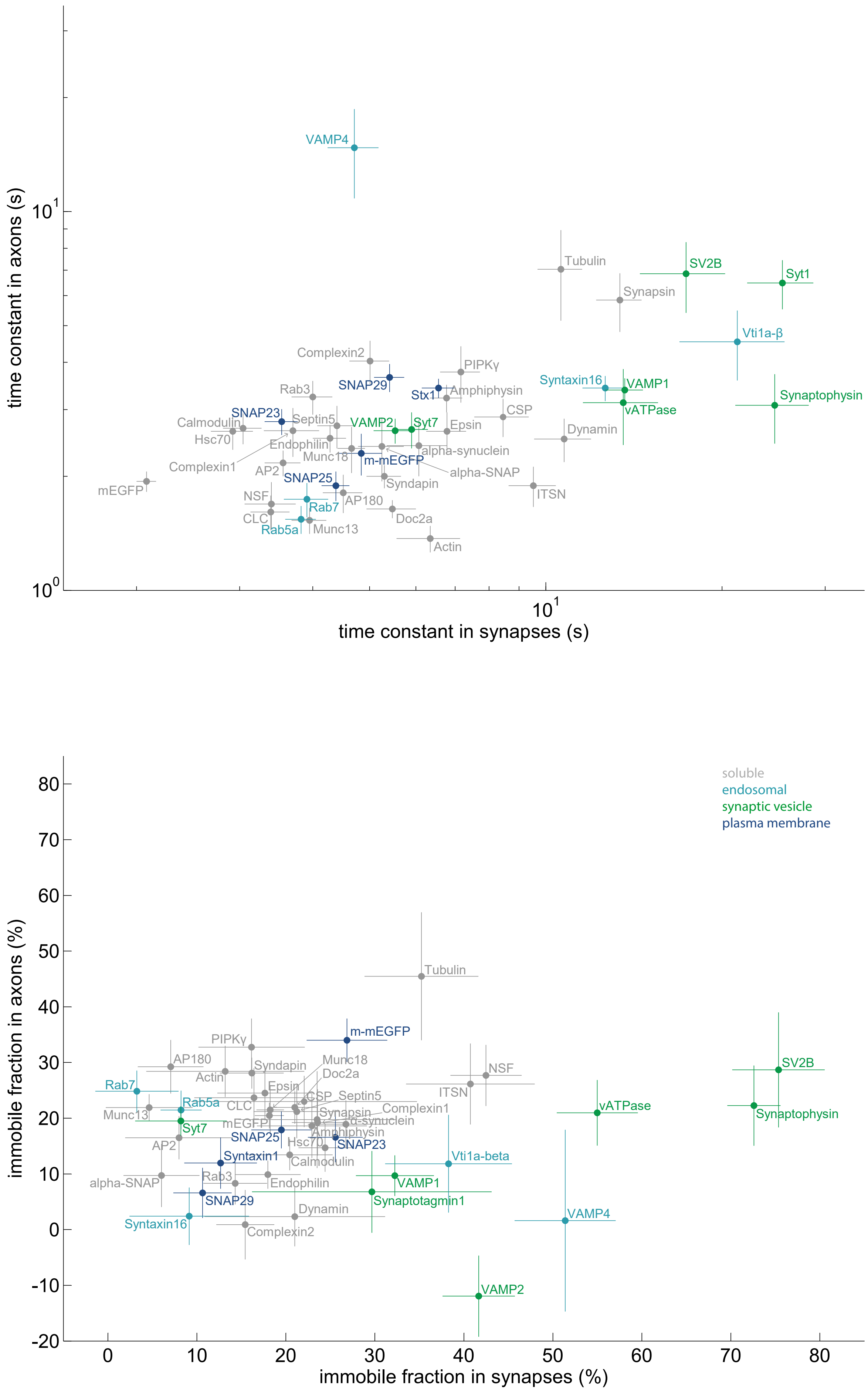

Appendix Fig. S4. Time constants and immobile fractions of analyzed proteins in axons and synapses. Symbols indicate means ± SEM; all data are shown as box plots in Appendix Fig. S3.

Multi-page figure

protein	axon		synapse	
	correlation coefficient	P value	correlation coefficient	P value
Actin	0.248	0.232	0.1558	0.4285
alpha-SNAP	0.1965	0.4657	0.082	0.6842
alpha-synuclein	0.1356	0.6298	0.3254	0.0604
Amphiphysin	0.0184	0.9321	0.2103	0.2116
AP180	0.2327	0.2335	0.1434	0.4112
AP2	0.1697	0.3789	0.1729	0.361
Calmodulin	0.1152	0.5445	0.1848	0.3284
Clathrin light chain	0.0407	0.8503	0.1165	0.5187
Complexin1	0.0214	0.9327	0.2452	0.2084
Complexin2	0.0511	0.7887	0.2544	0.175
CSP	0.1384	0.5003	0.2334	0.2958
Doc2a	0.0563	0.8136	0.5967	0.0055
Dynamin	0.2943	0.307	0.1019	0.628
Endophilin	0.3345	0.0708	0.1974	0.2957
Epsin	0.1276	0.5918	0.4722	0.0355
Hsc70	0.1229	0.5102	0.0099	0.9522
Intersectin 1-L	0.1207	0.6122	0.0359	0.848
mEGFP	0.0472	0.815	0.3224	0.0069
membrane mEGFP	0.4647	0.0255	0.3038	0.116
Munc13	0.26	0.1732	0.1578	0.4224
Munc18	0.2849	0.2107	0.1608	0.1569
NSF	0.0576	0.8383	0.1176	0.5754
PIP5KIgamma	0.0579	0.8441	0.0869	0.6729
Rab3a	0.3085	0.0972	0.1938	0.2963

protein	axon		synapse	
	correlation coefficient	P value	correlation coefficient	P value
Rab5a	0.123	0.5953	0.1128	0.4554
Rab7a	0.2886	0.122	0.149	0.4319
SCAMP1	n/a		0.2141	0.3388
Septin5	0.0643	0.7221	0.0474	0.7775
SNAP23	0.2536	0.2317	0.1341	0.5229
SNAP25	0.1183	0.6512	0.1965	0.1055
SNAP29	0.0932	0.6576	0.0565	0.7709
SV2B	0.2168	0.3874	0.1606	0.4534
Synapsin	0.0505	0.8475	0.075	0.7158
Synaptogyrin	n/a		0.5142	0.0722
Synaptophysin	0.4044	0.2173	0.1048	0.7334
Synaptotagmin1	0.3878	0.1119	0.3719	0.0331
Synaptotagmin7	0.2132	0.2761	0.0805	0.6781
Syndapin	0.1585	0.3944	0.4153	0.0202
Syntaxin1	0.2573	0.3189	0.479	0.0099
Syntaxin16	0.0758	0.7072	0.1993	0.2999
Tubulin	0.2352	0.5423	0.1208	0.6441
VAMP1	0.1877	0.3911	0.3558	0.0582
VAMP2	0.3157	0.2715	0.1144	0.5621
VAMP4	0.1008	0.7681	0.0888	0.68
vATPase	0.1657	0.4979	0.1831	0.3242
vGluT	n/a		0.0606	0.8369
Vti1a-beta	0.129	0.6216	0.1206	0.6227

Appendix Fig. S5. An analysis of the correlation between overexpression levels and mobility rates. Protein overexpression was quantified as the fluorescence intensity in the analyzed regions, and was compared with the time constants obtained from the respective regions. This page: table showing correlation coefficients and P values of all analyzed proteins. SCAMP1, Synaptogyrin, and vGluT were not detected in axons and therefore no time constants were obtained for these proteins in axons. No correlation was significant, when corrected for multiple testing (Benjamini-Hochberg procedure, with a false discovery rate as high as 0.2, meaning 20%). Following pages: scatter plots showing the relation between fluorescence intensity and time constants of all proteins. Each point represents one synapse/axonal region.

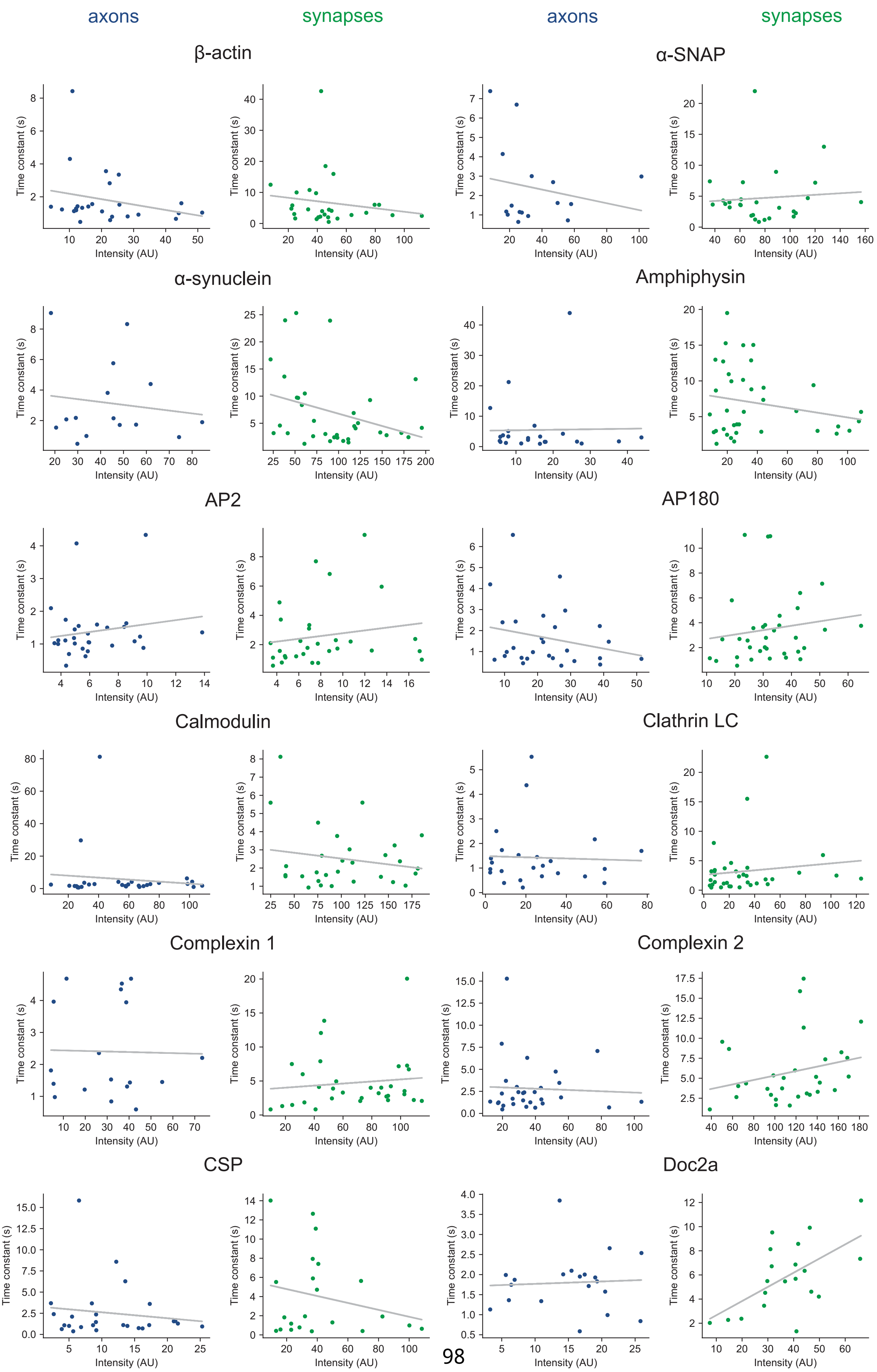
axons
synapses
axons
synapses
β-actin
α-SNAP
α-synuclein
Amphiphysin
AP2
AP180
Calmodulin
Clathrin LC
Complexin 1
Complexin 2
CSP
Doc2a
Time constant (s)
Intensity (AU)

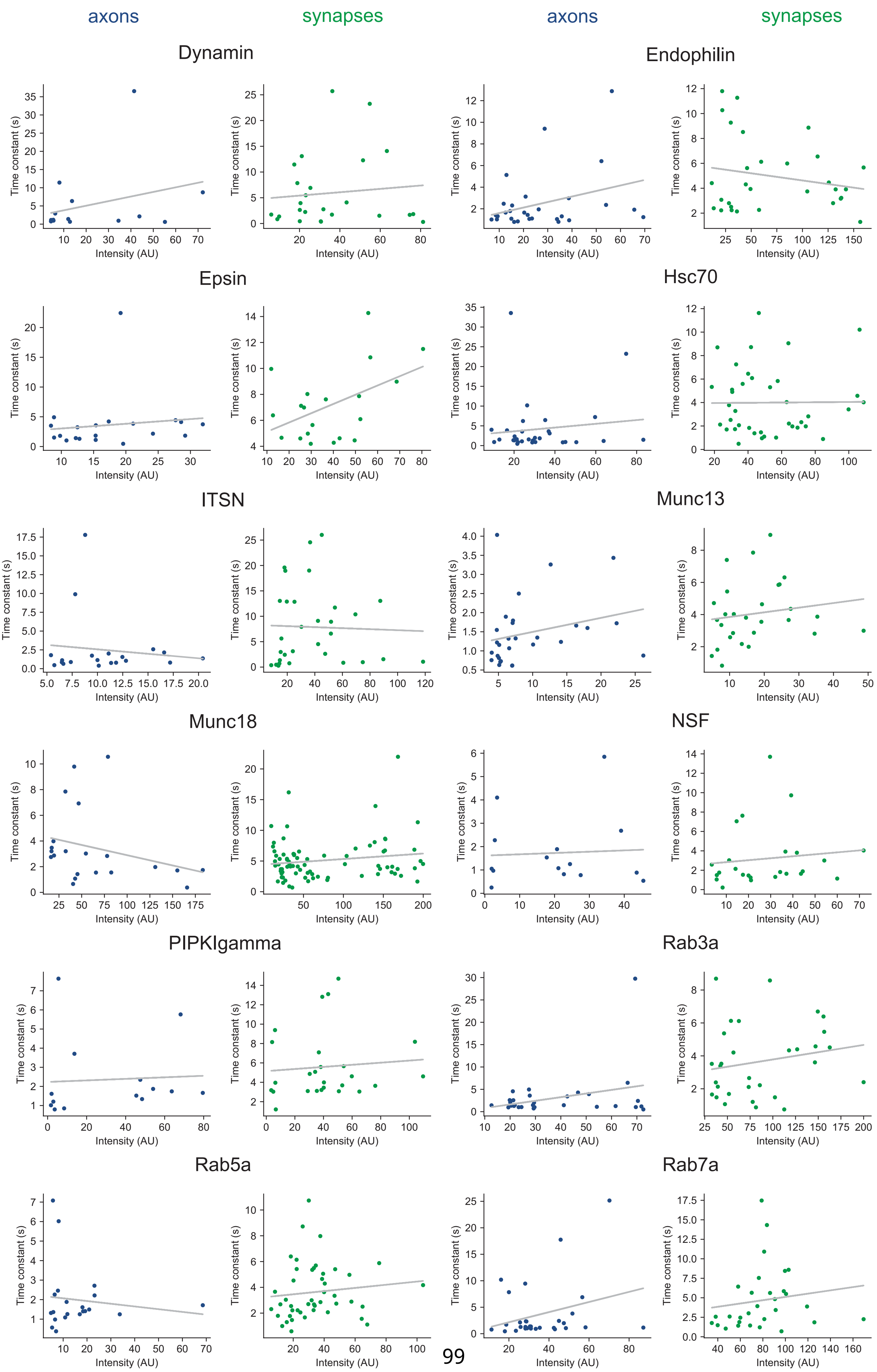
axons
synapses
axons
synapses
Dynamin
Endophilin
Epsin
Hsc70
ITSN
Munc13
Munc18
NSF
PIPKIgamma
Rab3a
Rab5a
Rab7a
Time constant (s)
Intensity (AU)

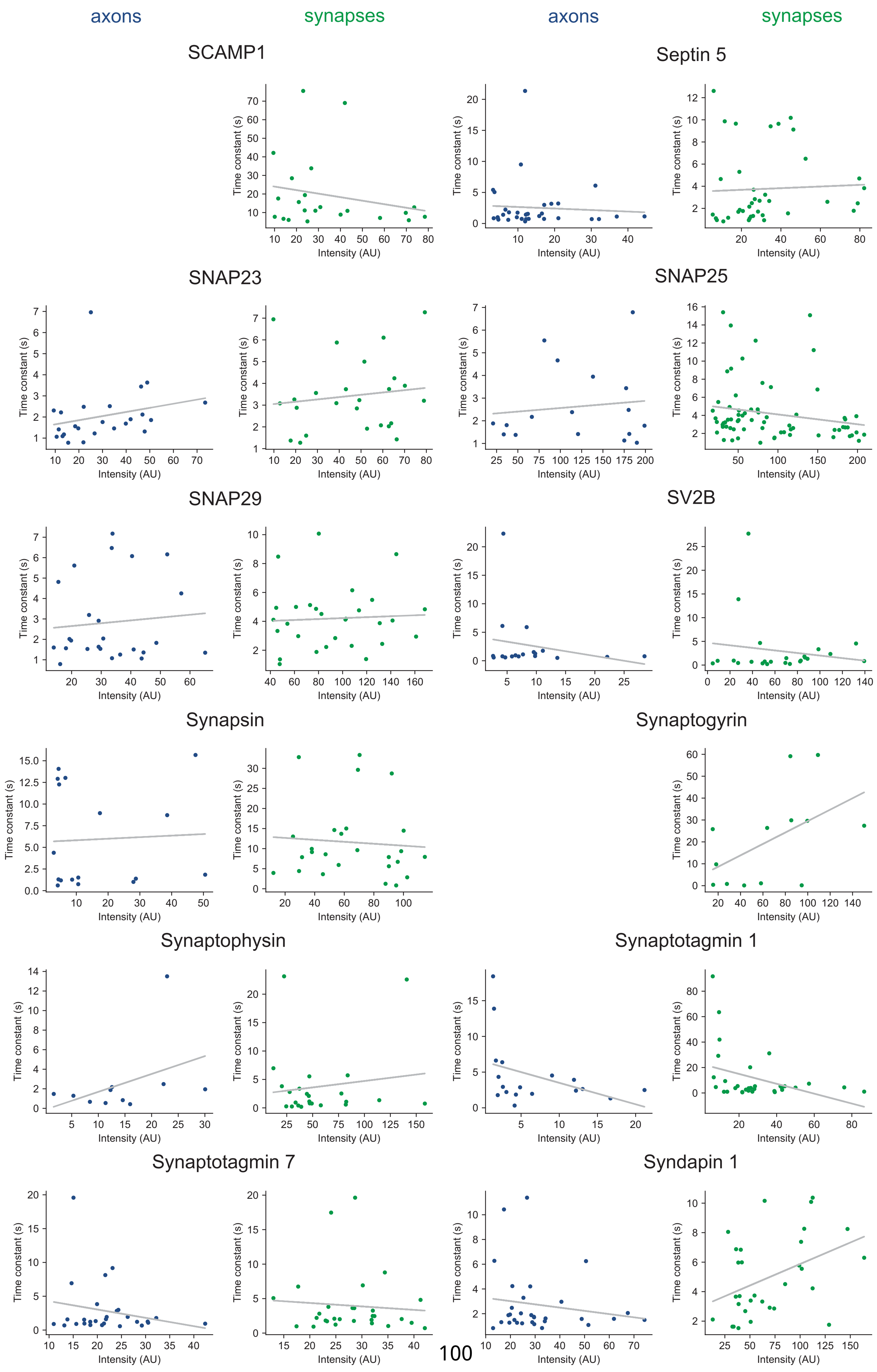
axons
synapses
axons
synapses
SCAMP1
Septin 5
SNAP23
SNAP25
SNAP29
SV2B
Synapsin
Synaptogyrin
Synaptophysin
Synaptotagmin 1
Synaptotagmin 7
Syndapin 1
Time constant (s)
Intensity (AU)

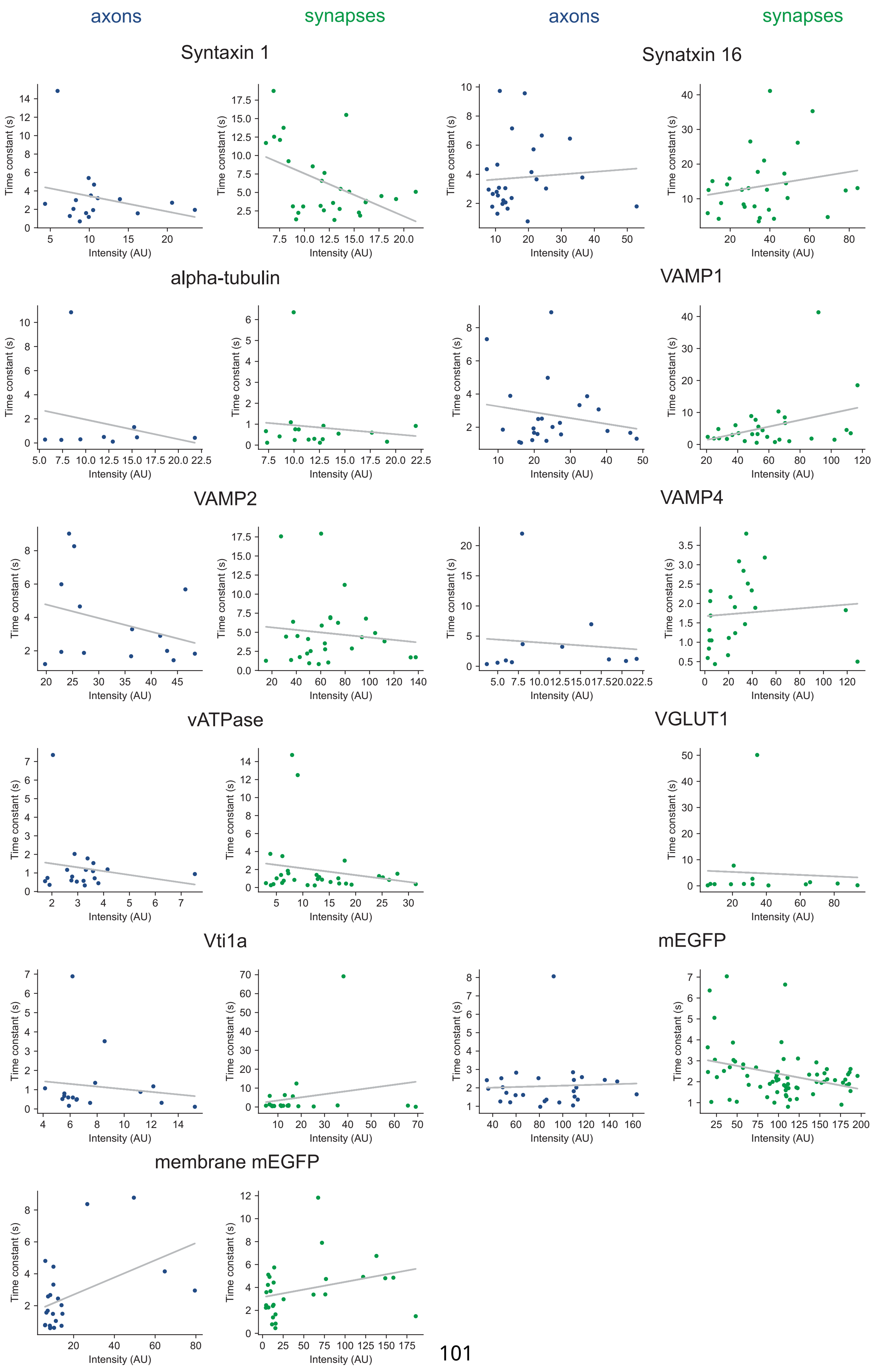

axons
synapses
axons
synapses
Syntaxin 1
Synatxin 16
alpha-tubulin
VAMP1
VAMP2
VAMP4
vATPase
VGLUT1
Vti1a
mEGFP
membrane mEGFP
Time constant (s)
Intensity (AU)

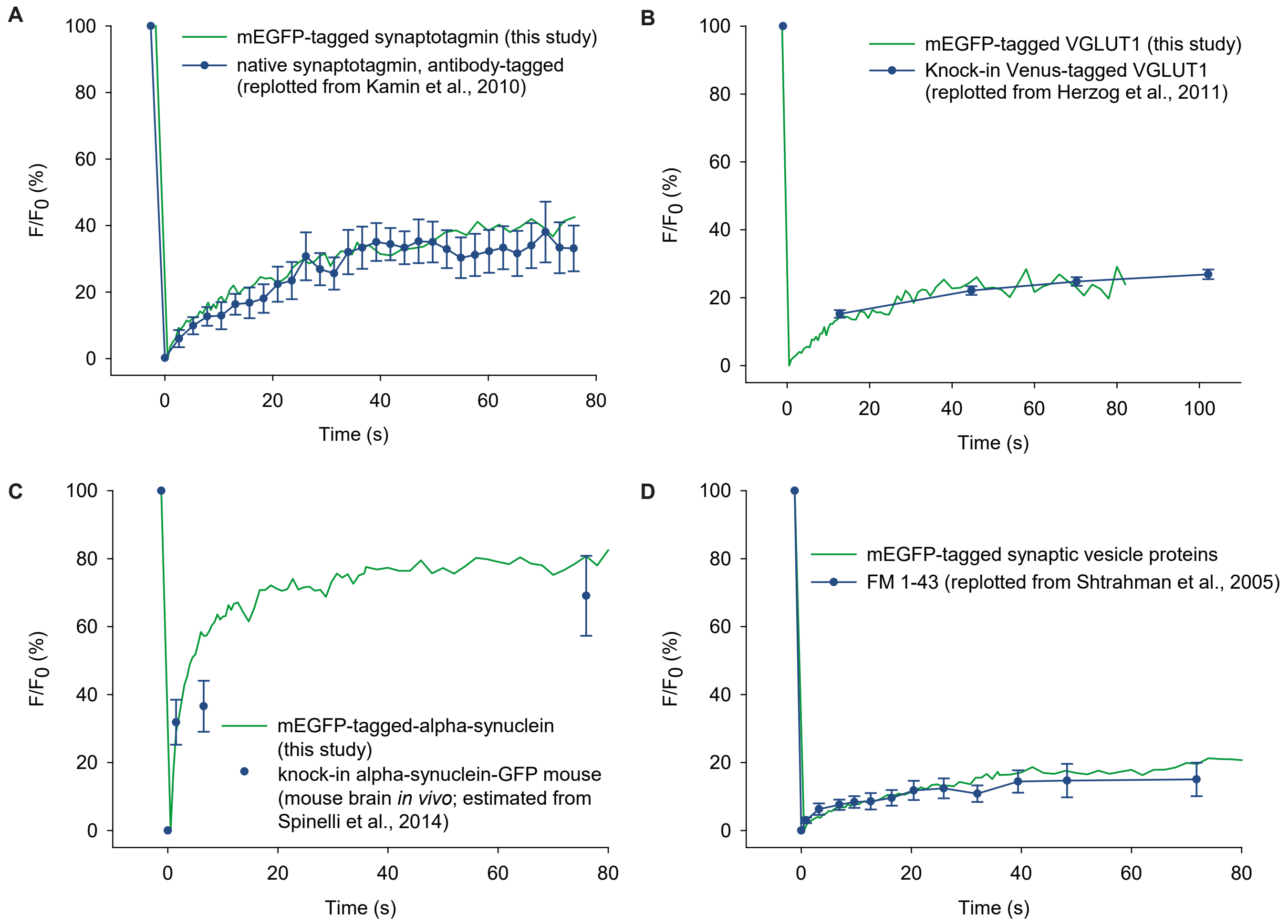

Appendix Fig. S6. Our FRAP results reproduce previous results obtained on knock-in tagged proteins or on endogenous proteins labeled with fluorescent tags. A, We compared our results with previously published FRAP experiments performed on endogenous synaptotagmin 1 visualized by the use of antibodies (Kamin et al., 2010). **B**, A similar comparison to VGLUT1, measured in cultures from mice tagged in-locus with a fluorescent version of the protein (Herzog et al., 2011). No intensity of VGLUT1Venus is shown at 0 s, since the first measurement came at ~12 seconds in the original publication. **C**, We obtained similar results to the ones measured in live brains of knock-in Syn-GFP mice (minimal expression, mimicking human disease; estimated from Spinelli et al., 2014); **D**, The average FRAP curve of several SV proteins (relying on the most vesicle-enriched proteins, as known from the literature: Synaptogyrin, Synaptophysin, SV2, VGLUT) measured here overlaps closely with the recovery of FM 1-43 labeled synaptic vesicles (measured in Shtrahman et al., 2005). All green traces indicate the median values of the respective FRAP distributions from our measurements, shown here without error bars, to avoid obscuring the other measurements shown. -The errors represent the error measurements from the original publications.

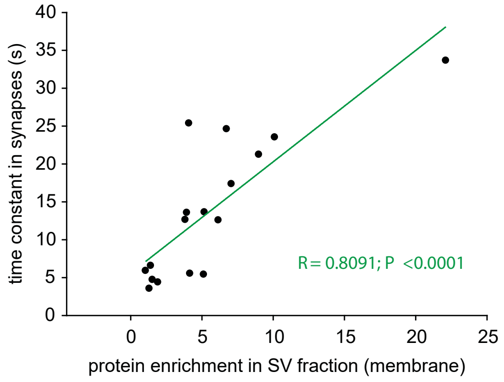

Appendix Fig. S7. Protein mobility is affected by association with synaptic vesicles. We compared the time constants in synapses to the enrichment of the proteins in synaptic vesicles, obtained from biochemical experiments (Takamori et al., 2006). Significant correlations were observed for membrane proteins.

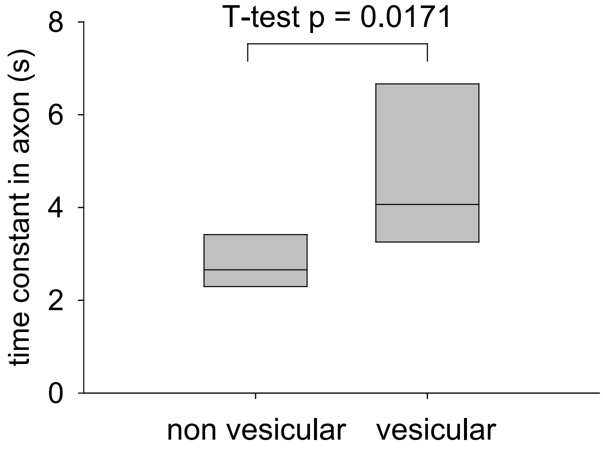

Appendix Fig. S8. Comparison of the axonal time constants of vesicular and non-vesicular proteins. True vesicular proteins move significantly slower in the axonal plasma membrane compared to other membrane proteins.

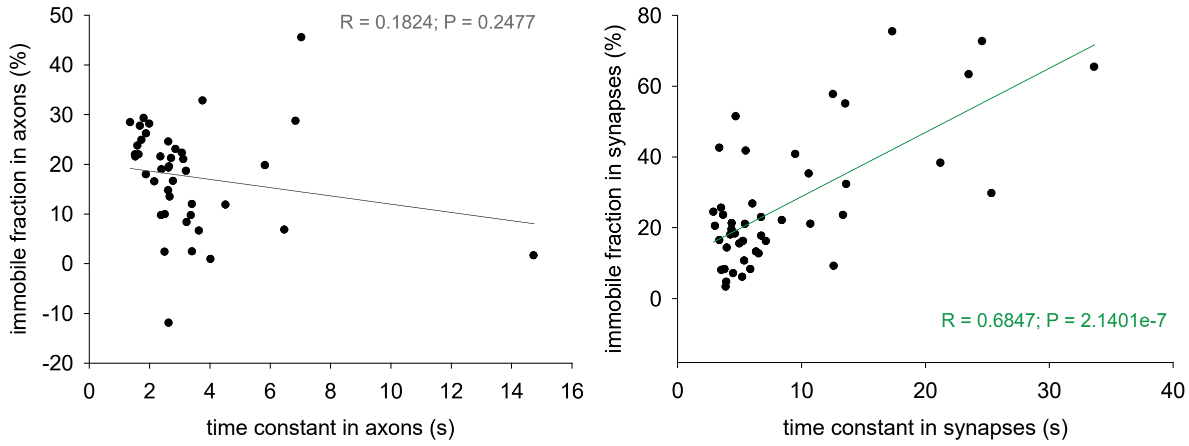

Appendix Fig. S9. Correlation between the time constants and the immobile fractions in axons (left) and synapses (right). A significant correlation is observed in synapses, but not in axons. Similar results were observed when analyzing the soluble or membrane proteins separately.

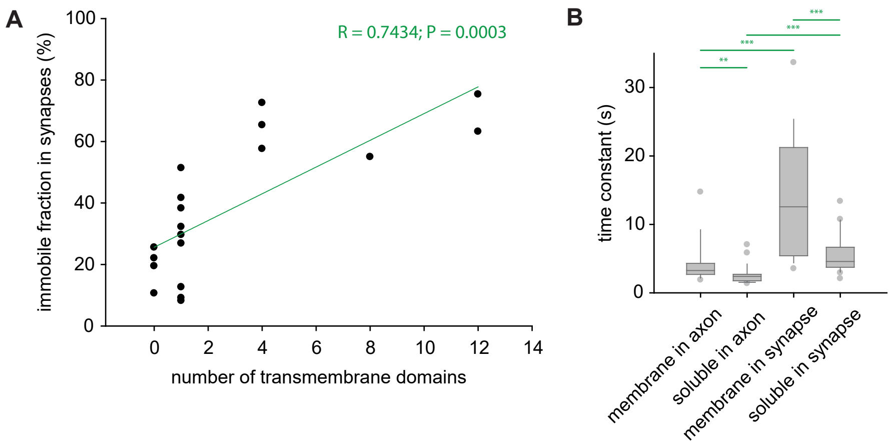

Appendix Fig. S10. An analysis of basic movement parameters of the proteins. A, Correlation of synaptic immobile fractions with the number of transmembrane domains, for the different membrane proteins. Significant correlation can be observed, which agrees with the previous literature, and with the expectation that proteins with large numbers of membrane domains diffuse more slowly. Similar trends were observed for the axonal measurements. **B**, A comparison of time constants in different compartments shows differences between soluble and membrane proteins, as well as differences between axons and synapse for both protein categories. Wilcoxon rank-sum test, ** P < 0.01, *** P < 0.001.

correlation coefficients (all proteins)

	%Ala	%Arg	%Asn	%Asp	%Cys	%Gln	%Glu	%Gly	%His	%Ile	%Leu	%Lys	%Met	%Phe	%Pro	%Ser	%Thr	%Trp	%Tyr	%Val
time constant (axon)	-0.2315	0.2361	-0.021	0.1318	-0.0858	-0.0959	-0.058	-0.0662	0.173	0.1588	0.1211	0.0165	0.0389	0.1638	-0.0407	-0.0305	-0.1738	0.0392	-0.1676	0.0711
immobile fraction (axon)	0.1919	-0.3364	-0.0339	-0.2722	0.2314	-0.307	-0.2546	0.3133	0.1215	-0.145	-0.1037	-0.3514	-0.2161	0.2179	0.2263	0.1955	0.2606	0.333	0.3387	0.0479
time constant (synapse)	0.0147	-0.1917	-0.0449	-0.4928	0.0444	-0.0803	-0.3212	0.2717	0.1994	-0.1362	0.1738	-0.3449	-0.1316	0.4891	0.1386	0.0702	-0.113	0.3353	0.4329	0.2829
immobile fraction (synapse)	-0.0571	-0.1539	0.0391	-0.3972	0.0411	-0.1205	-0.2769	0.3473	0.0895	0.0918	0.1223	-0.3725	0.1089	0.5618	0.058	-0.1224	-0.1717	0.4274	0.2278	0.196

P values (all proteins)

	%Ala	%Arg	%Asn	%Asp	%Cys	%Gln	%Glu	%Gly	%His	%Ile	%Leu	%Lys	%Met	%Phe	%Pro	%Ser	%Thr	%Trp	%Tyr	%Val
time constant (axon)	0.1402	0.1322	0.8951	0.4054	0.5891	0.5456	0.715	0.6772	0.2733	0.3152	0.445	0.9172	0.8066	0.2999	0.798	0.848	0.2709	0.8055	0.2889	0.6545
immobile fraction (axon)	0.2293	0.0315	0.8332	0.0852	0.1455	0.0509	0.1082	0.0461	0.4491	0.3656	0.5188	0.0243	0.1747	0.1711	0.1548	0.2207	0.0999	0.0334	0.0303	0.766
time constant (synapse)	0.9237	0.2071	0.7694	5.84E-04	0.7719	0.6001	0.0315	0.071	0.189	0.3722	0.2537	0.0203	0.3888	6.51E-04	0.3639	0.6468	0.4598	0.0243	2.97E-03	0.0597
immobile fraction (synapse)	0.7095	0.3128	0.799	6.90E-03	0.7886	0.4304	0.0655	0.0194	0.5586	0.5488	0.4235	0.0117	0.4766	5.92E-05	0.705	0.4232	0.2595	3.41E-03	0.1323	0.1969

correlation coefficients (soluble proteins)

	%Ala	%Arg	%Asn	%Asp	%Cys	%Gln	%Glu	%Gly	%His	%Ile	%Leu	%Lys	%Met	%Phe	%Pro	%Ser	%Thr	%Trp	%Tyr	%Val
time constant (axon)	-0.0258	0.0342	-0.2812	-0.0371	0.0236	0.161	0.0179	0.2308	0.2455	-0.1125	-0.1977	-0.1169	-0.0688	-0.0428	0.1963	-0.0462	0.0201	-0.2626	0.0994	-2.96E-03
immobile fraction (axon)	0.0919	-0.142	0.0506	-0.1787	0.1398	-0.3319	-0.3209	0.0972	0.2289	-0.0221	0.0659	-0.4187	-0.3365	0.0672	0.2076	0.3243	0.256	0.3344	0.1728	0.2185
time constant (synapse)	-0.0234	0.0646	-0.1527	-0.38	0.0964	0.1844	-0.2691	0.1874	0.4066	-0.2199	0.0856	-0.33	-0.3177	-0.2397	0.4968	0.2609	0.0945	0.0648	0.0938	0.0378
immobile fraction (synapse)	-0.2415	0.058	-0.181	-0.0556	-0.0939	0.0733	0.1136	0.3233	0.1866	0.076	0.1358	9.04E-03	-0.0206	-0.2933	0.1078	-0.2645	-5.57E-03	-0.1759	-0.1933	0.1152

P values (soluble proteins)

	%Ala	%Arg	%Asn	%Asp	%Cys	%Gln	%Glu	%Gly	%His	%Ile	%Leu	%Lys	%Met	%Phe	%Pro	%Ser	%Thr	%Trp	%Tyr	%Val
time constant (axon)	0.8963	0.8627	0.1472	0.8513	0.9051	0.4132	0.928	0.2374	0.2079	0.5688	0.3133	0.5536	0.7281	0.8287	0.3169	0.8153	0.9191	0.177	0.6147	0.9881
immobile fraction (axon)	0.6419	0.471	0.7983	0.3628	0.478	0.0845	0.0959	0.6228	0.2414	0.911	0.7391	0.0266	0.0799	0.7342	0.2892	0.0922	0.1885	0.0819	0.3791	0.264
time constant (synapse)	0.9059	0.7442	0.4379	4.61E-02	0.6256	0.3477	0.1662	0.3397	0.0318	0.2609	0.6651	0.0864	0.0995	2.19E-01	7.16E-03	0.1799	0.6323	0.7431	6.35E-01	0.8485
immobile fraction (synapse)	0.2158	0.7692	0.3567	7.79E-01	0.6345	0.7109	0.5649	0.0933	0.3417	0.7005	0.4908	0.9636	0.9172	1.30E-01	0.5849	0.1739	0.9776	3.71E-01	0.3244	0.5595

correlation coefficients (membrane proteins)

	%Ala	%Arg	%Asn	%Asp	%Cys	%Gln	%Glu	%Gly	%His	%Ile	%Leu	%Lys	%Met	%Phe	%Pro	%Ser	%Thr	%Trp	%Tyr	%Val
time constant (axon)	-0.2891	0.1989	-0.0694	0.2542	-0.2201	-0.3643	-0.037	-0.1717	0.1871	0.1232	0.1358	0.2299	-0.0628	0.2731	-0.0929	-0.0469	-0.4022	0.1853	-0.2418	7.08E-02
immobile fraction (axon)	0.0312	-0.47	0.1424	-0.4894	0.6138	-0.3071	-0.2817	0.7148	-0.0411	-0.0693	-0.1246	-0.384	0.364	0.5023	-0.048	-0.1209	0.2124	0.4839	0.5625	-0.2911
time constant (synapse)	0.3376	-0.5524	-0.3517	-0.6418	0.1587	-0.2442	-0.3701	0.5763	0.201	-0.5671	0.0472	-0.3229	-0.3757	0.636	0.2818	-0.0915	-0.1714	0.3832	0.7897	0.4095
immobile fraction (synapse)	0.3472	-0.5082	-0.1242	-0.6124	0.3779	-0.3034	-0.5924	0.637	0.0557	-0.2284	-0.0848	-5.88E-01	0.036	0.8235	0.3058	-0.1813	-2.79E-01	0.7279	0.5804	0.1564

P values (membrane proteins)

	%Ala	%Arg	%Asn	%Asp	%Cys	%Gln	%Glu	%Gly	%His	%Ile	%Leu	%Lys	%Met	%Phe	%Pro	%Ser	%Thr	%Trp	%Tyr	%Val
time constant (axon)	0.3162	0.4955	0.8136	0.3804	0.4497	0.2004	0.9	0.5572	0.5219	0.6748	0.6435	0.4292	0.8311	0.3448	0.752	0.8736	0.154	0.5258	0.405	0.81
immobile fraction (axon)	0.9194	0.1051	0.6427	0.0896	0.0257	0.3075	0.3511	6.03E-03	0.8941	0.822	0.6851	0.1952	0.2215	0.0802	0.8762	0.694	0.486	0.0938	0.0454	0.3346
time constant (synapse)	0.185	0.0215	0.1663	5.48E-03	0.5431	0.345	0.1437	0.0155	0.4391	0.0176	0.8572	0.2062	0.1373	6.06E-03	2.73E-01	0.7269	0.5108	0.129	1.63E-04	0.1026
immobile fraction (synapse)	0.1722	0.0373	0.6349	8.97E-03	0.1348	0.2365	0.0122	5.96E-03	0.8319	0.3779	0.7463	0.0131	0.8909	4.86E-05	0.2326	0.4862	0.2788	9.25E-04	0.0146	0.5489

Appendix Fig. S11. Correlations between FRAP parameters and the amino acid composition of the proteins. Correlation coefficients and P values for mobility parameters versus the % of each amino acid in the protein sequences are shown. Statistically significant correlations (after correction for multiple testing) are underlined.

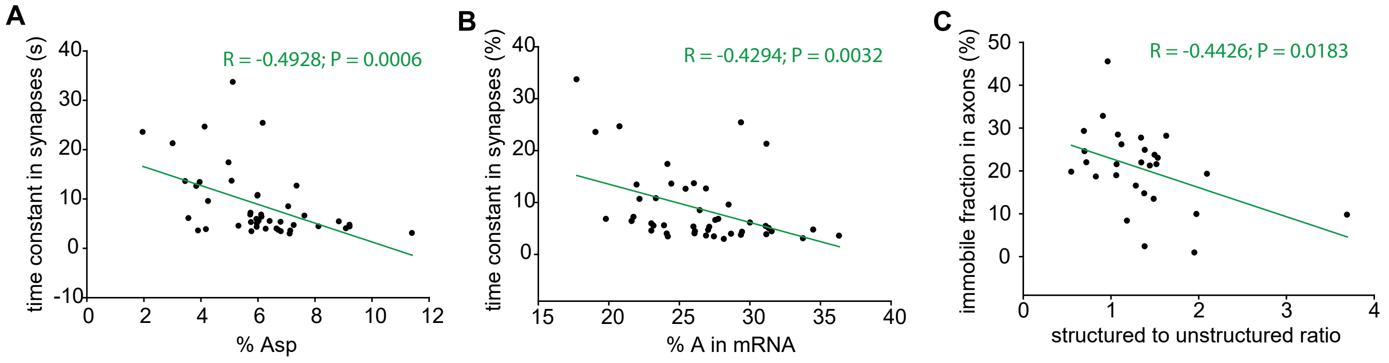

Appendix Fig. S12. Correlation of protein mobility parameters to different cell biology parameters. A, correlation between time constants in synapses (all proteins) and the percentage of aspartate residues in the protein sequence. **B**, correlation between time constants in synapses and the percentage of adenine in the mRNA sequences. **C**, correlations the immobile fraction in axons and structured-to-unstructured ratio (indicating the fraction of each protein sequence that is predicted to be structured, divided by the fraction that is predicted to be organized as random coils). The structural parameters were determined in Mandad et al., 2018.

	correlation coefficients (all proteins)			
	time constant (axon)	immobile fraction (axon)	time constant (synapse)	immobile fraction (synapse)
%A	0.2017	-5.05E-01	-4.29E-01	-0.2503
%C	-0.2156	4.14E-01	0.2356	0.0383
%G	-0.2056	-0.0926	-0.021	-0.1132
%U	0.241	0.1908	0.2827	3.93E-01

	correlation coefficients (soluble proteins)			
	time constant (axon)	immobile fraction (axon)	time constant (synapse)	immobile fraction (synapse)
%A	-0.2067	-0.4562	-0.4774	0.0485
%C	0.2265	0.2947	0.5332	-0.0606
%G	0.014	-0.1951	-0.1356	0.0549
%U	-0.0727	0.3509	0.0105	-0.0398

	correlation coefficients (membrane proteins)			
	time constant (axon)	immobile fraction (axon)	time constant (synapse)	immobile fraction (synapse)
%A	0.359	-0.5079	-0.6115	-0.5055
%C	-0.4523	0.4972	0.3974	0.2829
%G	-0.439	-0.0483	0.3256	-0.1117
%U	0.4198	0.1852	0.258	0.6008

	P values (all proteins)			
	time constant (axon)	immobile fraction (axon)	time constant (synapse)	immobile fraction (synapse)
%A	0.2003	7.68E-04	3.25E-03	0.0973
%C	0.1703	7.08E-03	0.1193	0.8027
%G	0.1915	0.5646	0.891	0.459
%U	0.1242	0.2321	0.0599	7.52E-03

	P values (soluble proteins)			
	time constant (axon)	immobile fraction (axon)	time constant (synapse)	immobile fraction (synapse)
%A	0.2914	1.47E-02	1.02E-02	0.8062
%C	0.2465	1.28E-01	3.48E-03	0.7593
%G	0.9435	0.3197	0.4916	0.7813
%U	0.7132	0.0671	0.9578	8.41E-01

	P values (membrane proteins)			
	time constant (axon)	immobile fraction (axon)	time constant (synapse)	immobile fraction (synapse)
%A	0.2075	0.0764	9.09E-03	0.0384
%C	0.1044	0.0838	0.1142	0.2713
%G	0.1163	0.8754	0.2021	0.6695
%U	0.1351	0.5448	0.3174	0.0108

Appendix Fig. S13. Correlations between FRAP parameters and nucleotide composition of proteins' mRNAs. Correlation coefficients and P values for mobility parameters versus the % percentage of each nucleotide in the mRNA sequences are shown. Statistically significant correlations (after correction for multiple testing) are underlined.

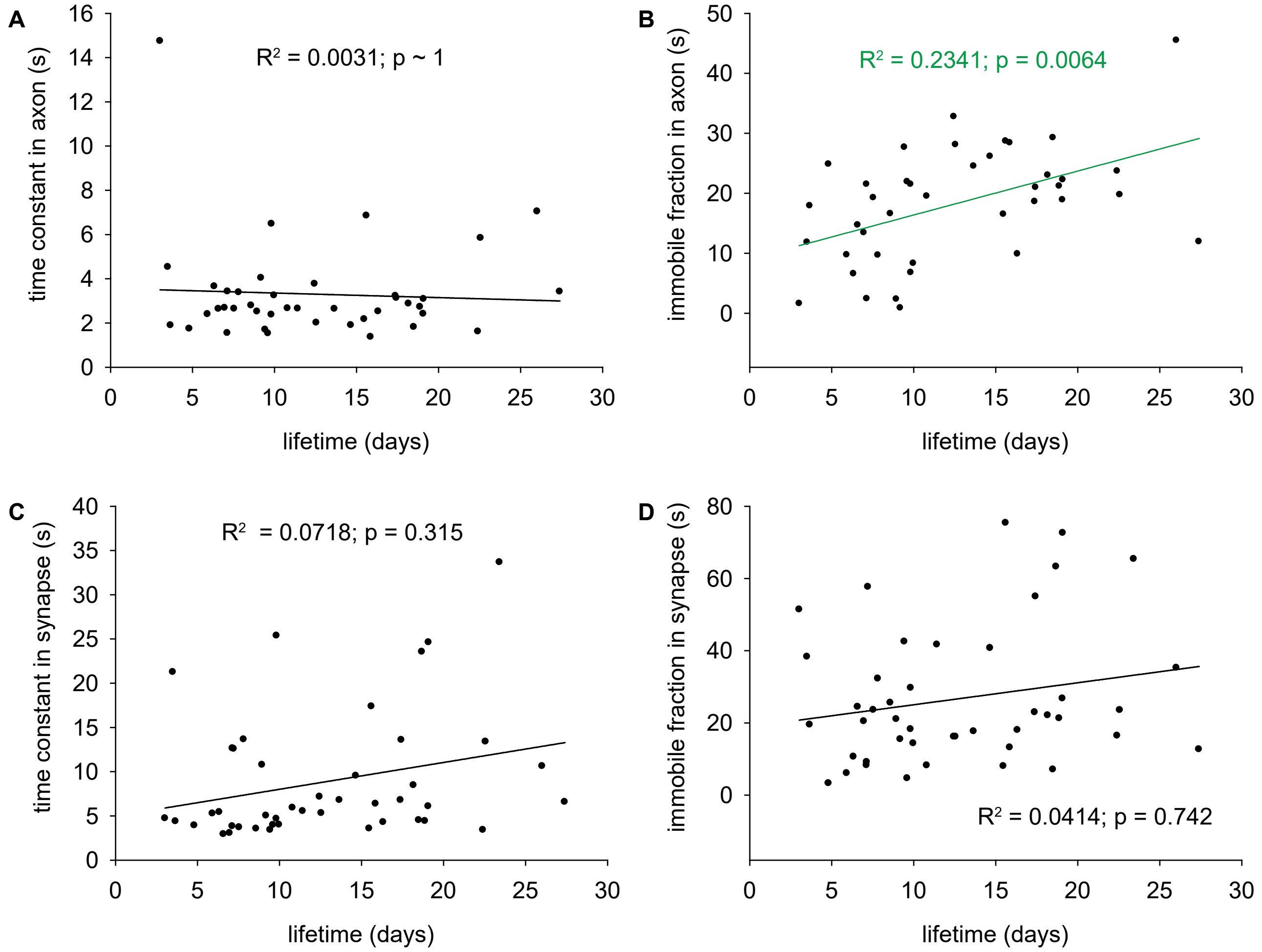

Appendix Fig. S14. A comparison of protein movement parameters to protein lifetimes. We plotted here the lifetimes of the proteins we analyzed (measured in vivo by Fornasiero et al., 2018) against the FRAP time constants and the immobile fractions measured in axons and synapses. A significant correlation was found between the axonal immobile fraction and the protein lifetime ($P < 0.01$, after a Bonferroni correction for multiple testing). Panel B is also shown in Fig. 4 F.

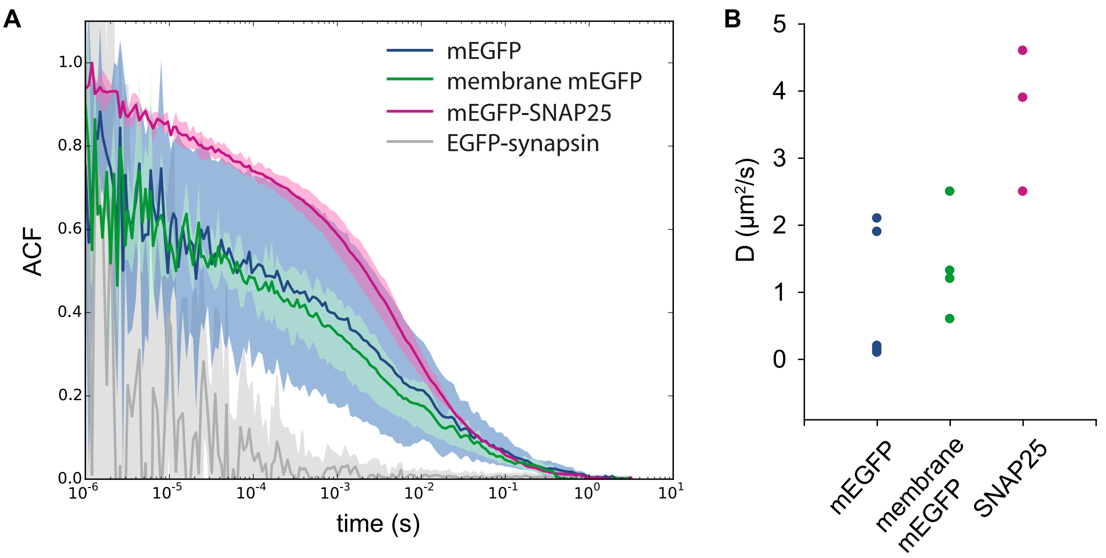

Appendix Fig. S15. FCS experiments for determining protein mobility in synapses and axons.
FCS experiments were conducted on neurons expressing mEGFP-SNAP25, EGFP-synapsin, soluble mEGFP or membrane-anchored mEGFP. The acquired autocorrelation curves were fitted using a single- or a dual-component model for particles diffusing in 2D (Lakowicz, 2006). **A,** the normalized average curves for each of the four expressed proteins. **B**, average diffusion coefficients determined at each measurement position for each protein. Diffusion was measured at 5, 4, and 3 different positions inside axons for mEGFP, membrane mEGFP and SNAP25 respectively. Each position was measured at least 20 times, with acquisition times between 10 and 30 s for each round of acquisition. For EGFP-synapsin the effects of bleaching and high density of the molecules dominate the measurement, implying that a reasonable correlation of the data is impossible. For membrane EGFP the measured diffusion coefficient is slightly higher than free mEGFP, 1.4 ± 0.4 $\mu m^2/s$ (the coefficient for free mEGFP was 0.9 ± 0.4 $\mu m^2/s$). For mEGFP-SNAP25 we measured a diffusion coefficient of 3.7 ± 0.6 $\mu m^2/s$. These values are surprising, since the membrane-attached mEGFP-SNAP25 as well as membrane mEGFP are expected to diffuse considerably slower than free mEGFP in the cytosol. At the same time, SNAP25 value is ~15-fold higher than expected from previous measurements, at ~0.24 $\mu m^2/s$ (for measurements in neuroendocrine PC12 cells (Knowles et al., 2010)).

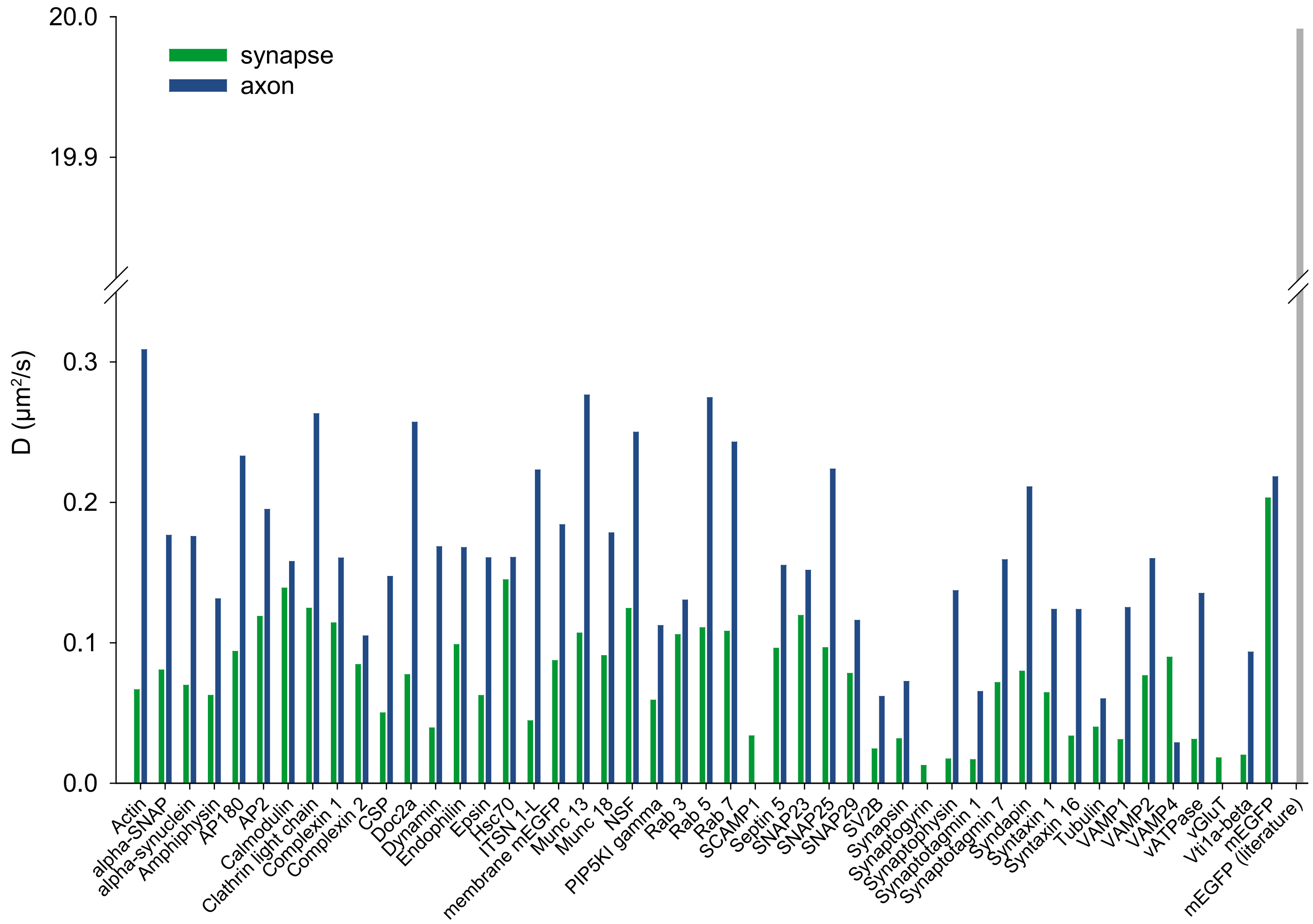

Appendix Fig. S16. Calculation of protein diffusion coefficients using a simple FRAP interpretation model. We applied a previously published equation dedicated to FRAP interpretation (Kang et al., 2012) to our dataset. The diffusion coefficients are shown here. A number of problems are evident. For example, the equation underestimates the diffusion coefficient of mEGFP by ~100 fold compared to the diffusion coefficient measured in eukaryotic cells (Sadovsky et al., 2017). Also, there is no evident difference between soluble and membrane proteins. Finally, free mEGFP is expected to be the fastest-moving molecule, but this does not seem to be the case when using this interpretation model.

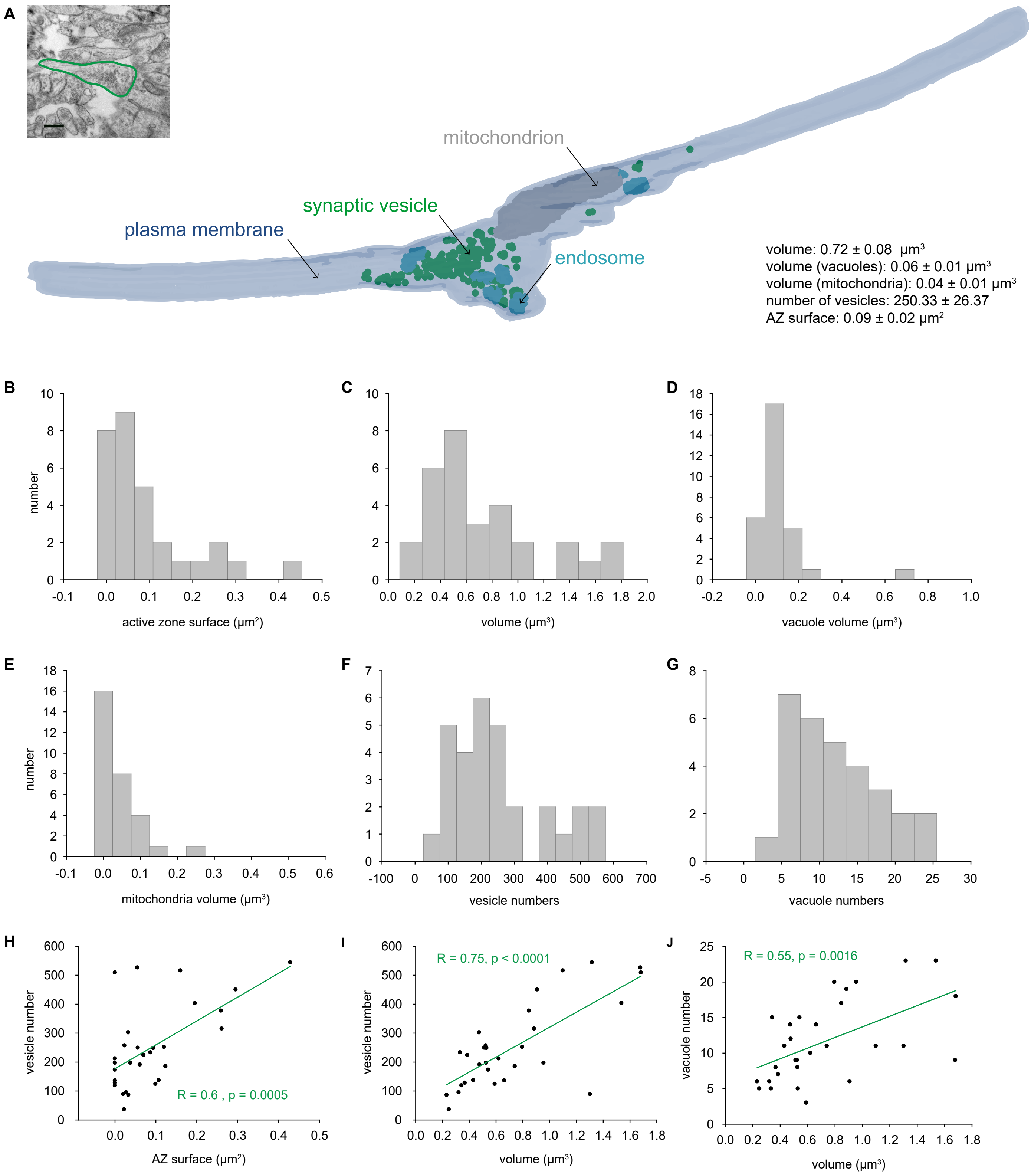

Appendix Fig. S17. 3D model of a synapse, and synaptic parameters measured from serial-section electron microscopy. A, A view of the 3D synapse used in this work. The inset shows one EM section of the selected synapse. Scale bar, 500 nm. Average measured parameters ± SEM shown in the right lower corner of the panel. We reconstructed 30 synapses from series of 70 nm-thick sections. Their various parameters, including active zone surface area, total volume, vacuole (endosome) total volume, mitochondria volume, vesicle numbers, and vacuole (endosome) numbers are indicated in the form of histograms (**B**-**G**), or as scatter plots (**H**-**J**). As expected from the previous literature (Murthy et al., 2001; Schikorski and Stevens, 1997, 2001), the different parameters correlate to each other. For example, the vesicle numbers correlate to the active zone surface area (**H**), and the synaptic volume also correlates to the vacuole and vesicle numbers (**I**-**J**).

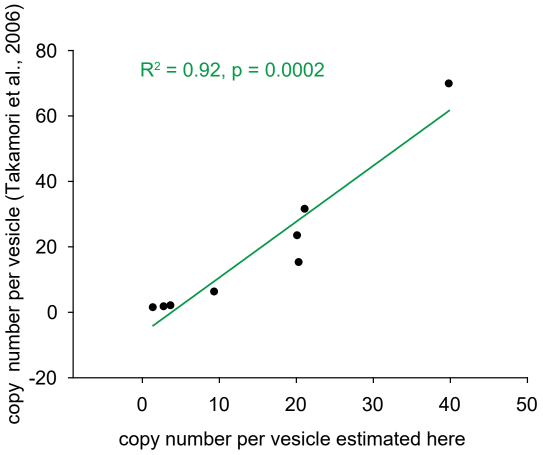

Appendix Fig. S18. The *in silico* models, in combination with the available literature, suggest accurate numbers of synaptic vesicle proteins per vesicle. The copy numbers of synaptic vesicle proteins have been estimated in a seminal paper in 2006 (Takamori et al., 2006) , and have been later confirmed in a further quantitative study (Wilhelm et al., 2014). To test whether our in silico models confirm these measured values, we proceeded as follows. We first estimated the numbers of proteins present in the synapto-axonal compartment, relying on values published in Wilhelm et al., 2014, adjusted to hippocampus neurons following the method of Richter et al., 2018. We then extracted from the models the fraction of proteins that is predicted to be present in the synapses. This parameter was already used in Fig. 4A-C. We then proceeded for *bona fide* synaptic vesicle proteins as follows. The fraction of each protein found on the synaptic plasma membrane or in vesicles can be obtained from the literature (Bodzęta et al., 2017; Granseth et al., 2006; Hoopmann et al., 2010; Voglmaier et al., 2006), which enables us to easily correct the previous parameter to determine the total copy numbers present in the vesicle population in the model synapse. Finally, these numbers can be divided by the number of vesicles in the model, to obtain a predicted copy number per synaptic vesicle. This value correlates excellently with the value from Takamori et al., 2006 ($R^2 > 0.9$)

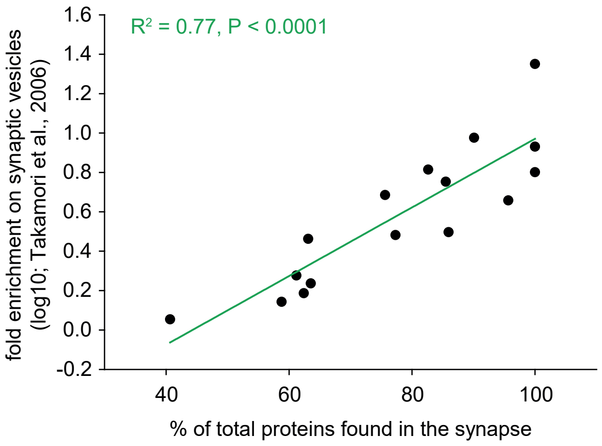

Appendix Fig. S19. The *in silico* models suggest accurate enrichment of the proteins in the vesicles. Our movement models enabled us to calculate easily the fraction of proteins found in the synapse, in comparison to the axon regions near the synapse, for all membrane proteins. We plotted this on the X axis, and compared it to the enrichment of the respective proteins in synaptic vesicles vs. the brain homogenate fraction as measured by Takamori et al., 2006.

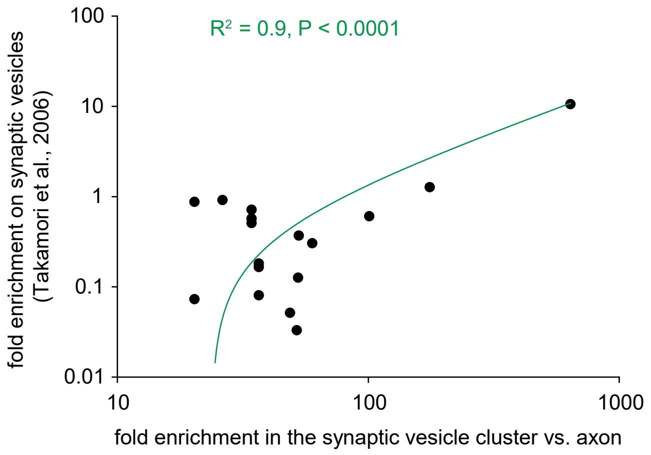

Appendix Fig. S20. The *in silico* models suggest accurate enrichment of soluble proteins in the synaptic vesicle cluster. We calculated the enrichment of soluble proteins in the vesicle cluster compared to the axon as predicted by our model. The values correlate well with the enrichment on synaptic vesicles vs. the brain homogenate fraction as measured by Takamori et al., 2006.

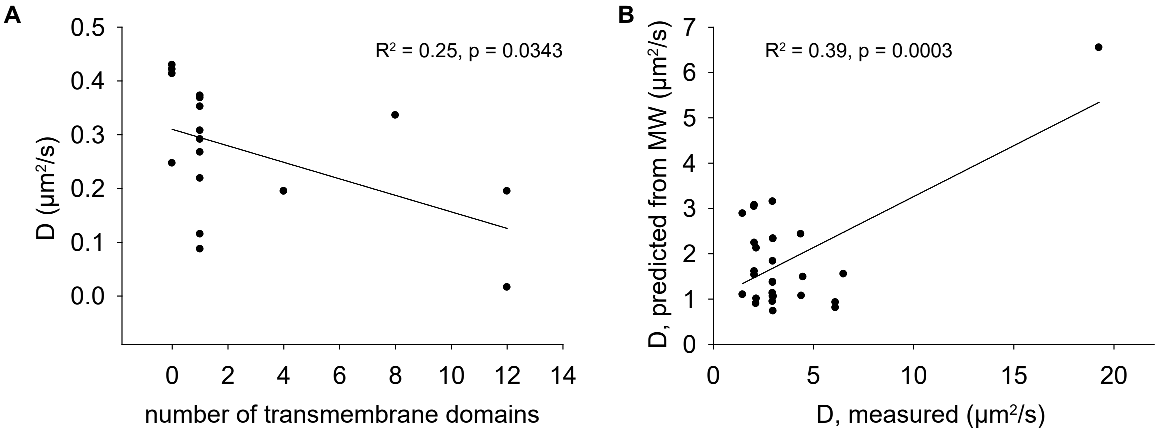

Appendix Fig. S21. An analysis of basic movement parameters of the proteins. A, We compared the diffusion coefficients obtained in the axons with the number of transmembrane domains, for the different membrane proteins. A significant, albeit not very strong, correlation can be observed, which agrees with the previous literature, and with the expectation that proteins with large numbers of membrane domains diffuse more slowly. **B**, We compared the diffusion coefficients obtained in the axons for soluble proteins with the ideal diffusion coefficients for the respective proteins, predicted from their molecular weights (according to Kumar et al., 2010). A significant correlation was obtained, which is again in agreement with the previous literature. If soluble EGFP is removed (upper right spot), the correlation loses significance, implying that the soluble proteins do not, in general, move according to their mass in the synaptic system.

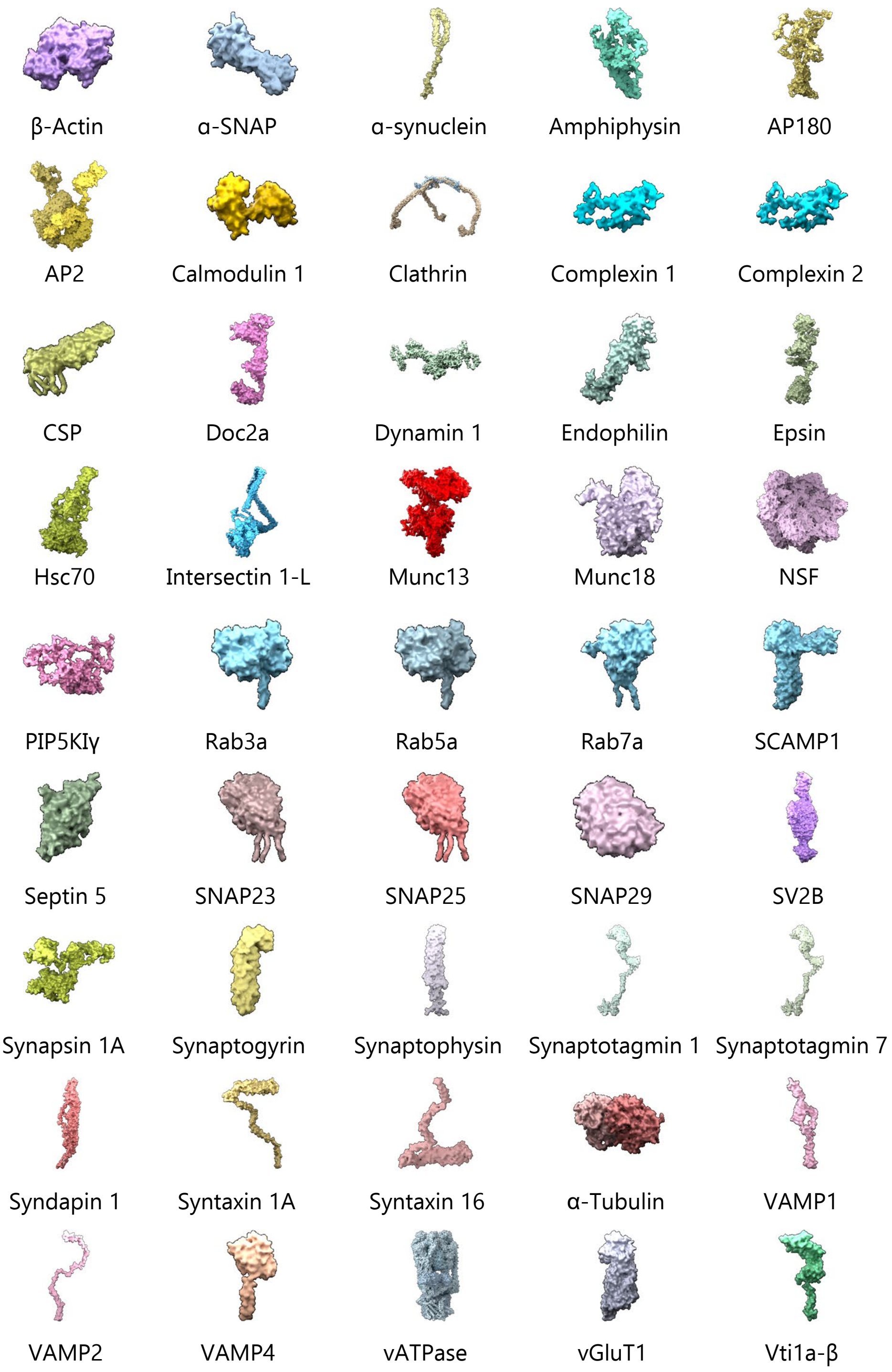

Appendix Fig. S22. The shapes and colors of the proteins shown in the Movies EV1-4. The proteins are not show to scale (the sizes are automatically scaled for all).
